# Metal-Catalyzed Hydrogen Atom Transfer (MHAT) Hydroalkylation with Electron-Deficient Alkynes

**DOI:** 10.1021/acs.orglett.4c03943

**Published:** 2024-11-28

**Authors:** Laura G. Rodríguez, Josep Bonjoch, Ben Bradshaw

**Affiliations:** Laboratori de Química Orgànica, Facultat de Farmàcia, IBUB, Universitat de Barcelona, Barcelona 08028, Spain

## Abstract

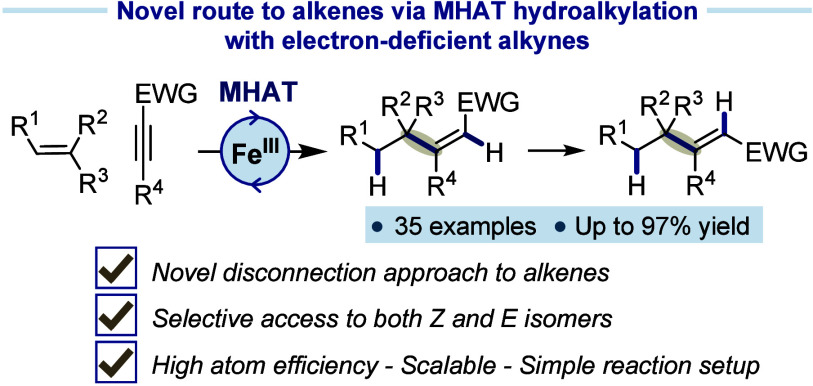

We present a novel strategy for olefin construction via the reductive coupling of electron-neutral alkenes with electron-deficient alkynes under metal-catalyzed hydrogen atom transfer conditions. This methodology provides selective access to both *trans* and the more challenging-to-synthesize *cis* isomers and permits the olefin to be installed next to sterically hindered centers, key factors in the synthesis of biologically active compounds. The reaction exhibits broad functional group tolerance and proceeds under mild, nontoxic conditions with high atom efficiency.

The direct addition of alkyl radicals to carbon–carbon π bonds is one of the most widely employed reactions in radical chemistry.^1^ A prominent variant of these additions is the Giese reaction, in which a nucleophilic radical intermediate^2^ adds to an electron-deficient π bond, constituting a formal conjugate addition process (Figure 1A).^3^

**Figure 1:**
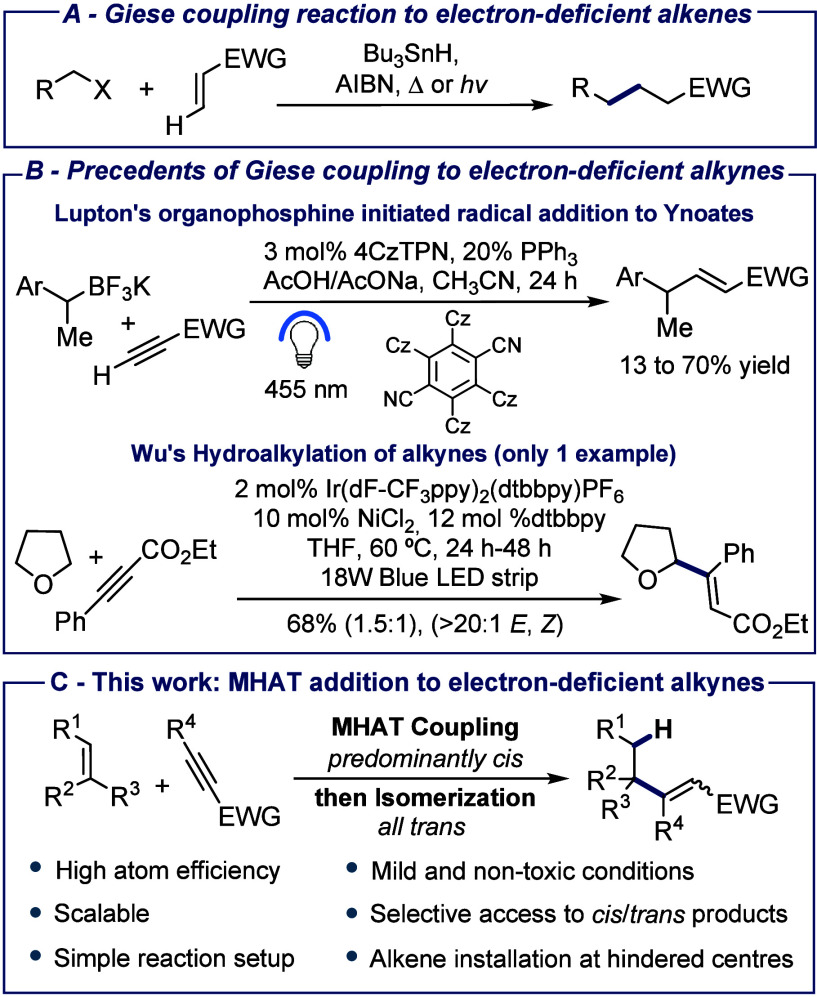
Giese addition to electron-deficient alkenes and alkynes.

For many years, these reaction mechanisms have offered distinct advantages over organometallic conjugate addition reactions, particularly when the corresponding organometallic reagent is challenging to synthesize or prone to instability, such as with secondary, tertiary, or heteroatom-stabilized substrates.^4^ Moreover, radical processes generally proceed under milder conditions and exhibit greater functional group tolerance, which can streamline the synthesis of complex molecules by avoiding the need for extensive protection strategies.^5^

Traditionally, Giese reactions have relied primarily on toxic tin hydrides, which generate neurotoxic and difficult-to-separate organotin residues, limiting the reach and utility of this process, particularly in pharmaceutical chemistry.^6^ The demand for safe, environmentally benign technologies has consequently driven the development of methods to overcome these limitations.^7^ However, despite significant advances, reports of Giese-type couplings to electron-deficient alkynes remain scarce (Figure 1B). Among the few examples described in the literature, Lupton reported a radical coupling initiated by organophospine addition to an ynoate under photochemical conditions.^8^ The addition of phosphine generates a more readily reducible species that would undergo radical–radical coupling with a radical generated from trifluoroborate derivatives. However, the reaction was limited to boronates bearing at least one aromatic group. In another example, Wu developed a metallaphotoredox approach for alkyne coupling. However, coupling with an electron-deficient alkyne was demonstrated in only a single reaction and gave a nonregioselective radical fragment coupling.^9^

As part of our efforts to develop new metal-catalyzed hydrogen atom transfer (MHAT) reactions,^10^ our group has designed several radical coupling reactions to form carbon–heteroatom π bonds, including additions to ketones,^11^ aldehydes,^12^ Cbz-hydrazones,^13^ Ts hydrazones,^14^ and isocyanides.^15^ Seeking to extend this work to the formation of carbon–carbon π bonds, we hypothesized that MHAT chemistry could serve as an ideal platform to address the scarcity of precedents in the area of alkyne couplings, providing a valuable addition to the synthetic toolbox. Specifically, developing a Giese addition to electron-deficient alkynes would offer distinct advantages over the classical Giese reaction.^16^ The double bond retained in the product could act as a key structural motif in numerous biologically active natural products or as a versatile handle for further functionalization, allowing the synthesis of value-added compounds.

Herein, we describe a method for the reductive coupling of electron-neutral alkenes with electron-deficient alkynes under MHAT conditions, enabling the formation of *sp*^3^–*sp*^2^ bonds and selectively generating both *trans* and the more challenging-to-synthesize *cis* isomers (Figure 1c).^17^ The reaction is straightforward to set up, demonstrates broad functional group tolerance for both the alkene donor and the alkyne acceptor, permits the olefin to be installed next to sterically hindered centers,^18^ and proceeds under mild, nontoxic conditions with high atom efficiency.

At the outset, we identified two key challenges: controlling the alkene geometry and preventing the coupled product from undergoing further reactions such as being reduced by the metal hydride species or acting as an acceptor in the reaction itself via a second Giese-type process.

We began by studying the reaction of alkene **1a** and methyl propiolate **2a**. Upon optimization, we achieved a 97% yield for the coupled product, predominantly the *cis* isomer **3a**, using 3.0 equiv of alkene donor **1a**, stoichiometric amounts of Fe(acac)_3_ and acceptor **2a**, and 1.5 equiv of PhSiH_3_ in EtOH at 60 °C for 16 h (Table 1, entry 1). This reaction was carried out on a 4.0 mmol scale, demonstrating the scalability of the method (for comparison, on a 0.4 mmol scale, a 95% yield was obtained). The *trans* isomer **4a** was also obtained as a minor component of the reaction and could be readily separated by column chromatography. During these optimization studies, we found that decreasing the donor amount (entry 2), changing the solvent (entries 3 and 4), increasing the amount of phenylsilane (entry 5), using a lower temperature (entry 6), increasing the amount of the acceptor (entry 7), shortening the reaction time (entry 8), working under open air conditions (entry 9), or using a combination of Fe(acac)_3_ and Fe(acac)_2_ catalysts (entry 10) was detrimental to the reaction outcome, resulting in either lower yields or significant amounts of reduced compound **5**. Although 3.0 equiv of **1a** were optimal, comparable results were obtained using 2 equiv of **1a** (entry 11), providing a cost-effective alternative that minimizes the use of the donor alkene. We envisaged that it might be possible to reduce the amount of **1a** used by introducing an electron-deficient alkene as a potential scavenger of any excess metal-hydride species. However, when we performed a competition experiment by the addition of 1.0 equiv of ethyl acrylate, we observed that electron-deficient alkenes coupled at the same rate as their alkyne counterparts, yielding 50% of each coupling product (entry 12). We also tested the addition of various alkene additives as radical scavengers to minimize overreduction. After extensive experimentation (see Supporting Information), the best result was obtained using 1.0 equiv of 2-methyl-2-butene (entry 13). Although the results did not match the optimized yield, these conditions may serve as useful alternatives when the alkene donor is particularly valuable.

**Table 1:**
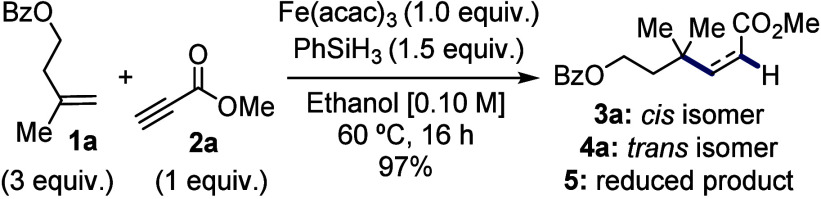
Optimization of the MHAT Reaction

Entry^a^	Deviation from optimum conditions	Ratio^b^**3a**/**4a**/**5**	Yield^c^ (%)
1	No deviation	62/35/3	97
2	1 equiv of **1a**	53/30/17	74
3	THF/MeOH 9:1 as solvent^d^	50/35/15	64
4	DCE as solvent^d^	53/38/9	52
5	2.5 equiv of PhSiH_3_^d^	50/32/18	71
6	25 °C instead of 60 °C^d^	41/39/20	55
7	2 equiv of **2a**^d^	50/31/19	69
8	3 h instead of 16 h	62/34/4	84
9	Open air conditions	52/36/12	68
10	0.5 equiv of Fe cat. as Fe(acac)_2_	63/31/6	87
11	2 equiv of **1a**	63/33/4	92
12	1 equiv of ethyl acrylate^d^	42/29/29	50^e^
13	1 equiv of 2-methyl-2-butene^d^	60/32/8	69

a All reactions were carried out on a 0.40 mmol scale except entry 1, which was carried out on a 4.0 mmol scale (0.40 mmol scale: 95% yield).

b Ratio refers to the proportion of **3a**/**4a**/**5** determined by NMR after isolation.

c Combined yield of **3a** and **4a**.

d 1 equiv of **1a**.

e 50% yield of the ethyl analogue of **5** was also isolated.

Isomerization of the alkene mixture of **3a** and **4a** could be achieved in almost quantitative yield by adding 2.0 equiv of thiophenol and 1.0 equiv of triethylamine at 40 °C under solvent-free conditions, followed by treatment with sodium periodate.^19^ This provided **4a** in 94% yield for the overall process starting from alkene **1a**.

With the optimal conditions established, we then explored the reaction scope (Scheme 1). The reaction demonstrated broad functional group tolerance, successfully coupling terminal 2-methyl alkenes **1a**–**1e**, containing ester, phthalimide, carbamate and sulfonyl groups, to form the *cis* compounds **3a**–**3e** as major products. The more sterically congested alkene **1f** also delivered coupled product **3f**. Additionally, terminal monosubstituted alkenes **1g**–**1h** produced the corresponding products **3g**–**3h**. Notably, as the carbon chain lengthened and the protecting group was positioned further from the reactive center, the *trans* isomer became the predominant product. In these cases, a slight increase in the amount of the reduced compound was also observed, likely due to less steric hindrance around the alkene.

**Scheme 1:**
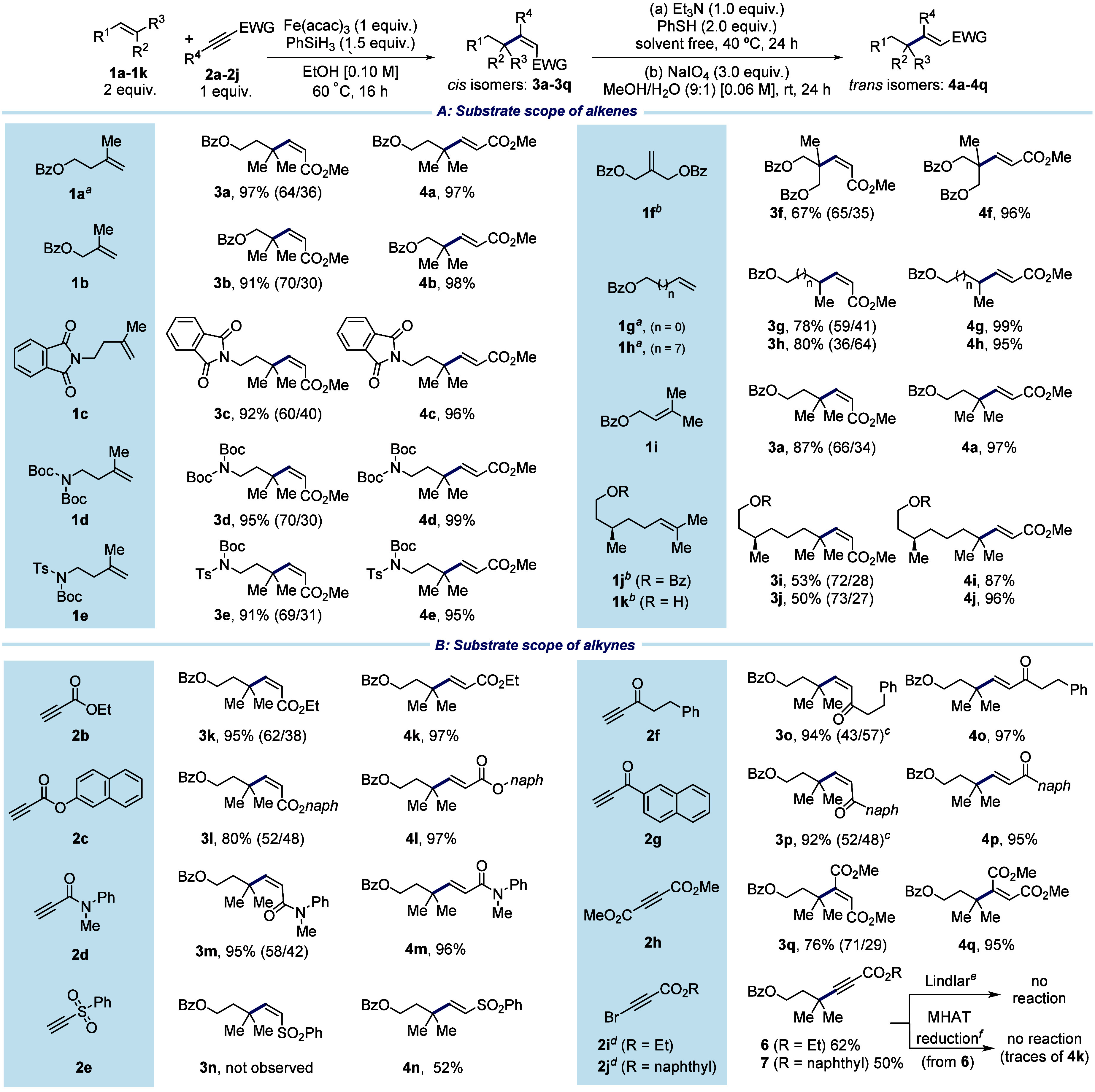
Scope of the Coupling of Unbiased Alkenes to Electron-Deficient Alkynes 3.0 equiv of donor were used. 5.0 equiv of donor and 0.5 equiv of PhSiH_3_ were used The *cis* product spontaneously isomerized, so the mixture was directly subjected to isomerization conditions. 1.0 equiv of NaHCO_3_ was added. Conditions: 0.20 equiv of Lindlar catalyst, 0.15 equiv of quinoline, MeOH (0.12 M), 25 °C, 24 h. Conditions: 1.0 equiv of Fe(acac)_3_, 2.5 equiv of PhSiH_3_, EtOH [0.10 M], 60 °C, 16 h.

Trisubstituted alkenes also proved to be viable substrates for coupling, with alkene **1i** forming **3a** in a similar way to the exocyclic alkene **1a**. Similar trisubstituted alkenes, both with and without alcohol protection, yielded products **3i** and **3j**, respectively, though in moderate yields, which was attributed to the more remote location of the unsaturation. Importantly, all *cis* products could be isomerized to their corresponding *trans* compounds **4a**–**4j** in nearly quantitative yields using the conditions developed. Next, we explored the scope of electron-deficient alkynes. Alkyne esters **2b** and **2c**, bearing ethyl and naphthyl groups, gave rise to products **3k** and **3l**, which could also be isomerized to the corresponding *trans* products **4k** and **4l**, again with nearly quantitative yields. Amide **2d** proved to be a competent acceptor, furnishing product **3m**. In contrast, with sulfonamide **2e**, the *cis* compound **3n** was not observed and only the *trans* product **4n** was detected, and it could not be ascertained whether **3n** did not form or isomerized under the reaction conditions or upon purification. However, ketones **3o** and **3p** derived from **2f** and **2g**, respectively, did undergo spontaneous isomerization to give **4o** and **4p**, indicating that the latter is more likely. Nevertheless, the *cis* compounds could be observed by NMR, and tentative proportions are provided after purification. Internal alkynes with a methyl ester and methyl or phenyl groups did not participate in the coupling reaction. However, incorporating a second ester group led to a successful coupling reaction between **1a** and **2h**, yielding product **3q** and in turn **4q** in good yield. Finally, we evaluated the reaction of alkynyl bromides using the conditions developed by Cui.^20^ While Cui’s work was limited to aromatic substituents, we found that the presence of an electron-withdrawing group on the bromoalkyne was also feasible, with **2i** and **2j** giving the alkynes **6** and **7**, respectively, via an addition–substitution mechanism rather than a Giese pathway. While an alternative route to selectively access the corresponding *cis* and *trans* alkenes **3k** and **4k** via a divergent reduction process of **6** could be envisioned, no reaction was observed under Lindlar reduction, and although MHAT reductive conditions favored the *trans* isomer, only trace amounts were obtained even after extended reaction times. These observations underscore the importance of our direct strategy for effectively accessing the targeted alkene products.

The proposed mechanism for the reaction is outlined in Figure 2a. Formation of the iron hydride species Fe(acac)_2_H **I** and hydrogen atom transfer to alkene **1a** generates the carbon-centered radical **II**.^16d^ This species then adds to electron-deficient alkyne **2a**, yielding vinyl radical **IV**. A proton-coupled electron-transfer (PCET)^21^ from Fe(acac)·EtOH follows, resulting in the formation of either **3a** or **4a**. The preference for the *cis* isomer **3a** is due to the occurrence of PCET to the more accessible face of **IV**. In contrast, formation of **4a** requires addition to the more hindered face of the molecule and is therefore unfavored. Carrying out the reaction in EtOD resulted in only 74% deuterium incorporation into the *cis* isomer and 69% into the *trans* isomer (Figure 2b). This suggests the involvement of an alternative pathway in which vinyl radical **IV** abstracts a hydrogen atom from either phenylsilane or metal hydride species **I**. Notably, this alternative pathway inhibits regeneration of the active catalytic species, which would explain why the reaction generally performed better with higher loadings of Fe(acac)_3_. Once the coupled products **3a** and **4a** are formed, they can be reduced by any hydride **I** present in the medium. To determine the source of reduced compound **5**, we exposed each isomer individually to the reaction conditions (Figure 2c). The pure *trans* alkene **4a** resulted in only a 10% reduction, whereas *cis* alkene **3a** was reduced more readily, yielding 57% of **5**. This indicates that **3a** was the primary source of **5** in the coupling reaction. However, these undesired reactions can be kept to a minimum by using an excess of the donor alkene.

**Figure 2:**
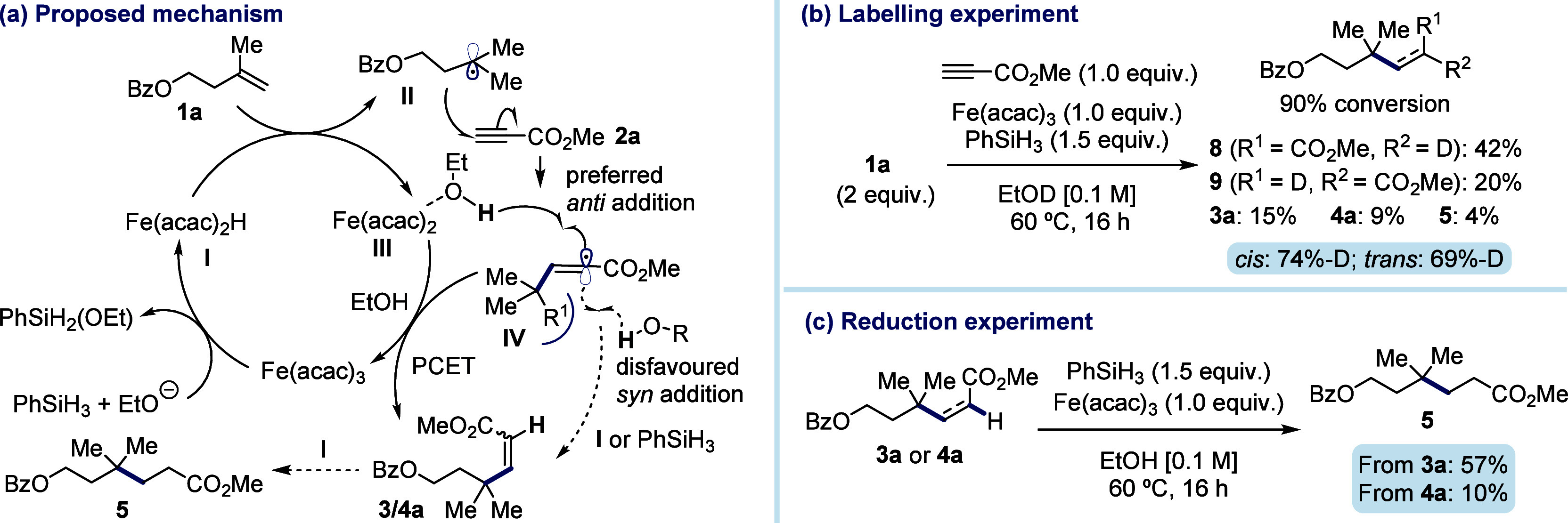
Proposed mechanism and supporting studies.

In conclusion, we have developed an approach to the Giese-type addition to electron-deficient alkynes under MHAT coupling conditions. This extends the synthetic toolkit for accessing structurally diverse alkenes and offers an alternative and complementary disconnection strategy to traditional olefination methods such as Horner–Wadsworth–Emmons and Still–Gennari olefination reactions. Moreover, with its mild conditions, high atom efficiency, and ability to install unsaturations adjacent to sterically congested centers, we anticipate that this methodology will find broad application in the synthesis of both small molecules and structurally complex, biologically active compounds.

## Data Availability

The data underlying this study are available in the published article and its Supporting Information.

## Supporting Information Available

General information, optimization of MHAT coupling, isomerization studies, Experimental Section, copies of NMR spectra (PDF)
FAIR data, including the primary NMR FID files, for compounds **1a**–**1j**, **2c**–**2g**, **2i**–**2j**, **3a**–**3m**, **3q**, **4a**–**4q**, **5**–**7**, **SI**-**A** and **SI-B** (ZIP)

## References

[ref1] aSilvi M.; Aggarwal V. K. Radical Addition to Strained σ-Bonds Enables the Stereocontrolled Synthesis of Cyclobutyl Boronic Esters. J. Am. Chem. Soc. 2019, 141, 9511–9515. 10.1021/jacs.9b03653. 31142107

[ref2] Garwood J. J. A.; Chen A. D.; Nagib D. A. Radical Polarity. J. Am. Chem. Soc. 2024, 146, 28034–28059. 10.1021/jacs.4c06774 PMC12129049 39363280

[ref3] aGiese B. Formation of CC Bonds by Addition of Free Radicals to Alkenes. Angew. Chem., Int. Ed. Engl. 1983, 22, 753–764. 10.1002/anie.198307531.

[ref4] aDieter R. K.; Alexander C. W.; Nice L. E. Conjugate Addition Reactions of α-Aminoalkylcuprates with α,β-Enones and Enals. Tetrahedron. 2000, 56, 2767–2778. 10.1016/S0040-4020(00)00130-7.

[ref5] Young I. S.; Baran P. S. Protecting-group-free synthesis as an opportunity for invention. Nat. Chem. 2009, 1, 193–205. 10.1038/nchem.216. 21378848

[ref6] Baguley P. A.; Walton J. C. Flight from the Tyranny of Tin: The Quest for Practical Radical Sources Free from Metal Encumbrances. Angew. Chem., Int. Ed. 1998, 37, 3072–3082. 10.1002/(SICI)1521-3773(19981204)37:22<3072::AID-ANIE3072>3.0.CO;2-9. 29711327

[ref7] Gant Kanegusuku A. L.; Roizen J. L. Recent Advances in Photoredox-Mediated Radical Conjugate Addition Reactions: An Expanding Toolkit for the Giese Reaction. Angew. Chem., Int. Ed. 2021, 60, 21116–21149. 10.1002/anie.202016666. PMC8382814 33629454

[ref8] Cao J.; Seitz A.; Forni J. A.; Polyzos A.; Lupton D. W. Radical Coupling Initiated by Organophosphine Addition to Ynoates. Angew. Chem., Int. Ed. 2023, 62, e202303869 10.1002/anie.202303869. 37188643

[ref9] Deng H. P.; Fan X. Z.; Chen Z. H.; Xu Q. H.; Wu J. Photoinduced Nickel-Catalyzed Chemo- and Regioselective Hydroalkylation of Internal Alkynes with Ether and Amide α-Hetero C(sp3)–H Bonds. J. Am. Chem. Soc. 2017, 139, 13579–13584. 10.1021/jacs.7b08158. 28862448

[ref10] aMukaiyama T.; Yamada T. Recent Advances in Aerobic Oxygenation. Bull. Chem. Soc. Jpn. 1995, 68, 17–35. 10.1246/bcsj.68.17.

[ref11] aSaladrigas M.; Bosch C.; Saborit G. V.; Bonjoch J.; Bradshaw B. Radical Cyclization of Alkene-Tethered Ketones Initiated by Hydrogen-Atom Transfer. Angew. Chem., Int. Ed. 2018, 57, 182–186. 10.1002/anie.201709659. 29115722

[ref12] Saladrigas M.; Puig J.; Bonjoch J.; Bradshaw B. Iron-Catalyzed Radical Intermolecular Addition of Unbiased Alkenes to Aldehydes. Org. Lett. 2020, 22, 8111–8115. 10.1021/acs.orglett.0c03081. 33017537

[ref13] Saladrigas M.; Loren G.; Bonjoch J.; Bradshaw B. Hydrogen Atom Transfer (HAT)-Triggered Iron-Catalyzed Intra- and Intermolecular Coupling of Alkenes with Hydrazones: Access to Complex Amines. ACS Catal. 2018, 8, 11699–11703. 10.1021/acscatal.8b03794.

[ref14] Saladrigas M.; Bonjoch J.; Bradshaw B. Iron Hydride Radical Reductive Alkylation of Unactivated Alkenes. Org. Lett. 2020, 22, 684–688. 10.1021/acs.orglett.9b04459. 31887044

[ref15] Puig J.; Bonjoch J.; Bradshaw B. Isocyanides as Acceptor Groups in MHAT Reactions with Unactivated Alkenes. Org. Lett. 2023, 25, 6539–6543. 10.1021/acs.orglett.3c02358. 37644914 PMC10496133

[ref16] aLo J. C.; Yabe Y.; Baran P. S. A Practical and Catalytic Reductive Olefin Coupling. J. Am. Chem. Soc. 2014, 136, 1304–1307. 10.1021/ja4117632. 24428607 PMC3971728

[ref17] One of the few reliable methods to form electron-deficient Z-alkenes is the Still–Gennari olefination, which has a different disconnection approach and usually requires the use of strong bases such as KHMDS and synthesis of the phosphonate precursor: Janicki I.; Kiełbasiński P. Still–Gennari Olefination and its Applications in Organic Synthesis. Adv. Syn. Catal. 2020, 362, 2552–2596. 10.1002/adsc.201901591.

[ref18] aTalele T. T. Natural-Products-Inspired Use of the Gem -Dimethyl Group in Medicinal Chemistry. J. Med. Chem. 2018, 61, 2166–2210. 10.1021/acs.jmedchem.7b00315. 28850227

[ref19] aHuang X.; David E.; Jubault P.; Besset T.; Couve-Bonnaire S. Organocatalyzed Sulfa-Michael Addition of Thiophenols on Trisubstituted α-Fluoroacrylates, a Straightforward Access to Chiral Fluorinated Compounds. J. Org. Chem. 2020, 85, 14055–14067. 10.1021/acs.joc.0c02081. 33054226

[ref20] Shen Y.; Huang B.; Zheng J.; Lin C.; Liu Y.; Cui S. Csp–Csp3 Bond Formation via Iron(III)-Promoted Hydroalkynylation of Unactivated Alkenes. Org. Lett. 2017, 19, 1744–1747. 10.1021/acs.orglett.7b00499. 28353346

[ref21] Kim D.; Rahaman S. M. W.; Mercado B. Q.; Poli R.; Holland P. L. Roles of Iron Complexes in Catalytic Radical Alkene Cross-Coupling: A Computational and Mechanistic Study. J. Am. Chem. Soc. 2019, 141, 7473–7485. 10.1021/jacs.9b02117. 31025567 PMC6953484

