# Supplementary material for: Metal-Catalyzed Hydrogen Atom Transfer (MHAT) Hydroalkylation with Electron-Deficient Alkynes
Source: Org Lett. 2024 Nov 28;26(49):10553–8. doi: 10.1021/acs.orglett.4c03943 (PMC11650775; doi:10.1021/acs.orglett.4c03943)

Supporting Information

Metal-Catalyzed Hydrogen Atom Transfer (MHAT) Hydroalkylation with Electron-Deficient Alkynes

Laura G. Rodríguez,[†] Josep Bonjoch,[†] and Ben Bradshaw*,[†]

[†]Laboratori de Química Orgànica, Facultat de Farmàcia, IBUB, Universitat de Barcelona, Av. Joan XXIII 27-31, 08028, Barcelona, Spain.
E-mail: benbradshaw@ub.edu

Contents

General information…………………………………………………………………...2

Optimization of the MHAT coupling……………………………………………..…3

Isomerization Studies……………………………………………………..…..……4

Experimental section……………………………………………………………………7

Copies of NMR spectra…………………………………………………………………28

GENERAL INFORMATION

All reactions were carried out under an argon atmosphere with dry, freshly distilled solvents under anhydrous conditions. Analytical thin-layer chromatography was performed on SiO_2 (Merck silica gel 60 F_{254}), and the spots were located with 1% aqueous $KMnO_4$ or 2% ethanolic anisaldehyde. Chromatography refers to flash chromatography and was carried out on SiO_2 (SDS silica gel 60 ACC, 35-75 μm, 230-240 mesh ASTM) or aluminium oxide (neutral) pH 6.5-7.5 (63-200 μm). Drying of organic extracts during workup of reactions was performed over anhydrous Na_2SO_4. Evaporation of solvent was accomplished with a rotary evaporator. NMR spectra were recorded in $CDCl_3$ except were stated otherwise and the chemical shifts of ^{1}H and ^{13}C NMR spectra are reported in ppm downfield (δ) from Me_4Si or $CDCl_3$. All NMR data assignments are supported by gCOSY and gHSQC experiments. High resolution mass spectra (HMRS) were performed using an electrospray (ESI) ionization source and a TOF analyzer (Agilent Technologies).

Compounds **1k**, **2a**, **2b** and **2h** were purchased from Sigma-Aldrich and directly used in the MHAT coupling reactions.

OPTIMIZATION OF THE MHAT COUPLING

Table 1. Optimization of the reaction conditions for the MHAT coupling between alkenes and electron deficient alkynes

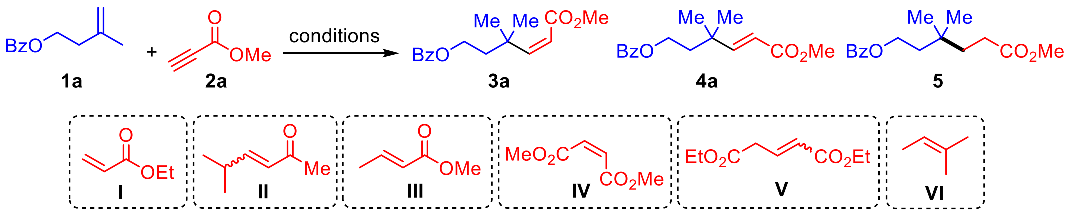

Entry	1a	2a	3a	4a	$PhSiH_3$ (equiv)	Fe^{III} (equiv)	Solvent [0.10 M]	Time (h)	Temperature (°C)	Ratio of 3a/4a/5 (yield %)
**1**	1	1	-	-	2.5	1	EtOH	16	60	50/32/18 (71)
**2**	1	1	-	-	1.5	1	EtOH	16	60	53/30/17 (74)
**3**	1	1	-	-	1.5	0.5	EtOH	16	60	56/33/11 (70)
**4**	1	1	-	-	1.5	1	THF/MeOH[a]	16	60	50/35/15 (64)
**5**	1	1	-	-	1.5	1	DCE	16	60	53/38/9 (52)
**6**	1	1	-	-	1.5	1	EtOH[b]	16	60	51/33/16 (74)
**7**	1	1	-	-	1.5	1	EtOH[c]	16	60	43/32/25 (56)
**8**	1	1	-	-	1.5	1	EtOH	16	25	41/39/20 (55)
**9**	2	1	-	-	1.5	1	EtOH	16	60	63/33/4 (92)
**10**	1	2	-	-	1.5	1	EtOH	16	60	50/31/19 (69)
**11**	2	1	-	-	1.5	0.5	EtOH	16	60	60/36/4 (69)
**12**	3	1	-	-	1.5	1	EtOH	16	60	62/35/3 (97)
**13**	3	1	-	-	1.5	1	EtOH	3	60	62/34/4 (84)
**14**	3	1	-	-	1	0.5	EtOH	16	60	61/37/2 (68)
**15**	3	1	-	-	1.5	0.5[d]	EtOH	16	60	63/31/6 (87)
**16**[e]	3	1	-	-	1.5	1	EtOH	16	60	52/36/12 (68)
**17**[e]	3	1	-	-	1.5	0.2	EtOH	16	60	56/35/9 (51)
**18**	-	-	-	1	1.5	1	EtOH	16	60	0/90/10
**19**	-	-	1	-	1.5	1	EtOH	16	60	43/0/57
**20**	1	1[f]	-	-	1.5	1	EtOH	16	60	50 (3a/4a/5) : 50
**21**	1	1[g]	-	-	2.5	1	EtOH	16	60	56/31/12 (66)
**22**	1	1[h]	-	-	2.5	1	EtOH	16	60	54/31/15 (58)
**23**	1	1[i]	-	-	2.5	1	EtOH	16	60	51/31/18 (63)
**24**	1	1[j]	-	-	2.5	1	EtOH	16	60	53/31/16 (64)
**25**	1	1[k]	-	-	2.5	1	EtOH	16	60	58/35/7 (72)
**26**	1	1[l]	-	-	2.5	1	EtOH	16	60	61/34/4 (56)
**27**	1	1[k]	-	-	1.5	1	EtOH	16	60	60/32/8 (69)
**28**	1	1[l]	-	-	1.5	1	EtOH	16	60	62/33/5 (53)

[a]10 equivalents of MeOH as additive. [b]EtOH [0.05 M]. [c]EtOH [0.20 M]. [d]0.5 equivalents of $Fe(acac)_2$ were added as well. [e]The reaction was run open to air. [f]1.0 equivalent of **I** was added. [g]1.0 equivalent of **II** was added. [h]1.0 equivalent of **III** was added. [i]1.0 equivalent of **IV** was added. [j]1.0 equivalent of **V** was added. [k]1.0 equivalent of **VI** was added. [l]3.0 equivalents of **VI** were added.

During the optimization studies, we evaluated each reaction parameter individually to assess its impact on efficiency. Subsequently, we examined the interactions between the different

parameters to identify the optimal conditions for the transformation. Once these conditions were established, additional experiments were conducted using a variety of additives.

Firstly, the reactivity of our electron deficient alkynes was compared to the Michael acceptors used by Baran's group[1] (Table 1, Entry 20). The results indicated that the reactivity of both acceptors was the same as 50% of each acceptor was consumed during the MHAT coupling. We then considered the use of more sterically hindered Michael acceptors, which, though less reactive toward coupling, might capture hydrogen radicals and reduce overreduction of the coupled products. However, testing additives **II-V** resulted in the formation of only products **3a**, **4a** and **5**, with no significant reduction in the amount of over-reduced product. In the case of additive **VI**, the reduction was slightly diminished, but without improving yields. Additionally, increasing the equivalents of the alkene further decreased the yields of the coupled product.

ISOMERIZATION STUDIES

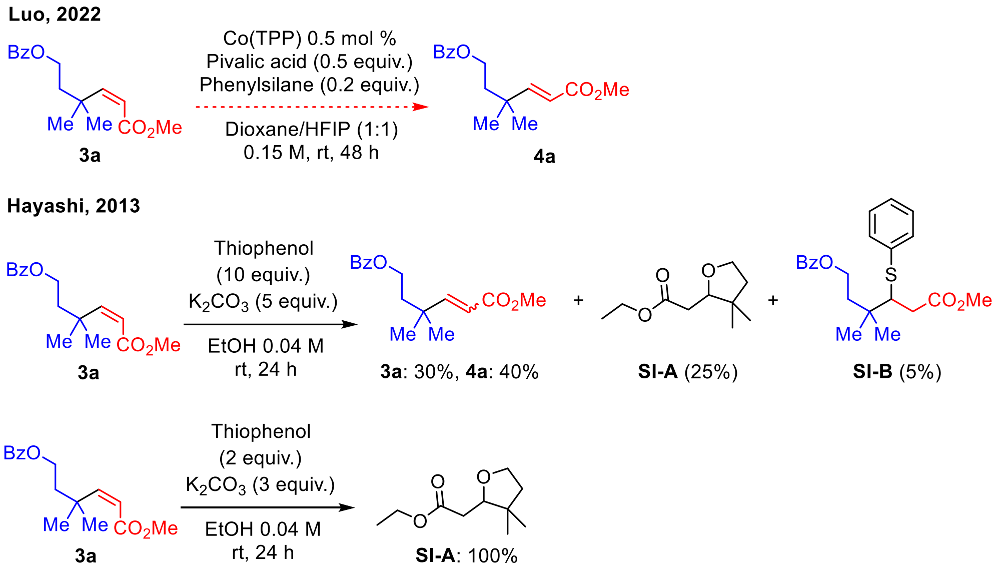

During our first attempts at the isomerization of the *cis* compounds, we used Co(TPP),[2] obtaining a full recovery of starting material. Then, we started exploring the coupling with a thiol to obtain a sulfide that could eventually be oxidized and subsequently eliminated to afford the *trans* product **4a**.[3] When **3a** underwent the reaction with 10 equivalents of thiophenol and 5 equivalents of potassium carbonate, a mixture of **3a**, **4a** and **SI-B** was obtained. However, the product of hydrolysis **SI-A** was also detected, which was detrimental for the efficiency of the coupling. When the amount of base was increased, the complete hydrolysis of the starting material was observed, and therefore we decided to move on to other alternatives involving the formation of a sulfide and subsequent oxidation and elimination.

---

[1] Lo, J. C.; Yabe, Y.; Baran, P. S. A Practical and Catalytic Reductive Olefin Coupling. J. Am. Chem. Soc. **2014**, *136*, 1304–1307.

[2] Fang, X.; Zhang, N.; Chen, S. C.; Luo, T. Scalable Total Synthesis of (-)-Triptonide: Serendipitous Discovery of a Visible-Light-Promoted Olefin Coupling Initiated by Metal-Catalyzed Hydrogen Atom Transfer (MHAT). *J. Am. Chem. Soc*. **2022**, *144*, 2292–2300.

[3] Mukaiyama, T.; Ishikawa, H.; Koshino, H.; Hayashi, Y. One-Pot Synthesis of (-)-Oseltamivir and Mechanistic Insights Into the Organocatalyzed Michael Reaction. *Chem. Eur. J*. **2013**, *19*, 17789–17800.

Table 2. Optimization of the reaction conditions for the isomerization of the *cis*/*trans* mixtures by the formation of a sulfide

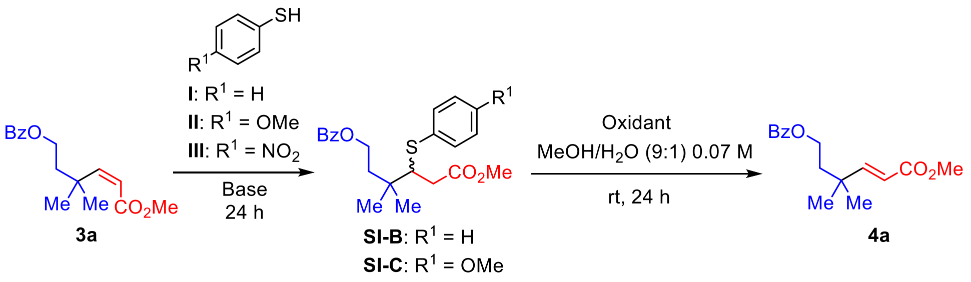

Entry	Reagent	Base	Oxidant	Temperature coupling (ºC)	Solvent	Conversion 3a/SI-B or C/4a
**1**	**I** (2.0 equiv.)	TEA (0.25 equiv.)	-	rt	EtOH 0.1 M	80/10/10
**2**	**I** (2.0 equiv.)	Amberlyst® A21 catalyst 0.1 g/mmol	-	rt	EtOH 0.1 M	100/0/0
**3**	**I** (2.0 equiv.)	PS-BEMP	-	rt	EtOH 0.1 M	95/2/3
**4**	**I** (2.0 equiv.)	LiOH (0.1 equiv.)	-	rt	EtOH 0.1 M	94/4/2
**5**	**I** (2.0 equiv.)	K_2CO_3 (0.1 equiv.)	-	rt	EtOH 0.1 M	93/3/4
**6**	**I** (2.0 equiv.)	TEA (0.25 equiv.)	-	rt	Benzene 0.1 M	100/0/0
**7**	**I** (2.0 equiv.)	TEA (0.25 equiv.)	-	rt	CH_2Cl_2 0.1 M	100/0/0
**8**	**I** (2.0 equiv.)	TEA (0.25 equiv.)	-	rt	THF 0.1 M	100/0/0
**9**	**I** (2.0 equiv.)	TEA (0.25 equiv.)	-	rt	No solvent	15/51/34
**10**	**I** (1.0 equiv.)	TEA (0.25 equiv.)	-	rt	No solvent	25/35/41
**11**	**I** (1.0 equiv.)	TEA (1.0 equiv.)	-	40 ºC	No solvent	9/52/39
**12**	**I** (2.0 equiv.)	TEA (1.0 equiv.)	-	40 ºC	No solvent	0/95/5
**13**[a]	**I** (2.0 equiv.)	TEA (1.0 equiv.)	-	40 ºC	No solvent	0/95/5
**14**	**II** (2.0 equiv.)	TEA (1.0 equiv.)	-	40 ºC	No solvent	0/88/12
**15**	**III** (2.0 equiv.)	TEA (1.0 equiv.)	-	40 ºC	No solvent	100/0/0
**16**[b]	**I** (2.0 equiv.)	TEA (1.0 equiv.)	$NaIO_4$ (3.0 equiv.)	40 ºC	No solvent	0/53/47
**17**[c]	**I** (2.0 equiv.)	TEA (1.0 equiv.)	$NaIO_4$ (3.0 equiv.)	40 ºC	No solvent	0/0/100
**18**[c]	**II** (2.0 equiv.)	TEA (1.0 equiv.)	$NaIO_4$ (3.0 equiv.)	40 ºC	No solvent	0/0/100

[a]Isomer **4a** was used for the coupling with thiophenol. [b]No evaporation of the base between the coupling and the oxidation steps. [c]The residue of the coupling was stirred at 70 ºC for thirty minutes under vacuum prior to oxidation.

The isomerization reaction was optimized by coupling *cis* compound **3a** with thiophenol using various bases. Only triethylamine provided significant amounts of the desired sulfide (Table 2, Entries 1-5), with some *trans* product **4a** also forming due to partial elimination of sulfide **SI-B**.

After identifying triethylamine as the optimal base, different solvents were tested (Entries 6-9). Solvent-free conditions gave 85% conversion of the *cis* isomer. Further optimization showed that reducing the thiol amount decreased efficiency (Entry 10), but raising the temperature to 40 ºC and using 1.0 equivalent of triethylamine improved results, despite a drop in spontaneous sulfide elimination (Entry 11). The optimal conditions (Entry 12) resulted in full conversion of **3a**, and applying them to the *trans* isomer **4a** also yielded sulfide **SI-B** (Entry 13). Testing two additional thiols (**II** and **III**) revealed 4-methoxythiophenol was also effective (Entries 14-15).

In the oxidation step, directly treating the coupling residue didn't fully accomplish the oxidation and elimination of **SI-B** (Entry 16). However, stirring at 70 ºC under vacuum before oxidation (Entry 17) quantitatively yielded *trans* product **4a** without sulfone thermolysis. The sulfone

spontaneously eliminated during oxidation, producing clean crude products. With 4-methoxythiophenol (**II**) complete oxidation was achieved (Entry 18), but additional sulfone thermolysis in toluene was required.

EXPERIMENTAL SECTION

SYNTHESIS OF THE ALKENE DONORS

General method for the benzoyl protection of the MHAT donors:[4] To a solution of the selected alkene (1.0 equiv.) and triethylamine (1.5 equiv.) in dichloromethane (0.67 M) was added 4-dimethylaminopyridine (0.1 equiv.) under an argon atmosphere. Then, the solution was cooled to 0 °C with an ice bath and benzoyl chloride (1.05 equiv.) was added dropwise. After stirring the solution for 3 hours at room temperature, the mixture was diluted with dichloromethane and subsequently washed with water and 10% citric acid. The organic layer was dried with sodium sulphate and concentrated. The corresponding product was purified by flash column chromatography on silica gel.

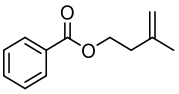

3-methylbut-3-en-1-yl benzoate (**1a**). The title compound was prepared according to general procedure using 3-methylbut-3-en-1-ol (1.72 g, 2.02 mL, 20.0 mmol, 1 equiv.), benzoyl chloride (2.95 g, 2.44 mL, 21.0 mmol, 1.05 equiv.), triethylamine (3.04 g, 4.16 mL, 30.0 mmol, 1.5 equiv.) and 4-dimethylaminopyridine (244 mg, 2.00 mmol, 0.1 equiv.) in dichloromethane (30 mL, 0.67 M). Purification by chromatography (Hex → Hex/AcOEt 10%) gave **1a** (3.80 g, 100%) as a colourless oil. Spectral data were identical to those previously reported;[4] ^{1}H NMR (400 MHz, $CDCl_3$) δ 8.07 – 8.01 (m, 2H), 7.58 – 7.52 (m, 1H), 7.47 – 7.40 (m, 2H), 4.87 – 4.80 (m, 2H), 4.44 (t, J = 6.8 Hz, 2H), 2.49 (t, J = 6.7 Hz, 2H), 1.82 (s, 3H); ^{13}C{^{1}H} NMR (101 MHz, $CDCl_3$) δ 166.7, 141.8, 133.0, 130.5, 129.7, 128.5, 112.5, 63.3, 36.9, 22.6 ppm.

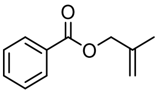

2-methylallyl benzoate (**1b**). The title compound was prepared according to general procedure using methallyl alcohol (721 mg, 0.84 mL, 10.0 mmol, 1 equiv.), benzoyl chloride (1.48 g, 1.22 mL, 10.5 mmol, 1.05 equiv.), triethylamine (1.52 g, 2.08 mL, 15.0 mmol, 1.5 equiv.) and 4-dimethylaminopyridine (122 mg, 1.00 mmol, 0.1 equiv.) in dichloromethane (15 mL, 0.67 M). Purification by chromatography (Hex → Hex/AcOEt 10%) gave **1b** (1.59 g, 90%) as a colourless oil. Spectral data were identical to those previously reported;[5] ^{1}H NMR (400 MHz, $CDCl_3$) δ 8.11 – 8.04 (m, 2H), 7.61 – 7.52 (m, 1H), 7.50 – 7.40 (m, 2H), 5.11 – 5.06 (m, 1H), 5.02 – 4.96 (m, 1H), 4.75 (s, 2H), 1.85 (s, 3H); ^{13}C{^{1}H} NMR (101 MHz, $CDCl_3$) δ 166.4, 140.1, 133.1, 130.3, 129.7, 128.5, 113.1, 68.2, 19.7 ppm.

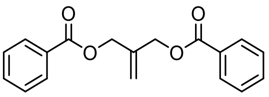

2-methylenepropane-1,3-diyl dibenzoate (**1f**). The title compound was prepared according to a modification of the general procedure using 2-methylenepropane-1,3-diol (705 mg, 0.65 mL, 8.0 mmol, 1.0 equiv.), benzoyl chloride (2.25 g, 1.86 mL, 16.0 mmol, 2.0 equiv.), triethylamine (2.43 g, 3.33 mL, 24.0 mmol, 3.0 equiv.) and 4-dimethylaminopyridine

[4] Xie, Y.; Sun, P. W.; Li, Y.; Wang, S.; Ye, M.; Li, Z. Ligand-Promoted Iron(III)-Catalyzed Hydrofluorination of Alkenes. *Angew. Chem. Int. Ed.* **2019**, *58*, 7097–7101.

[5] Bertrand, X.; Paquin, J. F. Direct Hydrofluorination of Methallyl Alkenes Using a Methanesulfonic Acid/Triethylamine Trihydrofluoride Combination. *Org. Lett.* **2019**, *21*, 9759–9762.

(195 mg, 1.60 mmol, 0.2 equiv.) in dichloromethane (12 mL, 0.67 M). Purification by chromatography (Hex → Hex/AcOEt 25%) gave **1f** (2.20 g, 93%) as a colourless solid. Spectral data were identical to those previously reported;[6] ^{1}H NMR (400 MHz, $CDCl_3$) δ 8.10 – 8.01 (m, 4H), 7.56 (ddt, *J* = 8.0, 6.9, 1.4 Hz, 2H), 7.48 – 7.38 (m, 4H), 5.45 (s, 2H), 4.96 (s, 4H); ^{13}C{^{1}H} NMR (101 MHz, $CDCl_3$) δ 166.3, 138.8, 133.3, 130.0, 129.8, 128.5, 117.4, 65.3 ppm.

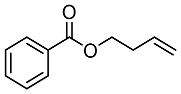

but-3-en-1-yl benzoate (**1g**). The title compound was prepared according to general procedure using 3-buten-1-ol (1.44 g, 1.72 mL, 20.0 mmol, 1.0 equiv.), benzoyl chloride (2.95 g, 2.44 mL, 21.0 mmol, 1.05 equiv.), triethylamine (3.04 g, 4.16 mL, 30.0 mmol, 1.5 equiv.) and 4-dimethylaminopyridine (244 mg, 2.00 mmol, 0.1 equiv.) in dichloromethane (30 mL, 0.67 M). Purification by chromatography (Hex → Hex/AcOEt 10%) gave **1g** (3.35 g, 95%) as a colourless oil. Spectral data were identical to those previously reported;[7] ^{1}H NMR (400 MHz, $CDCl_3$) δ 8.08 – 8.01 (m, 2H), 7.60 – 7.51 (m, 1H), 7.48 – 7.39 (m, 2H), 5.88 (ddt, *J* = 17.0, 10.2, 6.7 Hz, 1H), 5.22 – 5.14 (m, 1H), 5.15 – 5.07 (m, 1H), 4.38 (t, *J* = 6.7 Hz, 2H), 2.57 – 2.49 (m, 2H); ^{13}C{^{1}H} NMR (101 MHz, $CDCl_3$) δ 166.7, 134.2, 133.0, 130.5, 129.7, 128.5, 117.5, 64.1, 33.3 ppm.

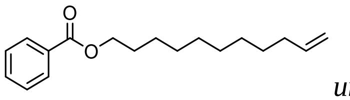

undec-10-en-1-yl benzoate (**1h**). The title compound was prepared according to general procedure using undec-10-en-1-ol (1.36 g, 1.60 mL, 8.0 mmol, 1.0 equiv.), benzoyl chloride (1.18 g, 0.98 mL, 8.4 mmol, 1.05 equiv.), triethylamine (1.21 g, 1.66 mL, 12.0 mmol, 1.5 equiv.) and 4-dimethylaminopyridine (98 mg, 0.8 mmol, 0.1 equiv.) in dichloromethane (12 mL, 0.67 M). Purification by chromatography (Hex → Hex/AcOEt 10%) gave **1h** (2.15 g, 98%) as a colourless oil. Spectral data were identical to those previously reported;[8] ^{1}H NMR (400 MHz, $CDCl_3$) δ 8.08 – 8.01 (m, 2H), 7.60 – 7.51 (m, 1H), 7.48 – 7.39 (m, 2H), 5.81 (ddt, *J* = 16.9, 10.2, 6.7 Hz, 1H), 4.99 (ddt, *J* = 17.1, 2.2, 1.5 Hz, 1H), 4.93 (ddt, *J* = 10.2, 2.3, 1.2 Hz, 1H), 4.32 (t, *J* = 6.7 Hz, 2H), 2.09 – 1.98 (m, 2H), 1.82 – 1.71 (m, 2H), 1.49 – 1.24 (m, 12H); ^{13}C{^{1}H} NMR (101 MHz, $CDCl_3$) δ 166.8, 139.3, 132.9, 130.7, 129.7, 128.4, 114.3, 65.3, 33.9, 29.6, 29.5, 29.4, 29.2, 29.1, 28.9, 26.2 ppm.

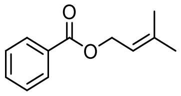

3-methylbut-2-en-1-yl benzoate (**1i**) The title compound was prepared according to general procedure using 3-methyl-2-buten-1-ol (689 mg, 0.81 mL, 8.0 mmol, 1.0 equiv.), benzoyl chloride (1.18 g, 0.98 mL, 8.4 mmol, 1.05 equiv.), triethylamine (1.21 g, 1.66 mL, 12.0 mmol, 1.5 equiv.) and 4-dimethylaminopyridine (98 mg, 0.8 mmol, 0.1 equiv.) in dichloromethane (12 mL, 0.67 M). Purification by chromatography (Hex → Hex/AcOEt 10%) gave **1i** (1.49 g, 98%) as a colourless oil. Spectral data were identical to those previously reported;[9] ^{1}H NMR (400 MHz, $CDCl_3$) δ 8.08 – 8.01 (m, 2H), 7.58 – 7.50 (m, 1H), 7.47 – 7.37

---

[6] Mitchell, J. M.; Finney, N. S. Synthetic studies of pseurotin A: preparation of an advanced lactam aldehyde intermediate. *Org. Biomol. Chem.* **2005**, *3*, 4274–4281.

[7] Hu, K. F.; Ning, X. S.; Qu, J. P.; Kang, Y. B. Tuning Regioselectivity of Wacker Oxidation in One Catalytic System: Small Change Makes Big Step. *J. Org. Chem.* **2018**, *83*, 11327–11332.

[8] Tappin, N. D. C; Michalska, W.; Rohrbach, S.; Renaud, P. Cyclopropanation of Terminal Alkenes though Sequential Atom-Transfer Radical Addition/1,3-Elimination. *Angew. Chem. Int. Ed.* **2019**, *58*, 14240–14244.

[9] Wuest, F. R.; Berndt, M. ^{11}C-C bond formation by palladium-mediated cross-coupling of alkenylzirconocenes with [^{11}C]methyl iodide. *J. Label. Compd. Radiopharm.* **2006**, *49*, 91–100.

(m, 2H), 5.53 – 5.41 (m, 1H), 4.82 (d, *J* = 7.2 Hz, 2H), 1.78 (d, *J* = 6.9 Hz, 6H); $^{13}C\{^{1}H\}$ NMR (101 MHz, $CDCl_3$) δ 166.8, 139.3, 132.9, 130.7, 129.7, 128.4, 118.8, 62.0, 25.9, 18.2 ppm.

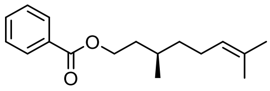
(R)-3,7-dimethyloct-6-en-1-yl benzoate (**1j**). The title compound was prepared according to general procedure using citronellol (1.25 g, 1.47 mL, 8.0 mmol, 1.0 equiv.), benzoyl chloride (1.18 g, 0.98 mL, 8.4 mmol, 1.05 equiv.), triethylamine (1.21 g, 1.66 mL, 12.0 mmol, 1.5 equiv.) and 4-dimethylaminopyridine (98 mg, 0.80 mmol, 0.1 equiv.) in dichloromethane (12 mL, 0.67 M). Purification by chromatography (Hex → Hex/AcOEt 10%) gave **1j** (2.00 g, 96%) as a colourless oil. Spectral data were identical to those previously reported;[10] 1H NMR (400 MHz, $CDCl_3$) δ 8.08 – 8.00 (m, 2H), 7.60 – 7.51 (m, 1H), 7.48 – 7.39 (m, 2H), 5.15 – 5.05 (m, 1H), 4.44 – 4.29 (m, 2H), 2.12 – 1.91 (m, 2H), 1.88 – 1.76 (m, 1H), 1.72 – 1.63 (m, 4H), 1.63 – 1.52 (m, 4H), 1.47 – 1.35 (m, 1H), 1.31 – 1.19 (m, 1H), 0.98 (d, *J* = 6.5 Hz, 3H); $^{13}C\{^{1}H\}$ NMR (101 MHz, $CDCl_3$) δ 166.8, 132.9, 131.5, 130.7, 129.7, 128.5, 124.7, 63.6, 37.1, 35.6, 29.7, 25.8, 25.5, 19.7, 17.8 ppm.

Synthesis of 2-(3-methylbut-3-en-1-yl)isoindoline-1,3-dione (1c).[11]

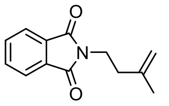
To a solution of 3-methyl-3-buten-1-ol (861 mg, 1.01 mL, 10.0 mmol, 1.0 equiv.), phthalimide (1.62 g, 11.0 mmol, 1.1 equiv.) and PPh_3 (2.89 g, 11.0 mmol, 1.1 equiv.) in anhydrous THF (30 mL, 0.33 M) was added diethyl azodicarboxylate (1.92 g, 1.73 mL, 11.0 mmol, 1.1 equiv.) dropwise at 0 °C and the resultant solution was left to stir at room temperature for 5 hours. Afterwards, the reaction mixture was concentrated under vacuum. Purification by chromatography (Hex → Hex/AcOEt 25%) gave **1c** (1.83 g, 85%) as a colourless oil. Spectral data were identical to those previously reported;[12] ^{1}H NMR (400 MHz, $CDCl_3$) δ 7.85 – 7.79 (m, 2H), 7.72 – 7.67 (m, 2H), 4.76 – 4.69 (m, 1H), 4.69 – 4.64 (m, 1H), 3.82 (t, *J* = 7.2 Hz, 2H), 2.39 (t, *J* = 7.2 Hz, 2H), 1.80 (s, 3H); $^{13}C\{^{1}H\}$ NMR (101 MHz, $CDCl_3$) δ 168.4, 142.3, 134.0, 132.2, 123.3, 112.9, 36.6, 36.5, 22.2 ppm.

Synthesis of di-*tert*-butyl (3-methylbut-3-en-1-yl)carbamate (1d).[13]

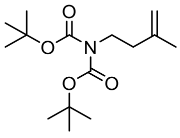
To a solution of 3-methyl-3-buten-1-ol (431 mg, 0.51 mL, 5.0 mmol, 1.0 equiv.), di-*tert*-butyl-iminodicarboxylate (1.19 g, 5.5 mmol, 1.1 equiv.) and PPh_3 (1.44 g, 5.5 mmol, 1.1

[10] García, C.; León, L. G.; Pungitore, C. R.; Ríos-Luci, C.; Daranas, A. H. Montero, J. C.; Pandiella, A.; Tonn, C. E.; Martín, V. S.; Padrón, J. M. Enhancement of antiproliferative activity by molecular simplification of catalpol. *Bioorg. Med. Chem.* **2010**, *18*, 2515–2523.
[11] Ren, J.; Du, F. H.; Jia, M. C.; Hu, Z. N.; Chen, Z.; Zhang, C. Ring Expansion Fluorination of Unactivated Cyclopropanes Mediated by New Monofluoroiodane(III) Reagent. *Angew. Chem. Int. Ed.* **2021**, *60*, 24171–24178.
[12] Siu, J. C.; Parry, J. B.; Lin, S. Aminoxyl-Catalyzed Electrochemical Diazidation of Alkenes Mediated by a Metastable Charge-Transfer Complex. *J. Am. Chem. Soc.* **2019**, *141*, 2825–2831.
[13] Zang, Z. L.; Zhao, S.; Karnakanti, S.; Liu, C. L.; Shao, P. L.; He, Y. Catalytic Multisite-Selective Acetoxylation Reactions at sp^2 vs sp^3C-H Bonds in Cyclic Olefins. *Org. Lett.* **2016**, *18*, 5014–5017.

equiv.) in anhydrous THF (15 mL, 0.33 M) was added diisopropyl azodicarboxylate (1.11 g, 1.1 mL, 5.5 mmol, 1.1 equiv.) dropwise at 0 °C and the resulting solution was left to stir at room temperature overnight. The solvent was evaporated, and the residue was suspended in Et_2O. After filtration, the organic extracts were concentrated. Purification by chromatography (Hex → Hex/AcOEt 25%) gave **1d** (599 mg, 42%) as a colourless oil; ^{1}H NMR (400 MHz, $CDCl_3$) δ 4.77 – 4.72 (m, 1H), 4.72 – 4.67 (m, 1H), 3.69 – 3.61 (m, 2H), 2.26 (t, *J* = 6.4 Hz, 2H), 1.75 (s, 3H), 1.49 (s, 18H); ^{13}C{^{1}H} NMR (101 MHz, $CDCl_3$) δ 152.6, 142.8, 112.3, 82.2, 45.3, 37.3, 28.2, 22.5 ppm. HRMS (ESI) m/z: $[M+H]^+$: calculated for $C_{15}H_{28}NO_4^+$ 286.2013, found 286.2015.

Synthesis of *tert*-butyl (3-methylbut-3-en-1-yl)(tosyl)carbamate (1e).[11]

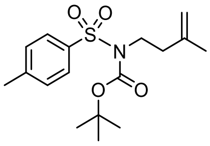

To a solution of 3-methyl-3-buten-1-ol (431 mg, 0.51 mL, 5.0 mmol, 1.0 equiv.), *tert*-butyl tosylcarbamate (1.49 g, 5.5 mmol, 1.1 equiv.) and PPh_3 (1.44 g, 5.5 mmol, 1.1 equiv.) in anhydrous THF (15 mL, 0.33 M) was added diethyl azodicarboxylate (958 mg, 0.86 mL, 5.5 mmol, 1.1 equiv.) dropwise at 0 °C and the resulting solution was left to stir at room temperature for 5 hours. Afterwards, the reaction mixture was concentrated under vacuum. Purification by chromatography (Hex → Hex/AcOEt 25%) gave **1e** (1.19 g, 70%) as a white solid; ^{1}H NMR (400 MHz, $CDCl_3$) δ 7.81 – 7.76 (m, 2H), 7.29 (d, *J* = 7.7 Hz, 2H), 4.84 – 4.73 (m, 2H), 3.96 – 3.88 (m, 2H), 2.48 – 2.44 (m, 2H), 2.43 (s, 3H), 1.79 (s, 3H), 1.34 (s, 9H); ^{13}C{^{1}H} NMR (101 MHz, $CDCl_3$) δ 151.0, 144.2, 142.3, 137.7, 129.3, 128.0, 112.7, 84.2, 46.0, 38.4, 28.0, 22.6, 21.7 ppm. HRMS (ESI) m/z: $[M+H]^+$: calculated for $C_{17}H_{26}NO_4S^+$ 340.1577, found 340.1575.

SYNTHESIS OF THE ELECTRON DEFICIENT ALKYNES

Synthesis of 2-naphthyl propiolate (2c).[14]

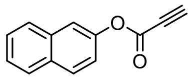

A solution of 2-naphthol (577 mg, 4.0 mmol, 1.0 equiv.) in tetrahydrofuran (40 mL, 0.10 M) at 0 ºC was treated with sodium hydride (dry 90%, 117 mg, 4.4 mmol, 1.1 equiv.). On a separate flask, a solution of propiolic acid (925 mg, 0.81 mL, 13.2 mmol, 3.3 equiv.) in tetrahydrofuran (40 mL, 0.10 M) at 0 ºC was treated sequentially with dicyclohexylcarbodiimide (2.72 g, 13.2 mmol, 3.3 equiv.) and the above naphthyl mixture. The resulting mixture was allowed to stir at room temperature for 16 h. Upon completion of the reaction, the solvent was evaporated and the residue was treated with acetonitrile (40 mL). Then, the mixture was filtered and concentrated under reduced pressure. Purification by chromatography (Hex → Hex/Et_2O 20%) gave **2c** (691 mg, 89%) as a white solid. Spectral data were identical to those previously reported;[14] ^{1}H NMR (400 MHz, $CDCl_3$) 7.91 – 7.79 (m, 3H), 7.64 (d, *J* = 2.4 Hz, 1H), 7.56 – 7.46 (m, 2H), 7.28 (dd, *J* = 8.9, 2.4 Hz, 1H), 3.11 (s, 1H) ppm; ^{13}C{^{1}H} NMR (101 MHz, $CDCl_3$) δ 151.2, 147.5, 133.7, 131.9, 129.9, 128.0, 127.0, 126.3, 120.5, 118.7, 77.1, 74.4 ppm.

Synthesis of *N*-methyl-*N*-phenylpropiolamide (2d).[15]

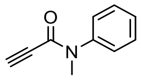

To a solution of *N*-methylaniline (536 mg, 541 μL, 5.0 mmol, 1.0 equiv.) in dichloromethane (20 mL, 0.25 M) was added dicyclohexylcarbodiimide (1.13 g, 5.5 mmol, 1.1 equiv.) and 4-dimethylaminopyridine (61 mg, 0.50 mmol, 0.1 equiv.) and the resulting mixture was stirred for 10 minutes at 0 °C. After, propiolic acid (385 mg, 341 μL, 5.5 mmol, 1.1 equiv.) was added dropwise and the solution was left to stir at room temperature for 3 hours. Upon completion of the reaction, the mixture was filtered through Celite, saturated with a Na_2CO_3 solution (15 mL) and extracted with dichloromethane (3 × 15 mL). The combined organic layers were washed with brine, dried with Na_2SO_4, filtered, and concentrated under vacuum. Purification by chromatography (Hex → Hex/AcOEt 25%) gave **2d** (422 mg, 53%) as a brown solid. Spectral data were identical to those previously reported;[16] ^{1}H NMR (400 MHz, $CDCl_3$) δ 7.47 – 7.32 (m, 3H), 7.32 – 7.25 (m, 2H), 3.60 (s, 0.4H, [m]), 3.33 (s, 2.6H, [M]), 3.28 (s, 0.1H, [m]), 2.81 (s, 0.8H, [M]); ^{13}C{^{1}H} NMR (101 MHz, $CDCl_3$) δ 153.1, 142.7, 129.4 (m), 129.3 (M), 128.3 (m), 127.3 (m), 127.2 (M), 125.5 (M), 80.2 (m), 79.6 (M), 76.4, 39.6 (m), 36.6 (M) ppm.

[14] Cervi, A.; Vo, Y.; Chai, C. L. L.; Banwell, M. G.; Lan, P.; Willis, A. C. Gold(I)-Catalyzed Intramolecular Hydroarylation of Phenol-Derived Propiolates and Certain Related Ethers as a Route to Selectively Functionalized Coumarins and 2*H*-Chromenes. *J. Org. Chem.* **2021**, *86*, 178–198.
[15] Chen, W. S.; Yang, F.; Wang, T.; Zhang, G. Q.; Wei, Y.; Wang, M. H.; Chen, Z. S.; Ji, K. Chemoselective Transformations of Amides: An Approach to Quinolones from β-Amido Ynones. *Org. Lett.* **2023**, *25*, 5762–5767.
[16] Reichart, B.; Guedes de la Cruz, G.; Zangger, K.; Kappe, C. O.; Glasnov, T. Copper/Nafion-Catalyzed Hydroarylation Process Involving Ketenimine Intermediates: A Novel and Synthetic Approach to 4-Sulfonamidoquinoline-2-ones and Derivatives Thereof. *Adv. Synth. Catal.* **2016**, *358*, 50–55.

Synthesis of (ethynylsulfonyl)benzene (**2e**).[17]

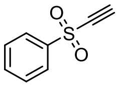

To a solution of trimethylsilylacetylene (648 mg, 939 μL, 6.60 mmol, 1.1 equiv.) in anhydrous tetrahydrofuran (13.2 mL, 0.5 M) was added *n*-BuLi (1.6 M in hexane, 3.6 mL, 5.70 mmol, 0.95 equiv.) dropwise at -78 °C under argon. The solution was left to stir for 30 minutes and a solution of phenyl disulfide (1.31 g, 6.0 mmol, 1.0 equiv.) in tetrahydrofuran (2 mL) was added dropwise. After stirring the solution for 1 hour at -78 °C, the reaction was stirred for an additional 30 minutes at room temperature and subsequently quenched with water. The mixture was extracted with diethyl ether (3 × 30 mL) and the combined organic layers were washed with brine, dried with Na_2SO_4, filtered, and concentrated. The residue was dissolved in dichloromethane (25 mL, 0.24 M) and *m*CPBA (2.59 g, 15.0 mmol, 2.5 equiv.) was added portionwise. The resulting solution was left to stir at room temperature for 2 hours and subsequently treated with a saturated solution of Na_2SO_3 and extracted with dichloromethane (3 × 20 mL). The organic layer was treated with silica for 2 hours at room temperature, filtered and concentrated. Purification by chromatography (Hex → Hex/AcOEt 25%) gave **2e** (528 mg, 53%) as a colourless liquid. Spectral data were identical to those previously reported;[15] ^{1}H NMR (400 MHz, $CDCl_3$) δ 8.07 – 7.97 (m, 2H), 7.77 – 7.67 (m, 1H), 7.66 – 7.56 (m, 2H), 3.48 (s, 1H).ppm.

Synthesis of 5-phenylpent-1-yn-3-one (**2f**).[19]

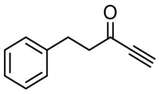

To a solution of trimethylsilylacetylene (491 mg, 692 μL, 5.00 mmol, 1.0 equiv.) in anhydrous tetrahydrofuran (10 mL, 0.5 M) was added *n*-BuLi (1.6 M in hexane, 3.0 mL, 4.75 mmol, 0.95 equiv.) dropwise at -78 °C under argon. The solution was left to stir for 30 minutes and 3-phenylpropionaldehyde (671 mg, 664 μL, 5.00 mmol, 1.0 equiv.) was added dropwise. After stirring the solution for 1 hour at -78 °C, the reaction was stirred for an additional 30 minutes at room temperature and subsequently quenched with water. The mixture was extracted with ethyl acetate (3 × 30 mL) and the combined organic layers were washed with brine, dried with Na_2SO_4, filtered, and concentrated. The residue was dissolved in methanol (20 mL, 0.25 M) and potassium carbonate (2.07 g, 15.0 mmol, 3.0 equiv.) was added in one portion. The mixture was allowed to stir at room temperature for 2 hours and then filtered through a silica plug. Afterwards, the crude product was dissolved in anhydrous dichloromethane (20 mL, 0.25 M) and Dess-Martin reagent (2.33 g, 5.5 mmol, 1.1 equiv.) was added. The solution was left to stir for 2 hours and subsequently concentrated. Purification by chromatography (Hex → Hex/AcOEt 25%) gave **2f** (617 mg, 78%) as a colourless liquid. Spectral data were identical to those previously reported;[18] ^{1}H NMR (400 MHz, $CDCl_3$) δ 7.34 – 7.23 (m, 2H), 7.25 – 7.16 (m, 3H), 3.23 (s, 1H), 3.04 – 2.96 (m, 2H), 2.97 – 2.88 (m, 2H); $^{13}C\{^1H\}$ NMR (101 MHz, $CDCl_3$) δ 186.4, 140.1, 128.7, 128.5, 126.5, 81.5, 79.0, 47.1, 29.7 ppm.

[17] García Ruano, J. L.; Alemán, J.; García Paredes, C. Oxidative Addition of Pd(0) to Ar-SO_2R Bonds: Heck-Type Reactions of Sulfones. *Org. Lett.* **2006**, *8*, 2683–2686.
[18] Bella, M.; Jorgensen, K. A. Organocatalytic Enantioselective Conjugate Addition to Alkynones. *J. Am. Chem. Soc.* **2004**, *126*, 5672–5673.

Synthesis of 1-(naphthalen-2-yl)prop-2-yn-1-one (2g).[19]

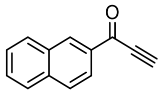

To a solution of trimethylsilylacetylene (688 mg, 1.0 mL, 7.00 mmol, 1.1 equiv.) in anhydrous tetrahydrofuran (13 mL, 0.5 M) was added *n*-BuLi (1.6 M in hexane, 4.0 mL, 6.40 mmol, 1.0 equiv.) dropwise at -78 °C under argon. The solution was left to stir for 30 minutes and a solution of 2-naphthaldehyde (1.00 g, 6.40 mmol, 1.0 equiv.) in tetrahydrofuran (2 mL) was added dropwise. After stirring the solution for 1 hour at -78 °C, the reaction was stirred for an additional 30 minutes at room temperature and subsequently quenched with water. The mixture was extracted with ethyl acetate (3 × 30 mL) and the combined organic layers were washed with brine, dried with Na_2SO_4, filtered, and concentrated. The residue was dissolved in methanol (13 mL, 0.5 M) and potassium carbonate (2.65 g, 19.2 mmol, 3.0 equiv.) was added in one portion. The mixture was allowed to stir at room temperature for 2 hours and then filtered through a silica plug. Afterwards, the crude product was dissolved in anhydrous dichloromethane (13 mL, 0.5 M) and Dess-Martin reagent (2.71 g, 6.40 mmol, 1.0 equiv.) was added. The solution was left to stir for 2 hours and subsequently concentrated. Purification by chromatography (Hex → Hex/AcOEt 25%) gave **2g** (1.08 g, 94%) as a white solid. Spectral data were identical to those previously reported;[20] ^{1}H NMR (400 MHz, $CDCl_3$) δ 8.77 – 8.71 (m, 1H), 8.13 (dd, *J* = 8.7, 1.8 Hz, 1H), 8.01 (dd, *J* = 8.1, 1.3 Hz, 1H), 7.93 – 7.85 (m, 2H), 7.64 (ddd, *J* = 8.2, 6.9, 1.4 Hz, 1H), 7.58 (ddd, *J* = 8.2, 6.9, 1.3 Hz, 1H), 3.51 (s, 1H); ^{13}C{^{1}H} NMR (101 MHz, $CDCl_3$) δ 177.4, 136.4, 133.8, 133.5, 132.5, 130.0, 129.4, 128.8, 128.1, 127.2, 123.7, 80.8, 80.5 ppm.

Synthesis of ethyl 3-bromopropiolate (2i).[21]

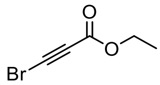

To a solution of ethyl propiolate (491 mg, 506 μL, 5.00 mmol, 1.0 equiv.) in acetone (10 mL, 0.5 M) was added silver nitrate (85 mg, 0.50 mmol, 0.1 equiv.) and the solution was left to stir at room temperature for 5 minutes. Afterwards, *N*-bromosuccinimide (979 mg, 5.50 mmol, 1.1 equiv.) was added and the solution was stirred at room temperature for 3 hours. The resulting mixture was filtered and concentrated under vacuum. Purification by chromatography (Hex → Hex/AcOEt 10%) gave **2i** (858 mg, 97%) as a colourless oil. Spectral data were identical to those previously reported;[21] ^{1}H NMR (400 MHz, $CDCl_3$) δ 4.23 (q, *J* = 7.1 Hz, 2H), 1.30 (t, *J* = 7.1 Hz, 3H); ^{13}C{^{1}H} NMR (101 MHz, $CDCl_3$) δ 152.6, 73.0, 62.6, 52.5, 14.1 ppm.

[19] Jie, Y.; Hu, H.; Xu, Z. F.; Duan, S.; Li, C. Y. Synthesis of 3-Methylidene-2,3-dihydropyrroles via Formal 1,2-Enamine Migration/Cyclization Cascade of Rhodium Carbenes. *Adv. Synth. Catal.* **2023**, *365*, 161–166.
[20] Nanko, M.; Shibuya, S.; Inaba, Y.; Ono, S.; Ito, S.; Mikami, K. gem-Digold Acetylide Complexes for Catalytic Intermolecular [4+2] Cycloaddition: Having Two Gold Centers Is Better for Asymmetric Catalysis. *Org. Lett.* **2018**, *20*, 7353–7357.
[21] Poulsen, T. B.; Bernardi, L.; Alemán, J.; Overgaard, J.; Jørgensen, K. A. Organocatalytic Asymmetric Direct α-Alkynylation of Cyclic β-Ketoesters. *J. Am. Chem. Soc.* **2007**, *129*, 441–449.

Synthesis of naphthalen-2-yl 3-bromopropiolate (2j).[21]

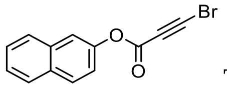

To a solution of naphthalen-2-yl propiolate (260 mg, 1.33 mmol, 1.0 equiv.) in acetone (2.7 mL, 0.5 M) was added silver nitrate (23 mg, 0.13 mmol, 0.1 equiv.) and the solution was left to stir at room temperature for 5 minutes. Afterwards, *N*-bromosuccinimide (260 mg, 1.46 mmol, 1.1 equiv.) was added and the solution was stirred at room temperature for 3 hours. The resulting mixture was filtered and concentrated under vacuum. Purification by chromatography (Hex → Hex/AcOEt 10%) gave **2j** (310 mg, 85%) as a white solid; ^{1}H NMR (400 MHz, $CDCl_3$) δ 7.90 – 7.76 (m, 3H), 7.61 (d, *J* = 2.2 Hz, 1H), 7.55 – 7.43 (m, 2H), 7.25 (dd, *J* = 8.9, 2.4 Hz, 1H).; $^{13}C\{^{1}H\}$ NMR (101 MHz, $CDCl_3$) δ 151.0, 147.6, 133.7, 131.8, 129.8, 127.93, 127.90, 127.0, 126.3, 120.5, 118.6, 72.6, 55.6 ppm. HRMS (ESI) m/z: $[M+H]^+$: calculated for $C_{13}H_8BrO_2^+$ 274.9702, found 274.9705.

MHAT COUPLING BETWEEN UNBIASED ALKENES AND ELECTRON DEFICIENT ALKYNES

General method for the MHAT reaction between non-activated alkenes and electron deficient alkynes: To a solution of the selected alkene (2.0 equiv.) in ethanol (0.10 M) was added the selected electron deficient alkyne (1.0 equiv.) and Fe(acac)$_3$ (1.0 equiv.). The resulting mixture was degassed for 5 minutes with argon and, subsequently, PhSiH$_3$ (1.5 equiv.) was added dropwise. The suspension was heated at 60 °C with a heating mantle for 16 hours and the resulting mixture was concentrated. The corresponding product was purified by flash column chromatography on silica gel.

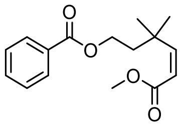

(Z)-6-methoxy-3,3-dimethyl-6-oxohex-4-en-1-yl benzoate (**3a**). The title compound was prepared according to a modification of the general procedure using 3-methylbut-3-en-1-yl benzoate (2.28 g, 12.0 mmol, 3.0 equiv.), methyl propiolate (336 mg, 0.35 mL, 4.0 mmol, 1.0 equiv.), Fe(acac)$_3$ (1.41 g, 4.0 mmol, 1.0 equiv.) and PhSiH$_3$ (649 mg, 0.74 mL, 6.0 mmol, 1.5 equiv.) in ethanol (40 mL, 0.1 M). Purification by chromatography (Hex → Hex/AcOEt 25%) gave **3/4a** (64:36) (1.07 g, 97%) as a colourless oil; ^{1}H NMR (400 MHz, CDCl$_3$) δ 8.06 – 7.99 (m, 2H), 7.58 – 7.51 (m, 1H), 7.47 – 7.40 (m, 2H), 6.03 (d, *J* = 13.0 Hz, 1H), 5.75 (d, *J* = 13.1 Hz, 1H), 4.37 (t, *J* = 7.1 Hz, 2H), 3.70 (s, 3H), 2.09 (t, *J* = 7.1 Hz, 2H), 1.28 (s, 6H); ^{13}C{^{1}H} NMR (101 MHz, CDCl$_3$) δ 166.9, 166.7, 153.8, 133.0, 130.5, 129.7, 128.5, 119.7, 62.7, 51.5, 40.6, 36.2, 28.0 ppm. HRMS (ESI) m/z: [M+H]$^+$: calculated for $C_{16}H_{21}O_4^+$ 277.1434, found 277.1433.

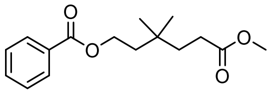

(Z)-6-methoxy-3,3-dimethyl-6-oxohexyl benzoate (**5**). The title compound was isolated during the synthesis of **3**/**4a** according to general procedure using 3-methylbut-3-en-1-yl benzoate (2.28 g, 12.0 mmol, 3.0 equiv.), methyl propiolate (336 mg, 0.35 mL, 4.0 mmol, 1.0 equiv.), Fe(acac)$_3$ (1.41 g, 4.0 mmol, 1.0 equiv.) and PhSiH$_3$ (649 mg, 0.74 mL, 6.0 mmol, 1.5 equiv.) in ethanol (40 mL, 0.1 M). Purification by chromatography (Hex → Hex/AcOEt 25%) gave **5** (33 mg, 3%) as a colourless oil; ^{1}H NMR (400 MHz, CDCl$_3$) δ 8.08 – 7.98 (m, 2H), 7.61 – 7.50 (m, 1H), 7.47 – 7.37 (m, 2H), 4.38 (t, *J* = 7.3 Hz, 2H), 3.65 (s, 3H), 2.38 – 2.28 (m, 2H), 1.71 (t, *J* = 7.2 Hz, 2H), 1.68 – 1.63 (m, 2H), 0.98 (s, 6H); ^{13}C{^{1}H} NMR (101 MHz, CDCl$_3$) δ 174.6, 166.8, 133.0, 130.5, 129.7, 128.5, 62.2, 51.7, 39.7, 37.0, 32.2, 29.5, 27.1 ppm. HRMS (ESI) m/z: [M+H]$^+$: calculated for $C_{16}H_{23}O_4^+$ 279.1591, found 279.1592.

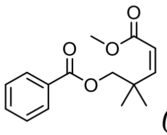

(Z)-5-methoxy-2,2-dimethyl-5-oxopent-3-en-1-yl benzoate (**3b**). The title compound was prepared according to general procedure using 2-methylallyl benzoate (282 mg, 1.60 mmol, 2.0 equiv.), methyl propiolate (67 mg, 71 μL, 0.80 mmol, 1.0 equiv.), Fe(acac)$_3$ (283 mg, 0.80 mmol, 1.0 equiv.) and PhSiH$_3$ (130 mg, 148 μL, 1.20 mmol, 1.5 equiv.) in ethanol (8 mL, 0.1 M). Purification by chromatography (Hex → Hex/AcOEt 25%) gave **3/4b** (70:30) (191 mg, 91%) as a colourless oil; ^{1}H NMR (400 MHz, CDCl$_3$) δ 8.07 – 8.00 (m, 2H), 7.60 – 7.51 (m, 1H), 7.49 – 7.39 (m, 2H), 6.11 (d, *J* = 13.0 Hz, 1H), 5.83 (d, *J* = 13.0 Hz, 1H), 4.33 (s, 2H), 3.70

(s, 3H), 1.34 (s, 6H); $^{13}C\{^1H\}$ NMR (101 MHz, $CDCl_3$) δ 166.7, 166.6, 151.2, 133.1, 130.4, 129.7, 128.5, 120.7, 72.6, 51.6, 37.8, 24.5 ppm. HRMS (ESI) m/z: $[M+H]^+$: calculated for $C_{15}H_{19}O_4^+$ 263.1278, found 263.1277.

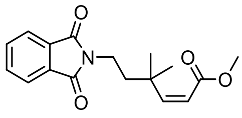

(Z)-methyl-6-(1,3-dioxoisoindolin-2-yl)-4,4-dimethylhex-2-enoate (**3c**). The title compound was prepared according to general procedure using 2-(3-methylbut-3-en-1-yl)isoindoline-1,3-dione (344 mg, 1.60 mmol, 2.0 equiv.), methyl propiolate (67 mg, 71 μL, 0.80 mmol, 1.0 equiv.), $Fe(acac)_3$ (283 mg, 0.80 mmol, 1.0 equiv.) and $PhSiH_3$ (130 mg, 148 μL, 1.20 mmol, 1.5 equiv.) in ethanol (8 mL, 0.1 M). Purification by chromatography (Hex → Hex/AcOEt 25%) gave **3/4c** (60:40) (222 mg, 92%) as a colourless oil; ^1H NMR (400 MHz, $CDCl_3$) δ 7.84 – 7.78 (m, 2H), 7.71 – 7.65 (m, 2H), 6.01 (d, J = 13.1 Hz, 1H), 5.75 (d, J = 13.1 Hz, 1H), 3.71 (s, 3H), 3.70 – 3.64 (m, 2H), 1.95 – 1.86 (m, 2H), 1.26 (s, 6H); $^{13}C\{^1H\}$ NMR (101 MHz, $CDCl_3$) δ 168.3, 166.9, 152.9, 133.9, 132.3, 123.2, 120.0, 51.5, 40.5, 36.3, 34.8, 27.5 ppm. HRMS (ESI) m/z: $[M+H]^+$: calculated for $C_{17}H_{20}NO_4^+$ 302.1387, found 302.1387.

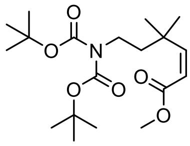

(Z)-methyl-6-((di-tert-butoxycarbonyl)amino)-4,4-dimethylhex-2-enoate (**3d**). The title compound was prepared according to general procedure using di-*tert*-butyl (3-methylbut-3-en-1-yl)carbamate (457 mg, 1.60 mmol, 2.0 equiv.), methyl propiolate (67 mg, 71 μL, 0.80 mmol, 1.0 equiv.), $Fe(acac)_3$ (283 mg, 0.80 mmol, 1.0 equiv.) and $PhSiH_3$ (130 mg, 148 μL, 1.20 mmol, 1.5 equiv.) in ethanol (8 mL, 0.1 M). Purification by chromatography (Hex → Hex/AcOEt 25%) gave **3/4d** (70:30) (282 mg, 95%) as a colourless oil; ^1H NMR (400 MHz, $CDCl_3$) δ 5.97 (d, J = 13.1 Hz, 1H), 5.73 (d, J = 13.1 Hz, 1H), 3.69 (s, 3H), 3.59 – 3.52 (m, 2H), 1.79 – 1.72 (m, 2H), 1.49 (s, 18H), 1.20 (s, 6H). $^{13}C\{^1H\}$ NMR (101 MHz, $CDCl_3$) δ 167.0, 153.2, 152.5, 119.6, 82.2, 51.4, 43.4, 41.6, 36.2, 28.2, 27.0 ppm. HRMS (ESI) m/z: $[M+H]^+$: calculated for $C_{19}H_{34}NO_6^+$ 372.2381, found 372.2383.

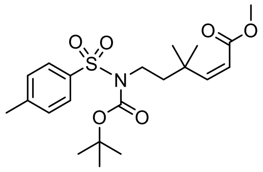

(Z)-methyl-6-((N-(tert-butoxycarbonyl)-4-methylphenyl)sulfonamido)-4,4-dimethylhex-2-enoate (**3e**). The title compound was prepared according to general procedure using tert-butyl (3-methylbut-3-en-1-yl)(tosyl)carbamate (407 mg, 1.20 mmol, 2.0 equiv.), methyl propiolate (50 mg, 54 μL, 0.60 mmol, 1.0 equiv.), $Fe(acac)_3$ (212 mg, 0.60 mmol, 1.0 equiv.) and $PhSiH_3$ (97 mg, 110 μL, 0.90 mmol, 1.5 equiv.) in ethanol (6 mL, 0.1 M). Purification by chromatography (Hex → Hex/AcOEt 25%) gave **3/4e** (69:31) (232 mg, 91%) as a colourless oil; ^1H NMR (400 MHz, $CDCl_3$) δ 7.81 – 7.74 (m, 2H), 7.29 (d, J = 8.0 Hz, 2H), 6.01 (d, J = 13.1 Hz, 1H), 5.78 (d, J = 13.1 Hz, 1H), 3.85 – 3.78 (m, 2H), 3.71 (s, 3H), 2.42 (s, 3H), 2.03 – 1.94 (m, 2H), 1.32 (s, 9H), 1.25 (s, 6H); $^{13}C\{^1H\}$ NMR (101 MHz, $CDCl_3$) δ 167.0, 152.7, 150.9, 144.1, 137.6, 129.3, 128.0, 120.0, 84.2, 51.5, 44.2, 42.5, 36.4, 28.0, 27.3, 21.7 ppm. HRMS (ESI) m/z: $[M+H]^+$: calculated for $C_{21}H_{32}NO_6S^+$ 426.1945, found 426.1948.

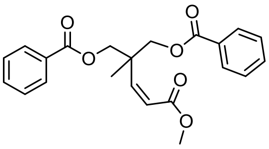

(Z)-2-(3-methoxy-3-oxoprop-1-en-1-yl)-2-methylpropane-1,3-diyl dibenzoate (**3f**). The title compound was prepared according to a modification of the general procedure using 2-methylenepropane-1,3-diyl dibenzoate (1.19 g, 4.00 mmol, 5.0 equiv.), methyl propiolate (67 mg, 71 µL, 0.80 mmol, 1.0 equiv.), $Fe(acac)_3$ (283 mg, 0.80 mmol, 1.0 equiv.) and $PhSiH_3$ (43 mg, 49 µL, 0.40 mmol, 0.5 equiv.) in ethanol (8 mL, 0.1 M). Purification by chromatography (Hex → Hex/AcOEt 25%) gave **3/4f** (65:35) (205 mg, 67%) as a colourless oil; ^{1}H NMR (400 MHz, $CDCl_3$) δ 8.05 – 7.98 (m, 4H), 7.59 – 7.51 (m, 2H), 7.46 – 7.39 (m, 4H), 6.17 (d, *J* = 13.1 Hz, 1H), 5.99 (d, *J* = 13.1 Hz, 1H), 4.61 (d, *J* = 0.8 Hz, 4H), 3.71 (s, 3H), 1.49 (s, 3H); ^{13}C{^{1}H} NMR (101 MHz, $CDCl_3$) δ 166.4, 166.2, 146.9, 133.2, 130.0, 129.8, 128.6, 122.9, 68.1, 51.8, 41.6, 19.3 ppm. HRMS (ESI) m/z: $[M+H]^+$: calculated for $C_{22}H_{23}O_6^+$ 383.1489, found 383.1489.

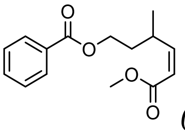

(Z)-6-methoxy-3-methyl-6-oxohex-4-en-1-yl benzoate (**3g**). The title compound was prepared according to a modification of the general procedure using but-3-en-1-yl benzoate (529 mg, 3.0 mmol, 3.0 equiv.), methyl propiolate (84 mg, 88 µL, 1.00 mmol, 1.0 equiv.), $Fe(acac)_3$ (353 mg, 1.00 mmol, 1.0 equiv.) and $PhSiH_3$ (162 mg, 184 µL, 1.50 mmol, 1.5 equiv.) in ethanol (10 mL, 0.1 M). Purification by chromatography (Hex → Hex/AcOEt 25%) gave **3/4g** (59:41) (205 mg, 78%) as a colourless oil; ^{1}H NMR (400 MHz, $CDCl_3$) δ 8.08 – 8.00 (m, 2H), 7.59 – 7.50 (m, 1H), 7.48 – 7.39 (m, 2H), 6.03 (dd, *J* = 11.5, 10.2 Hz, 1H), 5.77 (dd, *J* = 11.5, 0.9 Hz, 1H), 4.38 – 4.24 (m, 2H), 3.85 – 3.71 (m, 1H), 3.64 (s, 3H), 1.95 – 1.83 (m, 1H), 1.82 – 1.68 (m, 1H), 1.10 (d, *J* = 6.7 Hz, 3H). ^{13}C{^{1}H} NMR (101 MHz, $CDCl_3$) δ 166.7, 154.5, 132.9, 130.5, 129.7, 128.4, 119.0, 63.3, 51.2, 35.8, 30.1, 20.5 ppm. HRMS (ESI) m/z: $[M+H]^+$: calculated for $C_{15}H_{19}O_4^+$ 263.1278, found 263.1279.

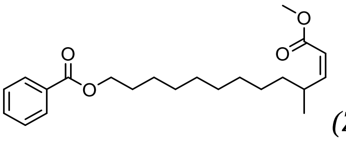

(Z)-13-methoxy-10-methyl-13-oxotridec-11-en-1-yl benzoate (**3h**). The title compound was prepared according to a modification of the general procedure using undec-10-en-1-yl benzoate (659 mg, 2.40 mmol, 3.0 equiv.), methyl propiolate (67 mg, 71 µL, 0.80 mmol, 1.0 equiv.), $Fe(acac)_3$ (283 mg, 0.80 mmol, 1.0 equiv.) and $PhSiH_3$ (130 mg, 148 µL, 1.20 mmol, 1.5 equiv.) in ethanol (8 mL, 0.1 M). Purification by chromatography (Hex → Hex/AcOEt 25%) gave **3/4h** (36:64) (231 mg, 80%) as a colourless oil; ^{1}H NMR (400 MHz, $CDCl_3$) δ 8.08 – 8.00 (m, 2H), 7.58 – 7.51 (m, 1H), 7.48 – 7.39 (m, 2H), 5.97 (dd, *J* = 11.6, 10.2 Hz, 1H), 5.71 (dd, *J* = 11.5, 0.9 Hz, 1H), 4.31 (t, *J* = 6.7 Hz, 2H), 3.70 (s, 3H), 3.54 – 3.42 (m, 1H), 1.81 – 1.70 (m, 2H), 1.48 – 1.37 (m, 2H), 1.38 – 1.19 (m, 12H), 0.99 (d, *J* = 6.7 Hz, 3H); ^{13}C{^{1}H} NMR (101 MHz, $CDCl_3$) δ 167.0, 166.8, 156.8, 132.9, 130.7, 129.7, 128.5, 117.9, 65.3, 51.1, 37.1, 32.9, 29.8, 29.6, 29.4, 28.9, 27.5, 26.2, 20.4 ppm. HRMS (ESI) m/z: $[M+H]^+$: calculated for $C_{22}H_{33}O_4^+$ 361.2373, found 361.2377.

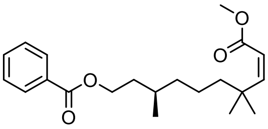

(Z),(R)-10-methoxy-3,7,7-trimethyl-10-oxodec-8-en-1-yl benzoate (**3i**). The title compound was prepared according to a modification of the general procedure using (*R)*-3,7-dimethyloct-6-en-1-yl benzoate (1.04 g, 4.00 mmol, 5.0 equiv.), methyl propiolate (67 mg, 71 μL, 0.80 mmol, 1.0 equiv.), Fe(acac)$_3$ (283 mg, 0.80 mmol, 1.0 equiv.) and $PhSiH_3$ (43 mg, 49 μL, 0.40 mmol, 0.5 equiv.) in ethanol (8 mL, 0.1 M). Purification by chromatography (Hex → Hex/AcOEt 25%) gave **3/4i** (72:28) (147 mg, 53%) as a colourless oil; ^{1}H NMR (400 MHz, $CDCl_3$) δ 8.07 – 8.00 (m, 2H), 7.59 – 7.51 (m, 1H), 7.48 – 7.39 (m, 2H), 5.92 (d, *J* = 13.1 Hz, 1H), 5.69 (d, *J* = 13.1 Hz, 1H), 4.42 – 4.28 (m, 1H), 3.69 (s, 3H), 1.86 – 1.74 (m, 1H), 1.71 – 1.60 (m, 1H), 1.61 – 1.51 (m, 1H), 1.52 – 1.42 (m, 2H), 1.39 – 1.15 (m, 4H), 1.15 (s, 6H), 0.95 (d, *J* = 6.5 Hz, 3H); ^{13}C{^{1}H} NMR (101 MHz, $CDCl_3$) δ 167.4, 166.8, 154.4, 132.9, 130.7, 129.7, 128.5, 118.9, 63.7, 51.4, 43.4, 37.8, 37.2, 35.7, 30.1, 27.3, 22.2, 19.7 ppm. HRMS (ESI) m/z: $[M+H]^+$: calculated for $C_{21}H_{31}O_4^+$ 347.2217, found 347.2218.

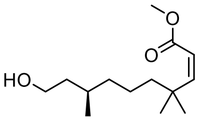

Methyl (R,Z)-10-hydroxy-4,4,8-trimethyldec-2-enoate (**3j**). The title compound was prepared according to a modification of the general procedure using *(R)*-3,7-dimethyloct-6-en-1-ol (938 mg, 6.00 mmol, 5.0 equiv.), methyl propiolate (101 mg, 106 μL, 1.20 mmol, 1.0 equiv.), Fe(acac)$_3$ (424 mg, 1.20 mmol, 1.0 equiv.) and $PhSiH_3$ (65 mg, 74 μL, 0.60 mmol, 0.5 equiv.) in ethanol (12 mL, 0.1 M). Purification by chromatography (Hex → Hex/AcOEt 25%) gave **3/4j** (73:27) (145 mg, 50%) as a colourless oil; ^{1}H NMR (400 MHz, $CDCl_3$) δ 5.92 (d, *J* = 13.1 Hz, 1H), 5.69 (d, *J* = 13.1 Hz, 1H), 3.69 (s, 3H), 3.69 – 3.58 (m, 2H), 1.65 – 1.49 (m, 3H), 1.51 – 1.39 (m, 2H), 1.40 – 1.30 (m, 1H), 1.32 – 1.18 (m, 3H), 1.14 (s, 6H), 1.14 – 1.07 (m, 1H), 0.87 (d, *J* = 6.6 Hz, 3H); ^{13}C{^{1}H} NMR (101 MHz, $CDCl_3$) δ 167.4, 154.5, 118.8, 61.3, 51.4, 43.3, 40.0, 37.9, 37.2, 29.5, 27.4, 27.3, 22.2, 19.8 ppm. HRMS (ESI) m/z: $[M+H]^+$: calculated for $C_{14}H_{27}O_3^+$ 243.1955, found 243.1957.

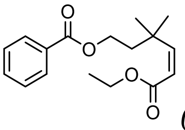

(Z)-6-ethoxy-3,3-dimethyl-6-oxohex-4-en-1-yl benzoate (**3k**). The title compound was prepared according to general procedure using 3-methylbut-3-en-1-yl benzoate (304 mg, 1.60 mmol, 2.0 equiv.), ethyl propiolate (78 mg, 80 μL, 0.80 mmol, 1.0 equiv.), Fe(acac)$_3$ (283 mg, 0.80 mmol, 1.0 equiv.) and $PhSiH_3$ (130 mg, 148 μL, 1.20 mmol, 1.5 equiv.) in ethanol (8 mL, 0.1 M). Purification by chromatography (Hex → Hex/AcOEt 25%) gave **3/4k** (62:38) (221 mg, 95%) as a colourless oil; ^{1}H NMR (400 MHz, $CDCl_3$) δ 8.06 – 7.99 (m, 2H), 7.54 (ddt, *J* = 8.0, 6.8, 1.4 Hz, 1H), 7.47 – 7.39 (m, 2H), 6.00 (d, *J* = 13.1 Hz, 1H), 5.75 (d, *J* = 13.1 Hz, 1H), 4.37 (t, *J* = 7.1 Hz, 2H), 4.16 (q, *J* = 7.1 Hz, 2H), 2.09 (t, *J* = 7.2 Hz, 2H), 1.31 – 1.25 (m, 9H); ^{13}C{^{1}H} NMR (101 MHz, $CDCl_3$) δ 166.7, 166.6, 153.1, 133.0, 130.6, 129.7, 128.4, 120.2, 62.7, 60.4, 40.6, 36.2, 28.0, 14.3 ppm. HRMS (ESI) m/z: $[M+H]^+$: calculated for $C_{17}H_{23}O_4^+$ 291.1591, found 291.1590.

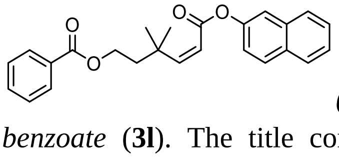

(Z)-3,3-dimethyl-6-(naphthalen-2-yloxy)-6-oxohex-4-en-1-yl benzoate (**3l**). The title compound was prepared according to general procedure using 3-

methylbut-3-en-1-yl benzoate (304 mg, 1.60 mmol, 2.0 equiv.), naphthalen-2-yl propiolate (157 mg, 0.80 mmol, 1.0 equiv.), $Fe(acac)_3$ (283 mg, 0.80 mmol, 1.0 equiv.) and $PhSiH_3$ (130 mg, 148 µL, 1.20 mmol, 1.5 equiv.) in ethanol (8 mL, 0.1 M). Purification by chromatography (Hex → Hex/AcOEt 25%) gave **3/4l** (52:48) (249 mg, 80%) as a colourless oil; ^{1}H NMR (400 MHz, $CDCl_3$) δ 8.07 – 8.00 (m, 2H), 7.87 – 7.74 (m, 3H), 7.58 (d, *J* = 2.3 Hz, 1H), 7.54 (t, *J* = 7.4 Hz, 1H), 7.51 – 7.37 (m, 4H), 7.28 – 7.22 (m, 1H), 6.28 (d, *J* = 12.9 Hz, 1H), 6.04 (d, *J* = 13.0 Hz, 1H), 4.43 (t, *J* = 7.1 Hz, 2H), 2.17 (t, *J* = 7.1 Hz, 2H), 1.37 (s, 6H); ^{13}C{^{1}H} NMR (101 MHz, $CDCl_3$) δ 166.7, 164.6, 157.1, 148.3, 133.9, 133.0, 131.6, 130.5, 129.7, 129.5, 128.5, 127.9, 127.8, 126.6, 125.8, 121.3, 119.3, 118.7, 62.6, 40.4, 36.4, 28.1 ppm. HRMS (ESI) m/z: $[M+H]^+$: calculated for $C_{25}H_{25}O_4^+$ 389.1747, found 389.1749.

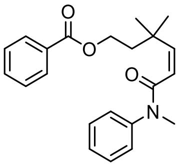

(Z)-3,3-dimethyl-6-(methyl(phenyl)amino)-6-oxohex-4-en-1-yl benzoate (**3m**). The title compound was prepared according to general procedure using 3-methylbut-3-en-1-yl benzoate (304 mg, 1.60 mmol, 2.0 equiv.), *N*-methyl-*N*-phenylpropiolamide (127 mg, 0.8 mmol, 1.0 equiv.), $Fe(acac)_3$ (283 mg, 0.80 mmol, 1.0 equiv.) and $PhSiH_3$ (130 mg, 148 µL, 1.20 mmol, 1.5 equiv.) in ethanol (8 mL, 0.1 M). Purification by chromatography (Hex → Hex/AcOEt 25%) gave **3/4m** (58:42) (267 mg, 95%) as a yellow oil; ^{1}H NMR (400 MHz, $CDCl_3$) δ 8.05 – 7.98 (m, 1.7H), 7.98 – 7.94 (m, 0.3H), 7.71 – 7.67 (m, 0.1H), 7.57 – 7.51 (m, 1.0H), 7.42 (t, *J* = 7.6 Hz, 2.0H), 7.36 (t, *J* = 7.5 Hz, 1.7H), 7.30 – 7.22 (m, 1.2H), 7.17 (d, *J* = 7.7 Hz, 1.5H), 7.03 (td, *J* = 7.7, 1.1 Hz, 0.2H), 6.83 – 6.77 (m, 0.1H), 5.60 (d, *J* = 13.0 Hz, 0.8H), 5.52 (d, *J* = 13.0 Hz, 0.8H), 4.44 (t, *J* = 6.8 Hz, 0.3H), 4.31 (t, *J* = 7.2 Hz, 1.5H), 3.32 (s, 2.4H), 3.20 (s, 0.4H), 2.27 (t, *J* = 6.8 Hz, 0.3H), 1.98 (t, *J* = 7.2 Hz, 1.7H), 1.48 (s, 0.8H), 1.25 (s, 5.7H); ^{13}C{^{1}H} NMR (101 MHz, $CDCl_3$) δ 168.7, 167.7, 166.7, 166.6, 151.1, 146.6, 144.6, 143.9, 132.9, 132.9, 130.6, 130.2, 129.7, 129.5, 129.2, 128.4, 127.5, 127.0, 126.3, 122.9, 121.9, 120.5, 108.3, 62.7, 62.2, 41.4, 39.8, 36.9, 36.2, 35.1, 29.8, 27.6, 26.3 ppm. HRMS (ESI) m/z: $[M+H]^+$: calculated for $C_{22}H_{26}NO_3^+$ 352.1907, found 352.1908.

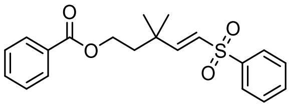

(E)-3,3-dimethyl-5-(phenylsulfonyl)pent-4-en-1-yl benzoate (**4n**). The title compound was prepared according to general procedure using 3-methylbut-3-en-1-yl benzoate (304 mg, 1.60 mmol, 2.0 equiv.), (ethynylsulfonyl)benzene (133 mg, 0.8 mmol, 1.0 equiv.), $Fe(acac)_3$ (283 mg, 0.80 mmol, 1.0 equiv.) and $PhSiH_3$ (130 mg, 148 µL, 1.20 mmol, 1.5 equiv.) in ethanol (8 mL, 0.1 M). Purification by chromatography (Hex → Hex/AcOEt 25%) gave **4n** (149 mg, 52%) as a colourless oil; ^{1}H NMR (400 MHz, $CDCl_3$) δ 8.06 – 7.99 (m, 2H), 7.87 – 7.80 (m, 2H), 7.64 – 7.52 (m, 2H), 7.54 – 7.46 (m, 2H), 7.49 – 7.40 (m, 2H), 7.06 (d, *J* = 15.3 Hz, 1H), 6.28 (d, *J* = 15.3 Hz, 1H), 4.28 (t, *J* = 6.9 Hz, 2H), 1.91 (t, *J* = 6.9 Hz, 2H), 1.17 (s, 6H); ^{13}C{^{1}H} NMR (101 MHz, $CDCl_3$) δ 166.6, 154.5, 140.7, 133.4, 133.2, 130.1, 129.7, 129.4, 128.6, 128.2, 127.7, 61.6, 40.3, 36.4, 26.5 ppm. HRMS (ESI) m/z: $[M+H]^+$: calculated for $C_{20}H_{23}O_4S^+$ 359.1312, found 359.1310.

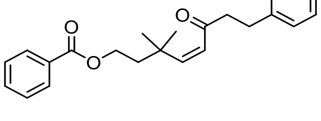

(Z)-3,3-dimethyl-6-oxo-8-phenyloct-4-en-1-yl benzoate (**3o**). The title compound was prepared according to general procedure using 3-methylbut-3-en-1-yl benzoate

(304 mg, 1.60 mmol, 2.0 equiv.), 5-phenylpent-1-yn-3-one (127 mg, 0.8 mmol, 1.0 equiv.), Fe(acac)$_3$ (283 mg, 0.80 mmol, 1.0 equiv.) and PhSiH$_3$ (130 mg, 148 µL, 1.20 mmol, 1.5 equiv.) in ethanol (8 mL, 0.1 M). Purification by chromatography (Hex → Hex/AcOEt 10%) gave **3/4o** (43:57) (264 mg, 94%) as a colourless oil. The product was not stable and isomerized into the *trans* isomer prior to full characterization. HRMS (ESI) m/z: [M+H]$^+$: calculated for $C_{23}H_{27}O_3^+$ 351.1955, found 351.1955.

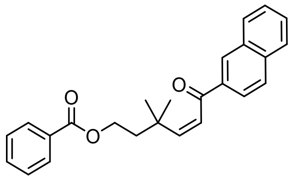

(Z)-3,3-dimethyl-6-(naphthalen-2-yl)-6-oxohex-4-en-1-yl benzoate (**3p**). The title compound was prepared according to general procedure using 3-methylbut-3-en-1-yl benzoate (304 mg, 1.60 mmol, 2.0 equiv.), 1-(naphthalen-2-yl)prop-2-yn-1-one (144 mg, 0.80 mmol, 1.0 equiv.), Fe(acac)$_3$ (283 mg, 0.80 mmol, 1.0 equiv.) and PhSiH$_3$ (130 mg, 148 µL, 1.20 mmol, 1.5 equiv.) in ethanol (8 mL, 0.1 M). Purification by chromatography (Hex → Hex/AcOEt 10%) gave **3/4p** (52:48) (274 mg, 92%) as a colourless oil. However, the product was not stable and isomerized into the *trans* isomer prior to full characterization. HRMS (ESI) m/z: [M+H]$^+$: calculated for $C_{25}H_{25}O_3^+$ 373.1798, found 373.1804.

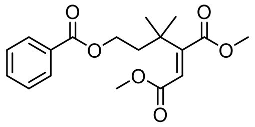

(Z)-dimethyl 2-(4-(benzoyloxy)-2-methylbutan-2-yl)fumarate (**3q**). The title compound was prepared according to general procedure using 3-methylbut-3-en-1-yl benzoate (304 mg, 1.60 mmol, 2.0 equiv.), dimethyl but-2-ynedioate (114 mg, 98 µL, 0.80 mmol, 1.0 equiv.), Fe(acac)$_3$ (283 mg, 0.80 mmol, 1.0 equiv.) and PhSiH$_3$ (130 mg, 148 µL, 1.20 mmol, 1.5 equiv.) in ethanol (8 mL, 0.1 M). Purification by chromatography (Hex → Hex/AcOEt 25%) gave **3/4q** (71:29) (203 mg, 76%) as a colourless oil; ^{1}H NMR (500 MHz, CDCl$_3$) δ 8.01 (dd, *J* = 8.4, 1.4 Hz, 2H), 7.57 – 7.50 (m, 1H), 7.45 – 7.38 (m, 2H), 6.38 (s, 1H), 4.37 (t, *J* = 7.0 Hz, 2H), 3.72 (s, 3H), 3.72 (s, 3H), 2.23 (t, *J* = 7.0 Hz, 2H), 1.32 (s, 6H); ^{13}C{^{1}H} NMR (126 MHz, CDCl$_3$) δ 168.8, 167.3, 166.6, 149.0, 132.9, 130.6, 129.6, 128.4, 125.2, 62.5, 52.2, 52.1, 39.7, 38.0, 27.7 ppm. HRMS (ESI) m/z: [M+H]$^+$: calculated for $C_{18}H_{23}O_6^+$ 335.1489, found 335.1488.

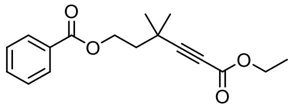

6-ethoxy-3,3-dimethyl-6-oxohex-4-yn-1-yl benzoate (**6**). The title compound was prepared according to a modification of the general procedure using 3-methylbut-3-en-1-yl benzoate (304 mg, 1.60 mmol, 2.0 equiv.), ethyl 3-bromopropiolate (142 mg, 0.80 mmol, 1.0 equiv.), Fe(acac)$_3$ (283 mg, 0.80 mmol, 1.0 equiv.), NaHCO$_3$ (67 mg, 0.80 mmol, 1.0 equiv.), and PhSiH$_3$ (130 mg, 148 µL, 1.20 mmol, 1.5 equiv.) in ethanol (8 mL, 0.1 M). Purification by chromatography (Hex → Hex/AcOEt 25%) gave **6** (143 mg, 62%) as a colourless oil; ^{1}H NMR (400 MHz, CDCl$_3$) δ 8.07 – 8.01 (m, 2H), 7.59 – 7.51 (m, 1H), 7.48 – 7.39 (m, 2H), 4.51 (t, *J* = 6.8 Hz, 2H), 4.18 (q, *J* = 7.1 Hz, 2H), 1.99 (t, *J* = 6.8 Hz, 2H), 1.37 (s, 6H), 1.28 (t, *J* = 7.1 Hz, 3H); ^{13}C{^{1}H} NMR (101 MHz, CDCl$_3$) δ 166.6, 154.0, 133.1, 130.3, 129.7, 128.5, 94.0, 73.7, 62.3, 62.0, 40.7, 30.2, 28.8, 14.2 ppm. HRMS (ESI) m/z: [M+H]$^+$: calculated for $C_{17}H_{21}O_4^+$ 289.1434, found 289.1432.

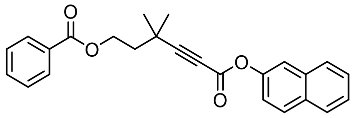 *3,3-dimethyl-6-(naphthalen-2-yloxy)-6-oxohex-4-yn-1-yl benzoate* (**7**). The title compound was prepared according to a modification of the general procedure using 3-methylbut-3-en-1-yl benzoate (228 mg, 1.20 mmol, 2.0 equiv.), naphthalen-2-yl 3-bromopropiolate (165 mg, 0.60 mmol, 1.0 equiv.), Fe(acac)$_3$ (212 mg, 0.60 mmol, 1.0 equiv.), $NaHCO_3$ (50 mg, 0.60 mmol, 1.0 equiv.), and $PhSiH_3$ (65 mg, 74 μL, 0.60 mmol, 1.0 equiv.) in ethanol (6 mL, 0.1 M). Purification by chromatography (Hex → Hex/AcOEt 25%) gave **7** (116 mg, 50%) as a colourless oil; ^{1}H NMR (400 MHz, $CDCl_3$) δ 8.12 – 8.04 (m, 2H), 7.89 – 7.76 (m, 3H), 7.58 – 7.52 (m, 2H), 7.49 (ddd, *J* = 7.6, 5.3, 1.6 Hz, 2H), 7.44 (t, *J* = 7.6 Hz, 2H), 7.23 (dd, *J* = 8.9, 2.3 Hz, 1H), 4.55 (t, *J* = 6.7 Hz, 2H), 2.04 (t, *J* = 6.8 Hz, 2H), 1.43 (s, 6H); ^{13}C{^{1}H} NMR (101 MHz, $CDCl_3$) δ 166.7, 152.3, 147.9, 133.8, 133.1, 131.8, 130.3, 129.8, 129.7, 128.5, 127.9, 127.9, 126.8, 126.1, 120.9, 118.7, 97.1, 73.3, 62.1, 40.7, 30.4, 28.7 ppm. HRMS (ESI) m/z: [M+H]$^+$: calculated for $C_{25}H_{23}O_4^+$ 387.1591, found 387.1591.

ISOMERIZATION OF THE E/Z MIXTURES OF THE MHAT COUPLING

General method for the isomerization of the *cis*/*trans* mixtures of the MHAT reaction between non-activated alkenes and electron deficient alkynes: To the purified *cis*/*trans* mixture (1.0 equiv.) obtained in the MHAT coupling was added triethylamine (1.0 equiv.) and thiophenol (2.0 equiv.) and the resulting mixture was stirred at 40 °C in an oil bath for 24 hours. Then, triethylamine was evaporated by heating at 70 °C in an oil bath and applying vacuum for 30 minutes, the crude mixture was dissolved in MeOH/H_2O (10:1, 0.06 M) and sodium periodate (3.0 equiv.) was added portionwise. After stirring the solution for 24 hours at room temperature, the mixture was extracted with dichloromethane. The organic layer was dried with sodium sulphate and concentrated. The corresponding product was purified by flash column chromatography on silica gel.

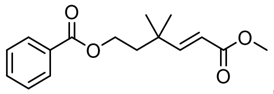
(E)-6-methoxy-3,3-dimethyl-6-oxohex-4-en-1-yl benzoate (**4a**). The title compound was prepared according to general procedure using the **3a** and **4a** mixture (100 mg, 0.36 mmol, 1.0 equiv.), triethylamine (36 mg, 50 µL, 0.36 mmol, 1.0 equiv.), thiophenol (79 mg, 73 µL, 0.72 mmol, 2.0 equiv.) and sodium periodate (231 mg, 1.08 mmol, 3.0 equiv.) in 10:1 MeOH/H_2O (6 mL, 0.06 M).

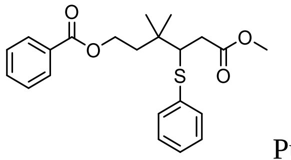
Purification by chromatography of the Michael adduct (Hex → Hex/AcOEt 10%) gave **SI-B** (139 mg, 100%) as a colourless oil; ^{1}H NMR (400 MHz, $CDCl_3$) δ 8.05 – 7.97 (m, 2H), 7.59 – 7.47 (m, 3H), 7.47 – 7.38 (m, 2H), 7.30 – 7.21 (m, 2H), 7.22 – 7.13 (m, 1H), 4.43 – 4.25 (m, 2H), 3.63 – 3.56 (m, 4H), 2.86 (dd, *J* = 15.9, 4.1 Hz, 1H), 2.59 (dd, *J* = 15.9, 10.0 Hz, 1H), 2.01 (ddd, *J* = 14.1, 8.2, 6.1 Hz, 1H), 1.87 (ddd, *J* = 13.9, 8.3, 6.5 Hz, 1H), 1.11 (s, 3H), 1.10 (s, 3H); ^{13}C{^{1}H} NMR (101 MHz, $CDCl_3$) δ 172.6, 166.7, 136.6, 133.0, 131.7, 130.4, 129.7, 129.1, 128.4, 127.0, 62.0, 56.9, 51.9, 38.1, 38.0, 37.4, 25.2, 25.1 ppm. HRMS (ESI) m/z: $[M+H]^+$: calculated for $C_{22}H_{27}O_4S^+$ 387.1625, found 387.1627.

Purification by chromatography of the oxidation step (Hex → Hex/AcOEt 10%) gave **4a** (97 mg, 97%) as a colourless oil; ^{1}H NMR (400 MHz, $CDCl_3$) δ 8.03 – 7.97 (m, 2H), 7.58 – 7.50 (m, 1H), 7.42 (t, *J* = 7.6 Hz, 2H), 7.00 (d, *J* = 15.9 Hz, 1H), 5.77 (d, *J* = 16.0 Hz, 1H), 4.31 (t, *J* = 6.9 Hz, 2H), 3.68 (s, 3H), 1.89 (t, *J* = 6.9 Hz, 2H), 1.16 (s, 6H); ^{13}C{^{1}H} NMR (101 MHz, $CDCl_3$) δ 167.4, 166.6, 157.3, 133.0, 130.3, 129.7, 128.5, 118.0, 62.0, 51.6, 40.6, 36.0, 26.8 ppm. HRMS (ESI) m/z: $[M+H]^+$: calculated for $C_{16}H_{21}O_4^+$ 277.1434, found 277.1436.

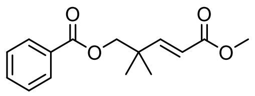
(E)-5-methoxy-2,2-dimethyl-5-oxopent-3-en-1-yl benzoate (**4b**). The title compound was prepared according to general procedure using the **3b** and **4b** mixture (94 mg, 0.36 mmol, 1.0 equiv.), triethylamine (36 mg, 50 µL, 0.36 mmol, 1.0 equiv.), thiophenol (79 mg, 73 µL, 0.72 mmol, 2.0 equiv.) and sodium periodate (231 mg, 1.08 mmol, 3.0 equiv.) in 10:1 MeOH/H_2O (6 mL, 0.06 M). Purification by chromatography (Hex → Hex/AcOEt 10%) gave **4b** (93 mg, 98%) as a colourless oil; ^{1}H NMR (400 MHz, $CDCl_3$) δ 8.05 – 7.98 (m, 2H), 7.60 – 7.53

(m, 1H), 7.48 – 7.40 (m, 2H), 7.04 (d, J = 16.0 Hz, 1H), 5.90 (d, J = 16.0 Hz, 1H), 4.17 (s, 2H), 3.74 (s, 3H), 1.21 (s, 6H); $^{13}C\{^{1}H\}$ NMR (101 MHz, $CDCl_3$) δ 167.3, 166.5, 154.4, 133.2, 130.1, 129.7, 128.6, 119.4, 71.7, 51.8, 37.7, 24.0 ppm. HRMS (ESI) m/z: $[M+H]^+$: calculated for $C_{15}H_{19}O_4^+$ 263.1278, found 263.1278.

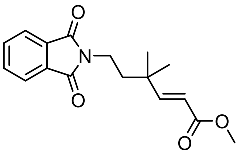

Methyl (E)-6-(1,3-dioxoisoindolin-2-yl)-4,4-dimethylhex-2-enoate (**4c**). The title compound was prepared according to general procedure using the **3c** and **4c** mixture (108 mg, 0.36 mmol, 1.0 equiv.) triethylamine (36 mg, 50 µL, 0.36 mmol, 1.0 equiv.), thiophenol (79 mg, 73 µL, 0.72 mmol, 2.0 equiv.) and sodium periodate (231 mg, 1.08 mmol, 3.0 equiv.) in 10:1 MeOH/H_2O (6 mL, 0.06 M). Purification by chromatography (Hex → Hex/AcOEt 25%) gave **4c** (104 mg, 96%) as a colourless oil; ^{1}H NMR (400 MHz, $CDCl_3$) δ 7.84 – 7.78 (m, 2H), 7.72 – 7.66 (m, 2H), 6.89 (d, J = 16.0 Hz, 1H), 5.77 (d, J = 16.0 Hz, 1H), 3.67 – 3.62 (m, 2H), 3.61 (s, 3H), 1.83 – 1.75 (m, 2H), 1.14 (s, 6H); $^{13}C\{^{1}H\}$ NMR (101 MHz, $CDCl_3$) δ 168.3, 167.2, 156.5, 134.0, 132.2, 123.3, 118.1, 51.5, 39.8, 36.2, 34.5, 26.4 ppm. HRMS (ESI) m/z: $[M+H]^+$: calculated for $C_{17}H_{20}NO_4^+$ 302.1387, found 302.1388.

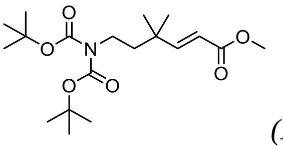

(E)-methyl-6-((di-tert-butoxycarbonyl)amino)-4,4-dimethylhex-2-enoate (**4d**). The title compound was prepared according to general procedure using the **3d** and **4d** mixture (134 mg, 0.36 mmol, 1.0 equiv.), triethylamine (36 mg, 50 µL, 0.36 mmol, 1.0 equiv.), thiophenol (79 mg, 73 µL, 0.72 mmol, 2.0 equiv.) and sodium periodate (231 mg, 1.08 mmol, 3.0 equiv.) in 10:1 MeOH/H_2O (6 mL, 0.06 M). Purification by chromatography (Hex → Hex/AcOEt 25%) gave **4d** (132 mg, 99%) as a colourless oil; ^{1}H NMR (400 MHz, $CDCl_3$) δ 6.91 (d, J = 16.0 Hz, 1H), 5.75 (d, J = 16.0 Hz, 1H), 3.71 (s, 3H), 3.53 – 3.44 (m, 2H), 1.67 – 1.60 (m, 2H), 1.49 (s, 18H), 1.09 (s, 6H); $^{13}C\{^{1}H\}$ NMR (101 MHz, $CDCl_3$) δ 167.4, 157.3, 152.3, 118.0, 82.4, 51.6, 43.1, 40.4, 35.9, 28.2, 26.4 ppm. HRMS (ESI) m/z: $[M+Na]^+$: calculated for $C_{19}H_{33}NNaO_6^+$ 394.2200, found 394.2212.

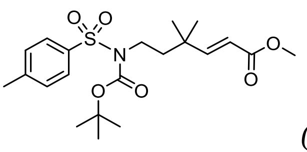

(E)-methyl-6-((N-(tert-butoxycarbonyl)-4-methylphenyl)sulfonamido)-4,4-dimethylhex-2-enoate (**4e**). The title compound was prepared according to general procedure using the **3e** and **4e** mixture (153 mg, 0.36 mmol, 1.0 equiv.), triethylamine (36 mg, 50 µL, 0.36 mmol, 1.0 equiv.), thiophenol (79 mg, 73 µL, 0.72 mmol, 2.0 equiv.) and sodium periodate (231 mg, 1.08 mmol, 3.0 equiv.) in 10:1 MeOH/H_2O (6 mL, 0.06 M). Purification by chromatography (Hex → Hex/AcOEt 25%) gave **4e** (146 mg, 95%) as a colourless oil; ^{1}H NMR (400 MHz, $CDCl_3$) δ 7.79 – 7.72 (m, 2H), 7.32 – 7.27 (m, 2H), 6.95 (d, J = 16.0 Hz, 1H), 5.81 (d, J = 16.0 Hz, 1H), 3.78 – 3.69 (m, 5H), 2.43 (s, 3H), 1.92 – 1.79 (m, 2H), 1.33 (s, 9H), 1.14 (s, 6H); $^{13}C\{^{1}H\}$ NMR (101 MHz, $CDCl_3$) δ 167.4, 156.9, 150.9, 144.3, 137.5, 129.4, 127.9, 118.3, 84.3, 51.6, 43.9, 41.6, 36.1, 28.0, 26.4, 21.7 ppm. HRMS (ESI) m/z: $[M+H]^+$: calculated for $C_{21}H_{32}NO_6S^+$ 426.1945, found 426.1948.

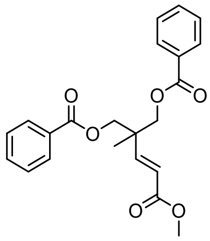 *(E)-2-(3-methoxy-3-oxoprop-1-en-1-yl)-2-methylpropane-1,3-diyl dibenzoate* (**4f**). The title compound was prepared according to general procedure using the mixture of **3f** and **4f** (138 mg, 0.36 mmol, 1.0 equiv.), triethylamine (36 mg, 50 µL, 0.36 mmol, 1.0 equiv.), thiophenol (79 mg, 73 µL, 0.72 mmol, 2.0 equiv.) and sodium periodate (231 mg, 1.08 mmol, 3.0 equiv.) in 10:1 MeOH/H_2O (6 mL, 0.06 M). Purification by chromatography (Hex → Hex/AcOEt 25%) gave **4f** (132 mg, 96%) as a colourless oil; ^{1}H NMR (400 MHz, $CDCl_3$) δ 8.05 – 7.97 (m, 4H), 7.62 – 7.52 (m, 2H), 7.49 – 7.39 (m, 4H), 7.10 (d, *J* = 16.2 Hz, 1H), 6.05 (d, *J* = 16.2 Hz, 1H), 4.50 – 4.34 (m, 4H), 3.75 (s, 3H), 1.34 (s, 3H); ^{13}C{^{1}H} NMR (101 MHz, $CDCl_3$) δ 166.7, 166.3, 149.4, 133.4, 129.8, 128.6, 121.8, 67.7, 51.9, 41.4, 19.5 ppm. HRMS (ESI) m/z: $[M+H]^+$: calculated for $C_{22}H_{23}O_6^+$ 383.1489, found 383.1488.

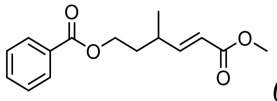 *(E)-6-methoxy-3-methyl-6-oxohex-4-en-1-yl benzoate* (**4g**). The title compound was prepared according to general procedure using the **3g** and **4g** mixture (94 mg, 0.36 mmol, 1.0 equiv.), triethylamine (36 mg, 50 µL, 0.36 mmol, 1.0 equiv.), thiophenol (79 mg, 73 µL, 0.72 mmol, 2.0 equiv.) and sodium periodate (231 mg, 1.08 mmol, 3.0 equiv.) in 10:1 MeOH/H_2O (6 mL, 0.06 M). Purification by chromatography (Hex → Hex/AcOEt 25%) gave **4g** (93 mg, 99%) as a colourless oil; ^{1}H NMR (400 MHz, $CDCl_3$) δ 8.05 – 7.98 (m, 2H), 7.60 – 7.51 (m, 1H), 7.48 – 7.38 (m, 2H), 6.91 (dd, *J* = 15.7, 8.0 Hz, 1H), 5.84 (dd, *J* = 15.7, 1.1 Hz, 1H), 4.33 (qt, *J* = 11.2, 6.5 Hz, 2H), 3.70 (s, 3H), 2.56 (dddd, *J* = 13.7, 8.0, 6.8, 1.2 Hz, 1H), 1.86 (q, *J* = 6.6 Hz, 2H), 1.14 (d, *J* = 6.7 Hz, 3H); ^{13}C{^{1}H} NMR (101 MHz, $CDCl_3$) δ 167.1, 166.6, 153.4, 133.1, 130.3, 129.7, 128.5, 120.2, 62.9, 51.6, 34.9, 33.9, 19.5 ppm. HRMS (ESI) m/z: $[M+H]^+$: calculated for $C_{15}H_{19}O_4^+$ 263.1278, found 263.1277.

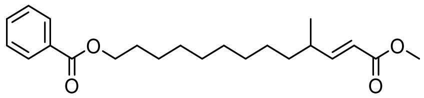 *(E)-13-methoxy-10-methyl-13-oxotridec-11-en-1-yl benzoate* (**4h**). The title compound was prepared according to general procedure using the **3h** and **4h** mixture (130 mg, 0.36 mmol, 1.0 equiv.), triethylamine (36 mg, 50 µL, 0.36 mmol, 1.0 equiv.), thiophenol (79 mg, 73 µL, 0.72 mmol, 2.0 equiv.) and sodium periodate (231 mg, 1.08 mmol, 3.0 equiv.) in 10:1 MeOH/H_2O (6 mL, 0.06 M). Purification by chromatography (Hex → Hex/AcOEt 10%) gave **4h** (123 mg, 95%) as a colourless oil; ^{1}H NMR (500 MHz, $CDCl_3$) δ 8.08 – 8.02 (m, 2H), 7.60 – 7.51 (m, 1H), 7.48 – 7.40 (m, 2H), 6.87 (dd, *J* = 15.7, 7.9 Hz, 1H), 5.77 (dd, *J* = 15.7, 1.2 Hz, 1H), 4.31 (t, *J* = 6.7 Hz, 2H), 3.73 (s, 3H), 2.37 – 2.22 (m, 1H), 1.82 – 1.72 (m, 2H), 1.49 – 1.39 (m, 2H), 1.39 – 1.19 (m, 12H), 1.04 (d, *J* = 6.7 Hz, 3H); ^{13}C{^{1}H} NMR (101 MHz, $CDCl_3$) δ 167.5, 166.8, 155.2, 132.9, 130.7, 129.6, 128.4, 119.3, 65.2, 51.5, 36.7, 36.1, 29.7, 29.6, 29.6, 29.4, 28.8, 27.3, 26.1, 19.5 ppm. HRMS (ESI) m/z: $[M+H]^+$: calculated for $C_{22}H_{33}O_4^+$ 361.2373, found 361.2373.

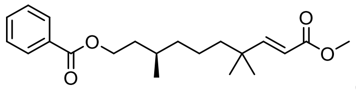 *(R,E)-10-methoxy-3,7,7-trimethyl-10-oxodec-8-en-1-yl benzoate* (**4i**). The title compound was prepared according to general procedure using the **3i** and **4i** mixture

(125 mg, 0.36 mmol, 1.0 equiv.), triethylamine (36 mg, 50 µL, 0.36 mmol, 1.0 equiv.), thiophenol (79 mg, 73 µL, 0.72 mmol, 2.0 equiv.) and sodium periodate (231 mg, 1.08 mmol, 3.0 equiv.) in 10:1 MeOH/H_2O (6 mL, 0.06 M). Purification by chromatography (Hex → Hex/AcOEt 10%) gave **4i** (109 mg, 87%) as a colourless oil; ^1H NMR (400 MHz, $CDCl_3$) δ 8.07 – 7.99 (m, 2H), 7.59 – 7.50 (m, 1H), 7.48 – 7.38 (m, 2H), 6.92 (d, *J* = 15.9 Hz, 1H), 5.71 (d, *J* = 16.0 Hz, 1H), 4.41 – 4.27 (m, 2H), 3.72 (s, 3H), 1.85 – 1.71 (m, 1H), 1.69 – 1.48 (m, 3H), 1.40 – 1.10 (m, 6H), 1.03 (s, 6H), 0.94 (d, *J* = 6.5 Hz, 3H); $^{13}C\{^1H\}$ NMR (101 MHz, $CDCl_3$) δ 167.7, 166.8, 158.8, 132.9, 130.6, 129.7, 128.5, 117.5, 63.6, 51.6, 42.6, 37.7, 36.9, 35.7, 30.0, 26.5, 26.5, 22.0, 19.7 ppm. HRMS (ESI) m/z: $[M+H]^+$: calculated for $C_{21}H_{31}O_4^+$ 347.2217, found 347.2216.

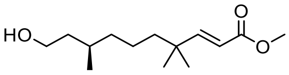
Methyl (R,E)-10-hydroxy-4,4,8-trimethyldec-2-enoate (**4j**). The title compound was prepared according to general procedure using the **3j** and **4j** mixture (87 mg, 0.36 mmol, 1.0 equiv.), triethylamine (36 mg, 50 µL, 0.36 mmol, 1.0 equiv.), thiophenol (79 mg, 73 µL, 0.72 mmol, 2.0 equiv.) and sodium periodate (231 mg, 1.08 mmol, 3.0 equiv.) in 10:1 MeOH/H_2O (6 mL, 0.06 M). Purification by chromatography (Hex → Hex/AcOEt 10%) gave **4j** (84 mg, 96%) as a colourless oil; ^1H NMR (400 MHz, $CDCl_3$) δ 6.91 (d, *J* = 16.0 Hz, 1H), 5.70 (d, *J* = 16.0 Hz, 1H), 3.72 (s, 3H), 3.71 – 3.57 (m, 2H), 1.63 – 1.47 (m, 2H), 1.40 – 1.04 (m, 7H), 1.03 (s, 6H), 0.86 (d, *J* = 6.6 Hz, 3H); $^{13}C\{^1H\}$ NMR (101 MHz, $CDCl_3$) δ 167.8, 158.9, 117.4, 61.2, 51.6, 42.6, 40.0, 37.8, 37.0, 29.5, 26.5, 26.4, 22.0, 19.7 ppm. HRMS (ESI) m/z: $[M+H]^+$: calculated for $C_{14}H_{27}O_3^+$ 243.1955, found 243.1956.

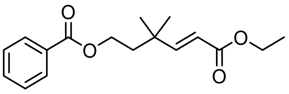
(E)-6-ethoxy-3,3-dimethyl-6-oxohex-4-en-1-yl benzoate (**4k**). The title compound was prepared according to general procedure using the **3k** and **4k** mixture (105 mg, 0.36 mmol, 1.0 equiv.), triethylamine (36 mg, 50 µL, 0.36 mmol, 1.0 equiv.), thiophenol (79 mg, 73 µL, 0.72 mmol, 2.0 equiv.) and sodium periodate (231 mg, 1.08 mmol, 3.0 equiv.) in 10:1 MeOH/H_2O (6 mL, 0.06 M). Purification by chromatography (Hex → Hex/AcOEt 10%) gave **4k** (101 mg, 97%) as a colourless oil; ^1H NMR (400 MHz, $CDCl_3$) δ 8.04 – 7.98 (m, 2H), 7.58 – 7.51 (m, 1H), 7.46 – 7.39 (m, 2H), 6.99 (d, *J* = 15.9 Hz, 1H), 5.77 (d, *J* = 15.9 Hz, 1H), 4.32 (t, *J* = 6.9 Hz, 2H), 4.14 (q, *J* = 7.1 Hz, 2H), 1.90 (t, *J* = 6.9 Hz, 2H), 1.26 (t, *J* = 7.2 Hz, 3H), 1.17 (s, 6H); $^{13}C\{^1H\}$ NMR (101 MHz, $CDCl_3$) δ 167.0, 166.7, 157.0, 133.0, 130.3, 129.7, 128.5, 118.4, 62.0, 60.4, 40.6, 36.0, 26.8, 14.4 ppm. HRMS (ESI) m/z: $[M+H]^+$: calculated for $C_{17}H_{23}O_4^+$ 291.1591, found 291.1594.

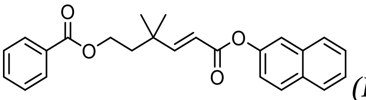
(E)-3,3-dimethyl-6-(naphthalen-2-yloxy)-6-oxohex-4-en-1-yl benzoate (**4l**). The title compound was prepared according to general procedure using the **3l** and **4l** mixture (140 mg, 0.36 mmol, 1.0 equiv.), triethylamine (36 mg, 50 µL, 0.36 mmol, 1.0 equiv.), thiophenol (79 mg, 73 µL, 0.72 mmol, 2.0 equiv.) and sodium periodate (231 mg, 1.08 mmol, 3.0 equiv.) in 10:1 MeOH/H_2O (6 mL, 0.06 M). Purification by chromatography (Hex → Hex/AcOEt 10%) gave **4l** (136 mg, 97%) as a colourless oil; ^1H NMR (400 MHz, $CDCl_3$) δ 8.04 (dd, *J* = 8.4, 1.4 Hz, 2H), 7.83 (d, *J* = 8.8 Hz, 2H), 7.81 – 7.74 (m, 1H), 7.56 – 7.51 (m, 1H), 7.50 (d, *J* = 1.8 Hz, 1H), 7.47 (ddd, *J* = 7.9, 5.7, 1.6 Hz, 2H), 7.46 – 7.37 (m, 2H), 7.26 (d, *J* = 15.9 Hz, 1H), 7.18 (dd, *J* = 8.9, 2.3 Hz, 1H), 6.03 (d, *J* = 16.0 Hz, 1H), 4.38 (t, *J* = 6.7 Hz, 2H), 1.97 (t, *J* = 6.8 Hz, 2H), 1.25 (s, 6H); $^{13}C\{^1H\}$ NMR (101 MHz, $CDCl_3$) δ 166.7, 165.5, 159.5, 148.5, 133.9, 133.1,

131.6, 130.2, 129.7, 129.4, 128.6, 127.9, 127.8, 126.6, 125.8, 121.4, 118.7, 117.6, 62.0, 40.7, 36.4, 26.8 ppm. HRMS (ESI) m/z: $[M+H]^+$: calculated for $C_{25}H_{25}O_4^+$ 389.1747, found 389.1749.

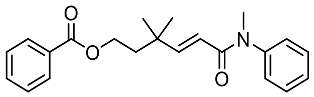

(E)-3,3-dimethyl-6-(methyl(phenyl)amino)-6-oxohex-4-en-1-yl benzoate (**4m**). The title compound was prepared according to general procedure using the **3m** and **4m** mixture (127 mg, 0.36 mmol, 1.0 equiv.), triethylamine (36 mg, 50 μL, 0.36 mmol, 1.0 equiv.), thiophenol (79 mg, 73 μL, 0.72 mmol, 2.0 equiv.) and sodium periodate (231 mg, 1.08 mmol, 3.0 equiv.) in 10:1 MeOH/H_2O (6 mL, 0.06 M). Purification by chromatography (Hex → Hex/AcOEt 25%) gave **4m** (121 mg, 96%) as a yellow oil; ^{1}H NMR (400 MHz, $CDCl_3$) δ 8.00 (dd, J = 8.3, 1.4 Hz, 2H), 7.58 – 7.52 (m, 1H), 7.44 (t, J = 7.6 Hz, 2H), 7.42 – 7.33 (m, 2H), 7.31 – 7.25 (m, 1H), 7.15 – 7.08 (m, 2H), 6.91 (d, J = 15.5 Hz, 1H), 5.66 (d, J = 15.5 Hz, 1H), 4.21 (t, J = 7.0 Hz, 2H), 3.33 (s, 3H), 1.80 (t, J = 7.0 Hz, 2H), 1.00 (s, 6H); ^{13}C{^{1}H} NMR (101 MHz, $CDCl_3$) δ 166.6, 166.4, 153.4, 143.8, 133.0, 130.4, 129.7, 129.6, 128.5, 127.5, 127.3, 118.6, 62.2, 40.4, 37.5, 35.8, 26.9 ppm. HRMS (ESI) m/z: $[M+H]^+$: calculated for $C_{22}H_{26}NO_3^+$ 352.1907, found 352.1908.

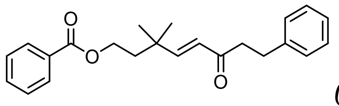

(E)-3,3-dimethyl-6-oxo-8-phenyloct-4-en-1-yl benzoate (**4o**). The title compound was prepared according to general procedure using the **3o** and **4o** mixture (126 mg, 0.36 mmol, 1.0 equiv.), triethylamine (36 mg, 50 μL, 0.36 mmol, 1.0 equiv.), thiophenol (79 mg, 73 μL, 0.72 mmol, 2.0 equiv.) and sodium periodate (231 mg, 1.08 mmol, 3.0 equiv.) in 10:1 MeOH/H_2O (6 mL, 0.06 M). Purification by chromatography (Hex → Hex/AcOEt 25%) gave **4o** (122 mg, 97%) as a colourless oil; ^{1}H NMR (400 MHz, $CDCl_3$) δ 8.03 – 7.95 (m, 2H), 7.58 – 7.51 (m, 1H), 7.46 – 7.37 (m, 2H), 7.31 – 7.22 (m, 2H), 7.22 – 7.12 (m, 3H), 6.81 (d, J = 16.2 Hz, 1H), 6.04 (d, J = 16.2 Hz, 1H), 4.30 (t, J = 6.9 Hz, 2H), 2.88 (ddd, J = 8.3, 6.5, 1.8 Hz, 2H), 2.79 (ddd, J = 8.7, 6.7, 1.8 Hz, 2H), 1.88 (t, J = 6.9 Hz, 2H), 1.15 (s, 6H); ^{13}C{^{1}H} NMR (101 MHz, $CDCl_3$) δ 199.7, 166.6, 155.2, 141.3, 133.1, 130.2, 129.7, 128.6, 128.5, 128.5, 126.7, 126.2, 62.0, 42.2, 40.6, 36.0, 30.1, 26.8 ppm. HRMS (ESI) m/z: $[M+H]^+$: calculated for $C_{23}H_{27}O_3^+$ 351.1955, found 351.1952.

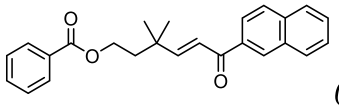

(E)-3,3-dimethyl-6-(naphthalen-2-yl)-6-oxohex-4-en-1-yl benzoate (**4p**). The title compound was prepared according to general procedure using the mixture of **3p** and **4p** (134 mg, 0.36 mmol, 1.0 equiv.), triethylamine (36 mg, 50 μL, 0.36 mmol, 1.0 equiv.), thiophenol (79 mg, 73 μL, 0.72 mmol, 2.0 equiv.) and sodium periodate (231 mg, 1.08 mmol, 3.0 equiv.) in 10:1 MeOH/H_2O (6 mL, 0.06 M). Purification by chromatography (Hex → Hex/AcOEt 25%) gave **4p** (127 mg, 95%) as a colourless oil; ^{1}H NMR (400 MHz, $CDCl_3$) δ 8.45 – 8.40 (m, 1H), 8.04 – 7.94 (m, 4H), 7.93 – 7.84 (m, 2H), 7.63 – 7.57 (m, 1H), 7.59 – 7.51 (m, 1H), 7.52 – 7.46 (m, 1H), 7.37 (t, J = 7.7 Hz, 2H), 7.17 (d, J = 15.7 Hz, 1H), 7.02 (d, J = 15.7 Hz, 1H), 4.41 (t, J = 6.9 Hz, 2H), 2.00 (t, J = 7.0 Hz, 2H), 1.29 (s, 6H); ^{13}C{^{1}H} NMR (101 MHz, $CDCl_3$) δ 190.7, 166.7, 157.4, 135.5, 135.5, 133.0, 132.6, 130.2, 130.1, 129.7, 129.7, 128.6, 128.5, 128.4, 127.9, 126.8, 124.6, 122.2, 62.1, 40.6, 36.5, 27.0 ppm. HRMS (ESI) m/z: $[M+H]^+$: calculated for $C_{25}H_{25}O_3^+$ 373.1798, found 373.1798.

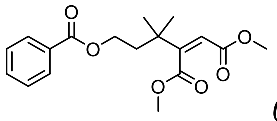

(E)-dimethyl 2-(4-(benzoyloxy)-2-methylbutan-2-yl)maleate (**4q**). The title compound was prepared according to general procedure using the **3q** and **4q** mixture (120 mg, 0.36 mmol, 1.0 equiv.), triethylamine (36 mg, 50 μL, 0.36 mmol, 1.0 equiv.), thiophenol (79 mg, 73 μL, 0.72 mmol, 2.0 equiv.) and sodium periodate (231 mg, 1.08 mmol, 3.0 equiv.) in 10:1 MeOH/H_2O (6 mL, 0.06 M). Purification by chromatography (Hex → Hex/AcOEt 10%) gave **4q** (114 mg, 95%) as a colourless oil; ^{1}H NMR (400 MHz, $CDCl_3$) δ 8.04 – 7.98 (m, 2H), 7.55 (ddt, *J* = 8.0, 6.9, 1.4 Hz, 1H), 7.46 – 7.39 (m, 2H), 5.88 (s, 1H), 4.36 (t, *J* = 7.0 Hz, 2H), 3.84 (s, 3H), 3.70 (s, 3H), 2.00 (t, *J* = 7.0 Hz, 2H), 1.25 (s, 6H); $^{13}C\{^1H\}$ NMR (101 MHz, $CDCl_3$) δ 168.8, 166.6, 165.5, 158.5, 133.0, 130.3, 129.7, 128.5, 117.8, 62.0, 52.4, 52.0, 39.4, 38.0, 27.1 ppm. HRMS (ESI) m/z: $[M+H]^+$: calculated for $C_{18}H_{23}O_6^+$ 335.1489, found 335.1488.

COPIES OF NMR SPECTRA

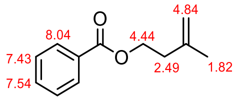

1a

san-1988-2023.1.fid 1H 400 MHz
Equip: B400Q / N.Inv: 1035091
N.Reg: 1988/2023
Usuari: san / Mostra: LRG322CH
Nom: LAURA RODRIGUEZ GONZALEZ
Data: 01/04/2023 04:51:58 h./ Ope.: servei Unitat RMN
Experiment: A_1H-zg30 Solvent: CDCl3 Operator: M ANTONIA MOLINS

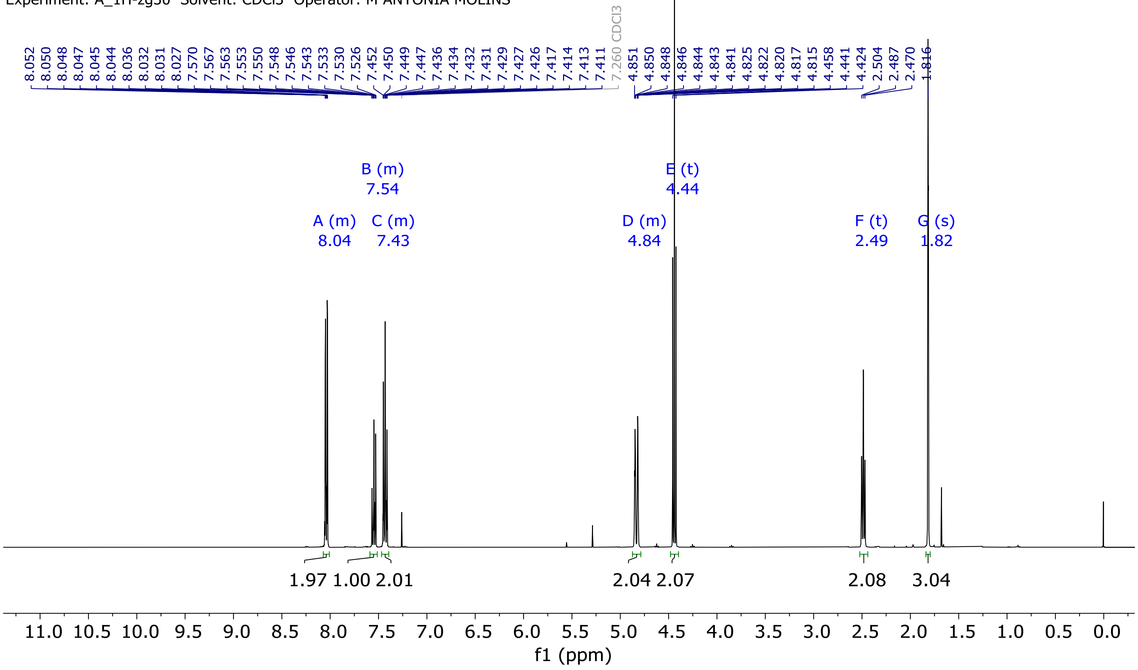

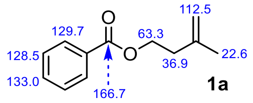

1a

san-1988-2023.2.fid 13C{1H} 101 MHz
Equip: B400Q / N.Inv: 1035091
N.Reg: 1988/2023
Usuari: san / Mostra: LRG322CH
Nom: LAURA RODRIGUEZ GONZALEZ
Data: 01/04/2023 04:51:58 h./ Ope.: servei Unitat RMN
Experiment: A_13C-zgpg30 Solvent: CDCl3 Operator: M ANTONIA MOLINS

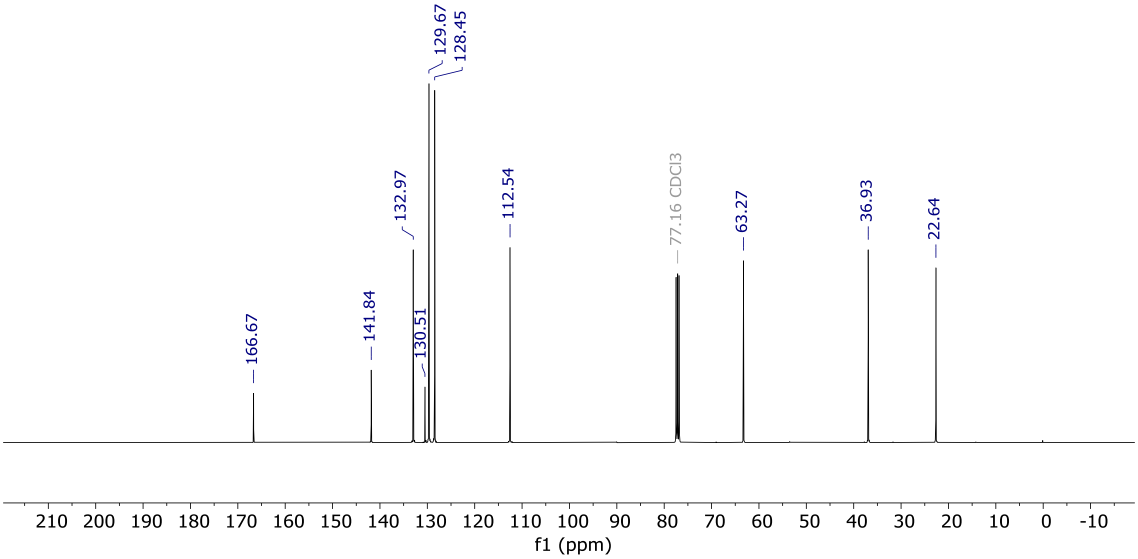

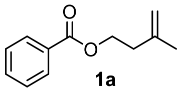

1a

2D-COSY

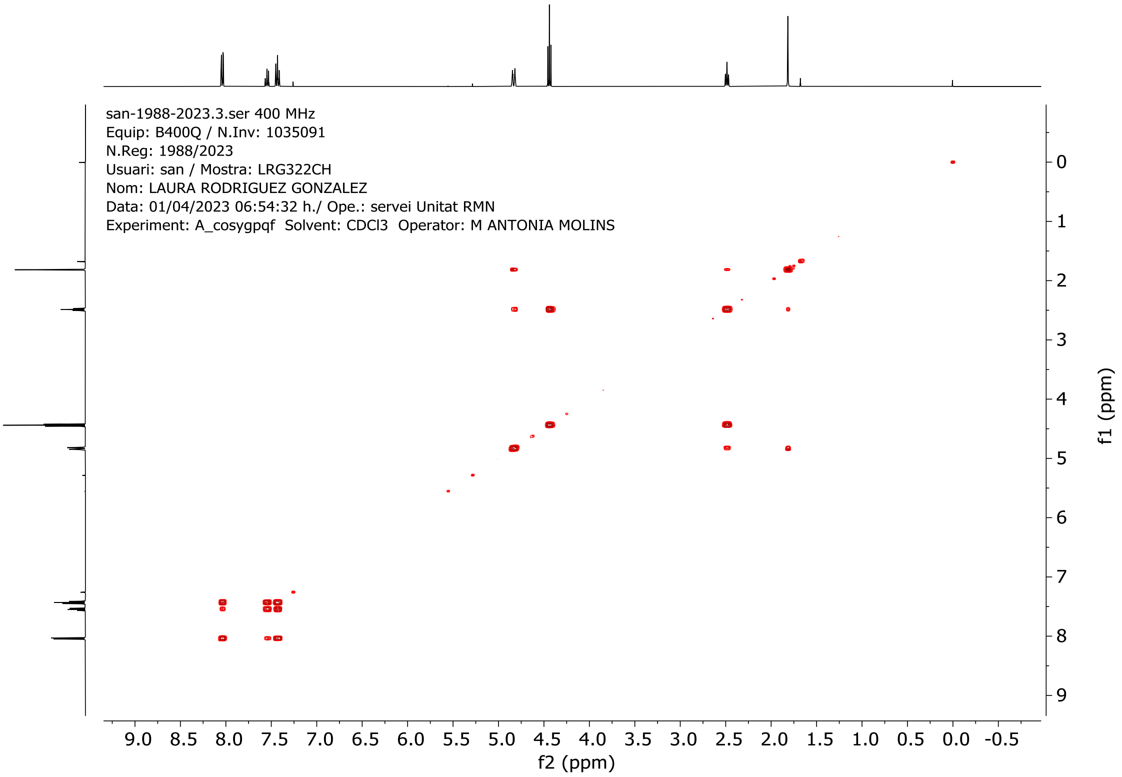

2D-HSQC

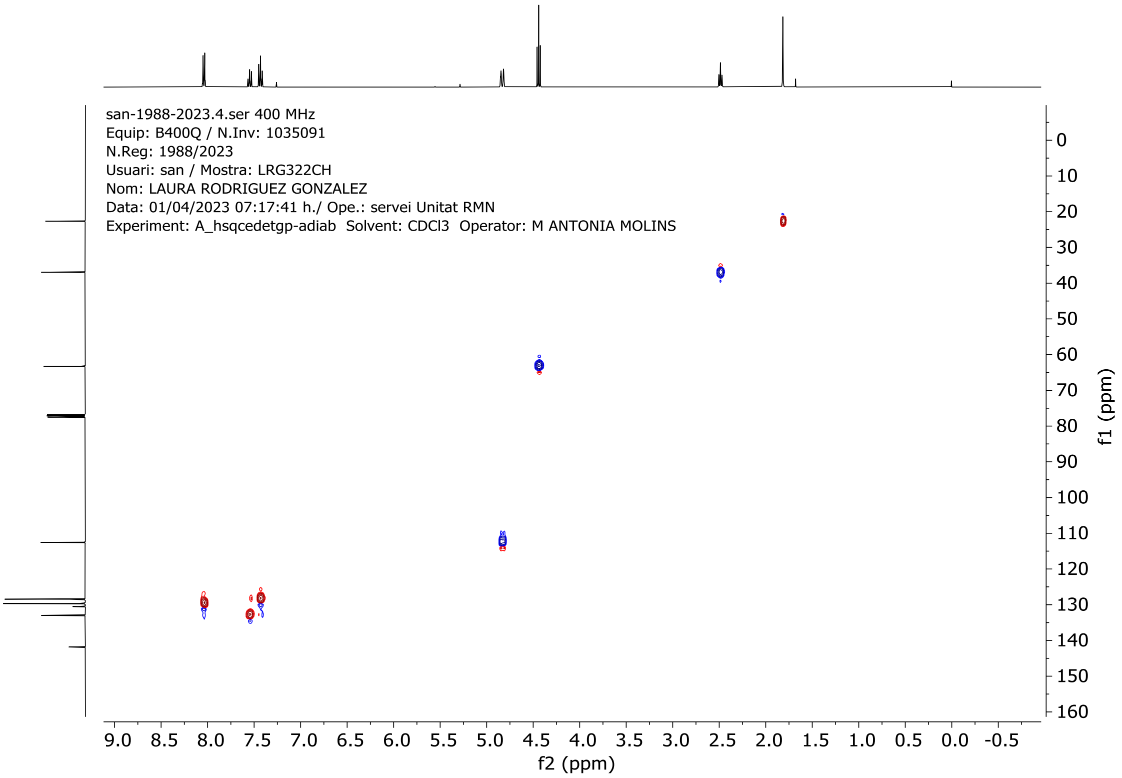

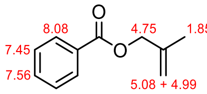

1b

LRG339COL.1.fid 1H 400 MHz
Equip: B400F / N.Inv: 1037597
N.Reg: 23030307
Usuari: san / Mostra: LRG339COL
Nom: LAURA RODRIGUEZ GONZALEZ
Data: 07/03/2023 15:53:58 h./ Ope.: AUTOSERVEI
Experiment: A-H1-zg30 Solvent: CDCl3

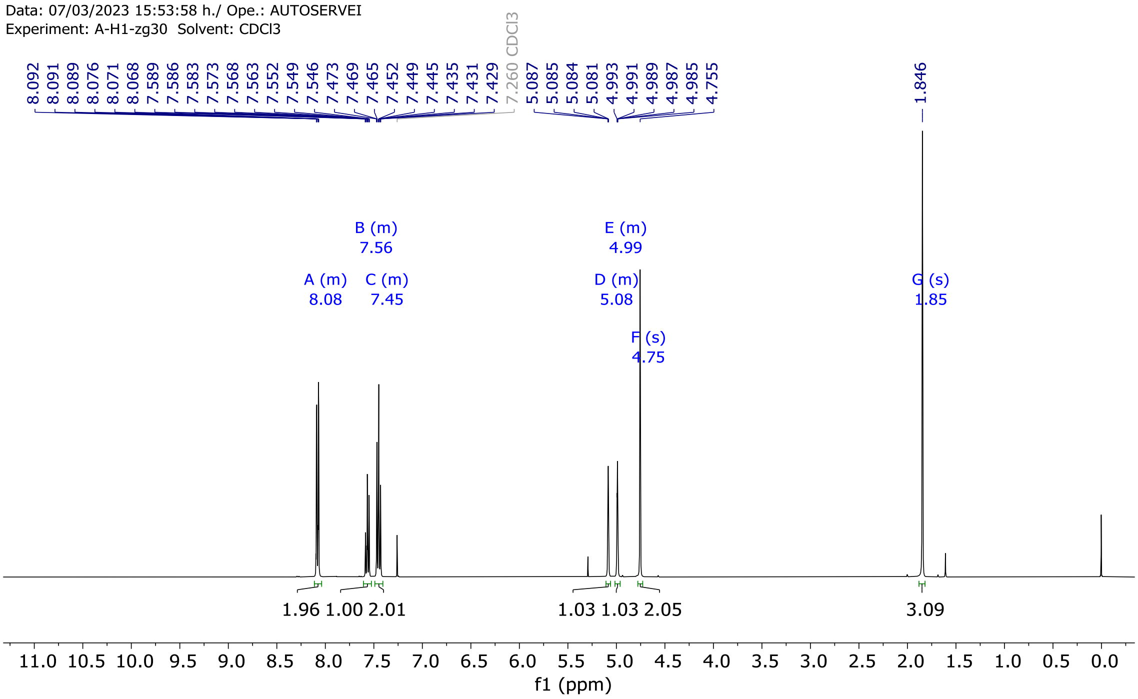

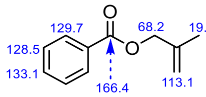

1b

san-1990-2023.2.fid 13C{1H} 101 MHz
Equip: B400Q / N.Inv: 1035091
N.Reg: 1990/2023
Usuari: san / Mostra: LRG339CH
Nom: LAURA RODRIGUEZ GONZALEZ
Data: 01/04/2023 08:02:53 h./ Ope.: servei Unitat RMN
Experiment: A_13C-zgpg30 Solvent: CDCl3 Operator: M ANTONIA MOLINS

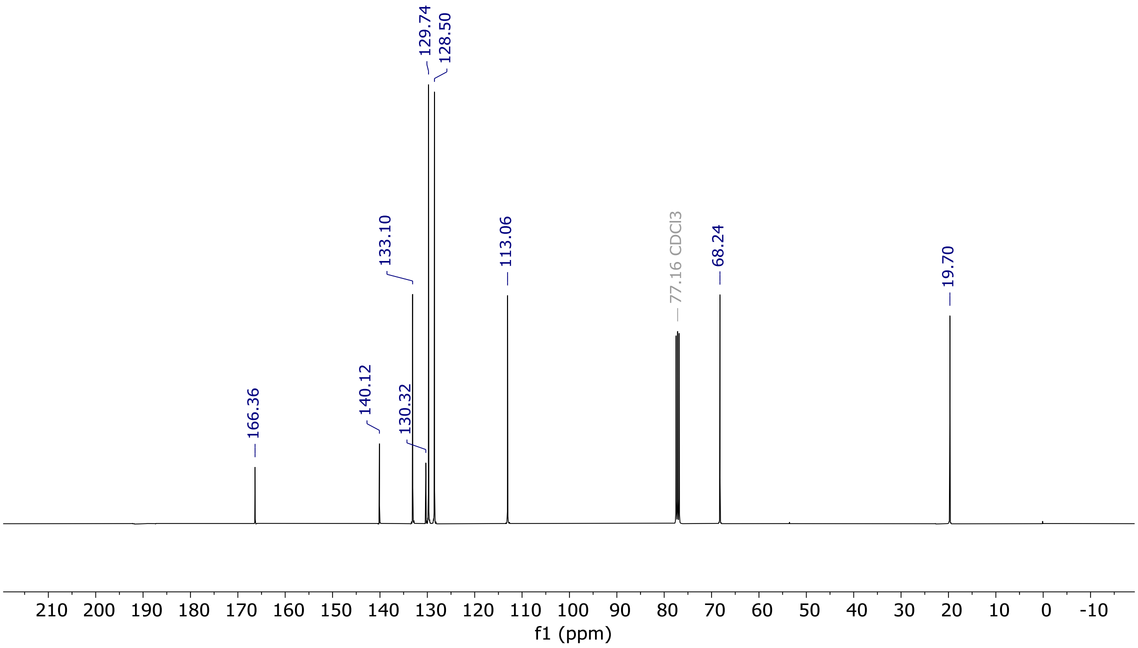

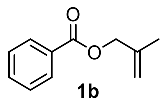

1b

2D-COSY

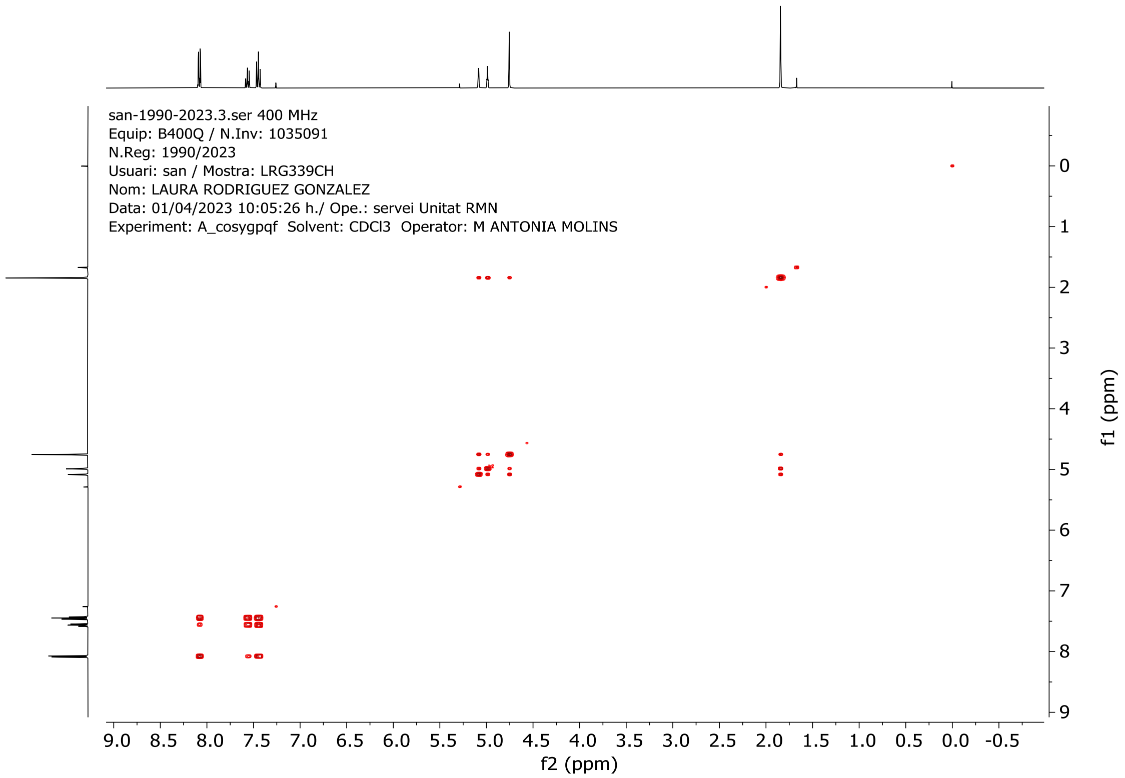

2D-HSQC

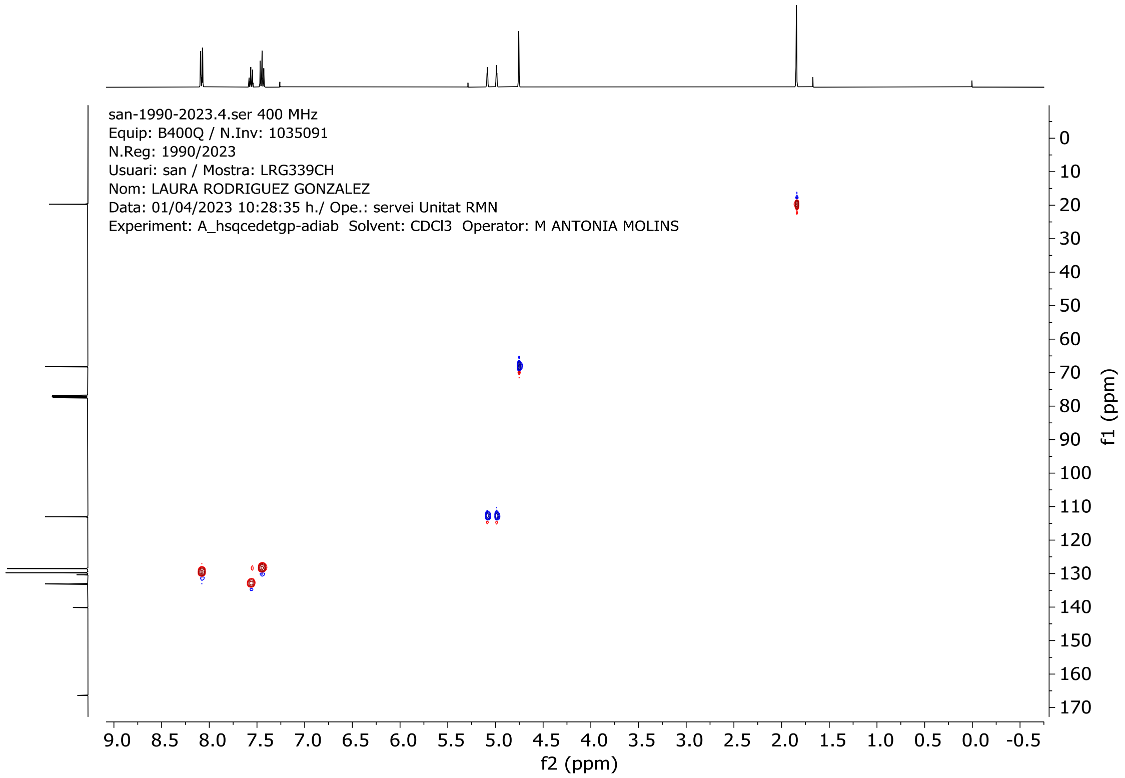

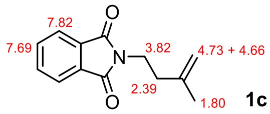

1c

LRG357CH.1.fid 1H 400 MHz
Equip: B400F / N.Inv: 1037597
N.Reg: 23030893
Usuari: san / Mostra: LRG357CH
Nom: LAURA RODRIGUEZ GONZALEZ
Data: 24/03/2023 12:47:27 h./ Ope.: AUTOSERVEI
Experiment: A-H1-zg30 Solvent: CDCl3

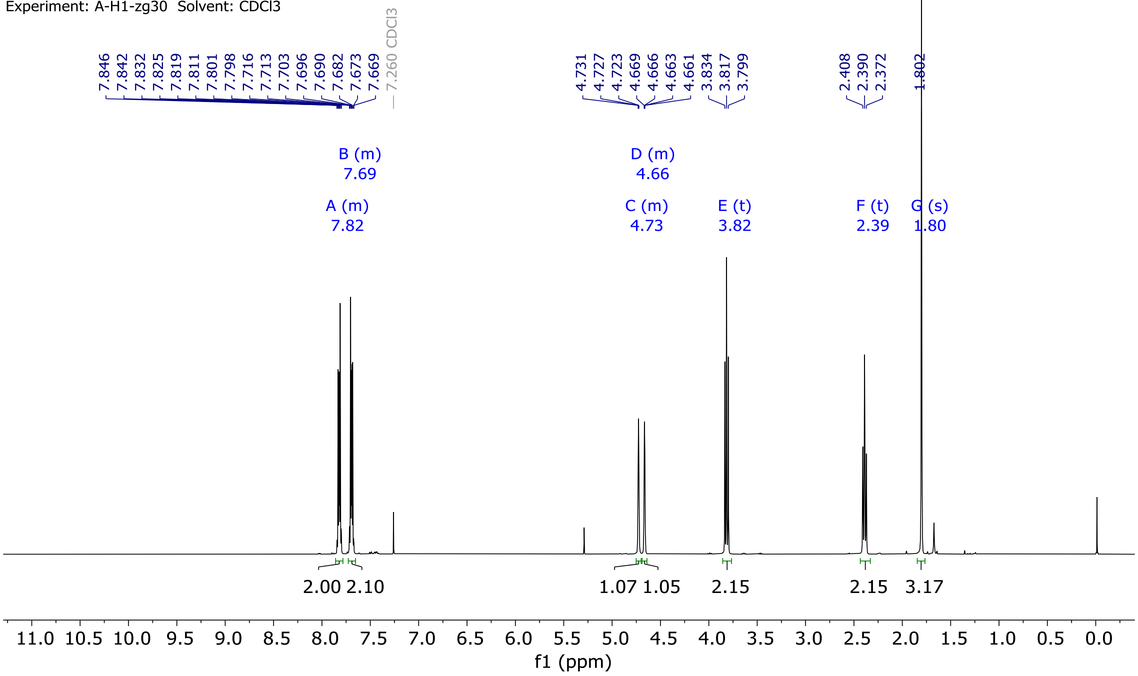

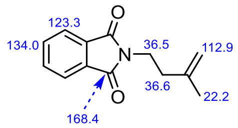

1c

LRG357CH.2.fid 13C{1H} 101 MHz
Equip: B400F / N.Inv: 1037597
N.Reg: 23030893
Usuari: san / Mostra: LRG357CH
Nom: LAURA RODRIGUEZ GONZALEZ
Data: 25/03/2023 12:32:53 h./ Ope.: AUTOSERVEI
Experiment: A-C13-zgpg30 Solvent: CDCl3

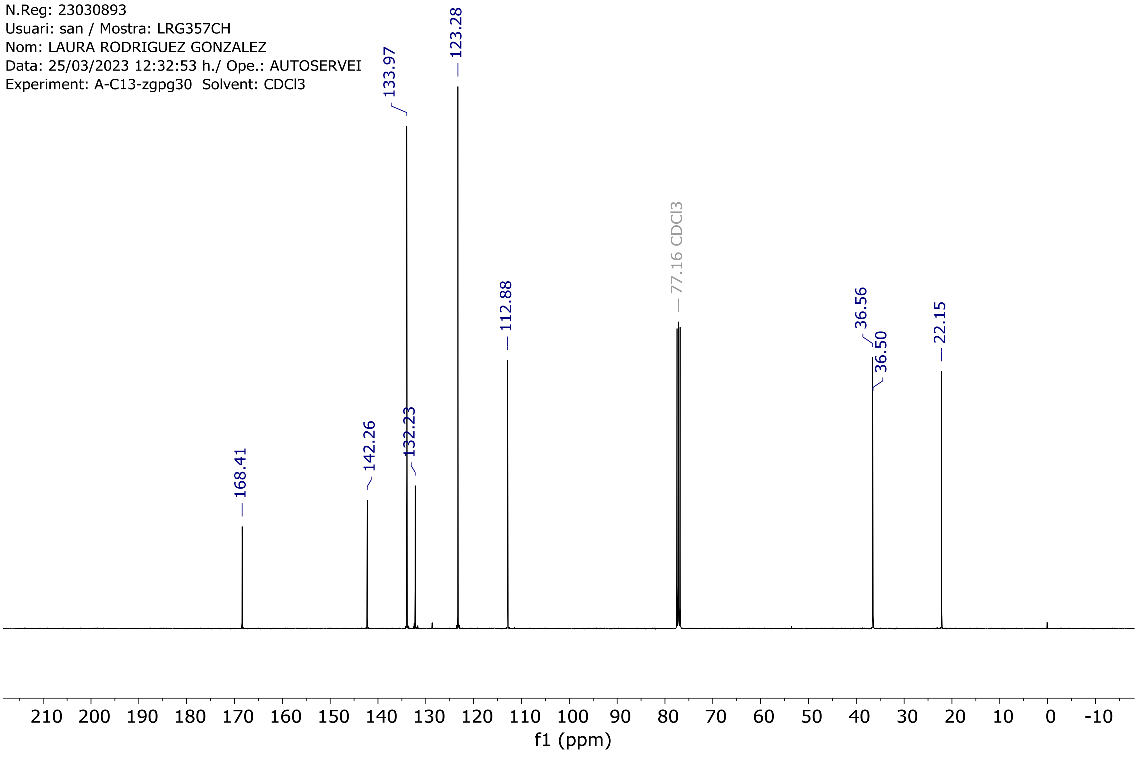

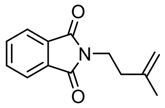

1c

2D-COSY

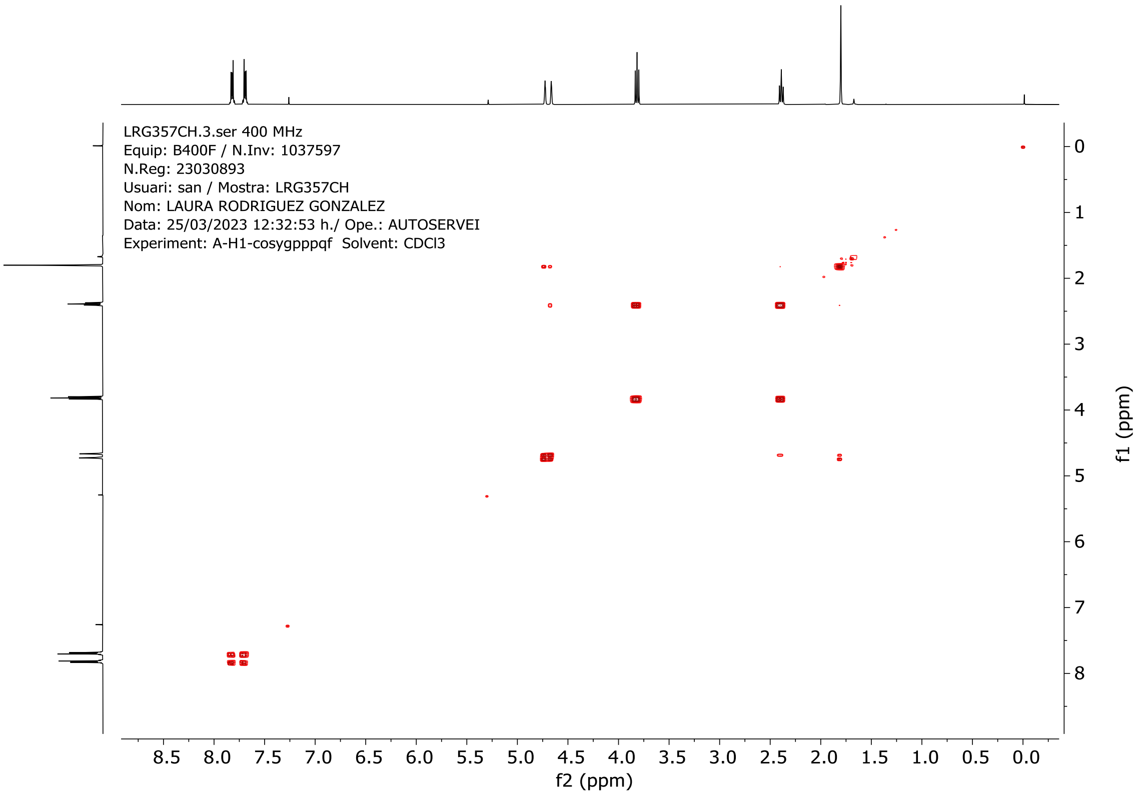

2D-HSQC

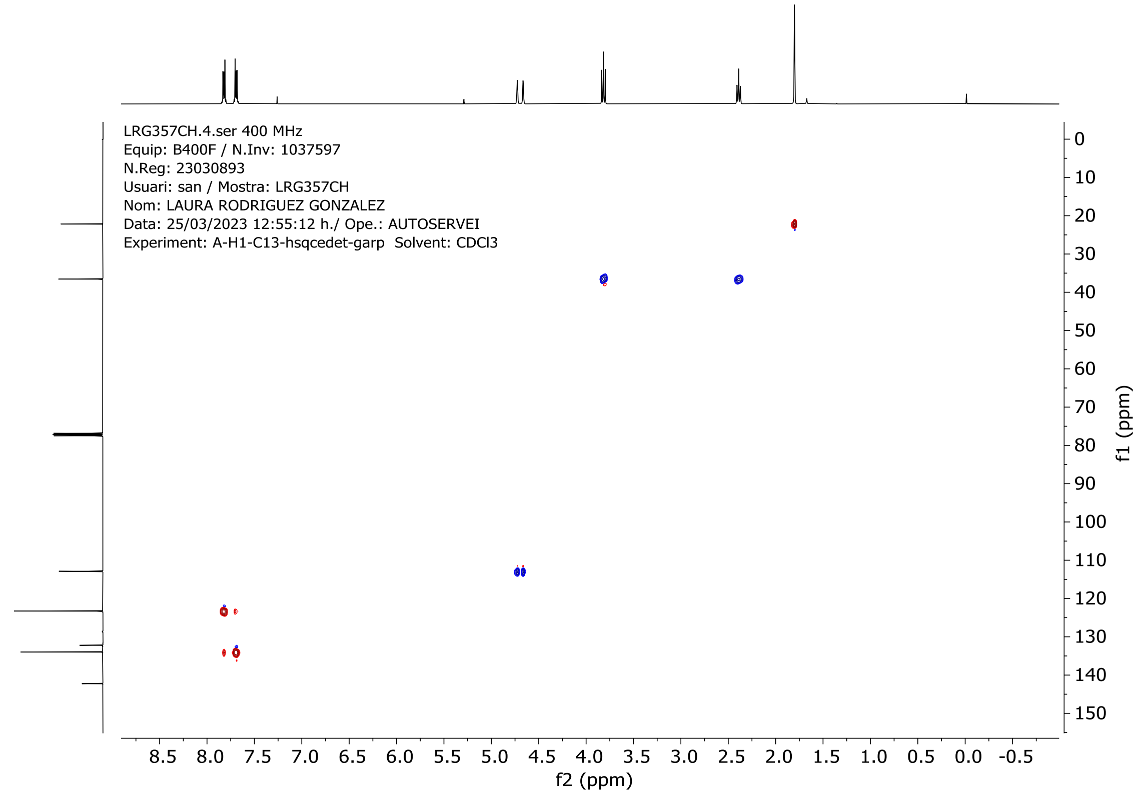

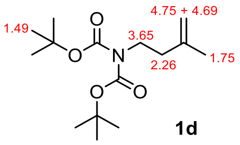

1d

23040011_B400FA_03042023_LRG362COL3T17T19.1.fid 1H 400 MHz
Equip: B400F / N.Inv: 1037597
N.Reg: 23040011
Usuari: san / Mostra: LRG362COL3T17T19
Nom: LAURA RODRIGUEZ GONZALEZ
Data: 03/04/2023 11:24:55 h./ Ope.: AUTOSERVEI
Experiment: A-H1-zg30 Solvent: CDCl3

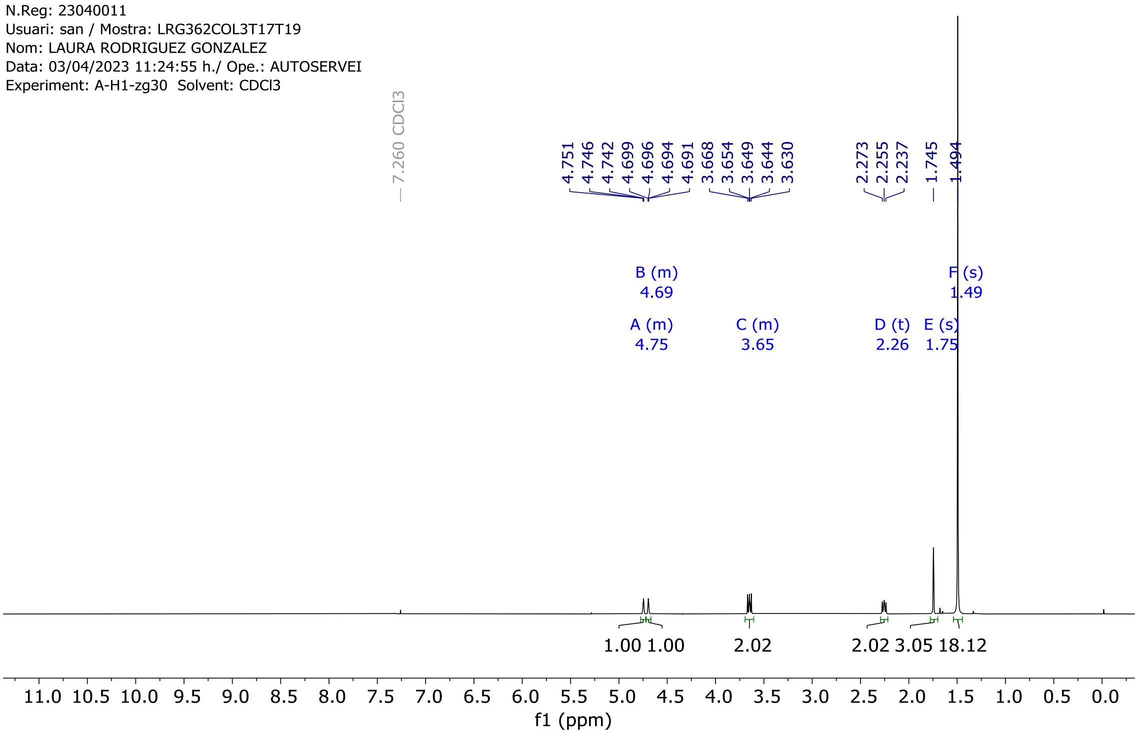

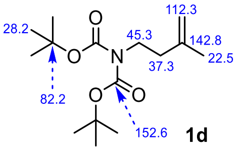

1d

san-2011-2023.2.fid 13C{1H} 101 MHz
Equip: B400Q / N.Inv: 1035091
N.Reg: 2011/2023
Usuari: san / Mostra: LRG362CH
Nom: LAURA RODRIGUEZ GONZALEZ
Data: 03/04/2023 23:19:06 h./ Ope.: servei Unitat RMN
Experiment: A_13C-zgpg30 Solvent: CDCl3 Operator: Victoria Munoz Torrero

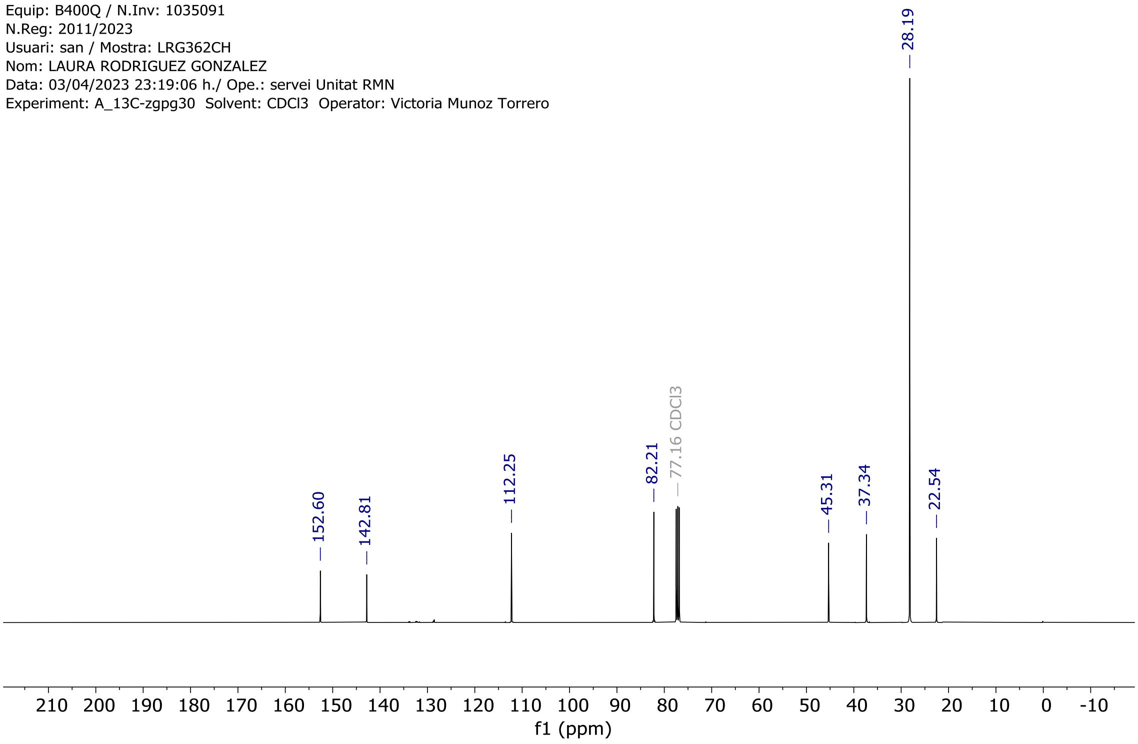

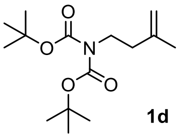

1d

2D-COSY

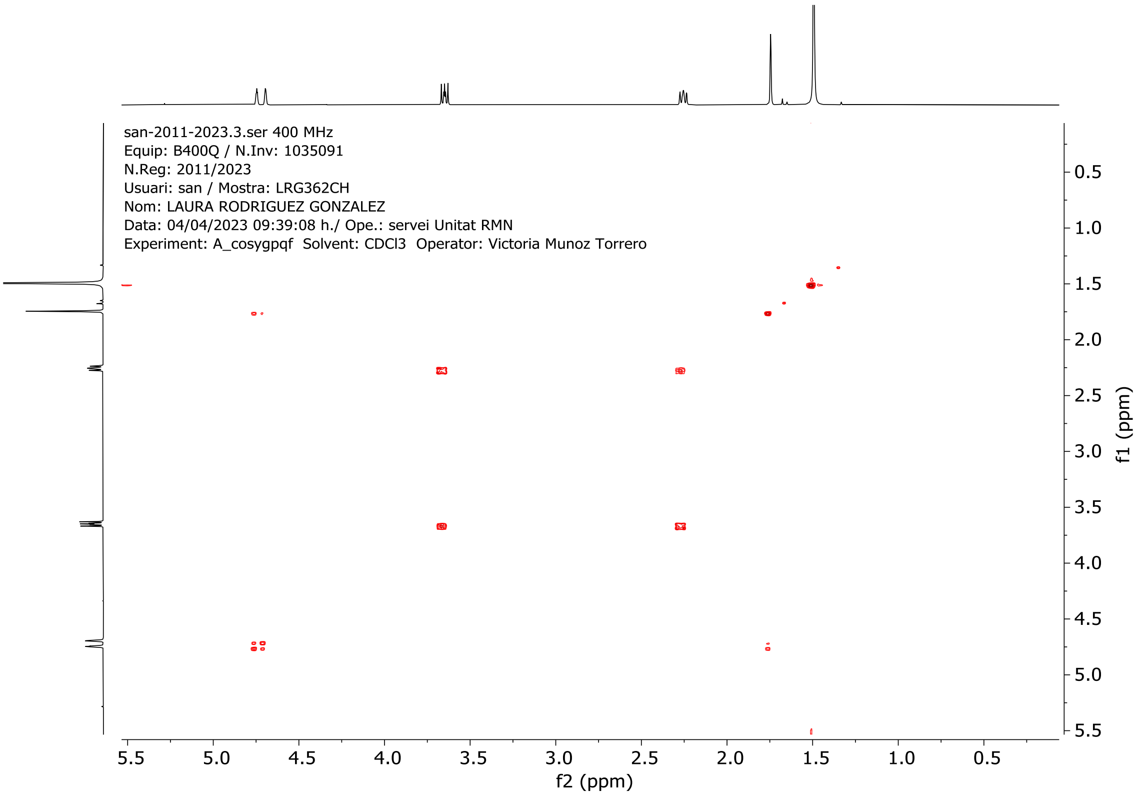

2D-HSQC

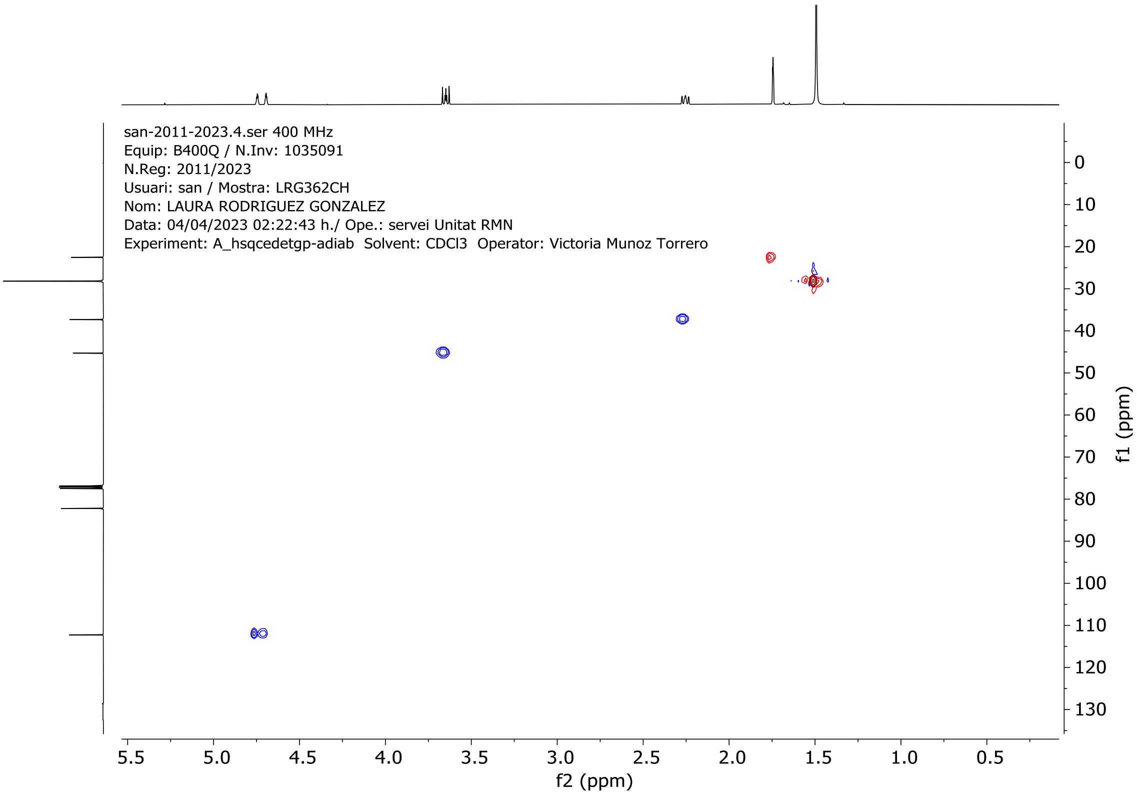

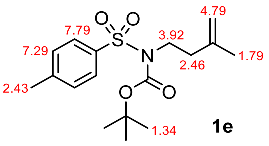

1e

23040598_B400FA_26042023_LRG375COLT16T20.1.fid 1H 400 MHz
Equip: B400F / N.Inv: 1037597
N.Reg: 23040598
Usuari: san / Mostra: LRG375COLT16T20
Nom: LAURA RODRIGUEZ GONZALEZ
Data: 25/04/2023 16:35:45 h./ Ope.: AUTOSERVEI
Experiment: A-H1-zg30 Solvent: CDCl3

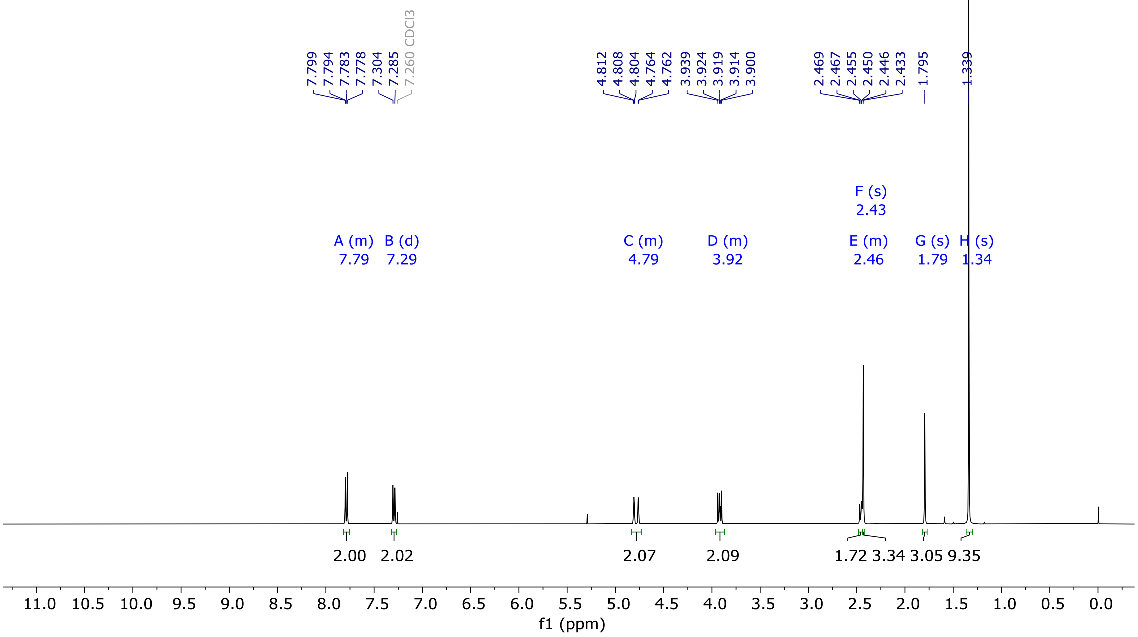

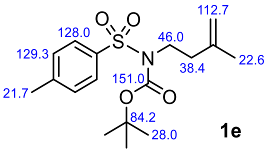

1e

23040598_B400FA_26042023_LRG375COLT16T20.2.fid 13C{1H} 101 MHz
Equip: B400F / N.Inv: 1037597
N.Reg: 23040598
Usuari: san / Mostra: LRG375COLT16T20
Nom: LAURA RODRIGUEZ GONZALEZ
Data: 26/04/2023 07:27:52 h./ Ope.: AUTOSERVEI
Experiment: A-C13-zgpg30 Solvent: CDCl3

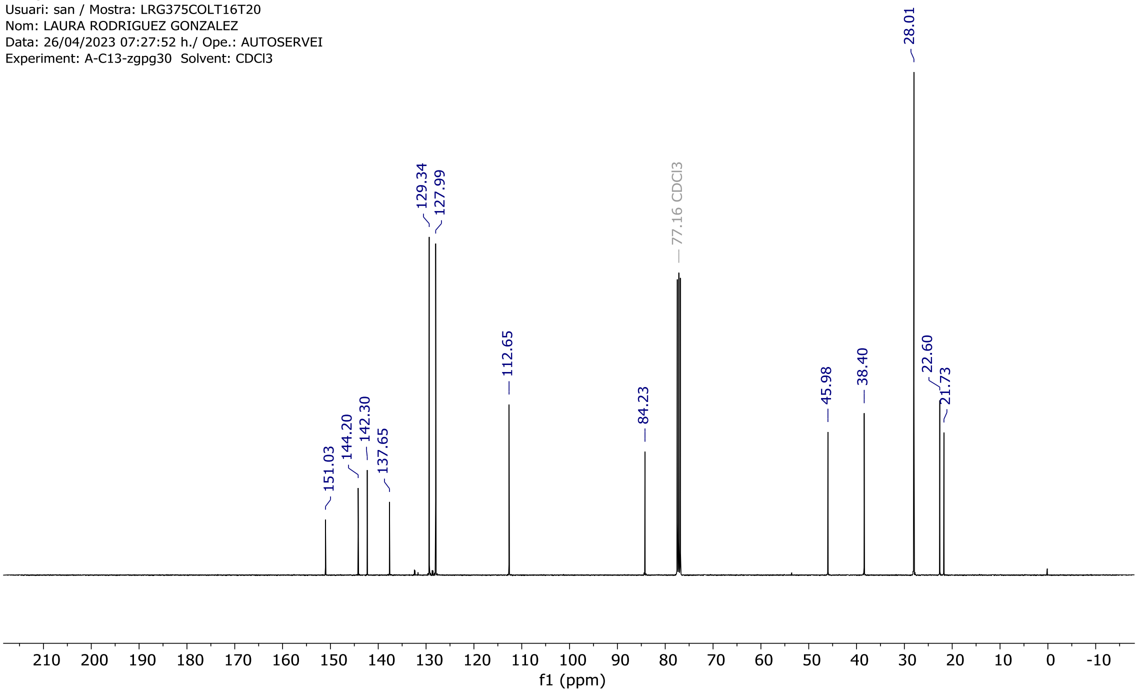

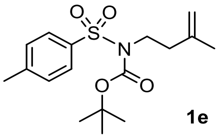

1e

2D-COSY

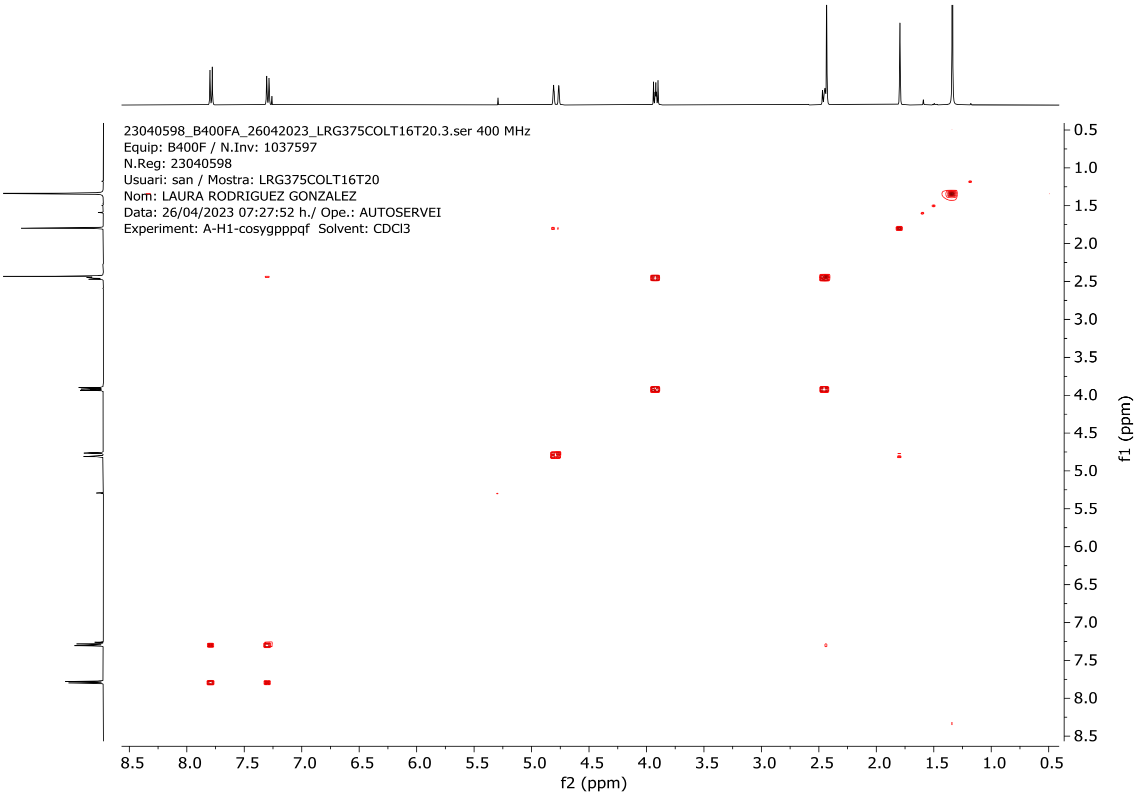

2D-HSQC

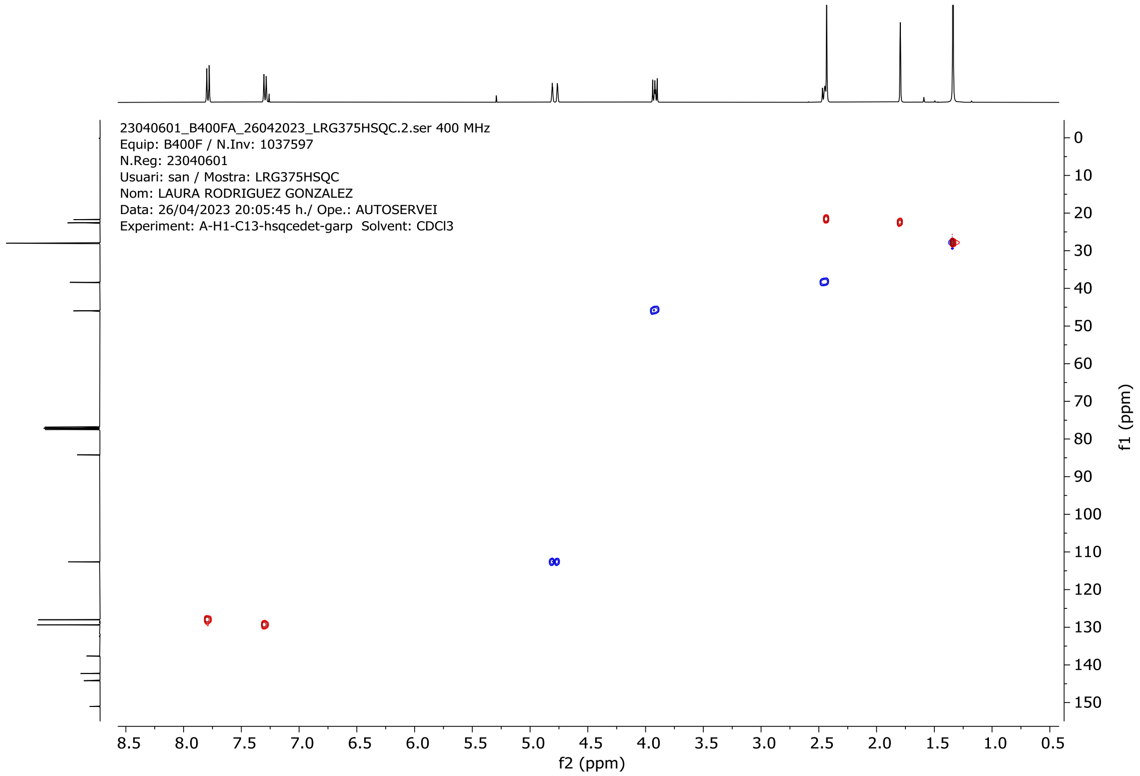

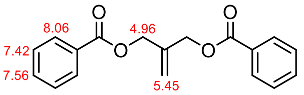

1f

LRG370T13T17.1.fid 1H 400 MHz
Equip: B400F / N.Inv: 1037597
N.Reg: 23040346
Usuari: san / Mostra: LRG370T13T17
Nom: LAURA RODRIGUEZ GONZALEZ
Data: 18/04/2023 14:47:36 h./ Ope.: AUTOSERVEI
Experiment: A-H1-zg30 Solvent: CDCl3

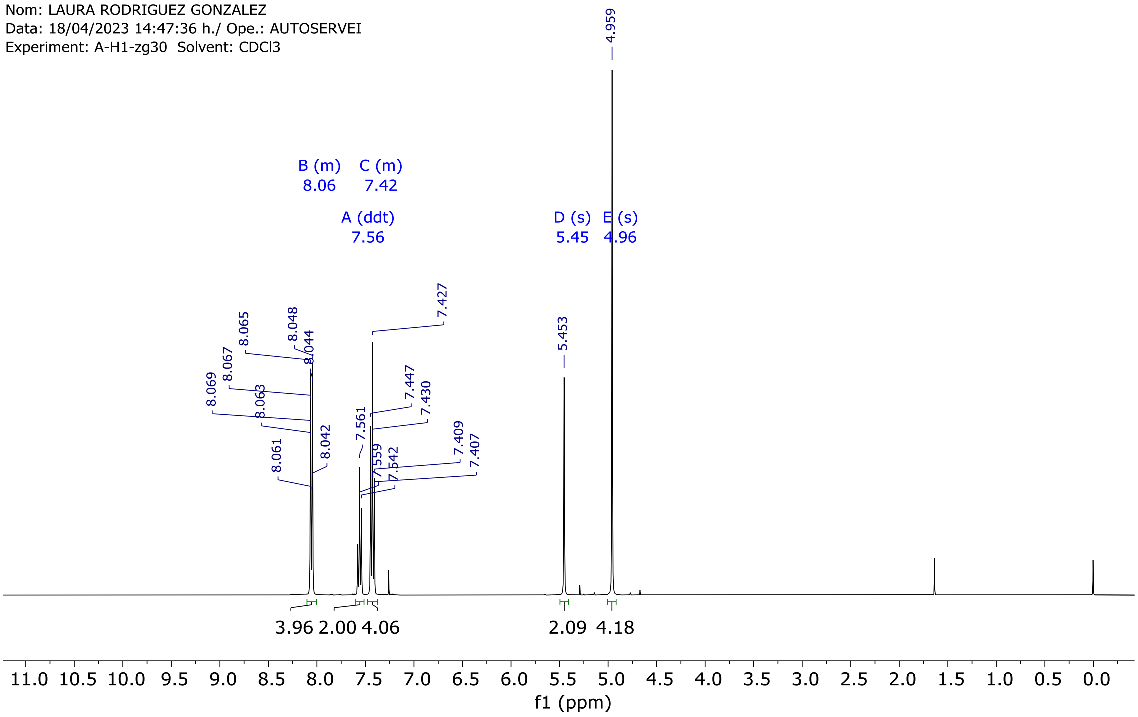

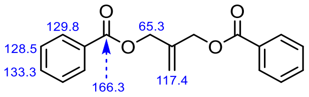

1f

san-2230-2023.4.fid 13C{1H} 101 MHz
Equip: B400Q / N.Inv: 1035091
N.Reg: 2230/2023
Usuari: san / Mostra: LRG370CH
Nom: LAURA RODRIGUEZ GONZALEZ
Data: 20/04/2023 02:40:18 h./ Ope.: servei Unitat RMN
Experiment: A_13C-zgpg30 Solvent: CDCl3 Operator: M ANTONIA MOLINS

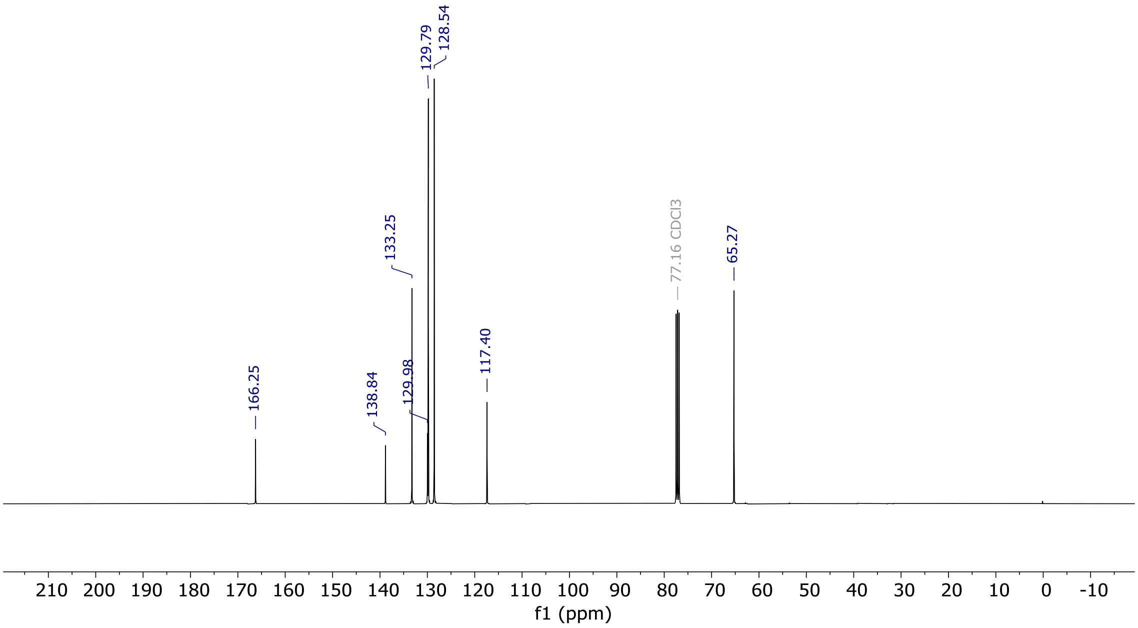

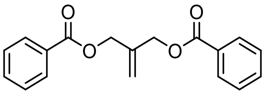

1f

2D-COSY

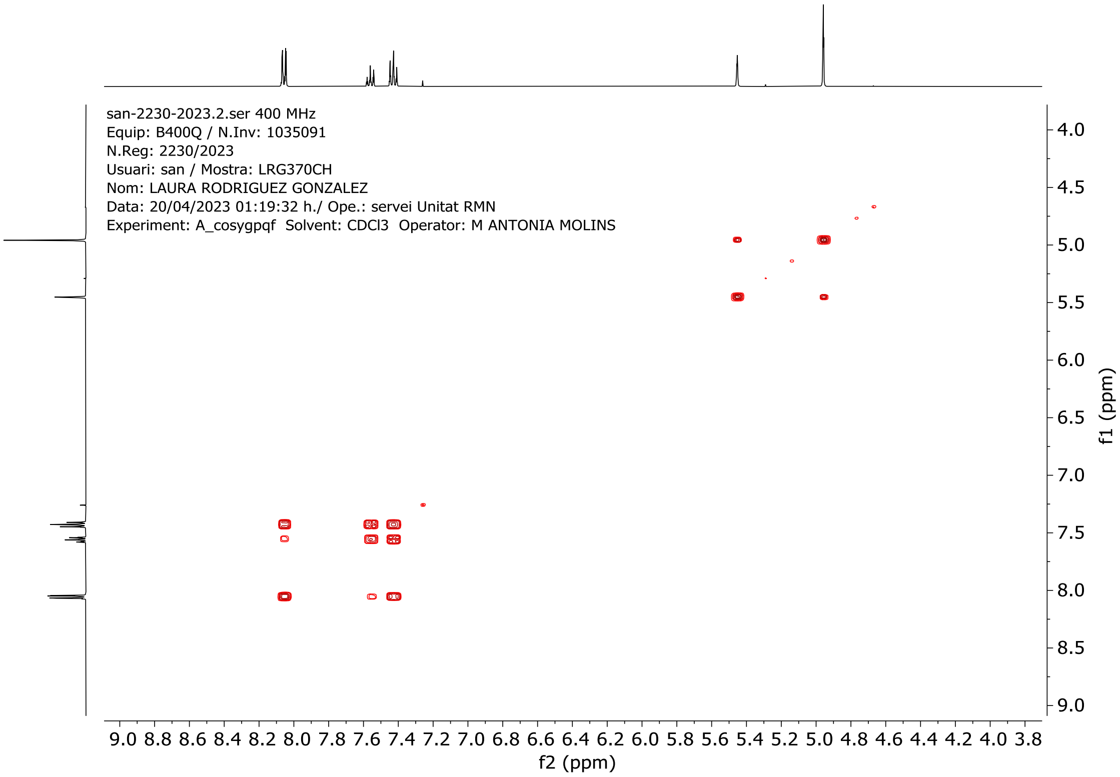

2D-HSQC

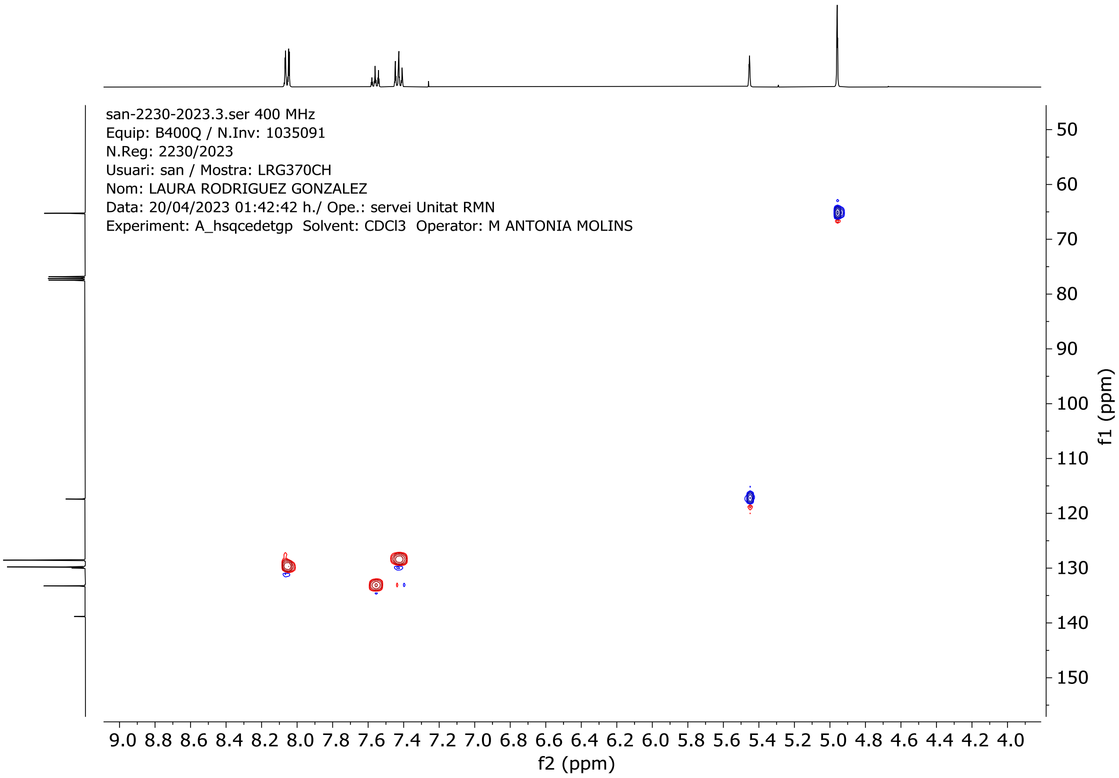

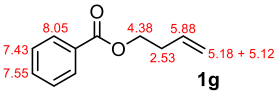

1g

san-1991-2023.1.fid 1H 400 MHz
Equip: B400Q / N.Inv: 1035091
N.Reg: 1991/2023
Usuari: san / Mostra: LRGJPB525CH
Nom: LAURA RODRIGUEZ GONZALEZ
Data: 01/04/2023 11:14:09 h./ Ope.: servei Unitat RMN
Experiment: A_1H-zg30 Solvent: CDCl3 Operator: M ANTONIA MOLINS

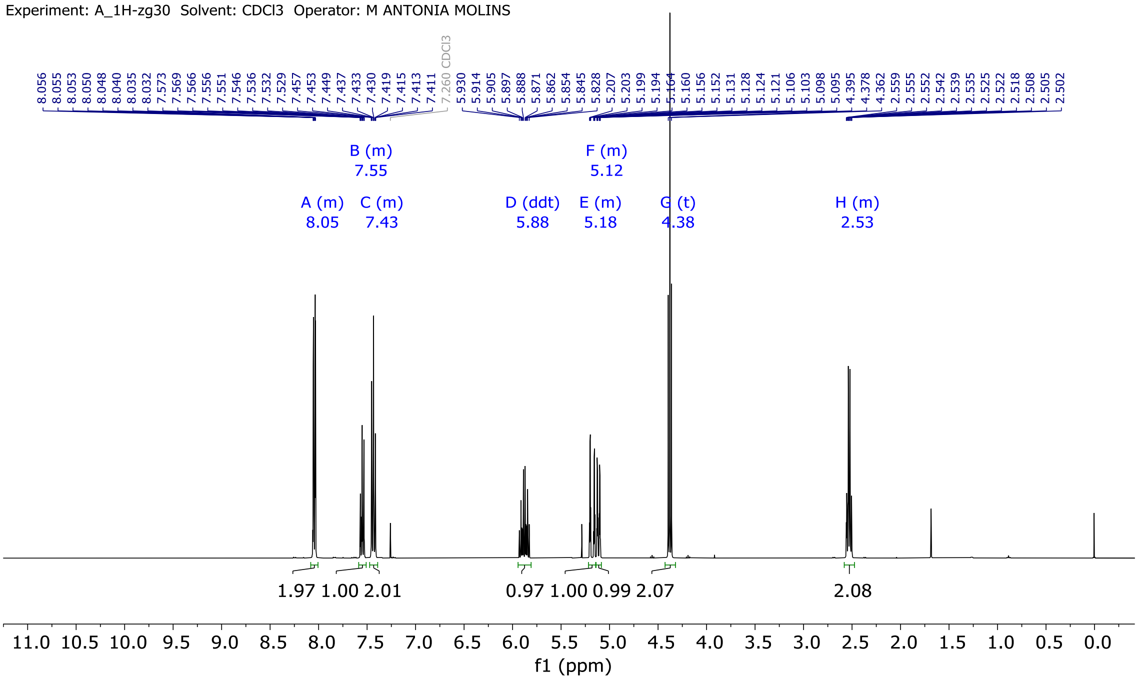

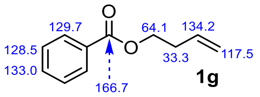

1g

san-1991-2023.2.fid 13C{1H} 101 MHz
Equip: B400Q / N.Inv: 1035091
N.Reg: 1991/2023
Usuari: san / Mostra: LRGJPB525CH
Nom: LAURA RODRIGUEZ GONZALEZ
Data: 01/04/2023 11:14:09 h./ Ope.: servei Unitat RMN
Experiment: A_13C-zgpg30 Solvent: CDCl3 Operator: M ANTONIA MOLINS

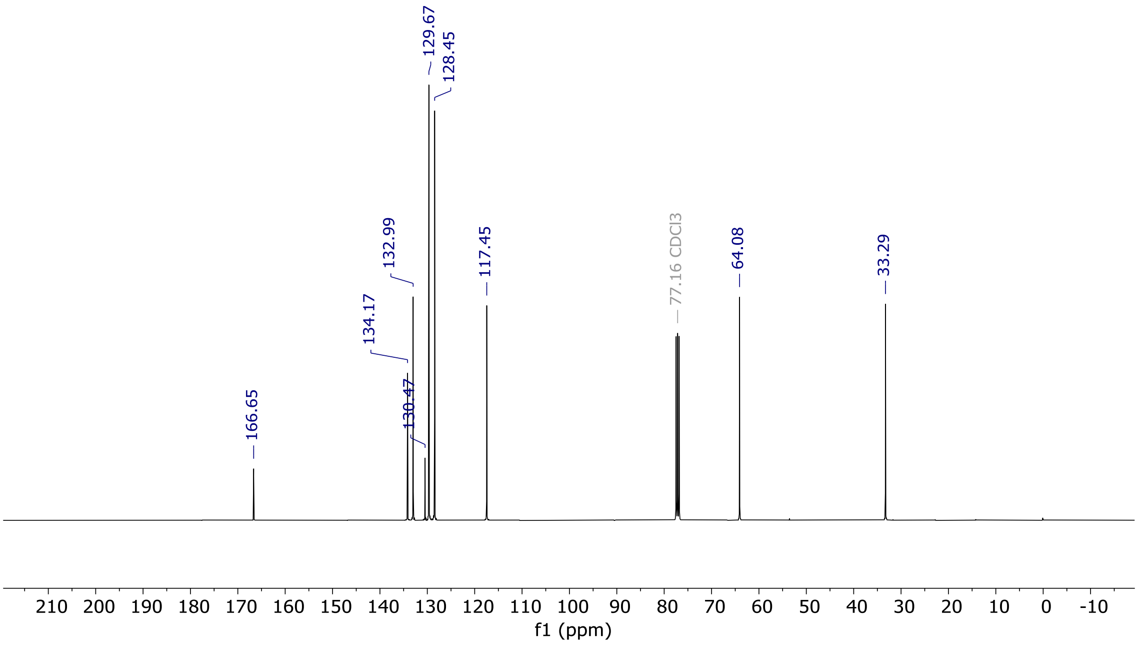

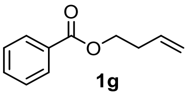

1g

2D-COSY

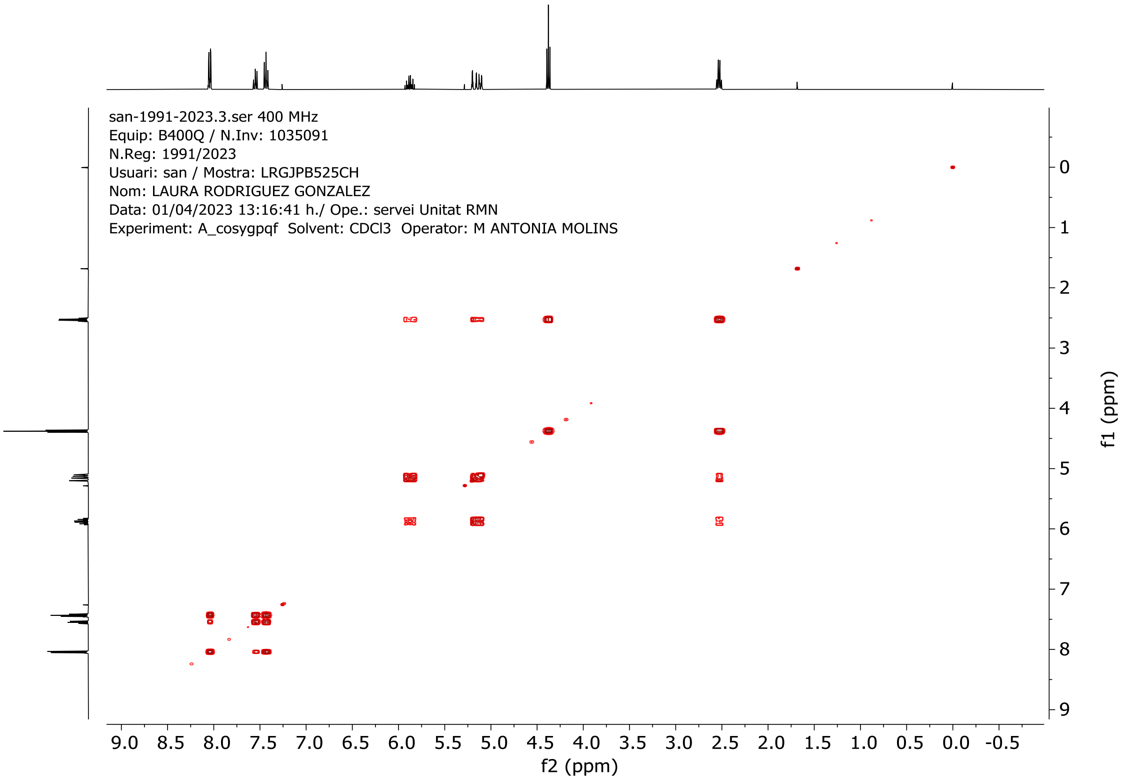

2D-HSQC

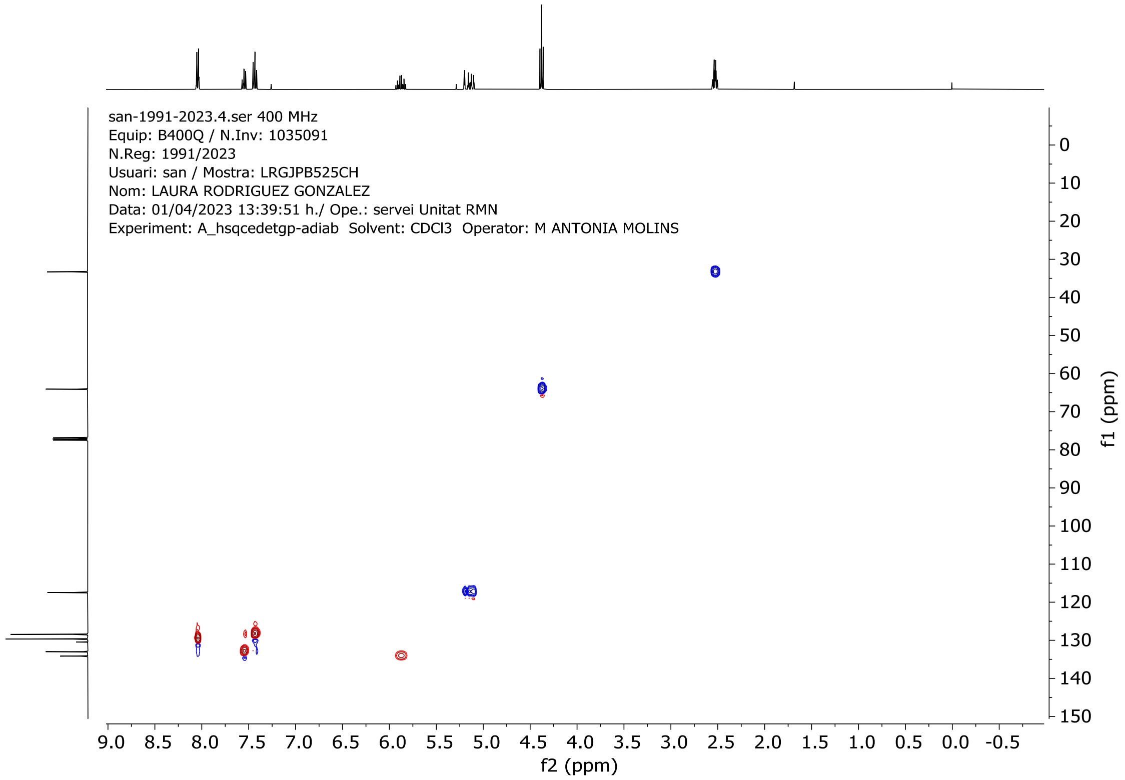

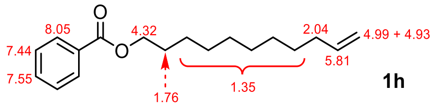

1h

2585-2023_B400FA_04052023_LRG325CH.1.fid 1H 400 MHz
Equip: B400F / N.Inv: 1037597
N.Reg: 2585/2023
Usuari: san / Mostra: LRG325CH
Nom: LAURA RODRIGUEZ GONZALEZ
Data: 03/05/2023 10:03:44 h./ Ope.: servei Unitat RMN
Experiment: A-H1-zg30 Solvent: CDCl3

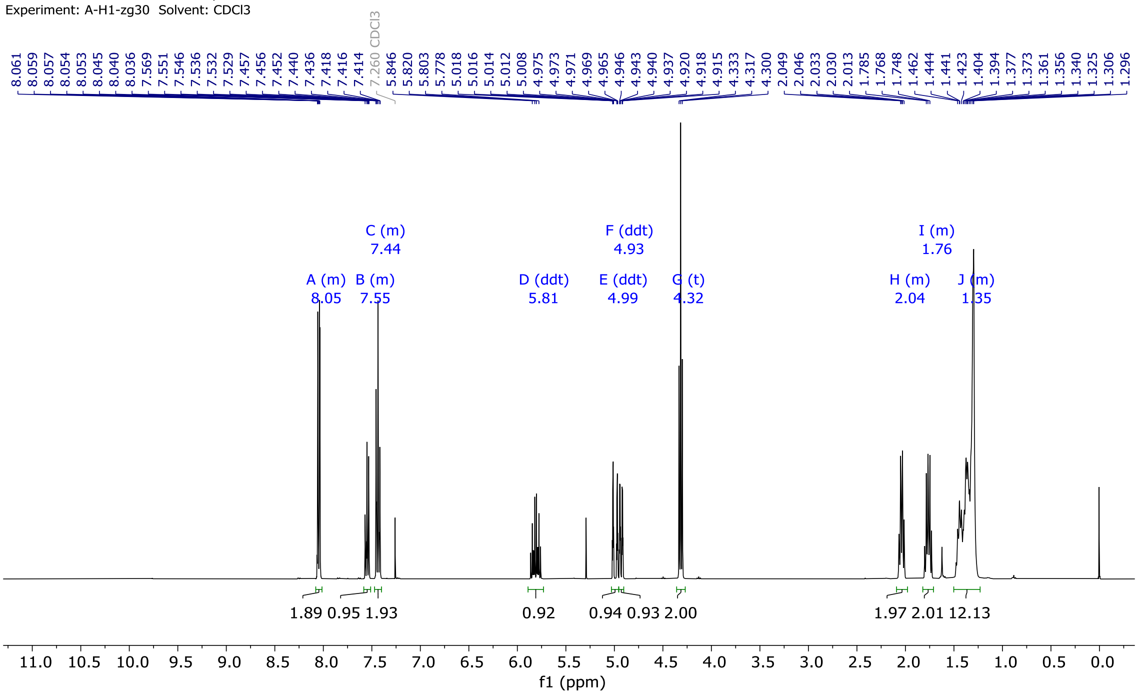

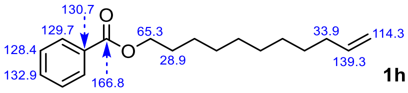

1h

2585-2023_B400FA_04052023_LRG325CH.4.fid 13C{1H} 101 MHz
Equip: B400F / N.Inv: 1037597
N.Reg: 2585/2023
Usuari: san / Mostra: LRG325CH
Nom: LAURA RODRIGUEZ GONZALEZ
Data: 03/05/2023 22:25:42 h./ Ope.: servei Unitat RMN
Experiment: A-C13-zgpg30 Solvent: CDCl3

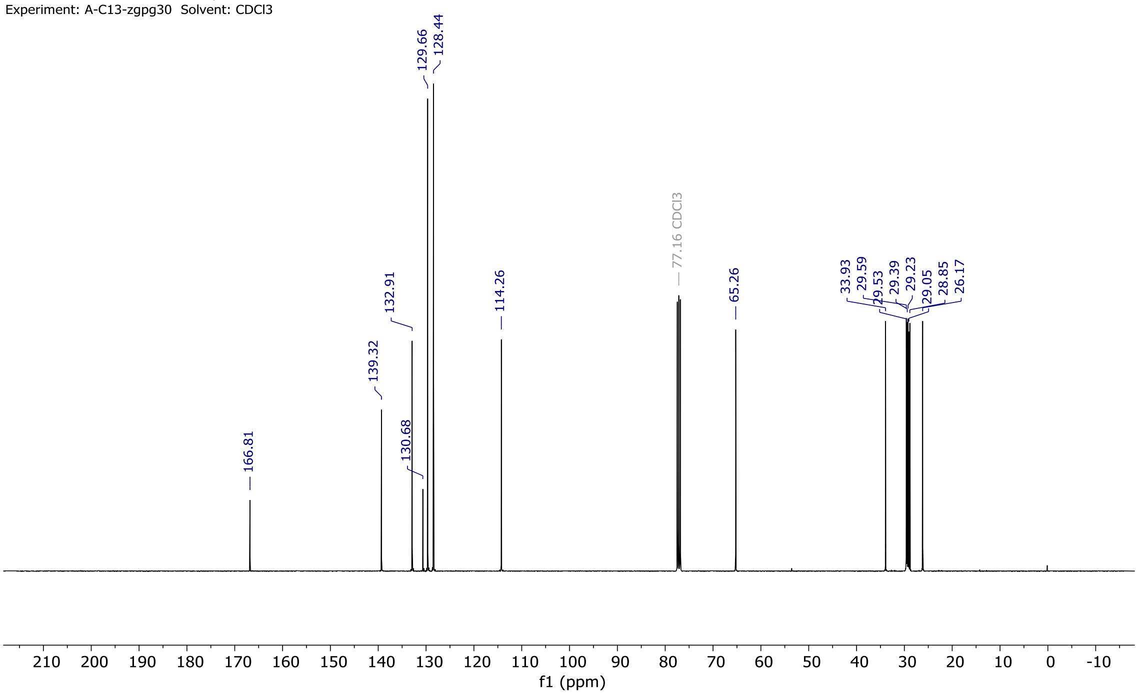

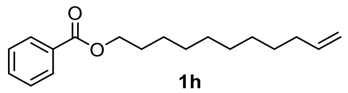

1h

2D-COSY

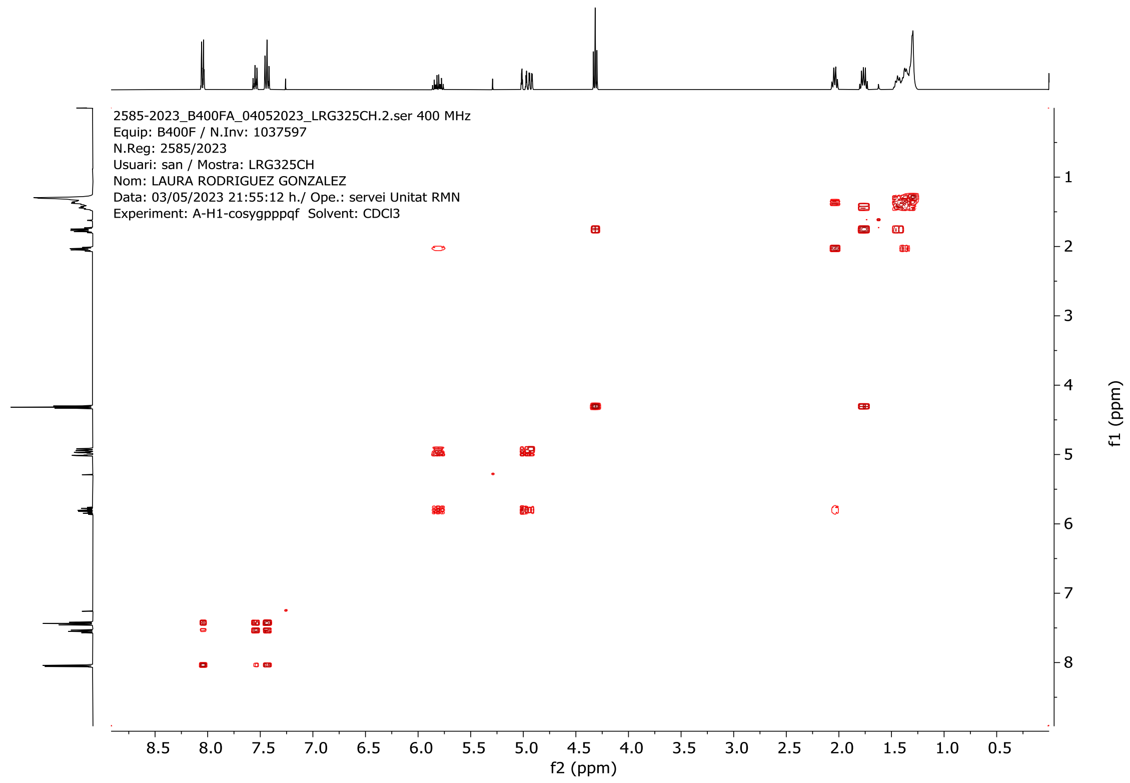

2D-HSQC

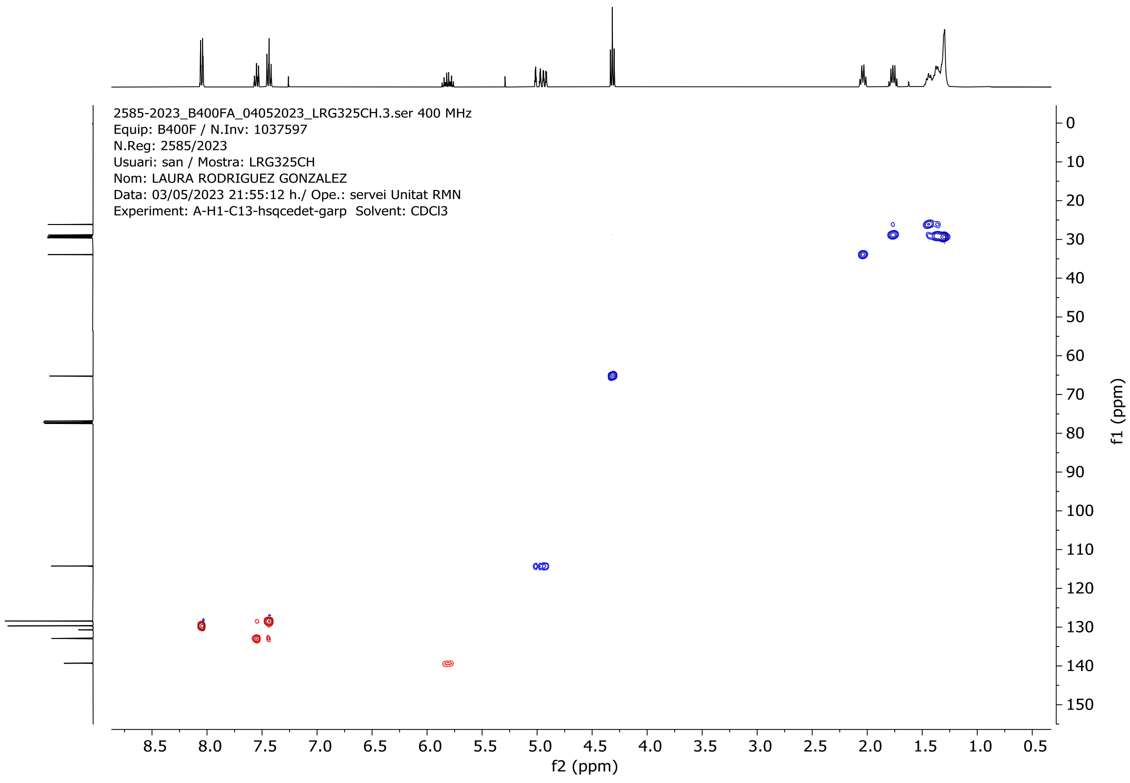

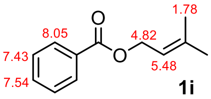

1i

23100207_B400FA_23102023_LRG483COLT4T18.1.fid 1H 400 MHz
Equip: B400F / N.Inv: 1037597
N.Reg: 23100207
Usuari: san / Mostra: LRG483COLT4T18
Nom: LAURA RODRIGUEZ GONZALEZ
Data: 23/10/2023 17:41:07 h./ Ope.: AUTOSERVEI
Experiment: A-H1-zg30 Solvent: CDCl3

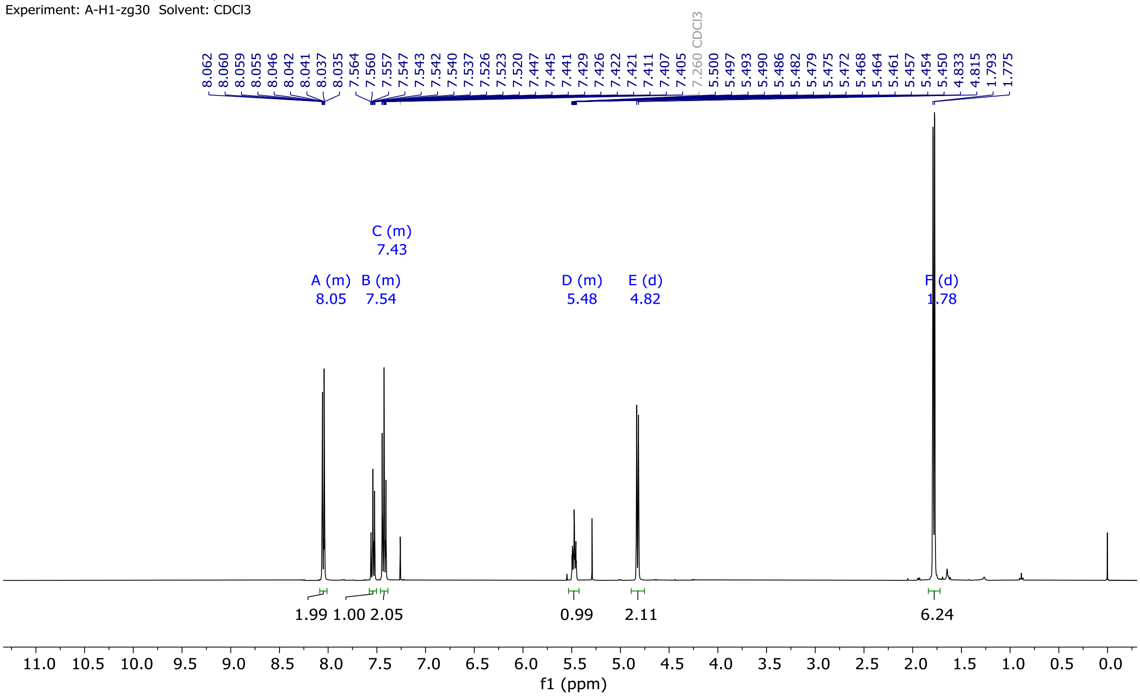

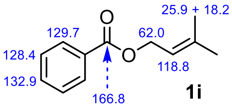

1i

23100211_B400FA_23102023_LRG483CH.2.fid 13C{1H} 101 MHz
Equip: B400F / N.Inv: 1037597
N.Reg: 23100211
Usuari: san / Mostra: LRG483CH
Nom: LAURA RODRIGUEZ GONZALEZ
Data: 23/10/2023 20:27:31 h./ Ope.: AUTOSERVEI
Experiment: A-C13-zgpg30 Solvent: CDCl3

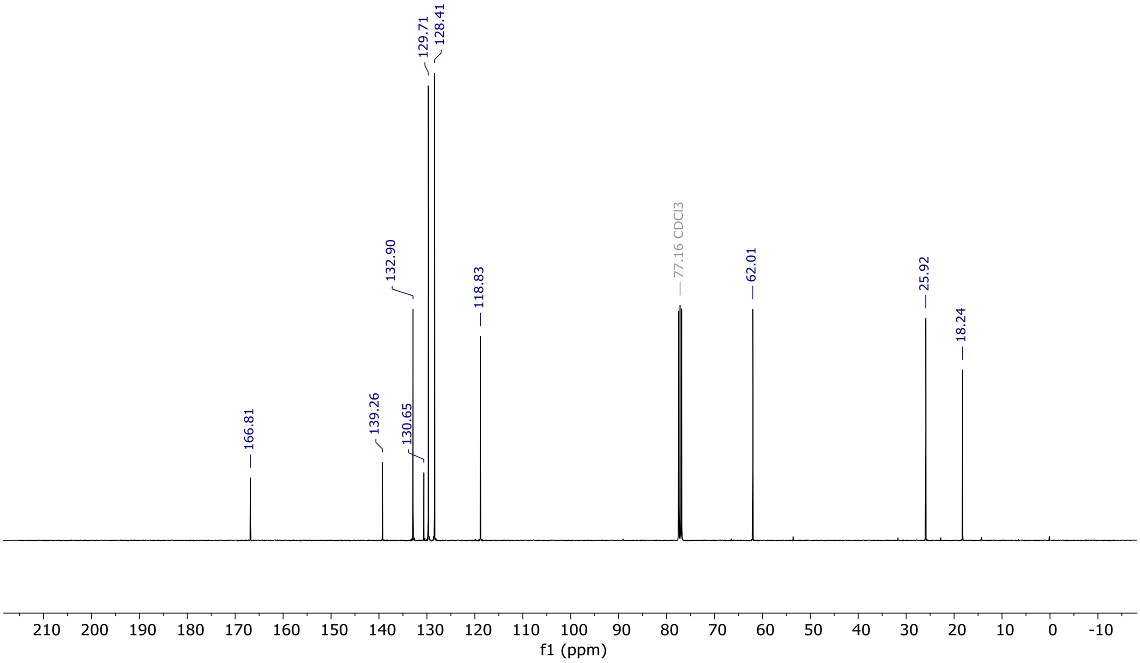

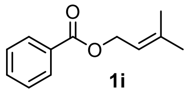

1i

2D-COSY

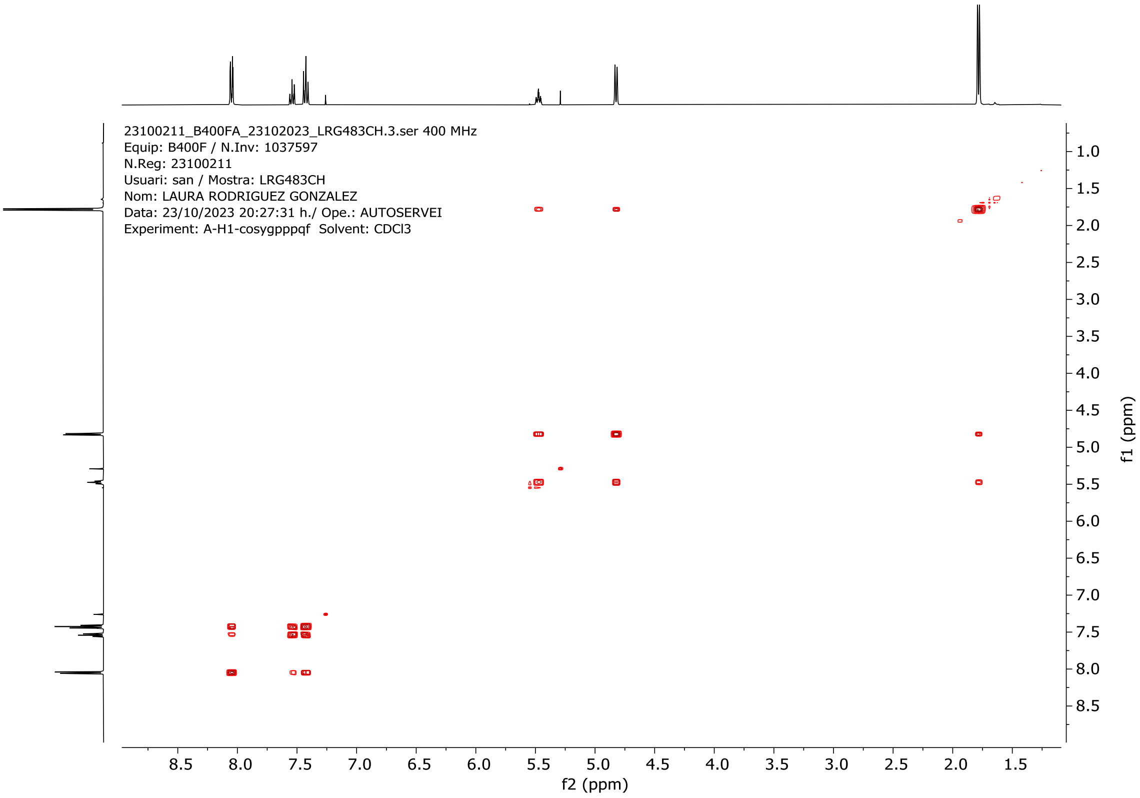

2D-HSQC

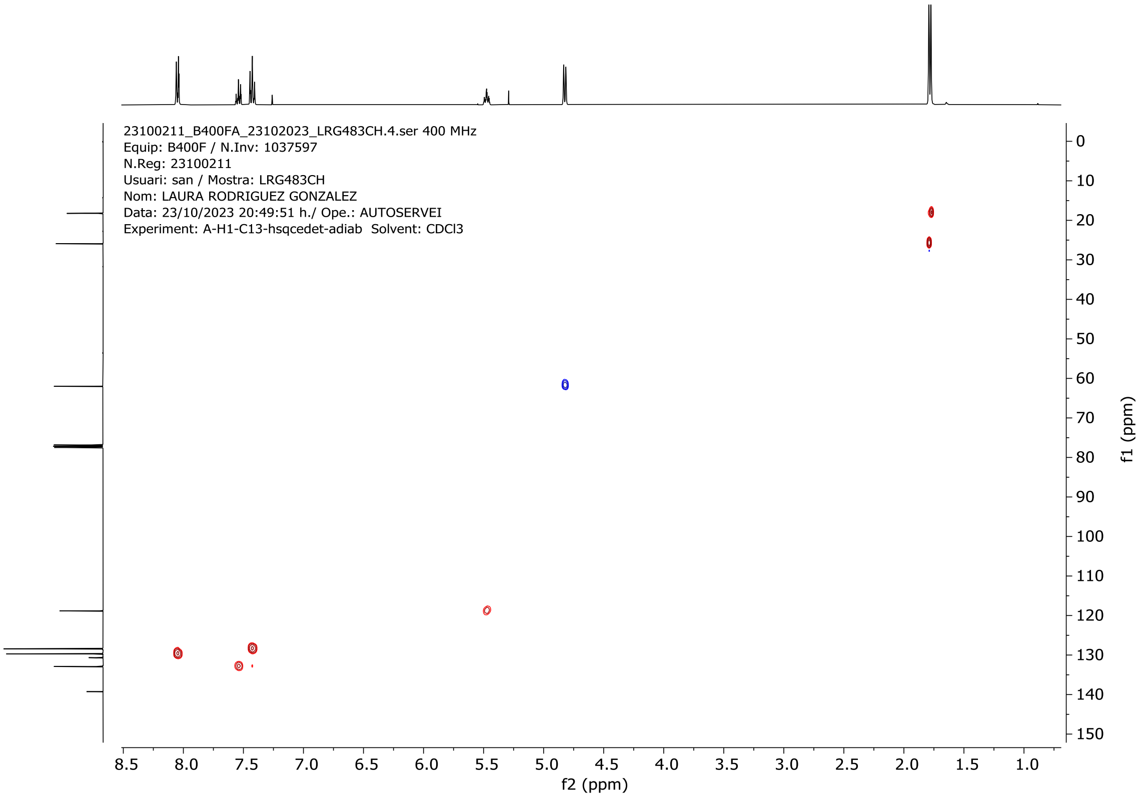

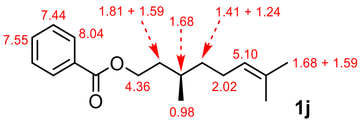

1j

san-1892-2023.1.fid 1H 400 MHz
Equip: B400Q / N.Inv: 1035091
N.Reg: 1892/2023
Usuari: san / Mostra: LRG363CH
Nom: LAURA RODRIGUEZ GONZALEZ
Data: 29/03/2023 15:42:23 h./ Ope.: servei Unitat RMN
Experiment: A_1H-zg30 Solvent: CDCl3 Operator: FRANCISCO CARDENAS LOPEZ

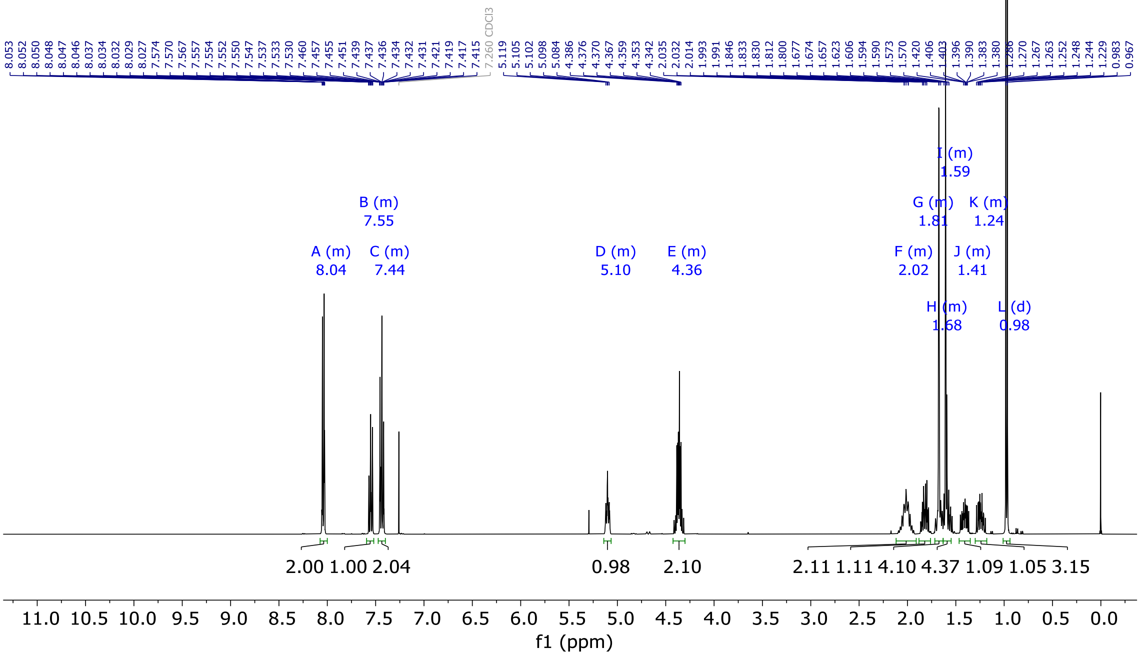

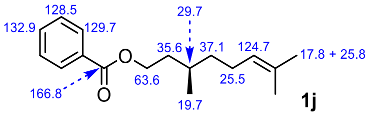

1j

san-1892-2023.3.fid 13C{1H} 101 MHz
Equip: B400Q / N.Inv: 1035091
N.Reg: 1892/2023
Usuari: san / Mostra: LRG363CH
Nom: LAURA RODRIGUEZ GONZALEZ
Data: 29/03/2023 16:05:35 h./ Ope.: servei Unitat RMN
Experiment: A_13C-zgpg30 Solvent: CDCl3 Operator: FRANCISCO CARDENAS LOPEZ

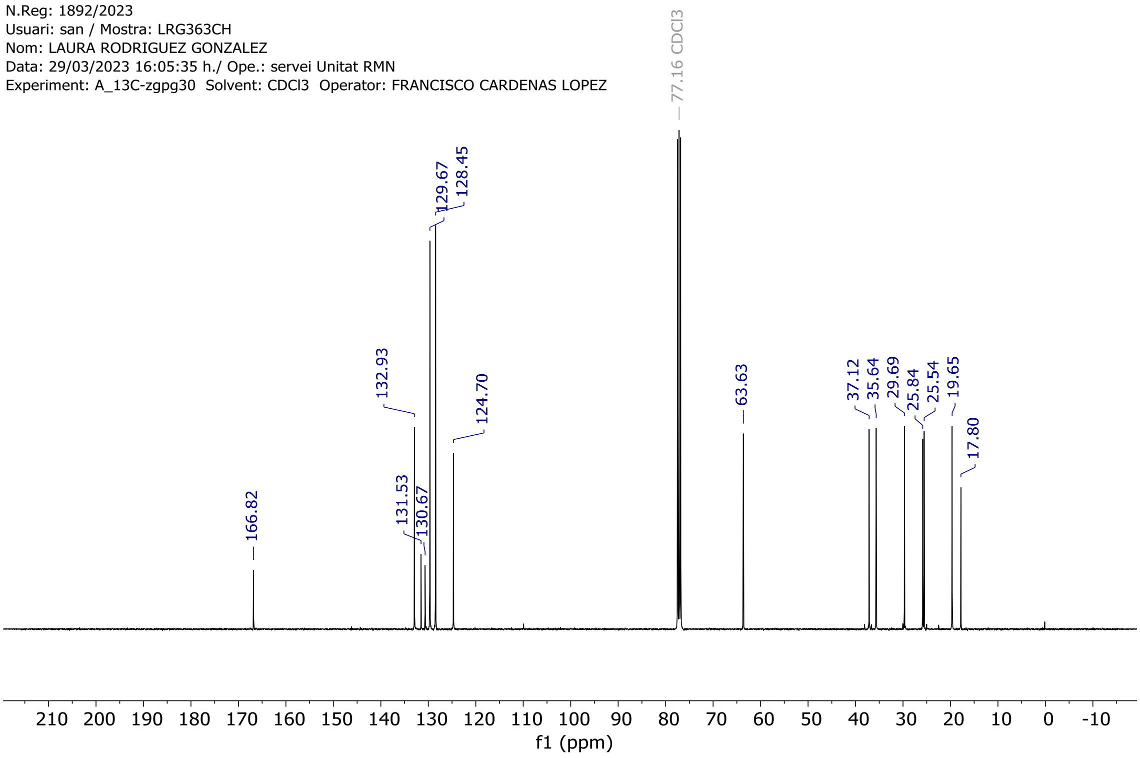

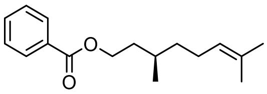

1j

2D-COSY

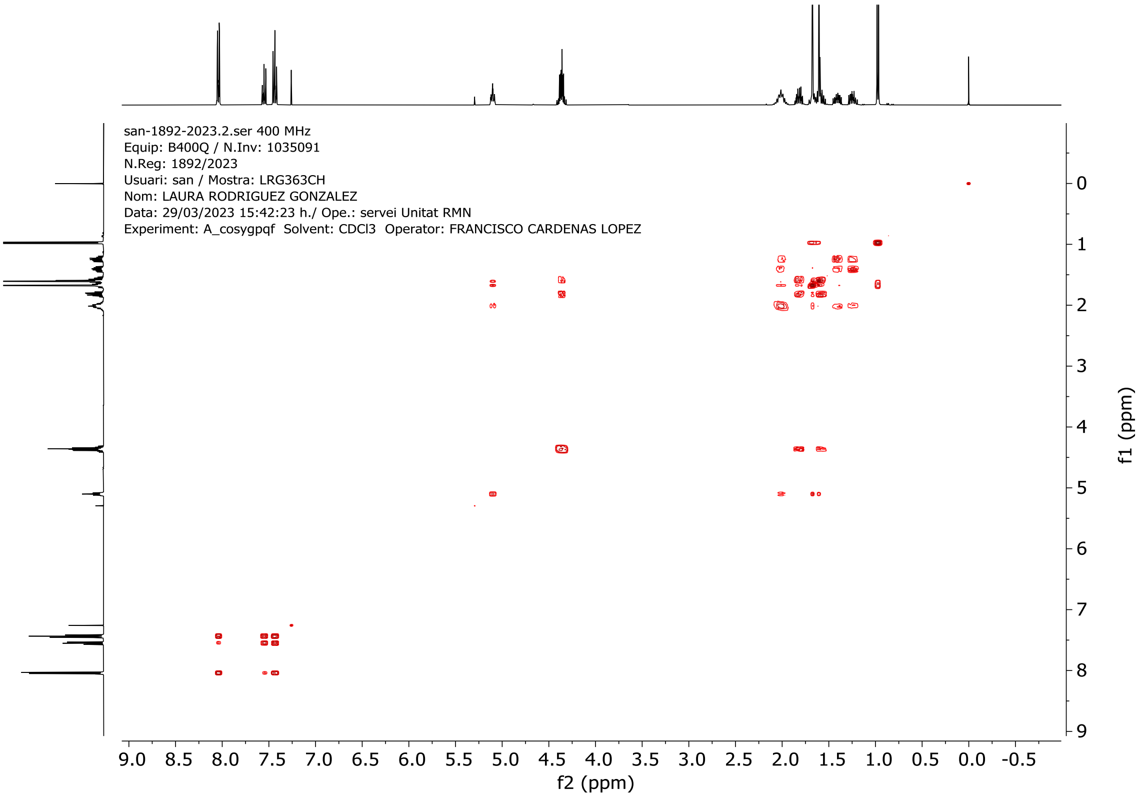

2D-HSQC

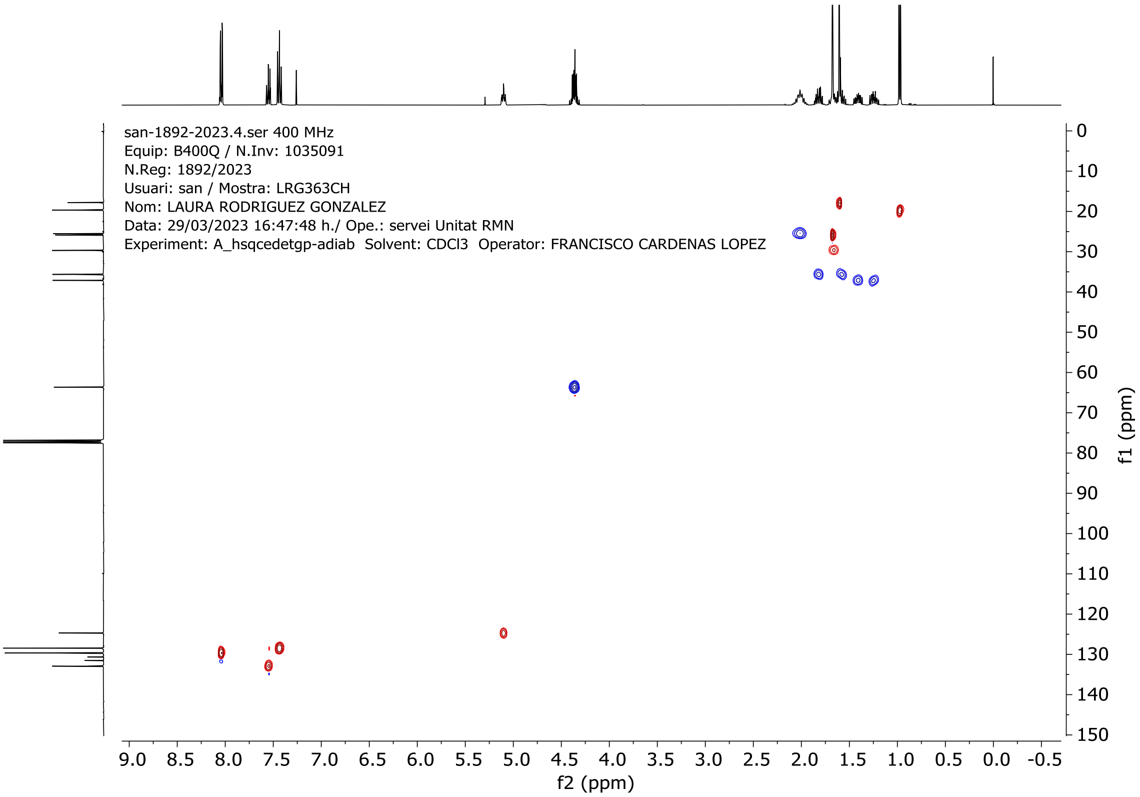

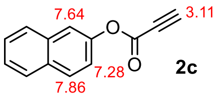

2c

24110213_B400FA_12112024_BB3001.1.fid 1H 400 MHz
Equip: B400F / N.Inv: 1037597
N.Reg: 24110213
Usuari: san / Mostra: BB3001
Nom: LAURA RODRIGUEZ GONZALEZ
Data: 12/11/2024 13:00:41 h./ Ope.: AUTOSERVEI
Experiment: A-H1-zg30 Solvent: CDCl3

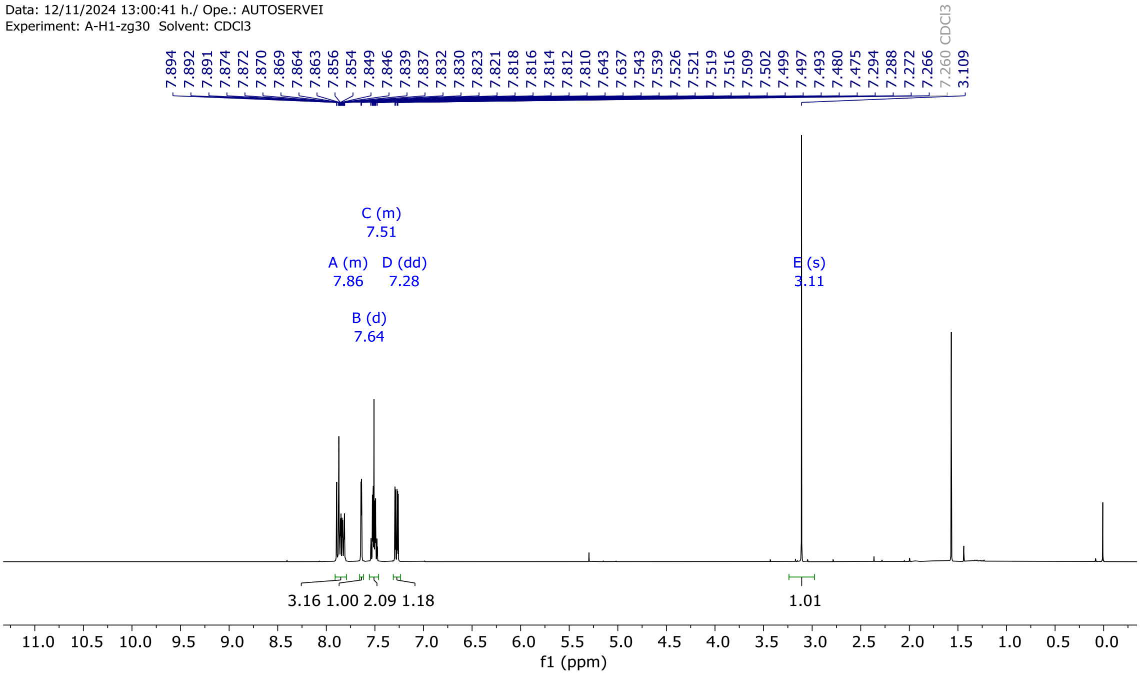

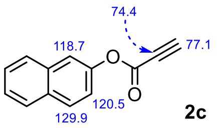

2c

24110213_B400FA_13112024_BB3001.2.fid 13C{1H} 101 MHz
Equip: B400F / N.Inv: 1037597
N.Reg: 24110213
Usuari: san / Mostra: BB3001
Nom: LAURA RODRIGUEZ GONZALEZ
Data: 13/11/2024 06:15:11 h./ Ope.: AUTOSERVEI
Experiment: A-C13-zgpg30 Solvent: CDCl3

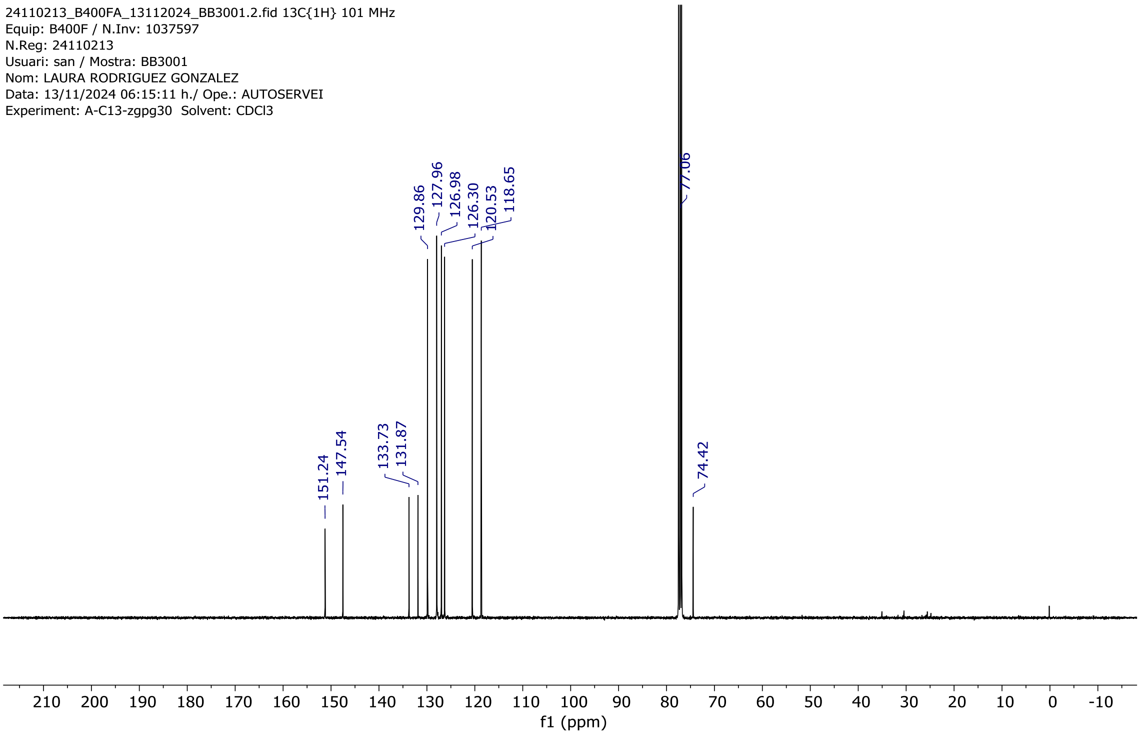

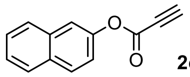

2c

2D-COSY

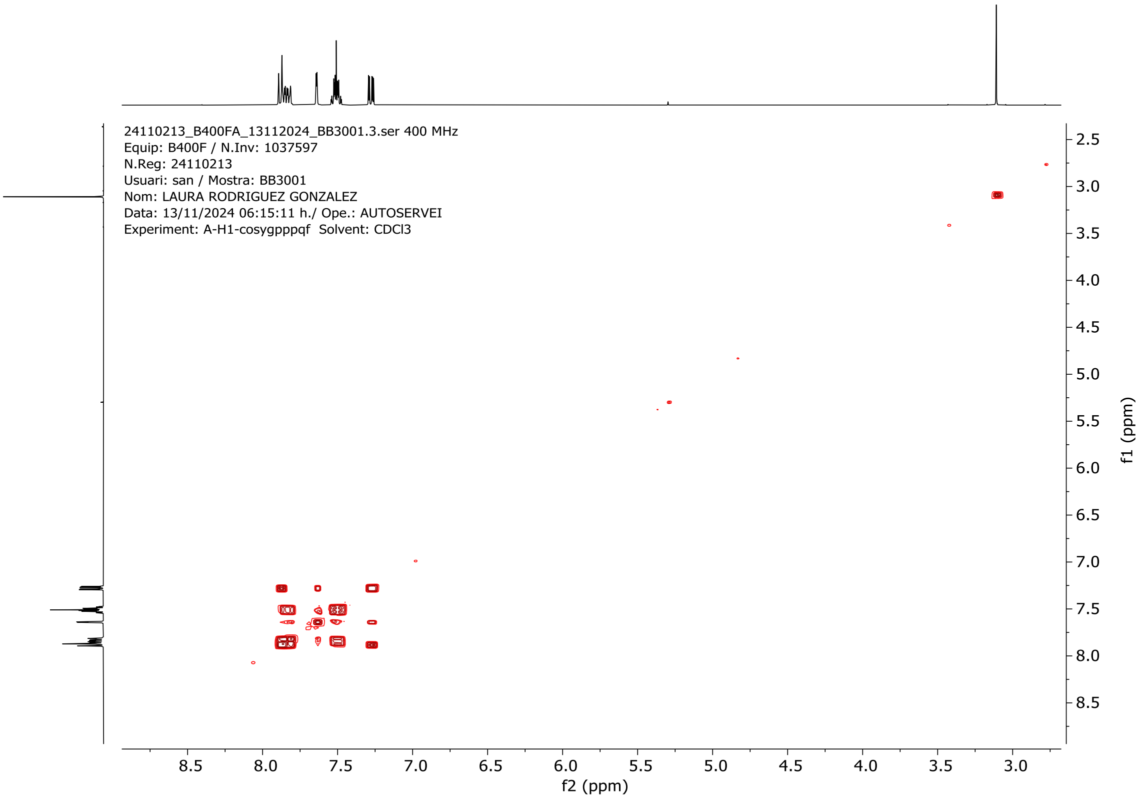

2D-HSQC

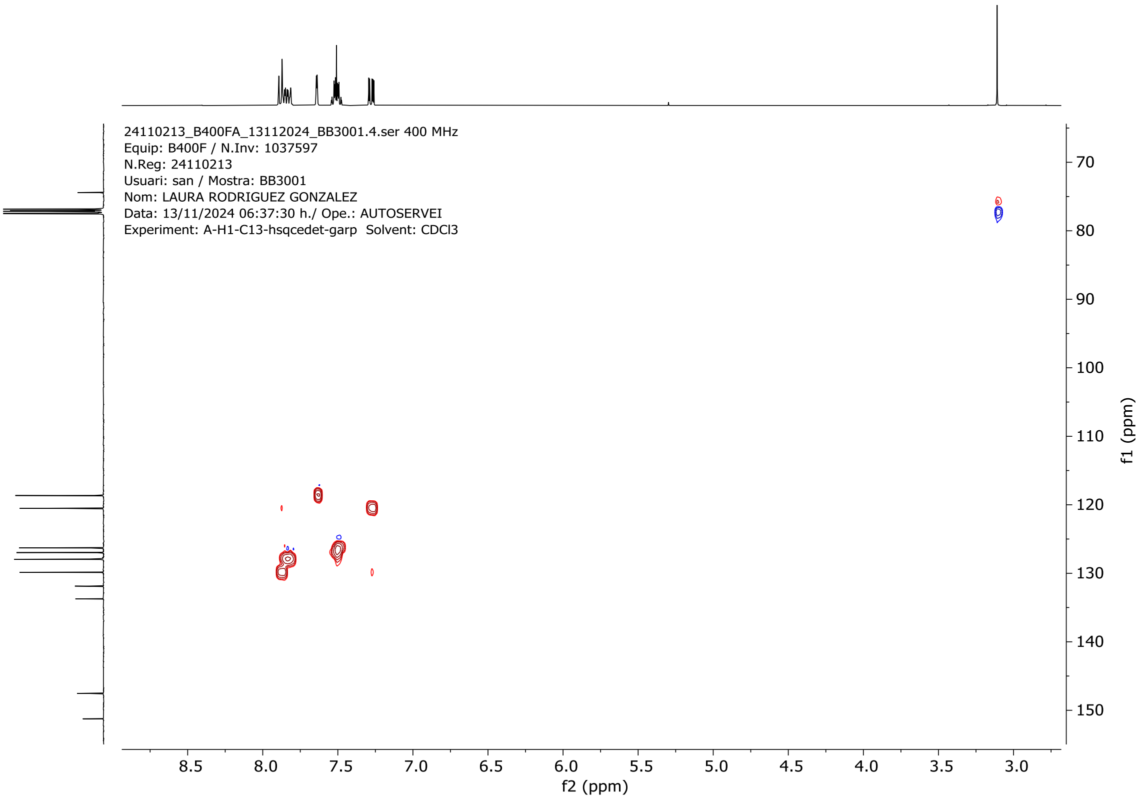

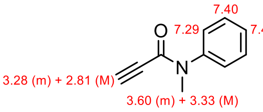

2d

23070179_B400FA_08072023_LRG396CH.1.fid 1H 400 MHz
Equip: B400F / N.Inv: 1037597
N.Reg: 23070179
Usuari: san / Mostra: LRG396CH
Nom: LAURA RODRIGUEZ GONZALEZ
Data: 07/07/2023 15:23:51 h./ Ope.: AUTOSERVEI
Experiment: A-H1-zg30 Solvent: CDCl3

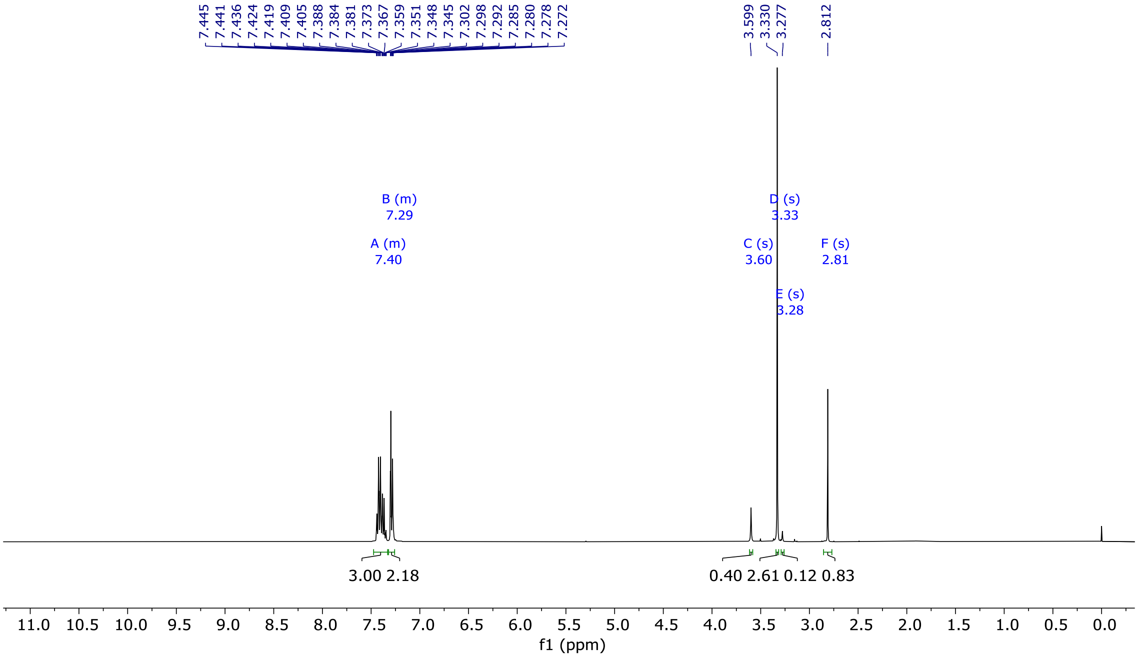

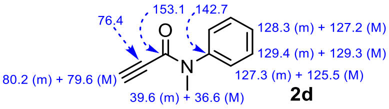

2d

23070179_B400FA_08072023_LRG396CH.2.fid 13C{1H} 101 MHz
Equip: B400F / N.Inv: 1037597
N.Reg: 23070179
Usuari: san / Mostra: LRG396CH
Nom: LAURA RODRIGUEZ GONZALEZ
Data: 08/07/2023 03:27:09 h./ Ope.: AUTOSERVEI
Experiment: A-C13-zgpg30 Solvent: CDCl3

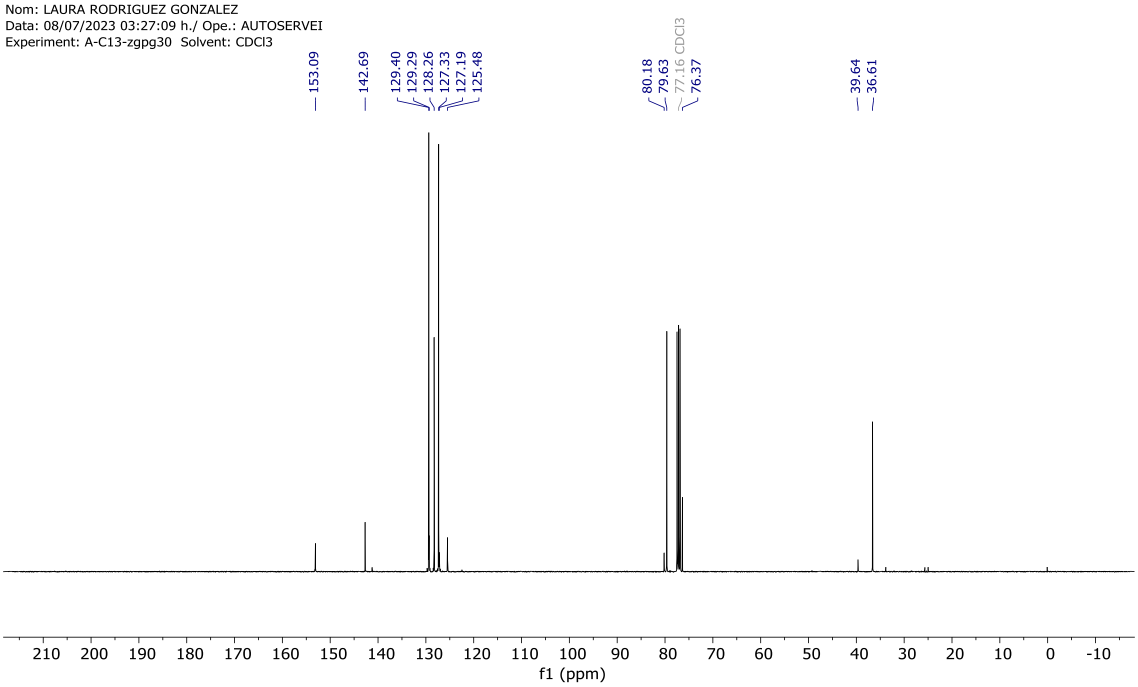

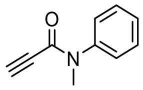

2d

2D-COSY

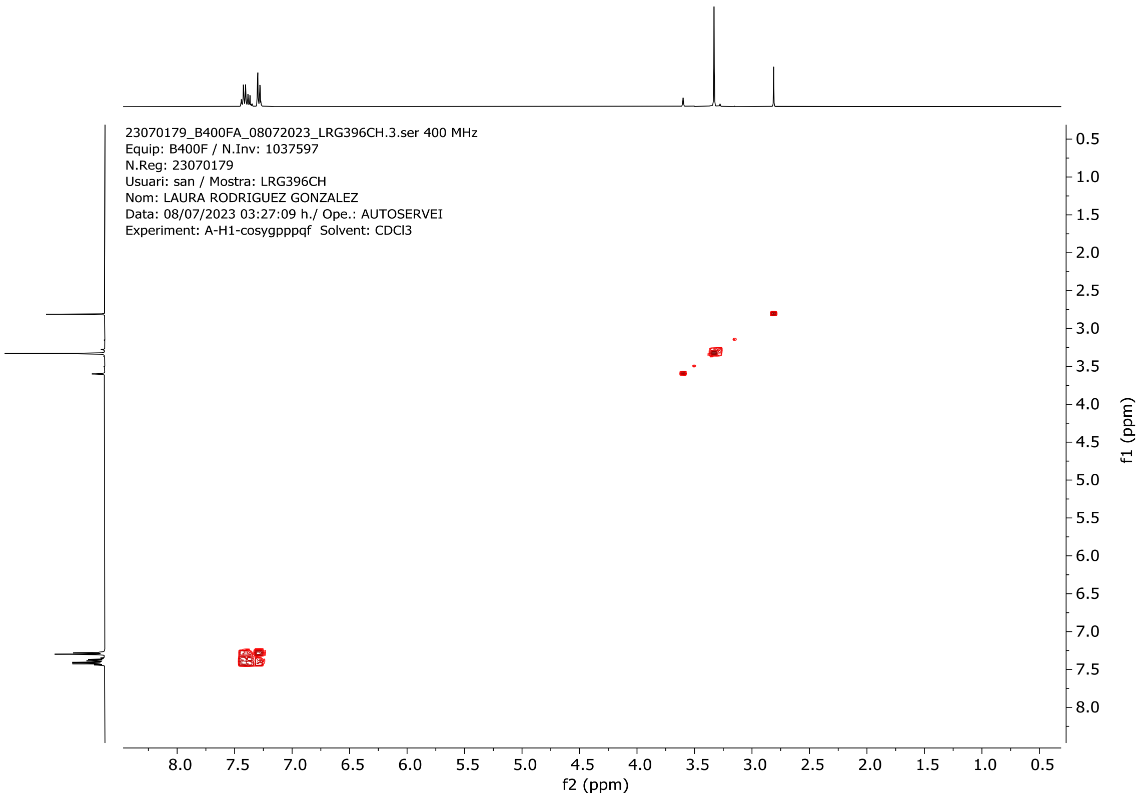

2D-HSQC

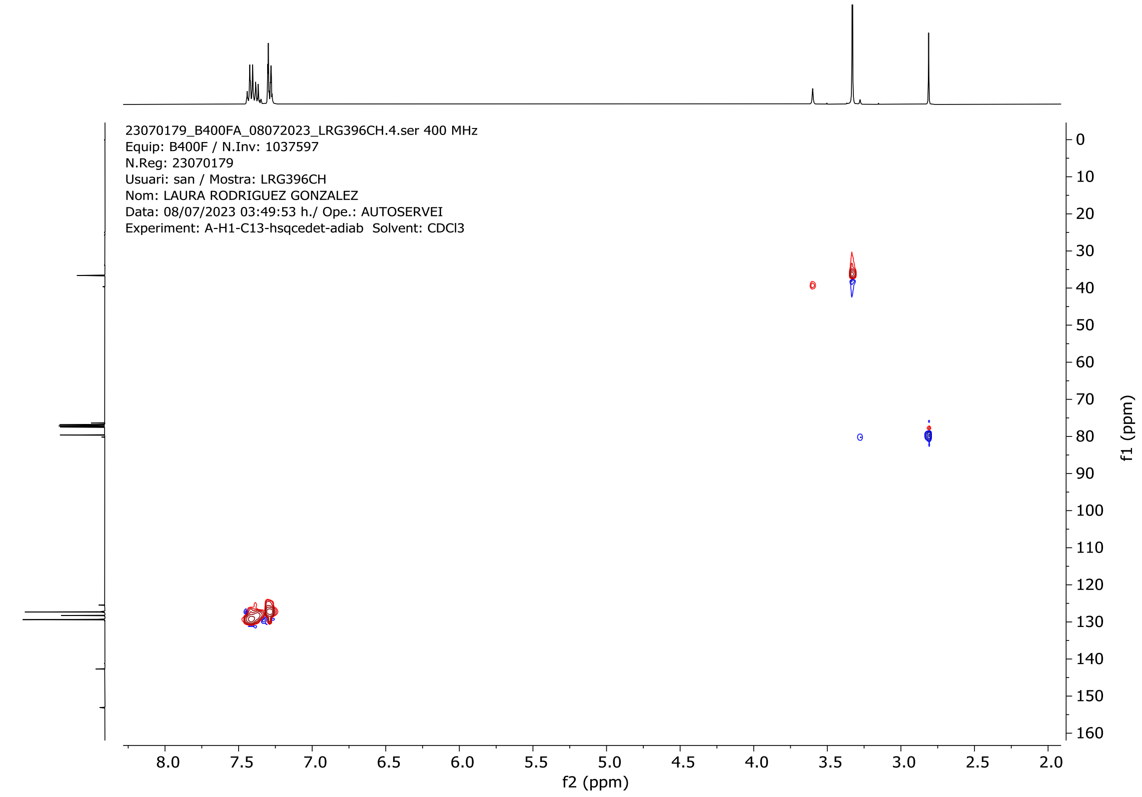

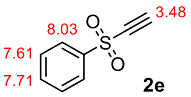

2e

LRG415COLT12T18.1.fid 1H 400 MHz
Equip: B400F / N.Inv: 1037597
N.Reg: 23070012
Usuari: san / Mostra: LRG415COLT12T18
Nom: LAURA RODRIGUEZ GONZALEZ
Data: 03/07/2023 14:26:00 h./ Ope.: AUTOSERVEI
Experiment: A-H1-zg30 Solvent: CDCl3

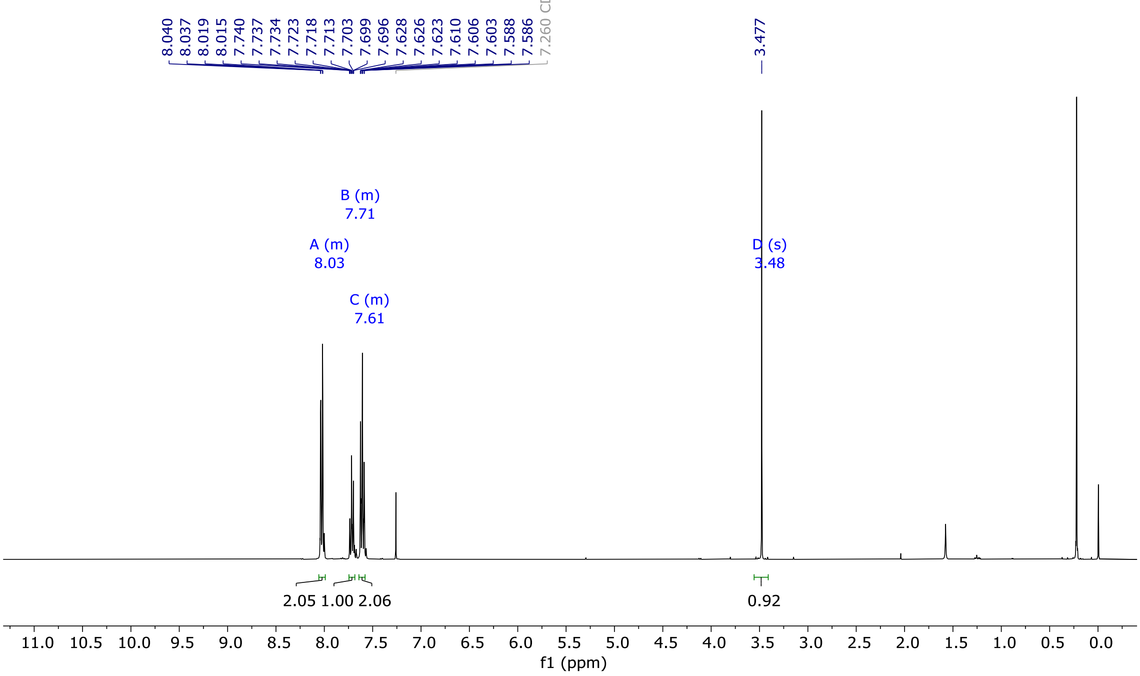

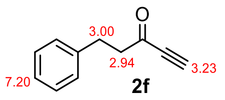

23060696_B400FA_28062023_LRG421CH.1.fid 1H 400 MHz
Equip: B400F / N.Inv: 1037597
N.Reg: 23060696
Usuari: san / Mostra: LRG421CH
Nom: LAURA RODRIGUEZ GONZALEZ
Data: 27/06/2023 18:49:48 h./ Ope.: AUTOSERVEI
Experiment: A-H1-zg30 Solvent: CDCl3

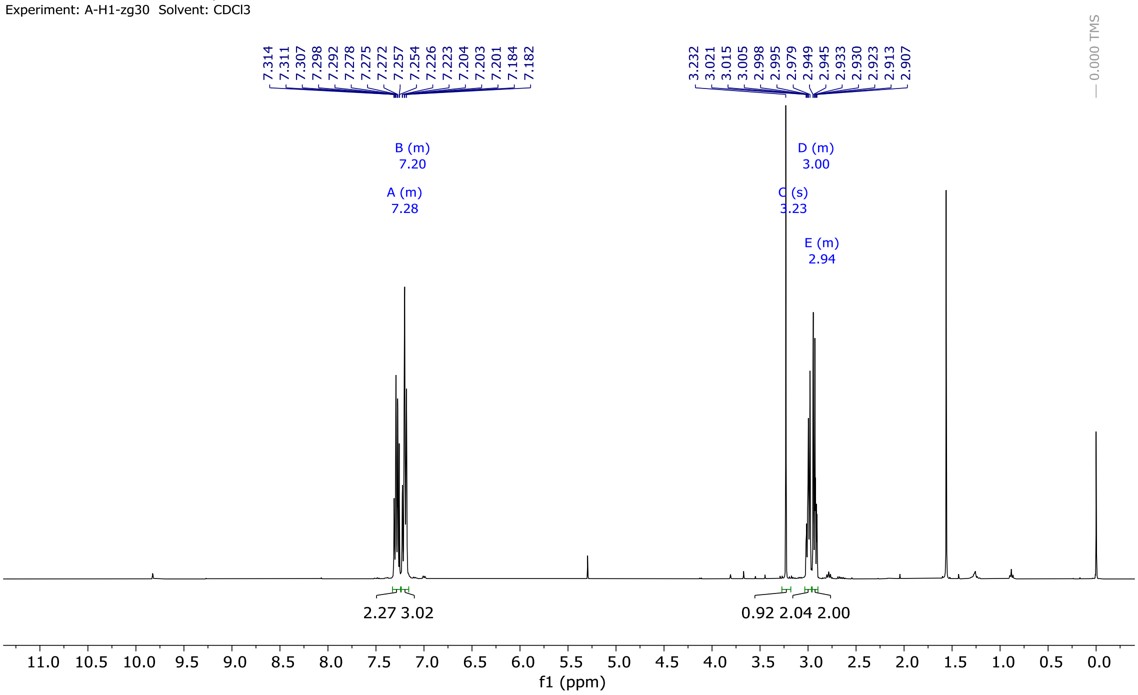

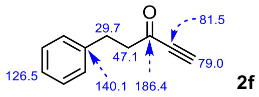

23060696_B400FA_28062023_LRG421CH.2.fid 13C{1H} 101 MHz
Equip: B400F / N.Inv: 1037597
N.Reg: 23060696
Usuari: san / Mostra: LRG421CH
Nom: LAURA RODRIGUEZ GONZALEZ
Data: 28/06/2023 07:05:44 h./ Ope.: AUTOSERVEI
Experiment: A-C13-zgpg30 Solvent: CDCl3

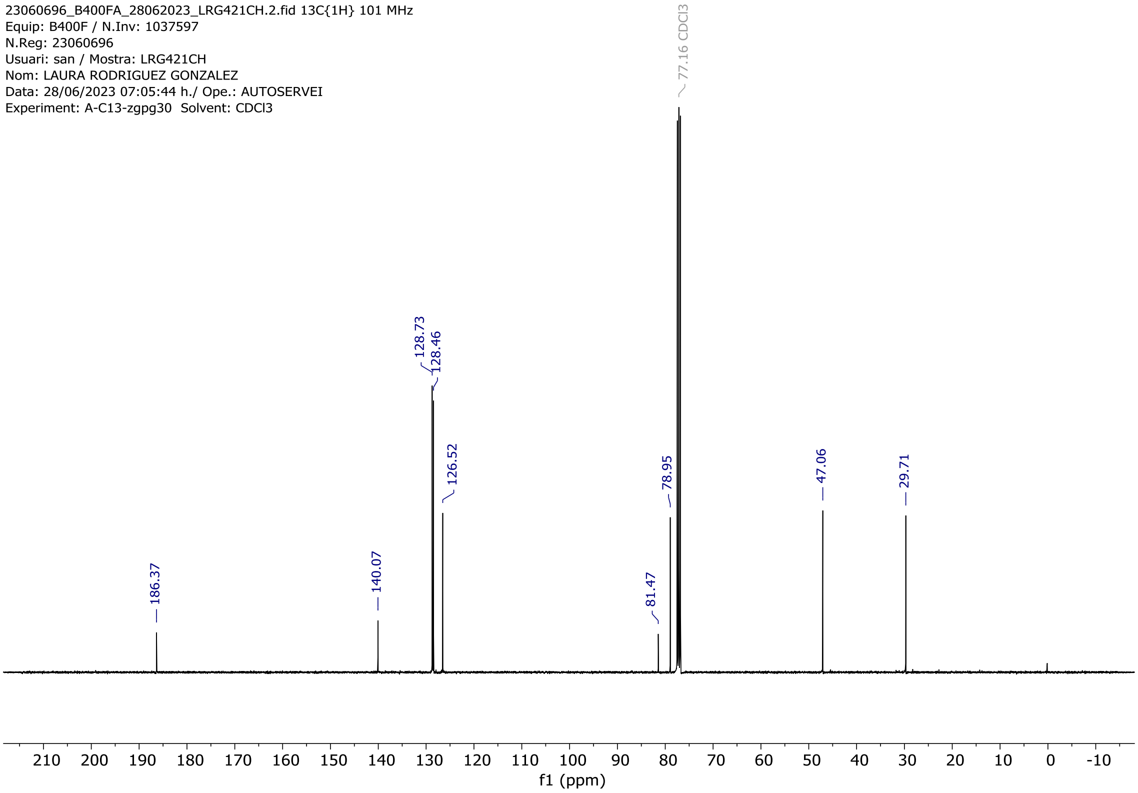

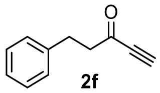

2f

2D-COSY

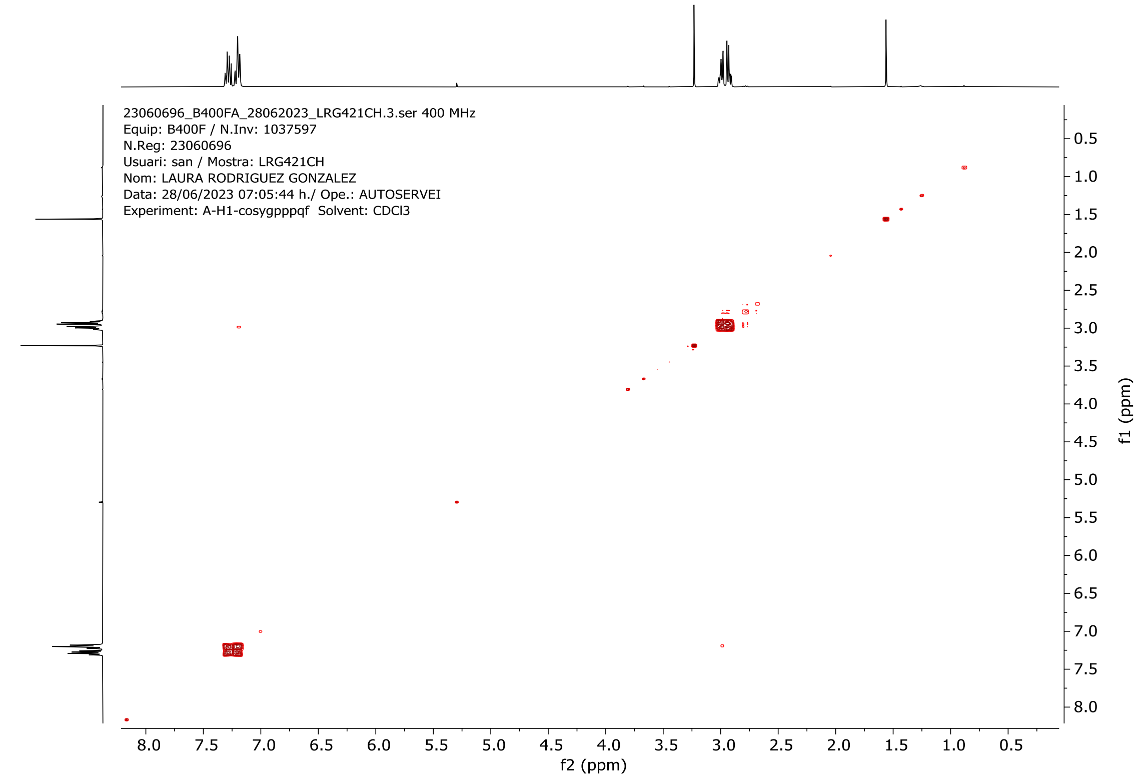

2D-HSQC

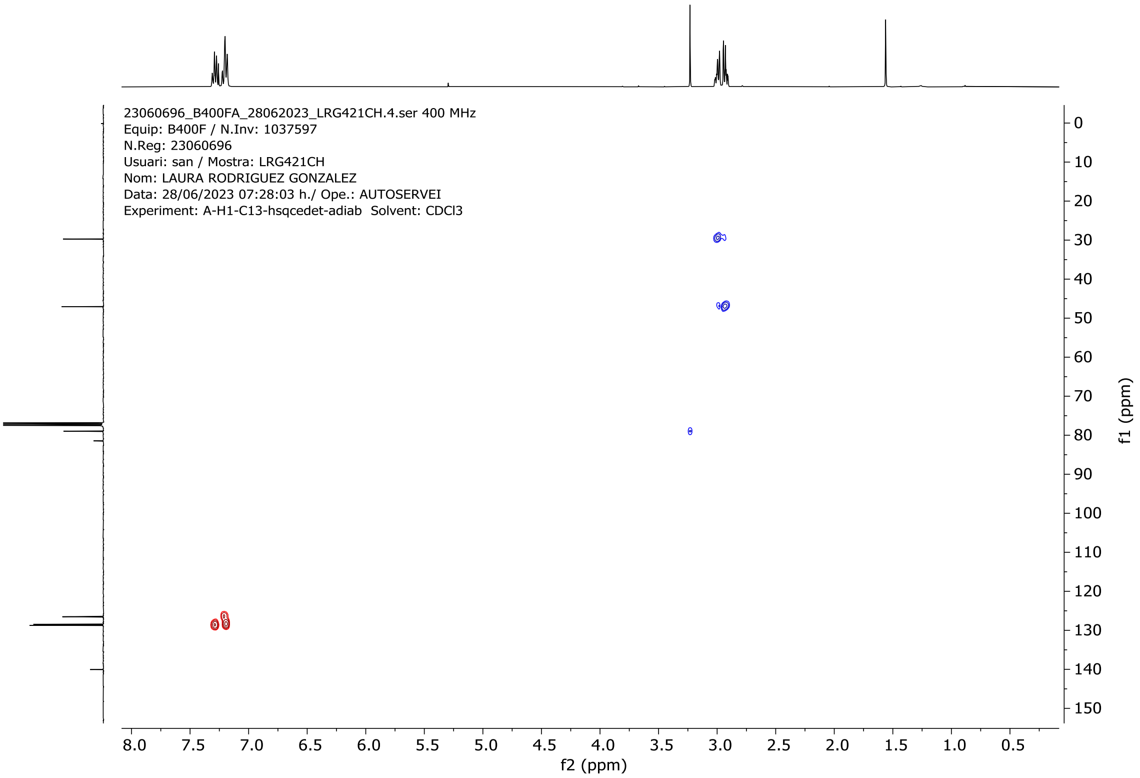

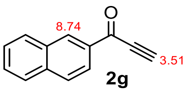

23070180_B400FA_08072023_LRG413CH.1.fid 1H 400 MHz
Equip: B400F / N.Inv: 1037597
N.Reg: 23070180
Usuari: san / Mostra: LRG413CH
Nom: LAURA RODRIGUEZ GONZALEZ
Data: 07/07/2023 15:29:39 h./ Ope.: AUTOSERVEI
Experiment: A-H1-zg30 Solvent: CDCl3

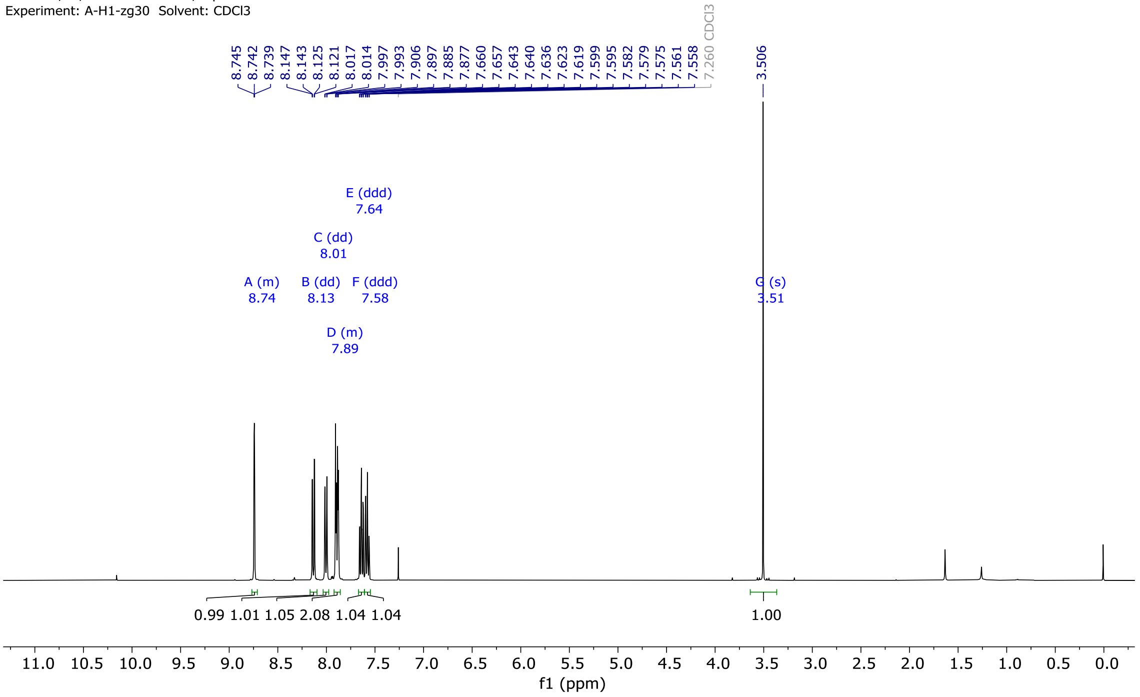

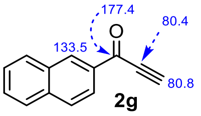

23070180_B400FA_08072023_LRG413CH.2.fid 13C{1H} 101 MHz
Equip: B400F / N.Inv: 1037597
N.Reg: 23070180
Usuari: san / Mostra: LRG413CH
Nom: LAURA RODRIGUEZ GONZALEZ
Data: 08/07/2023 06:13:49 h./ Ope.: AUTOSERVEI
Experiment: A-C13-zgpg30 Solvent: CDCl3

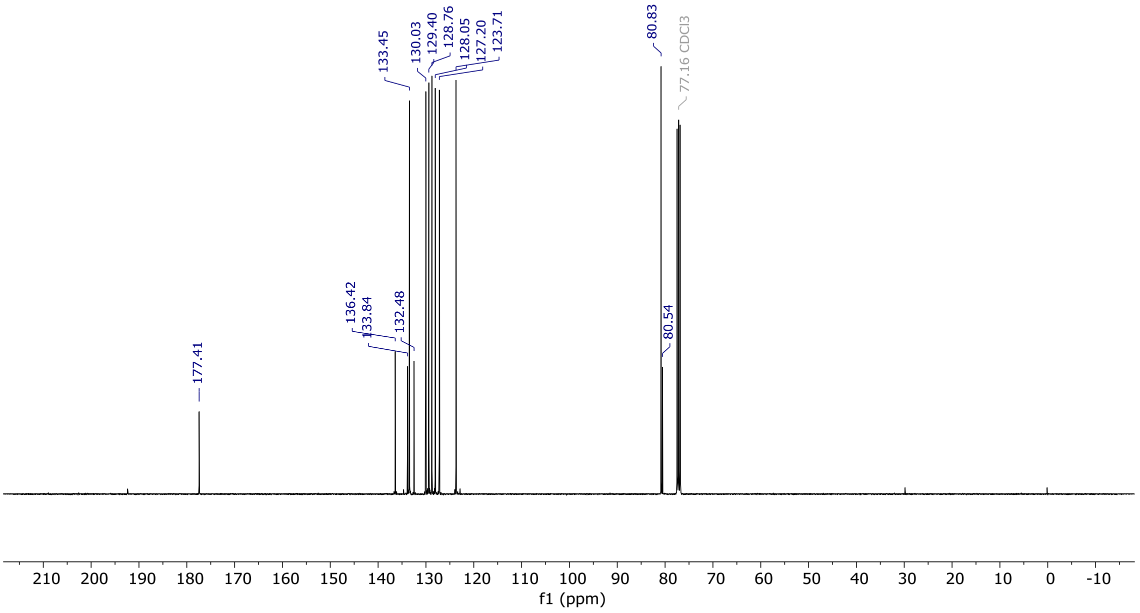

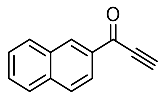

2g

2D-COSY

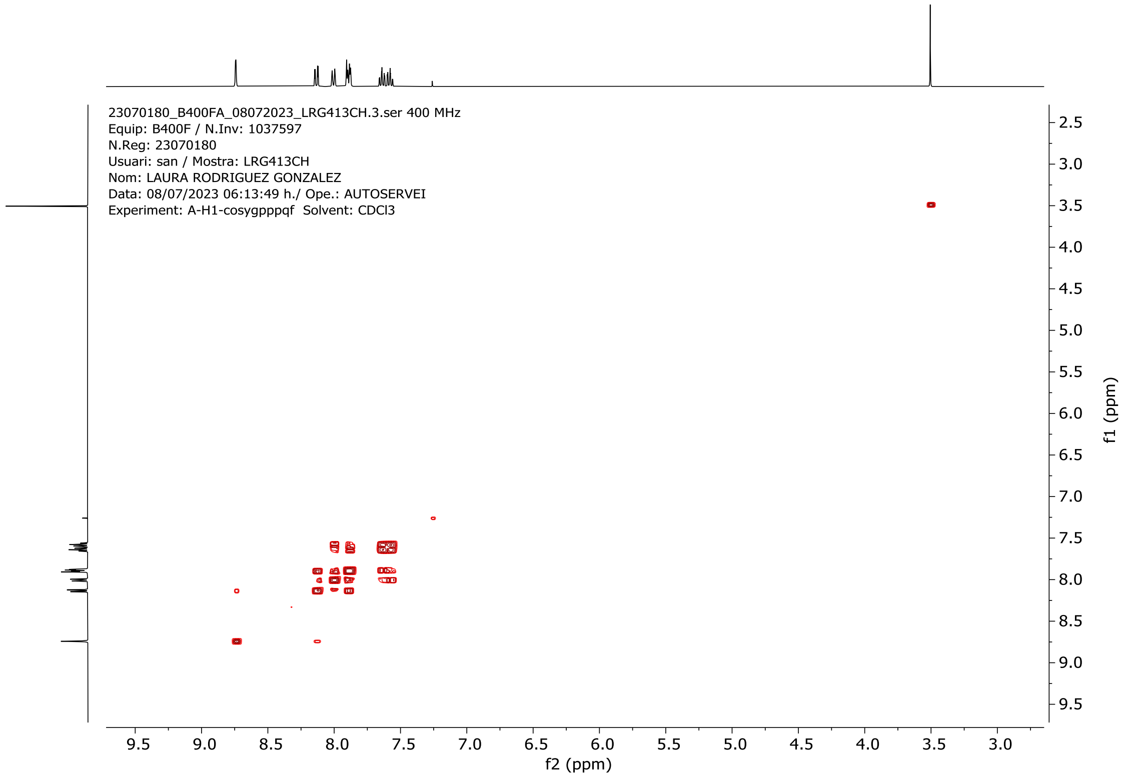

2D-HSQC

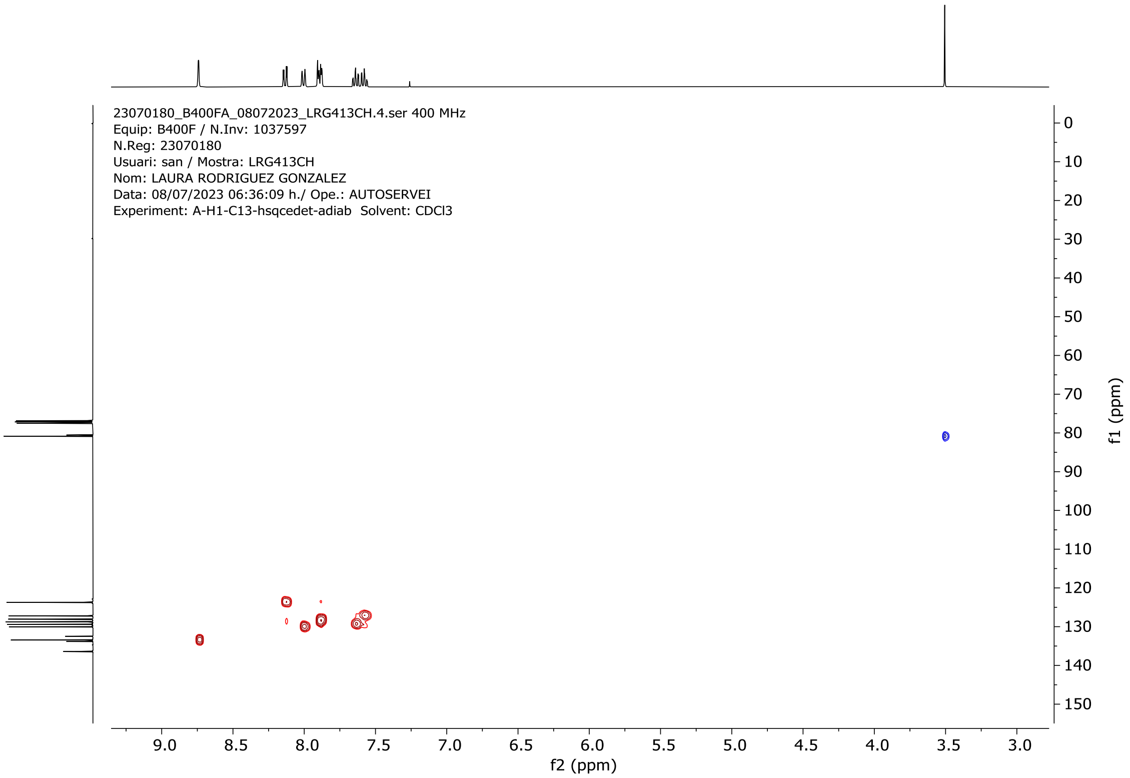

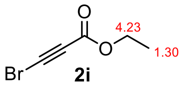

23070178_B400FA_08072023_LRG384CH.1.fid 1H 400 MHz
Equip: B400F / N.Inv: 1037597
N.Reg: 23070178
Usuari: san / Mostra: LRG384CH
Nom: LAURA RODRIGUEZ GONZALEZ
Data: 07/07/2023 15:17:30 h./ Ope.: AUTOSERVEI
Experiment: A-H1-zg30 Solvent: CDCl3

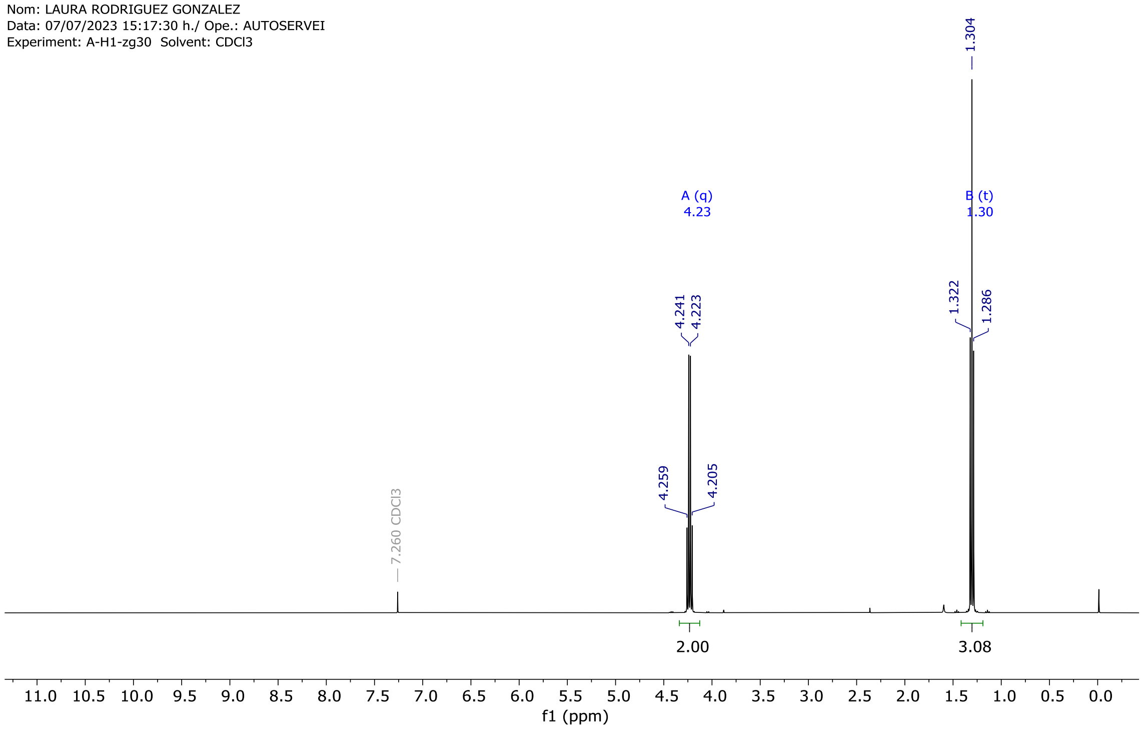

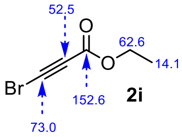

23070178_B400FA_08072023_LRG384CH.2.fid 13C{1H} 101 MHz
Equip: B400F / N.Inv: 1037597
N.Reg: 23070178
Usuari: san / Mostra: LRG384CH
Nom: LAURA RODRIGUEZ GONZALEZ
Data: 08/07/2023 00:41:33 h./ Ope.: AUTOSERVEI
Experiment: A-C13-zgpg30 Solvent: CDCl3

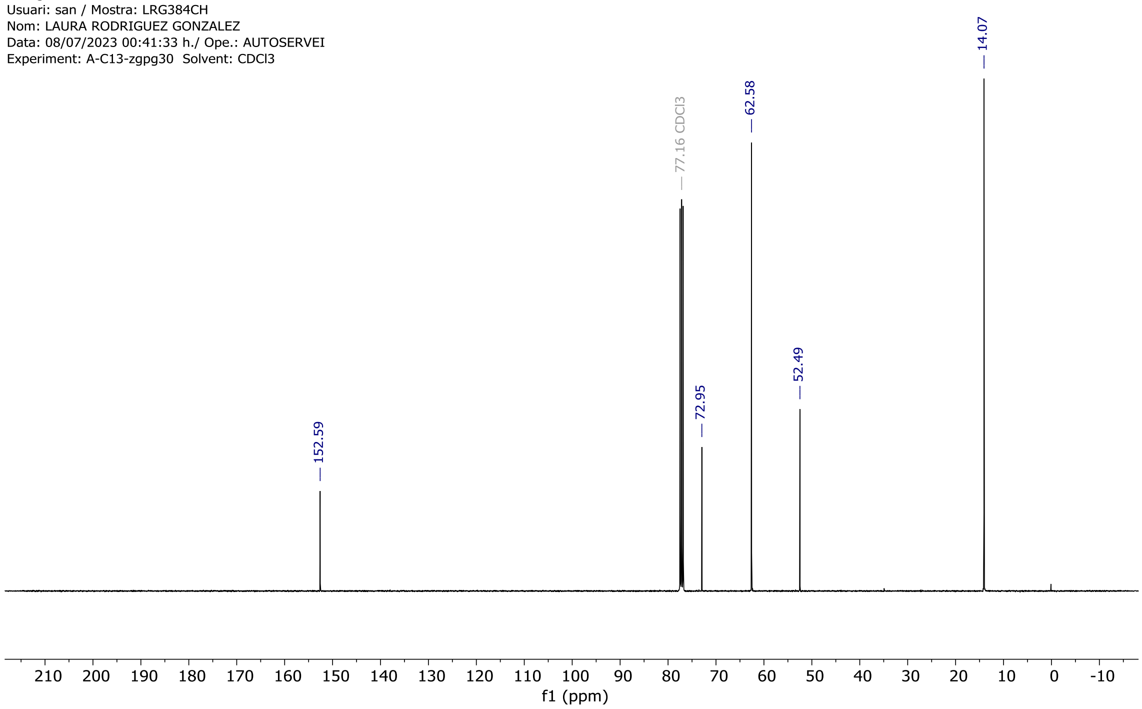

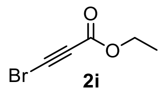

2i

2D-COSY

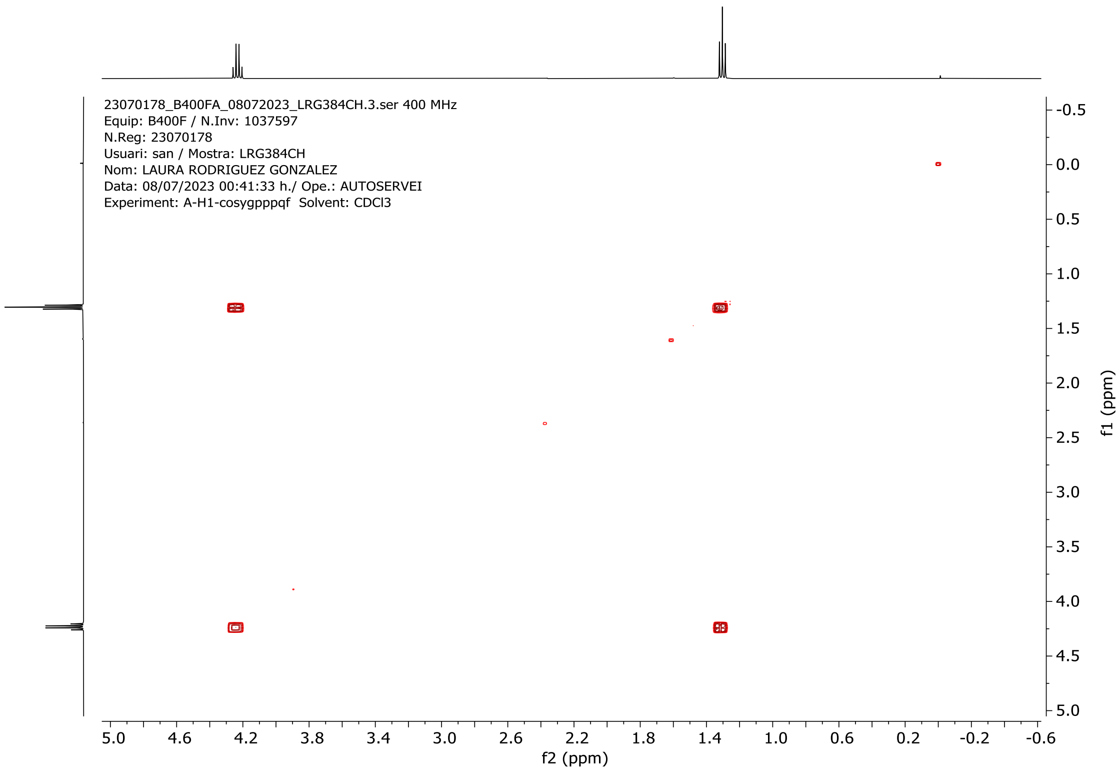

2D-HSQC

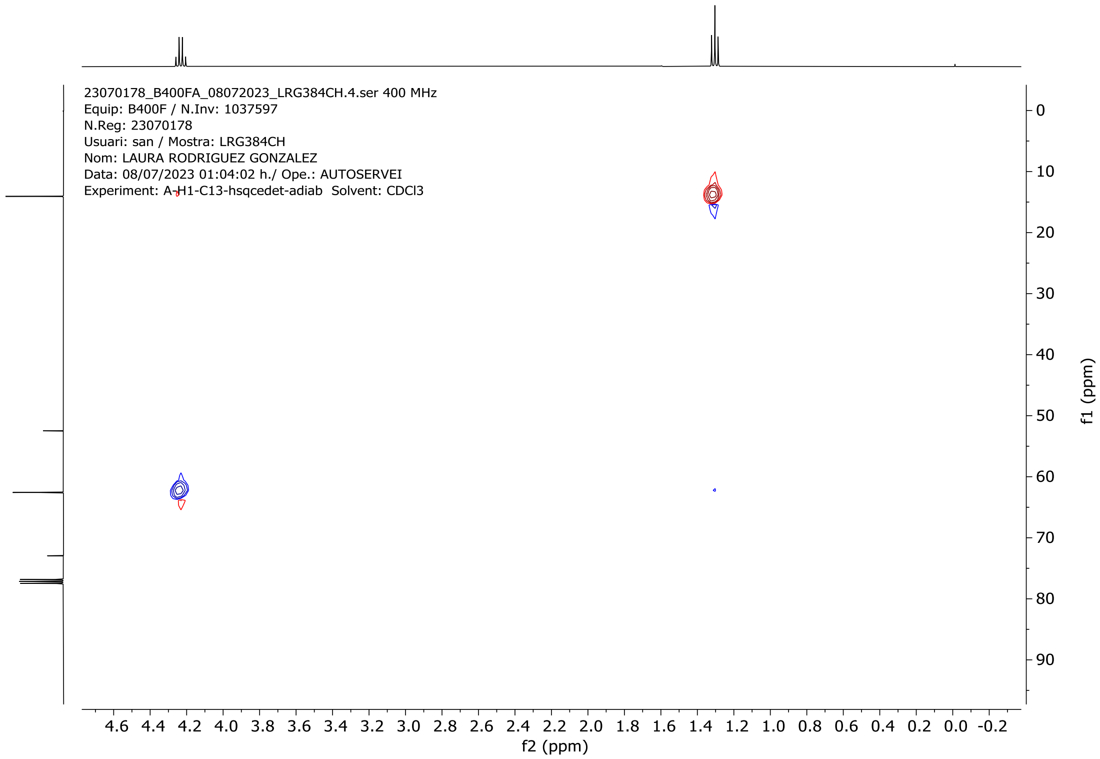

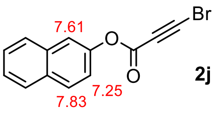

2j

24110250_B400FA_13112024_LRG660T11.1.fid 1H 400 MHz
Equip: B400F / N.Inv: 1037597
N.Reg: 24110250
Usuari: san / Mostra: LRG660T11
Nom: LAURA RODRIGUEZ GONZALEZ
Data: 13/11/2024 10:48:54 h./ Ope.: AUTOSERVEI
Experiment: A-H1-zg30 Solvent: CDCl3

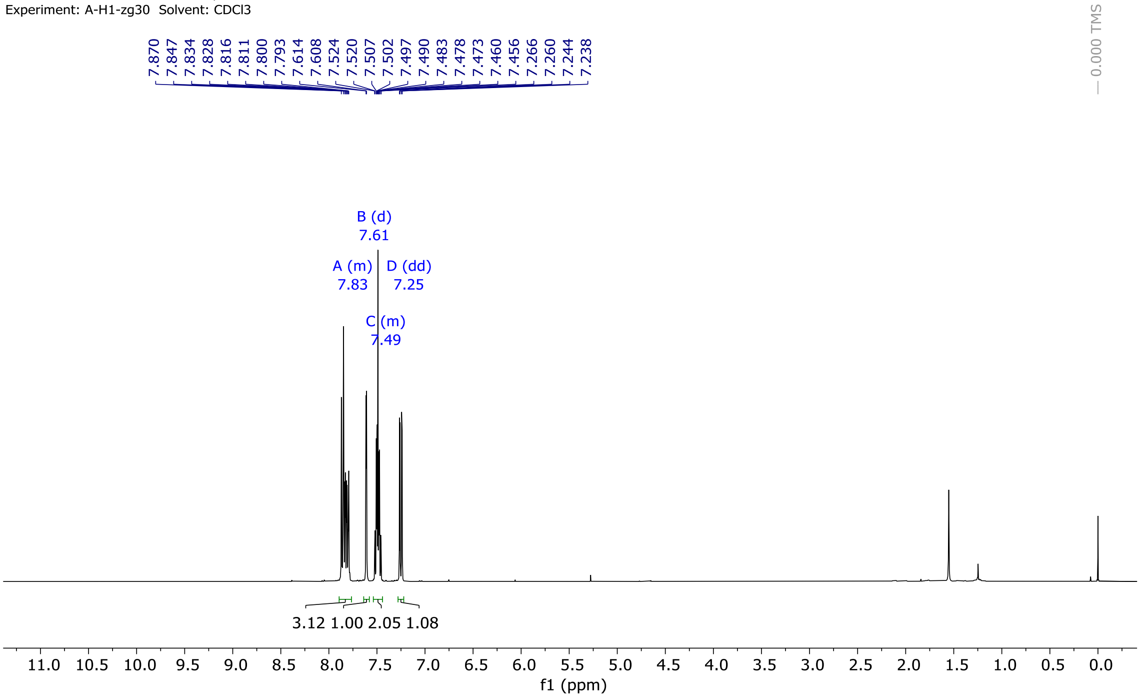

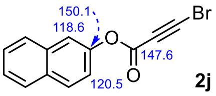

2j

auto-13112024-105914.2.fid 13C{1H} 101 MHz
Equip: B400Q / N.Inv: 1035091
N.Reg: 24110413
Usuari: san / Mostra: LRG660COLQ
Nom: LAURA RODRIGUEZ GONZALEZ
Data: 13/11/2024 21:59:05 h./ Ope.: AUTOSERVEI
Experiment: A_13C-zgpg30 Solvent: CDCl3 Operator:

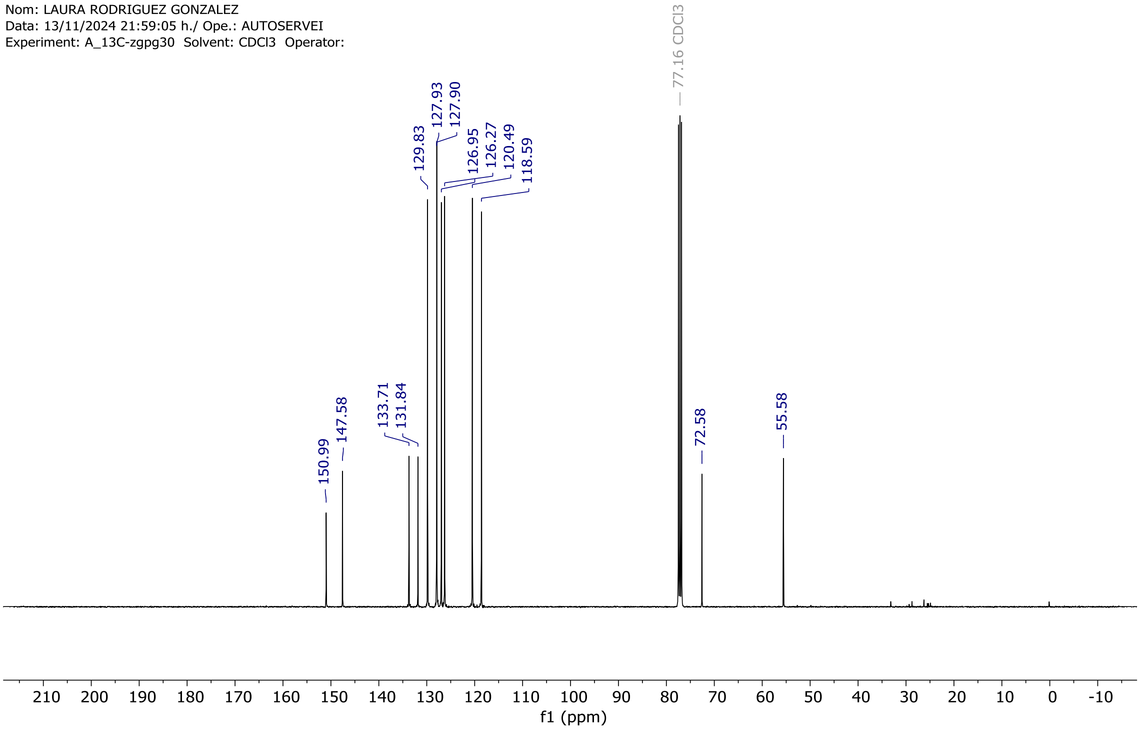

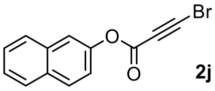

2j

2D-COSY

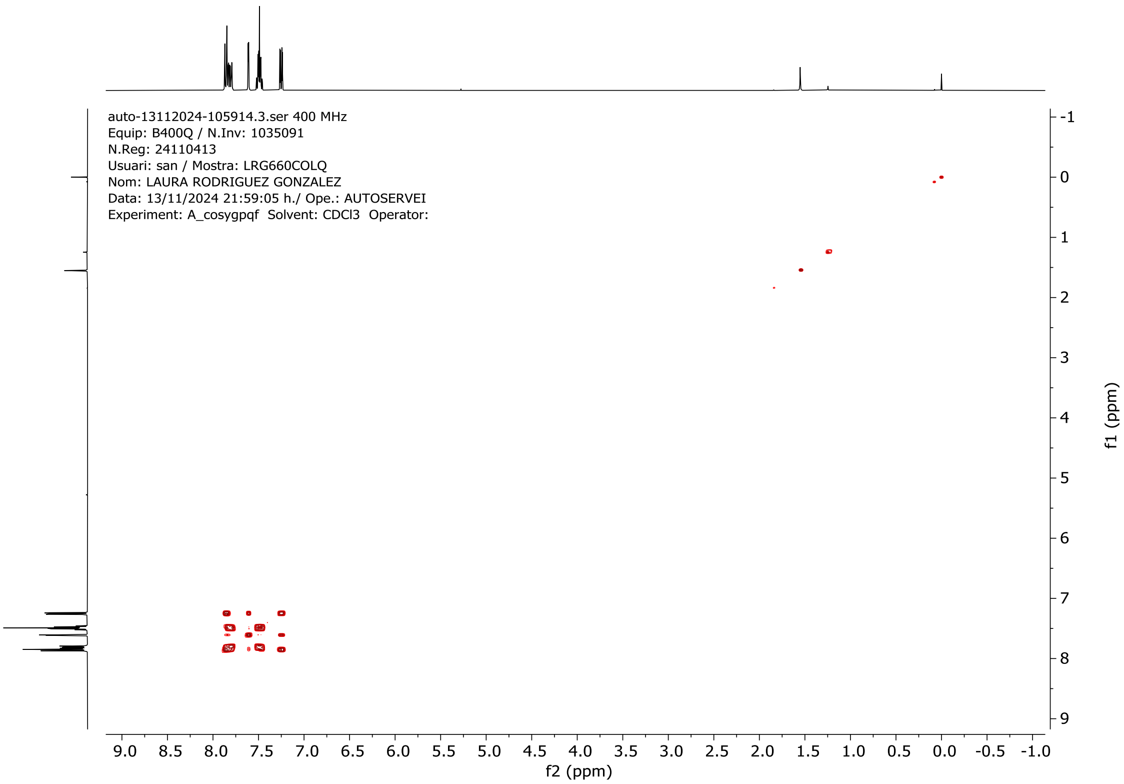

2D-HSQC

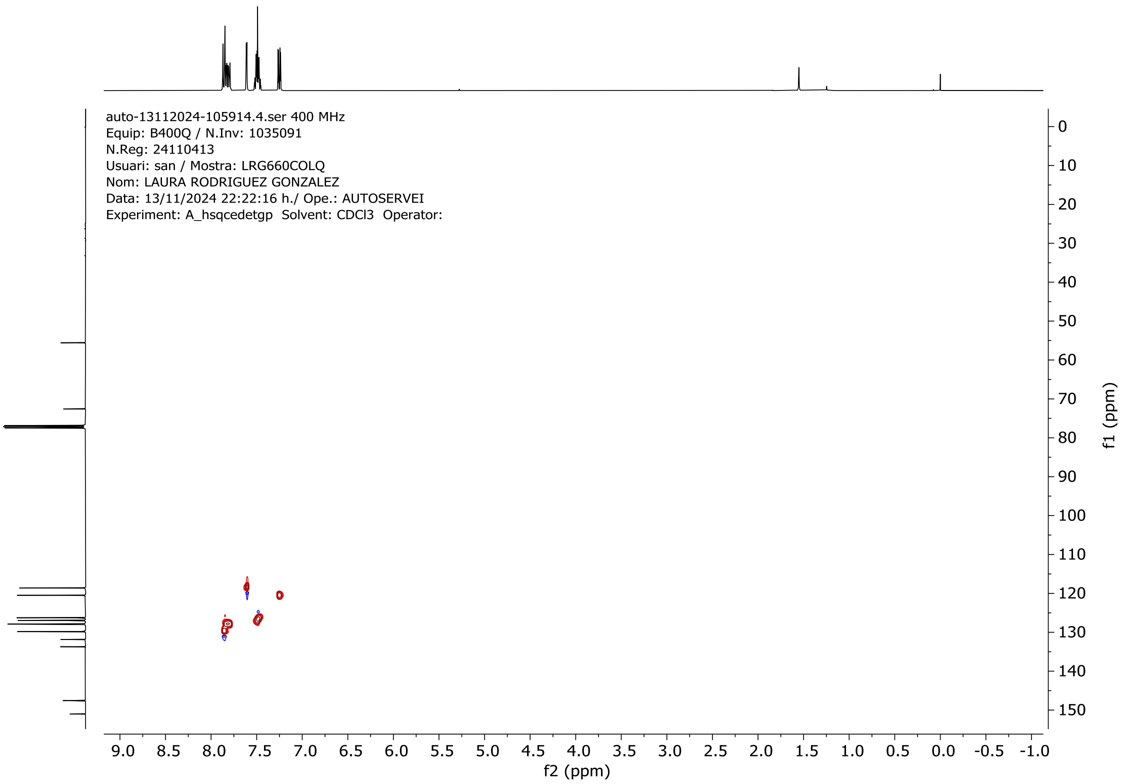

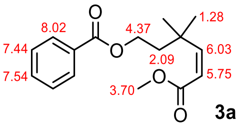

LRG320COLT21T22.1.fid 1H 400 MHz
Equip: B400F / N.Inv: 1037597
N.Reg: 23020561
Usuari: san / Mostra: LRG320COLT21T22
Nom: LAURA RODRIGUEZ GONZALEZ
Data: 14/02/2023 15:43:08 h./ Ope.: AUTOSERVEI
Experiment: A-H1-zg30 Solvent: CDCl3

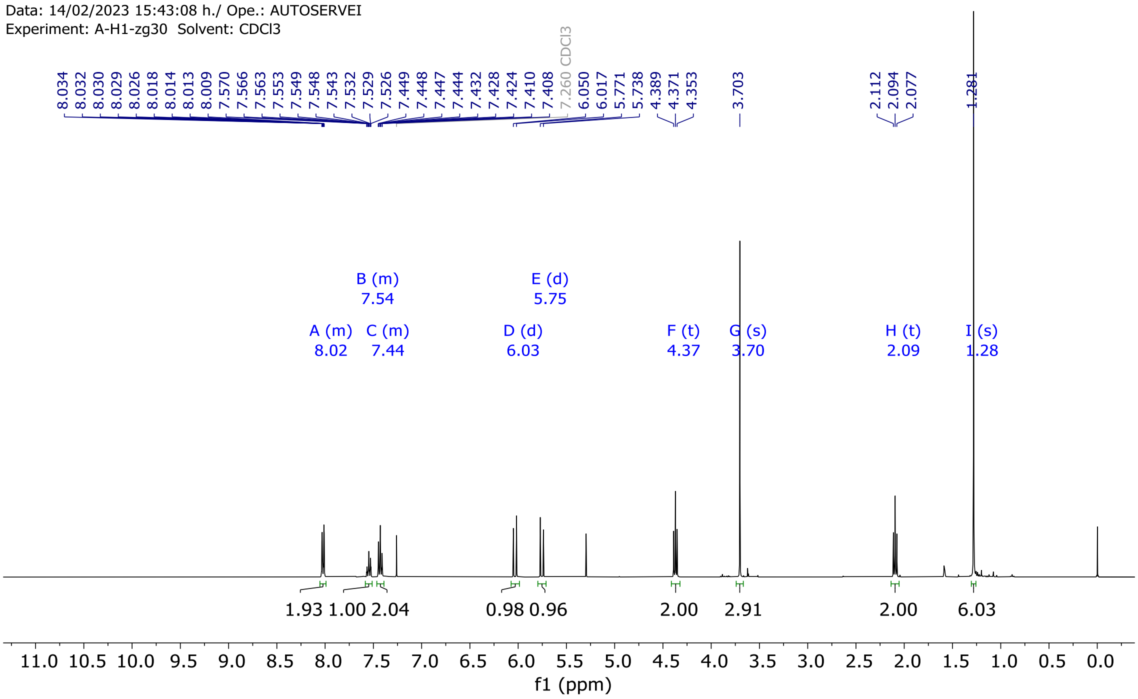

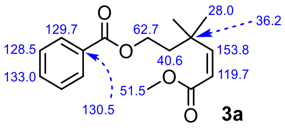

LRG320CH.2.fid 13C{1H} 101 MHz
Equip: B400F / N.Inv: 1037597
N.Reg: 23020581
Usuari: san / Mostra: LRG320CH
Nom: LAURA RODRIGUEZ GONZALEZ
Data: 15/02/2023 03:35:29 h./ Ope.: AUTOSERVEI
Experiment: A-C13-zgpg30 Solvent: CDCl3

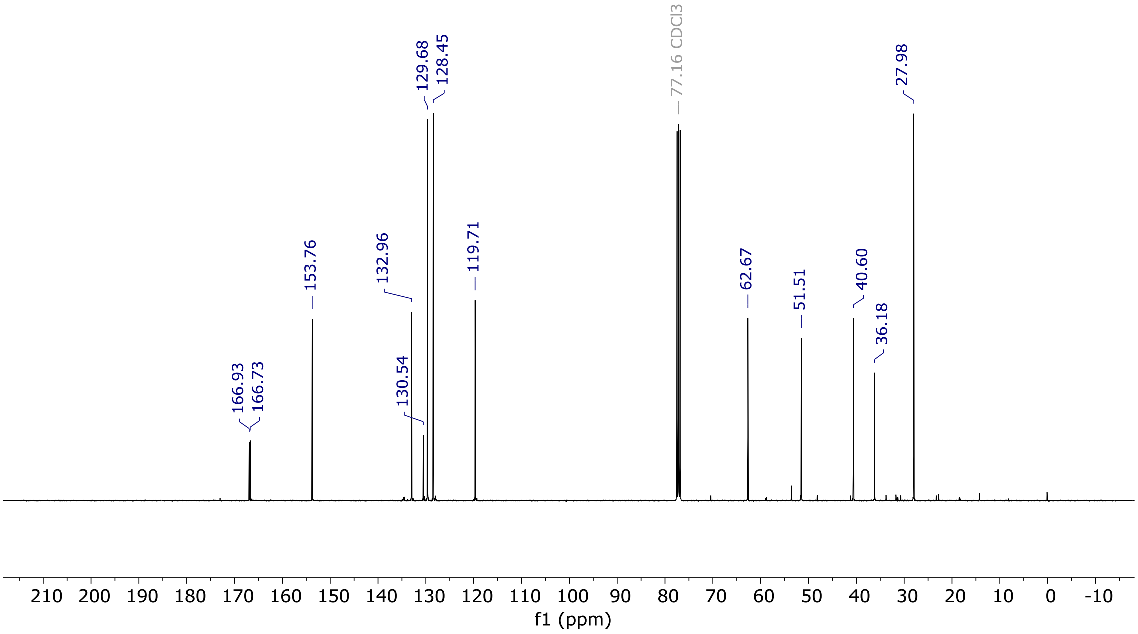

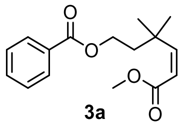

3a

2D-COSY

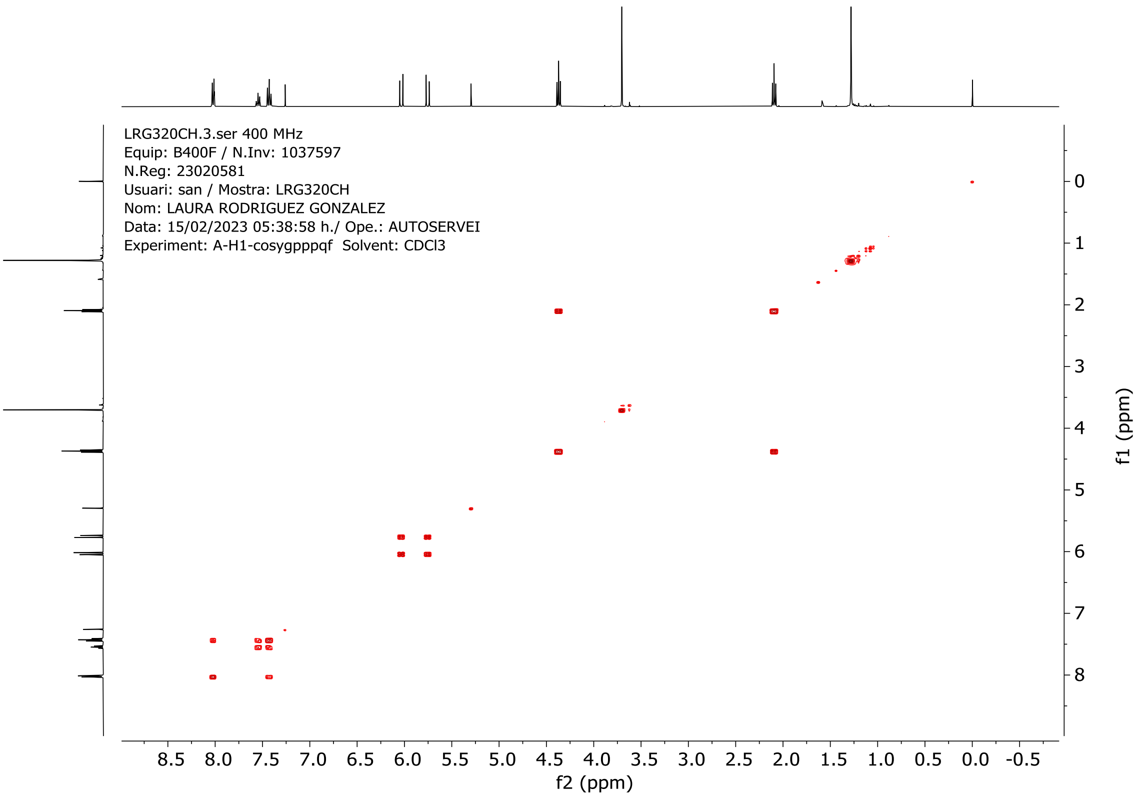

2D-HSQC

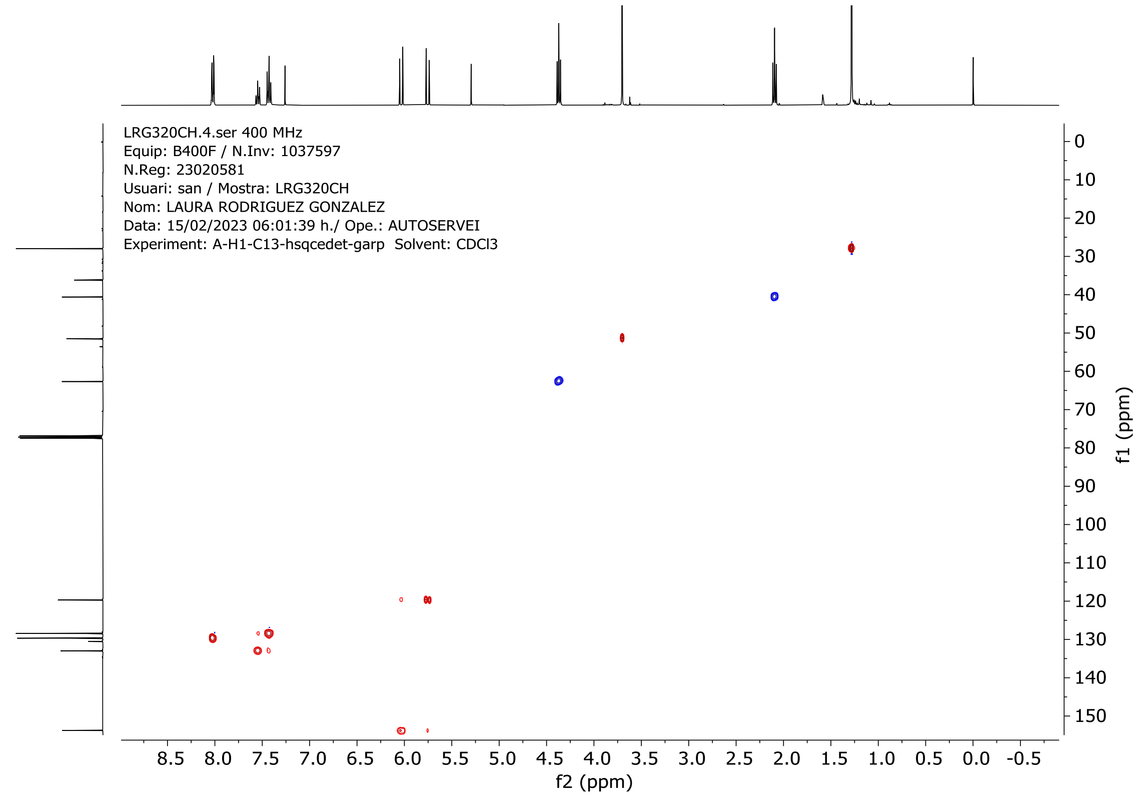

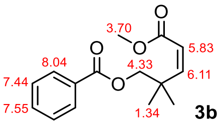

3b

23030506_B400FA_15032023_LRG351T24T27.1.fid 1H 400 MHz
Equip: B400F / N.Inv: 1037597
N.Reg: 23030506
Usuari: san / Mostra: LRG351T24T27
Nom: LAURA RODRIGUEZ GONZALEZ
Data: 15/03/2023 13:33:59 h./ Ope.: AUTOSERVEI
Experiment: A-H1-zg30 Solvent: CDCl3

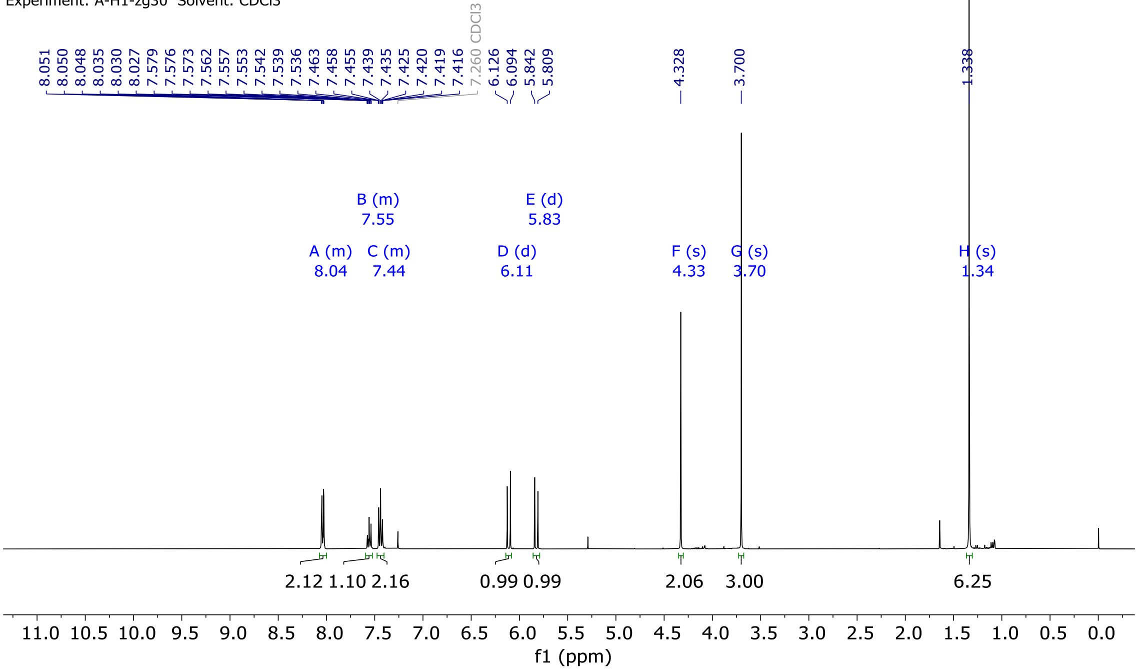

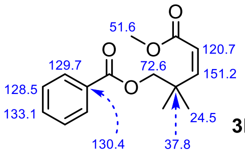

3b

LRG351T24T27CH.2.fid 13C{1H} 101 MHz
Equip: B400F / N.Inv: 1037597
N.Reg: 23030504
Usuari: san / Mostra: LRG350T23
Nom: LAURA RODRIGUEZ GONZALEZ
Data: 16/03/2023 04:47:49 h./ Ope.: AUTOSERVEI
Experiment: A-C13-zgpg30 Solvent: CDCl3

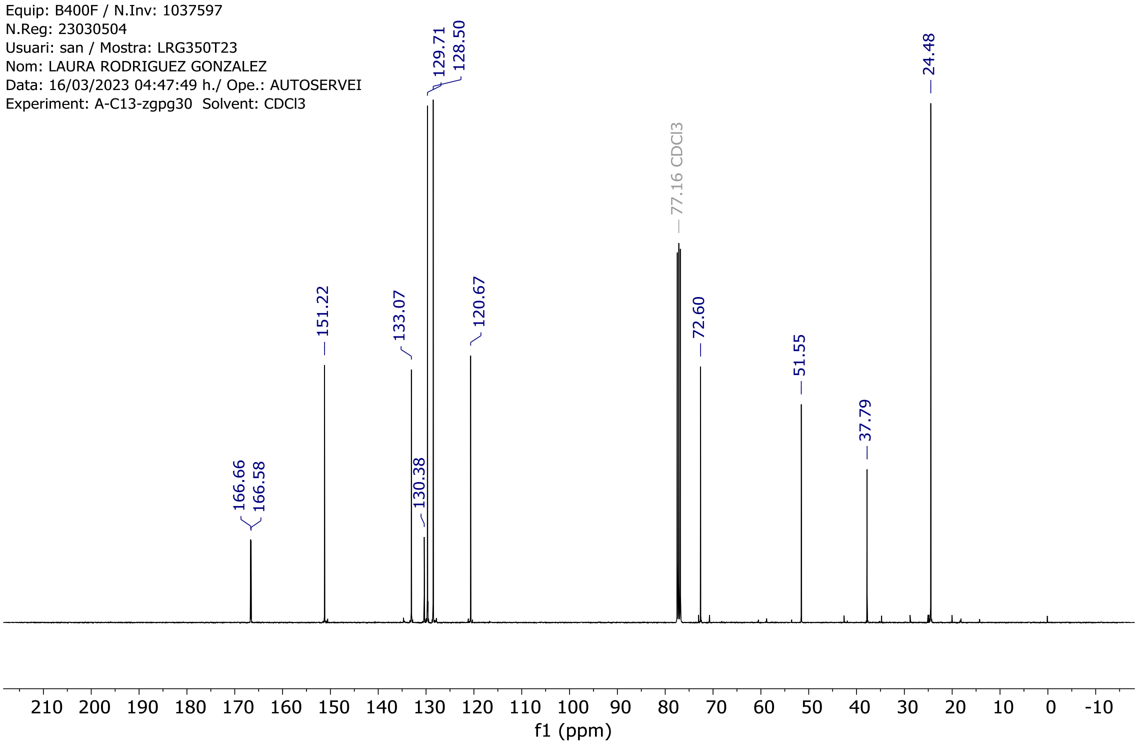

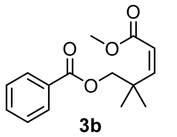

3b

2D-COSY

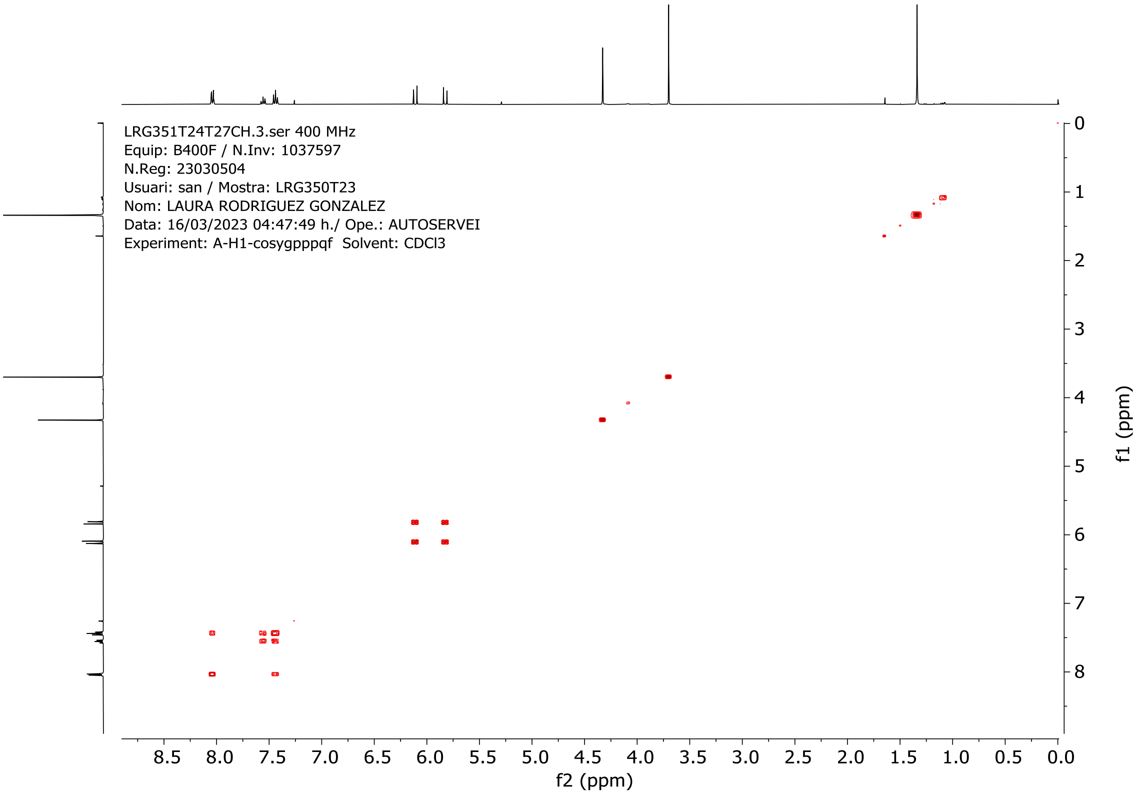

2D-HSQC

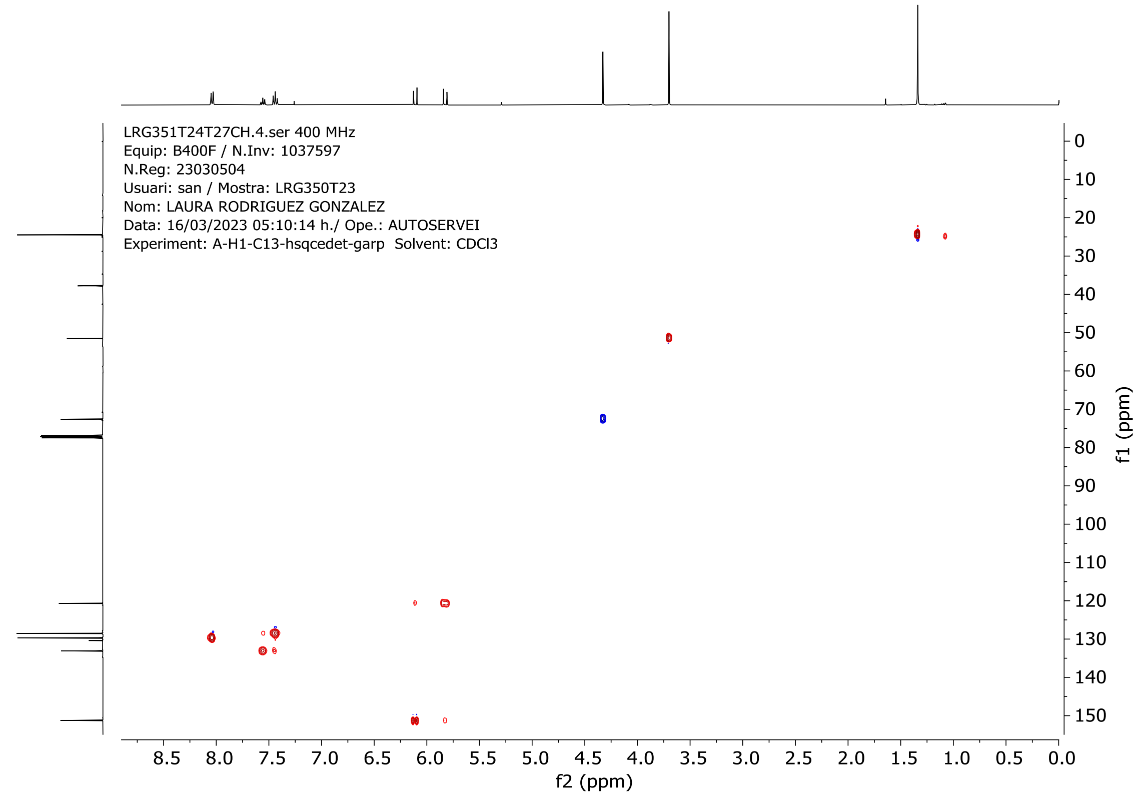

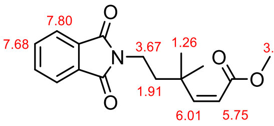

3c

san-1797-2023.1.fid 1H 400 MHz
Equip: B400Q / N.Inv: 1035091
N.Reg: 1797/2023
Usuari: san / Mostra: LRG360CH
Nom: LAURA RODRIGUEZ GONZALEZ
Data: 24/03/2023 14:17:49 h./ Ope.: servei Unitat RMN
Experiment: A_1H-zg30 Solvent: CDCl3 Operator: Victoria Munoz Torrero

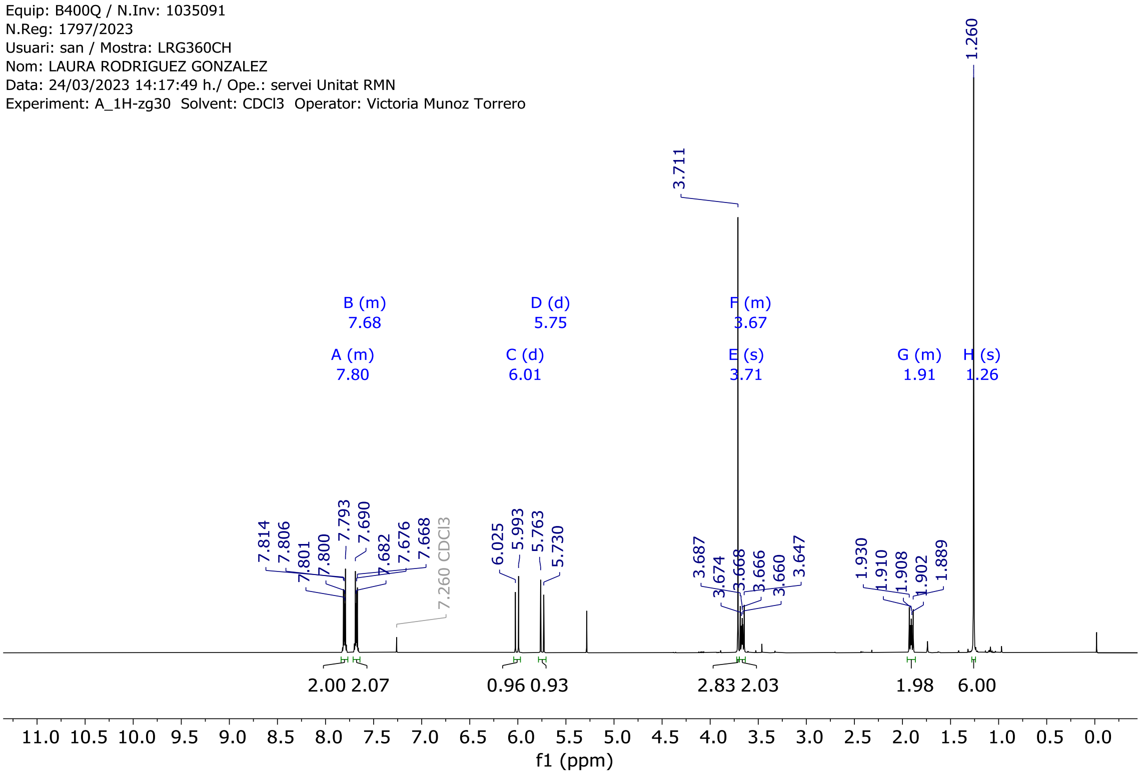

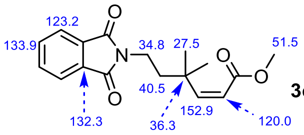

3c

san-1797-2023.5.fid 13C{1H} 101 MHz
Equip: B400Q / N.Inv: 1035091
N.Reg: 1797/2023
Usuari: san / Mostra: LRG360CH
Nom: LAURA RODRIGUEZ GONZALEZ
Data: 24/03/2023 16:20:34 h./ Ope.: servei Unitat RMN
Experiment: A_13C-zgpg30 Solvent: CDCl3 Operator: Victoria Munoz Torrero

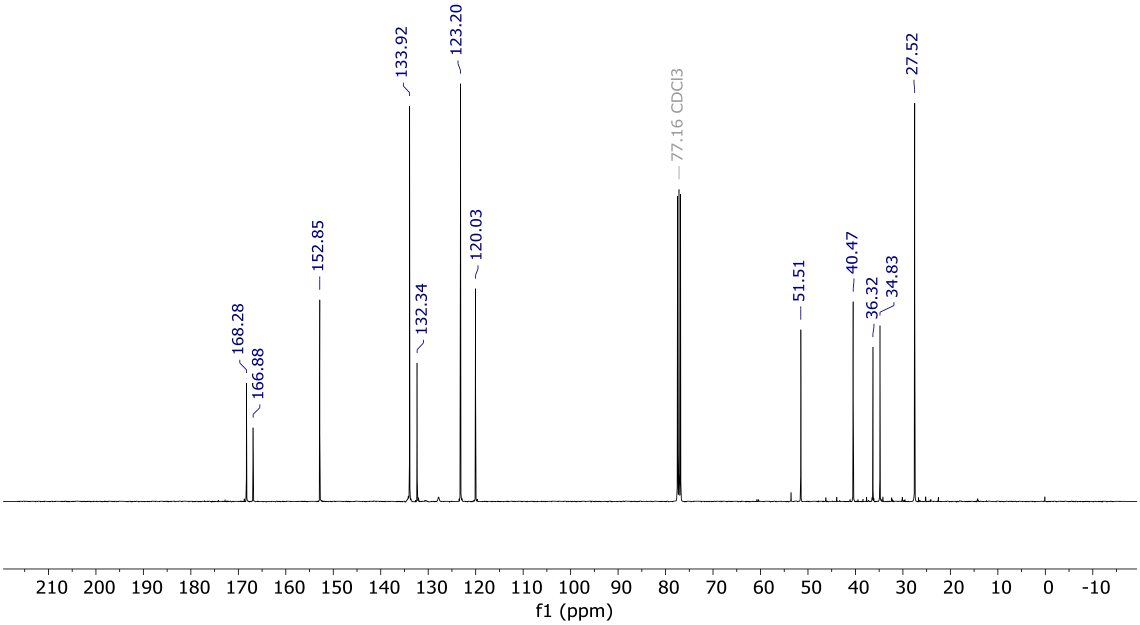

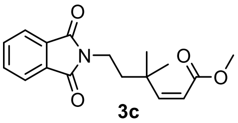

3c

2D-COSY

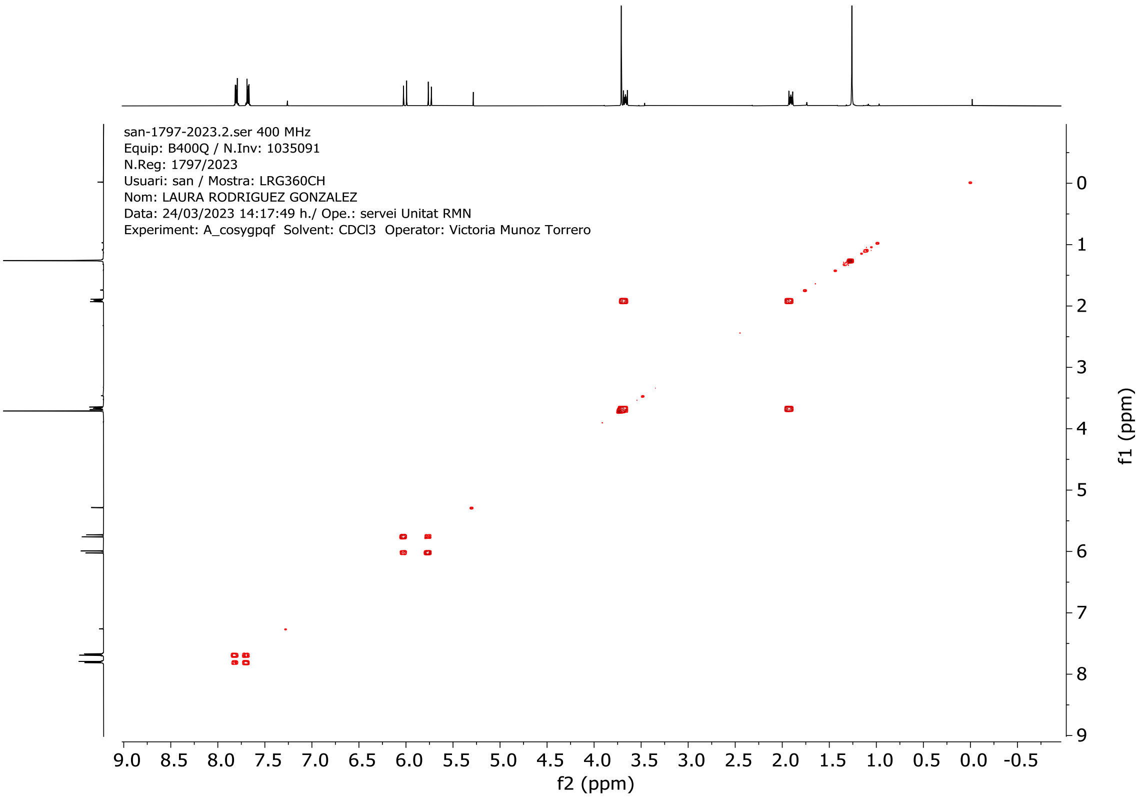

2D-HSQC

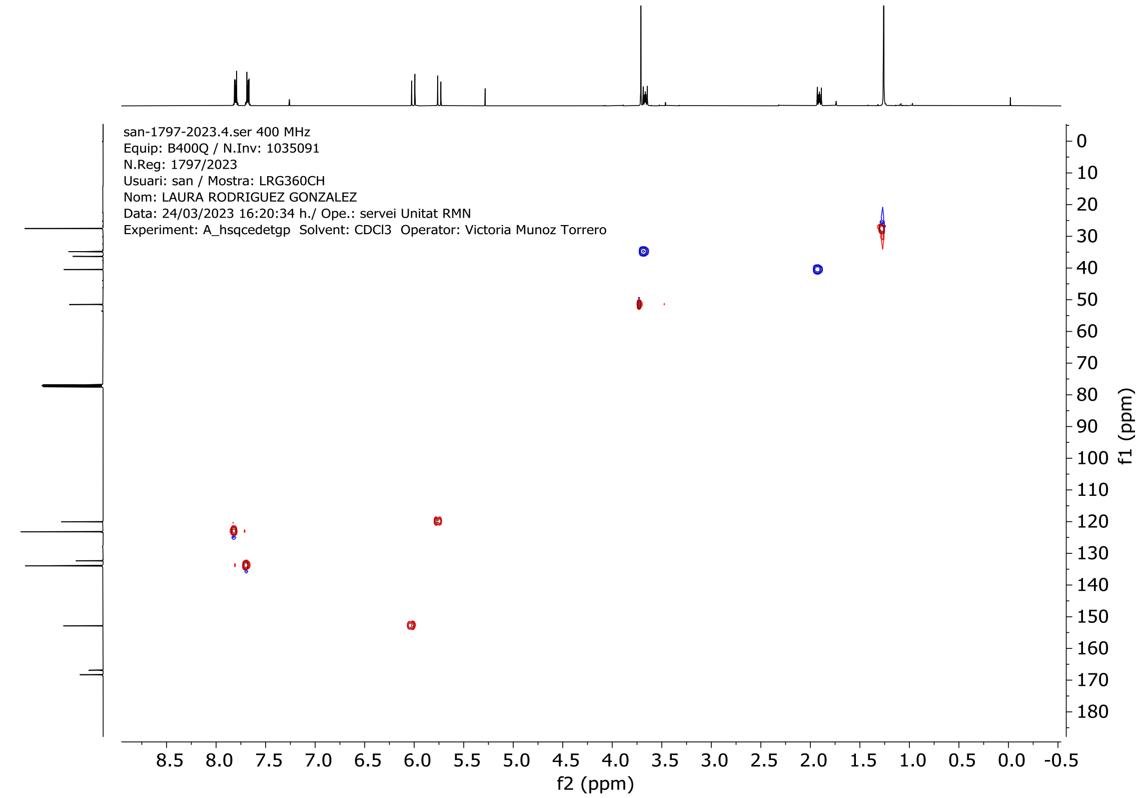

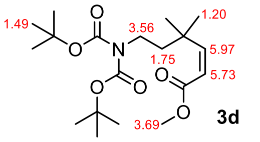

san-1922-2023.1.fid 1H 400 MHz
Equip: B400Q / N.Inv: 1035091
N.Reg: 1922/2023
Usuari: san / Mostra: LRG365CH
Nom: LAURA RODRIGUEZ GONZALEZ
Data: 30/03/2023 03:20:17 h./ Ope.: servei Unitat RMN
Experiment: A_1H-zg30 Solvent: CDCl3 Operator: FRANCISCO CARDENAS LOPEZ

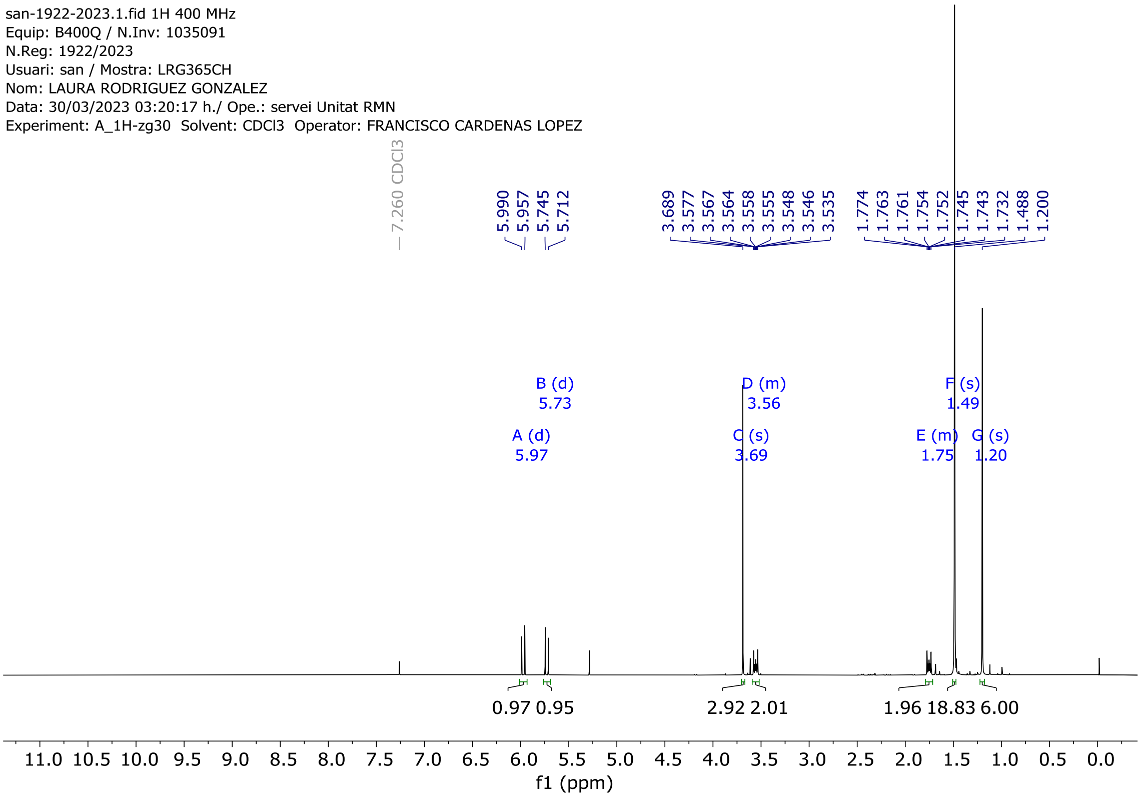

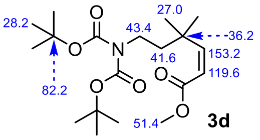

san-1922-2023.2.fid 13C{1H} 101 MHz
Equip: B400Q / N.Inv: 1035091
N.Reg: 1922/2023
Usuari: san / Mostra: LRG365CH
Nom: LAURA RODRIGUEZ GONZALEZ
Data: 30/03/2023 03:20:17 h./ Ope.: servei Unitat RMN
Experiment: A_13C-zgpg30 Solvent: CDCl3 Operator: FRANCISCO CARDENAS LOPEZ

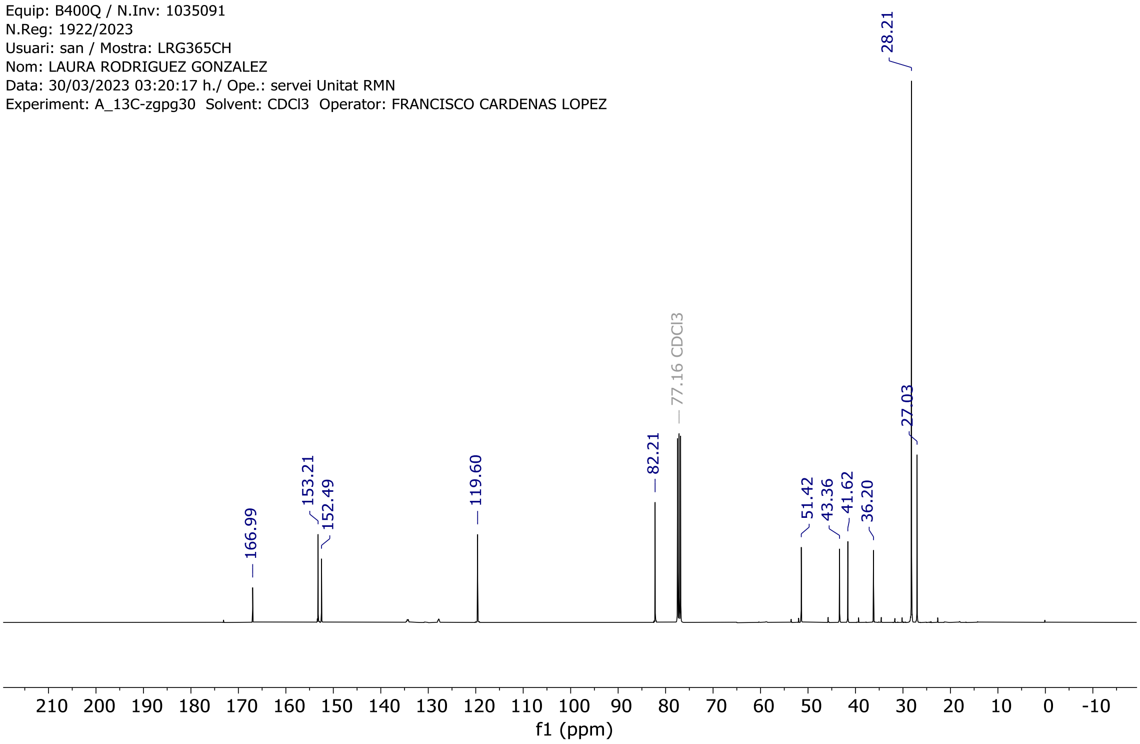

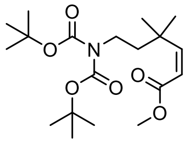

3d

2D-COSY

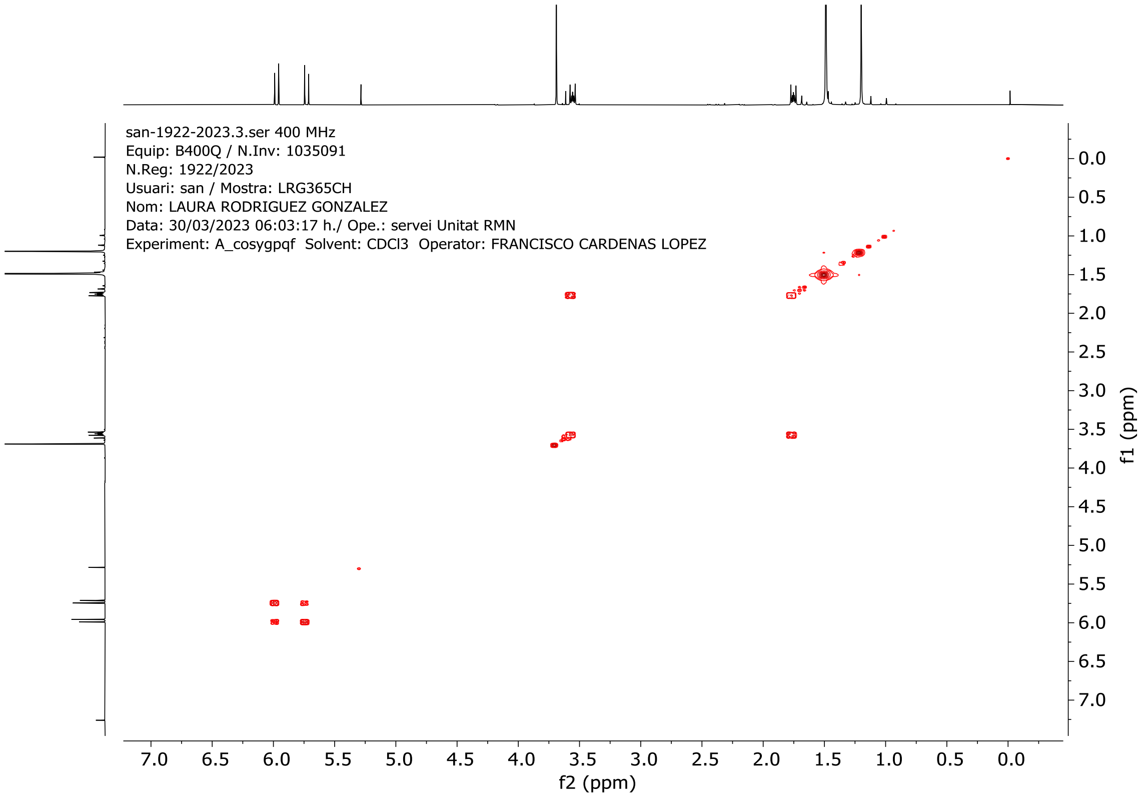

2D-HSQC

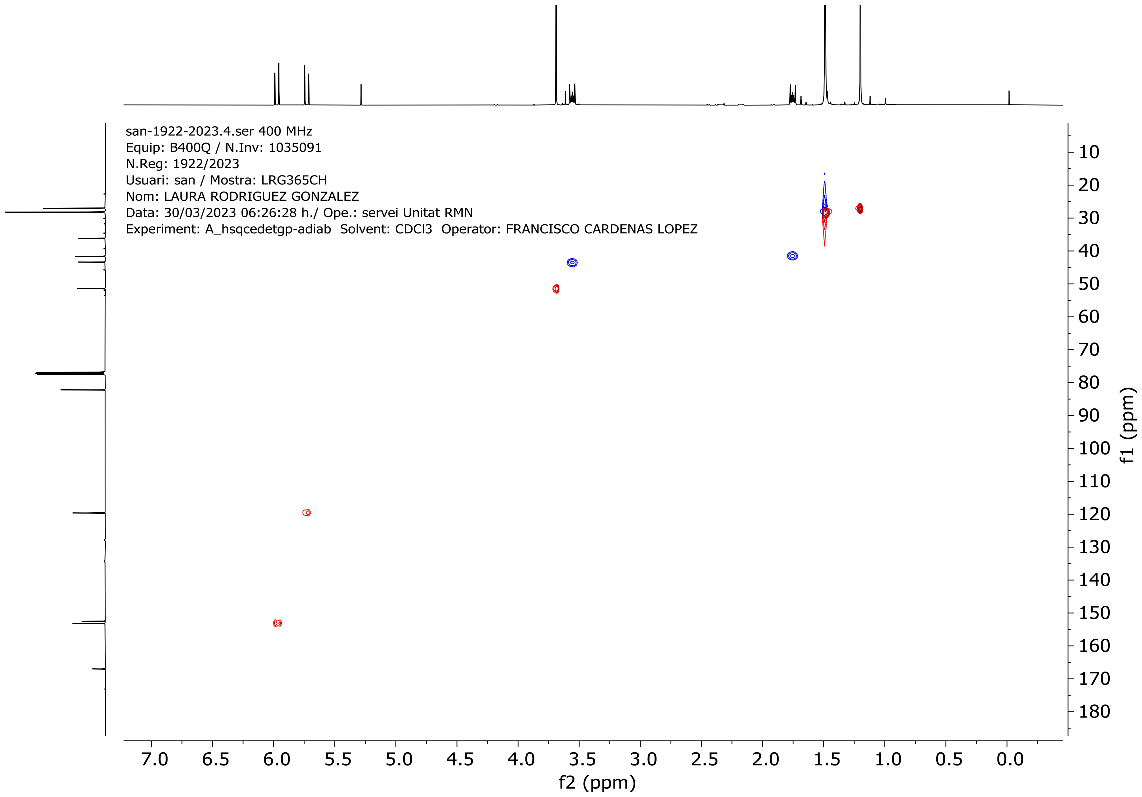

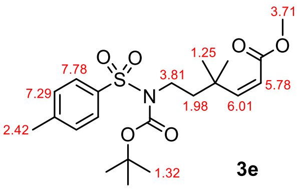

3e

23040662_B400FA_27042023_LRG378CH.1.fid 1H 400 MHz
Equip: B400F / N.Inv: 1037597
N.Reg: 23040662
Usuari: san / Mostra: LRG378CH
Nom: LAURA RODRIGUEZ GONZALEZ
Data: 27/04/2023 13:37:28 h./ Ope.: AUTOSERVEI
Experiment: A-H1-zg30 Solvent: CDCl3

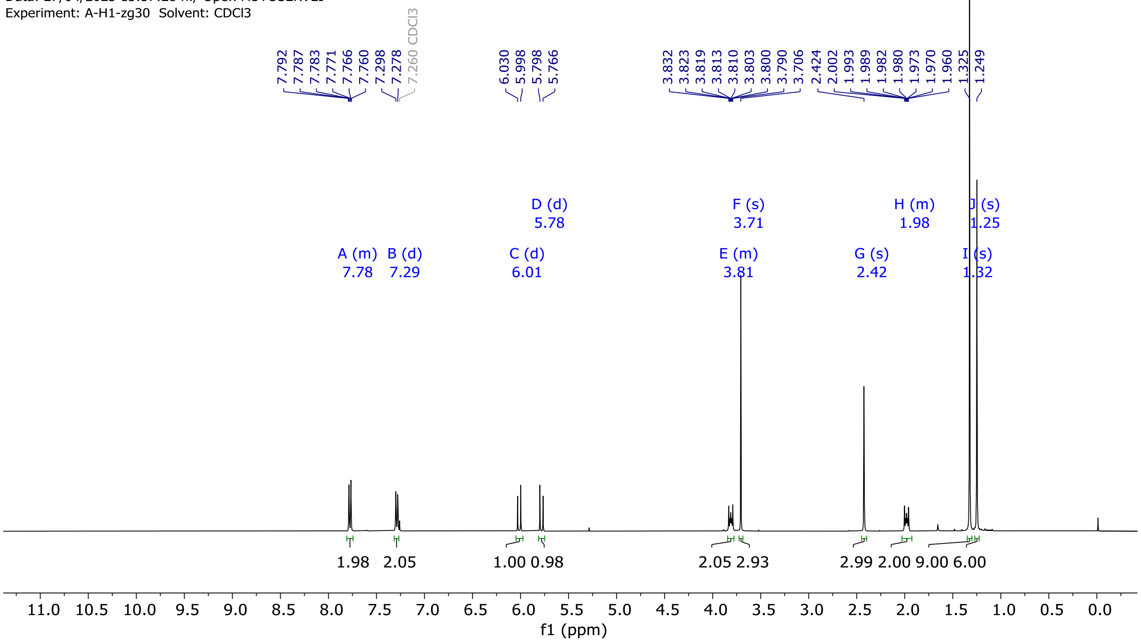

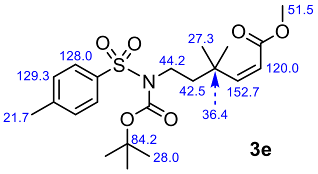

3e

23040662_B400FA_28042023_LRG378CH.2.fid 13C{1H} 101 MHz
Equip: B400F / N.Inv: 1037597
N.Reg: 23040662
Usuari: san / Mostra: LRG378CH
Nom: LAURA RODRIGUEZ GONZALEZ
Data: 28/04/2023 00:33:21 h./ Ope.: AUTOSERVEI
Experiment: A-C13-zgpg30 Solvent: CDCl3

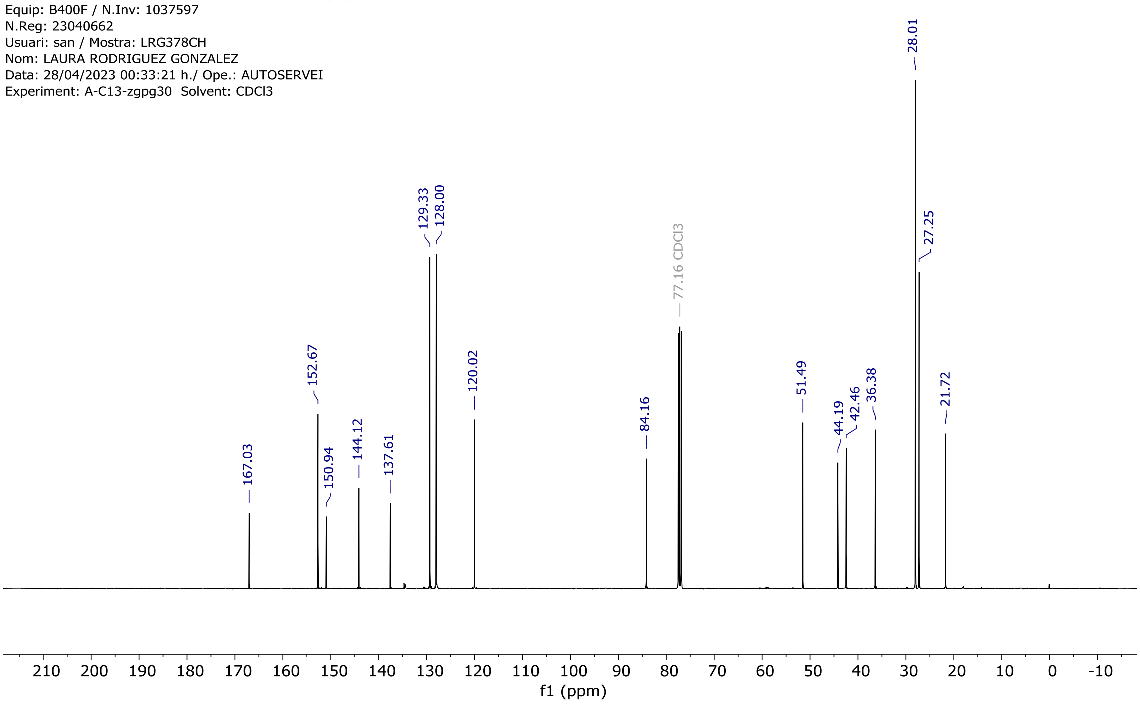

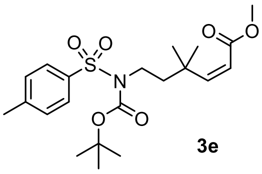

3e

2D-COSY

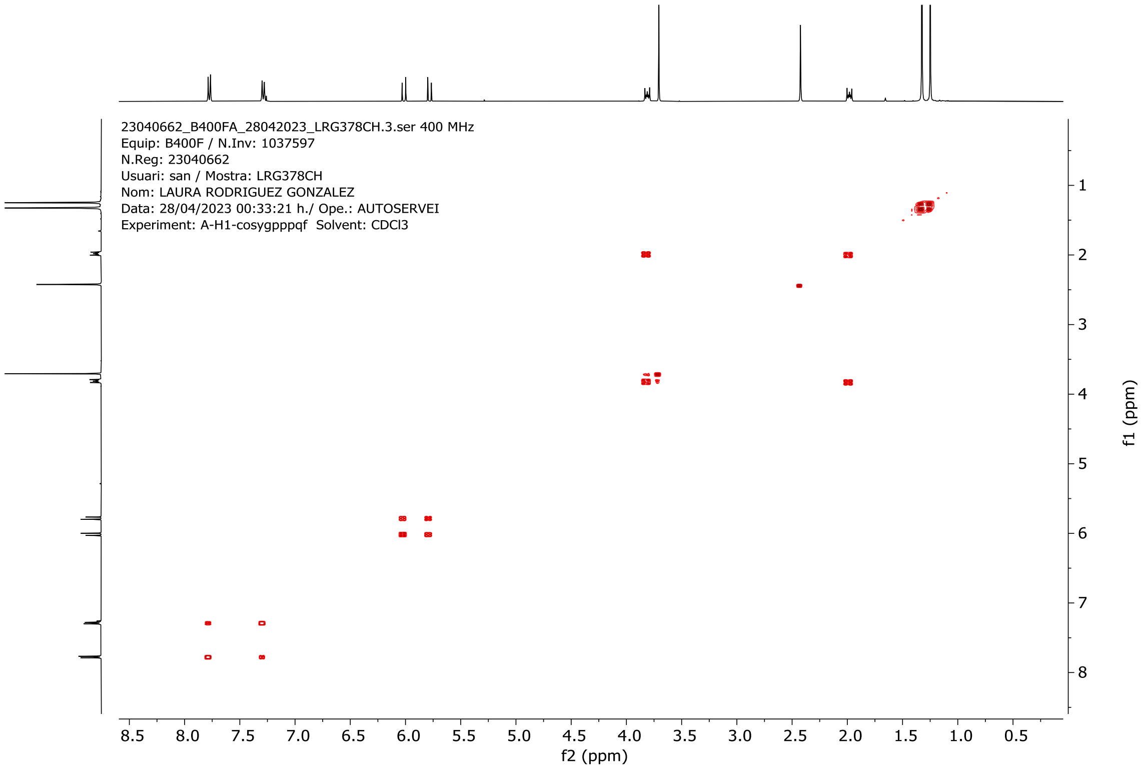

2D-HSQC

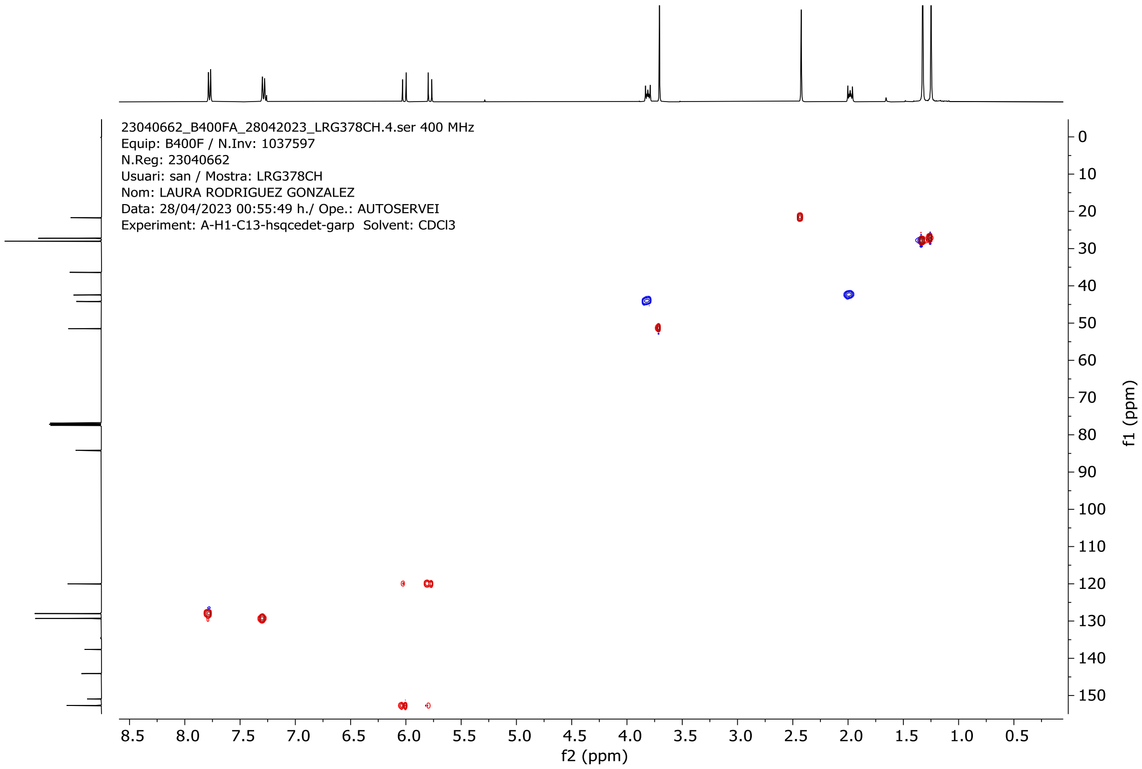

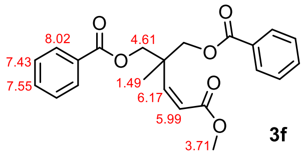

3f

23040428_B400FA_19042023_LRG374RECT32.1.fid 1H 400 MHz
Equip: B400F / N.Inv: 1037597
N.Reg: 23040428
Usuari: san / Mostra: LRG374RECT32
Nom: LAURA RODRIGUEZ GONZALEZ
Data: 19/04/2023 16:14:22 h./ Ope.: AUTOSERVEI
Experiment: A-H1-zg30 Solvent: CDCl3

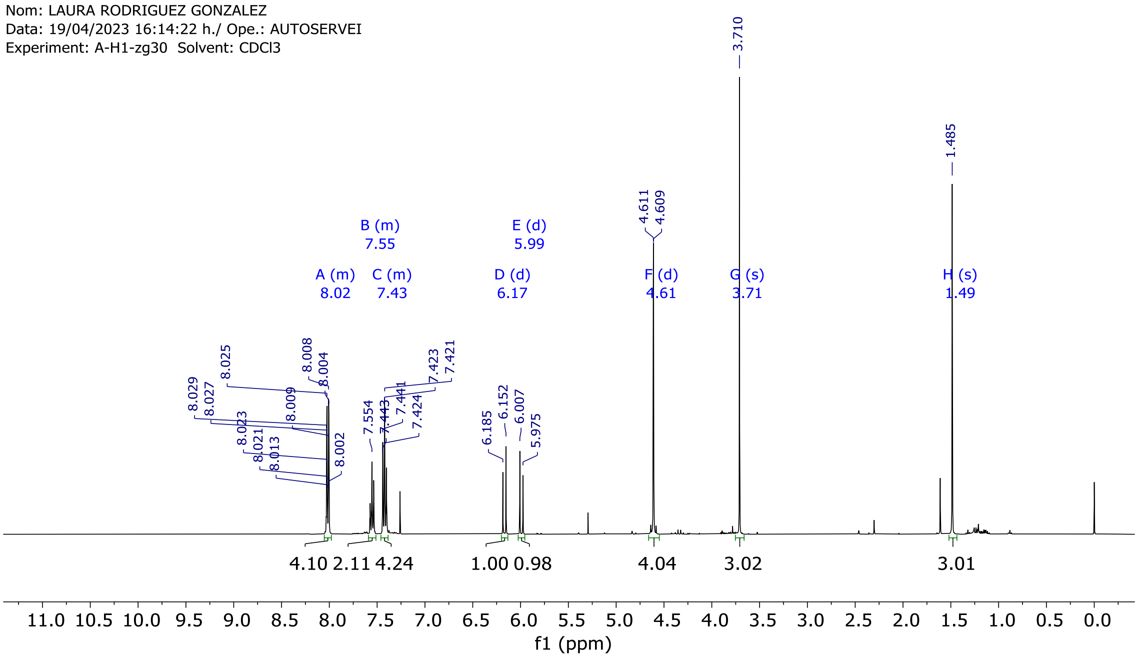

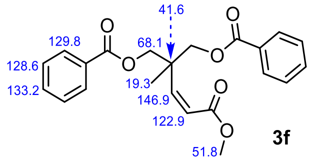

3f

san-2256-2023.4.fid 13C{1H} 101 MHz
Equip: B400Q / N.Inv: 1035091
N.Reg: 2256/2023
Usuari: san / Mostra: LRG374CH
Nom: LAURA RODRIGUEZ GONZALEZ
Data: 20/04/2023 22:49:36 h./ Ope.: servei Unitat RMN
Experiment: A_13C-zgpg30 Solvent: CDCl3 Operator: M ANTONIA MOLINS

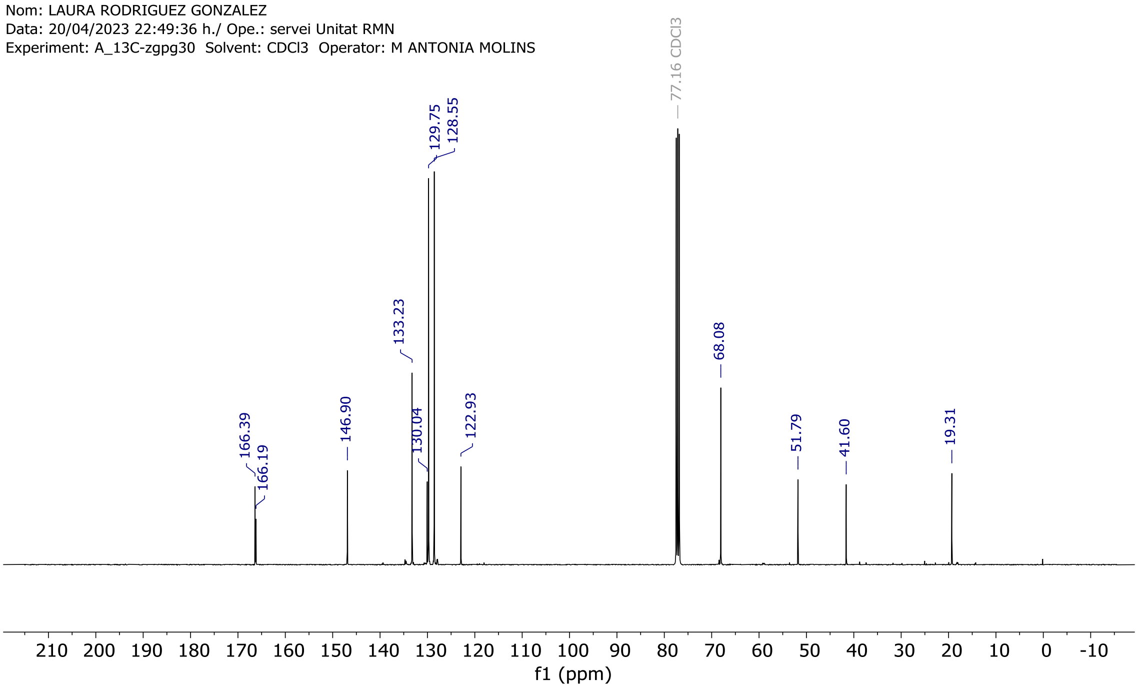

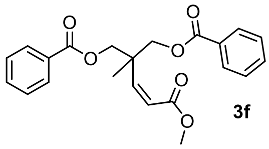

3f

2D-COSY

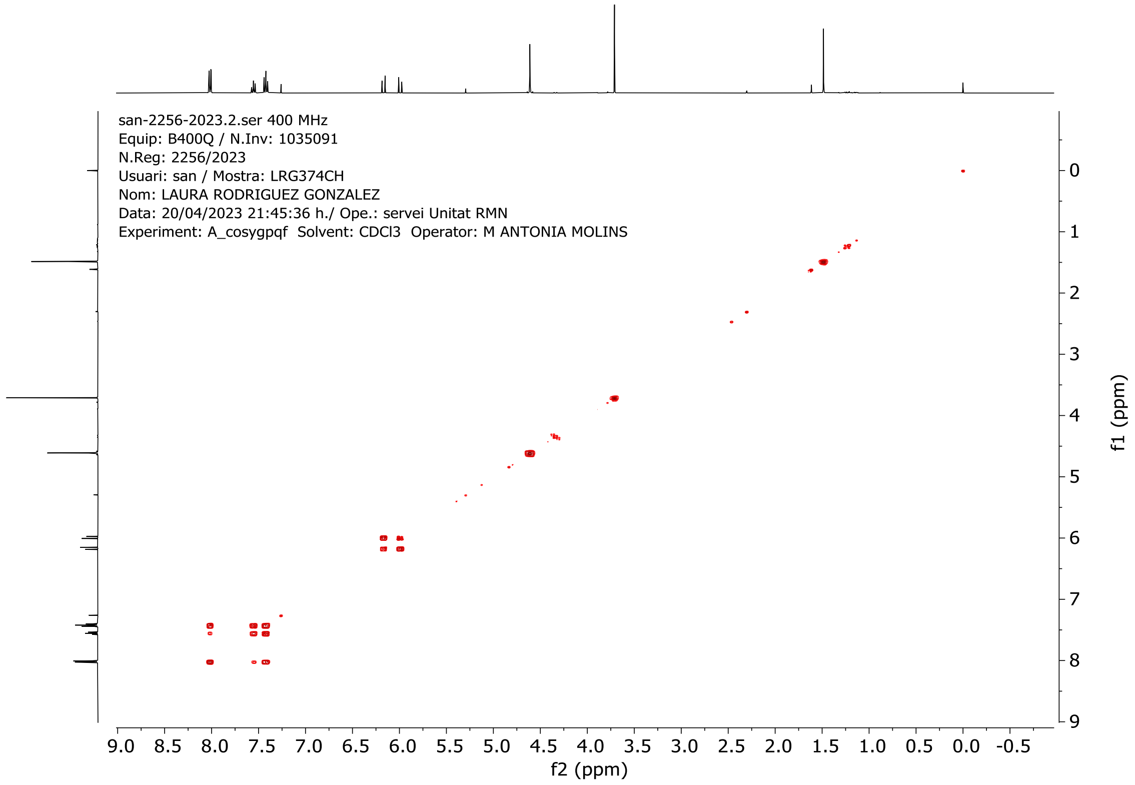

2D-HSQC

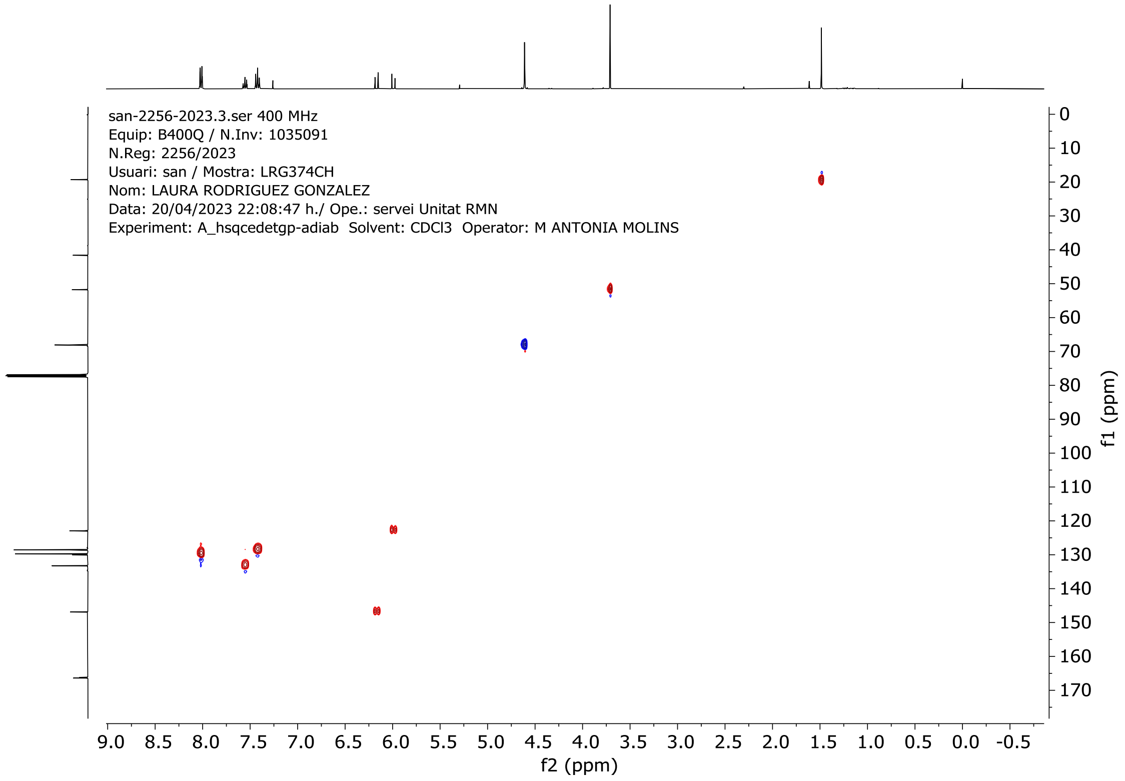

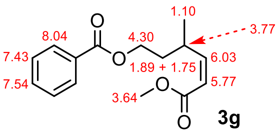

3g

23030566_B400FA_17032023_LRG353CH.1.fid 1H 400 MHz
Equip: B400F / N.Inv: 1037597
N.Reg: 23030566
Usuari: san / Mostra: LRG353CH
Nom: LAURA RODRIGUEZ GONZALEZ
Data: 16/03/2023 15:39:19 h./ Ope.: AUTOSERVEI
Experiment: A-H1-zg30 Solvent: CDCl3

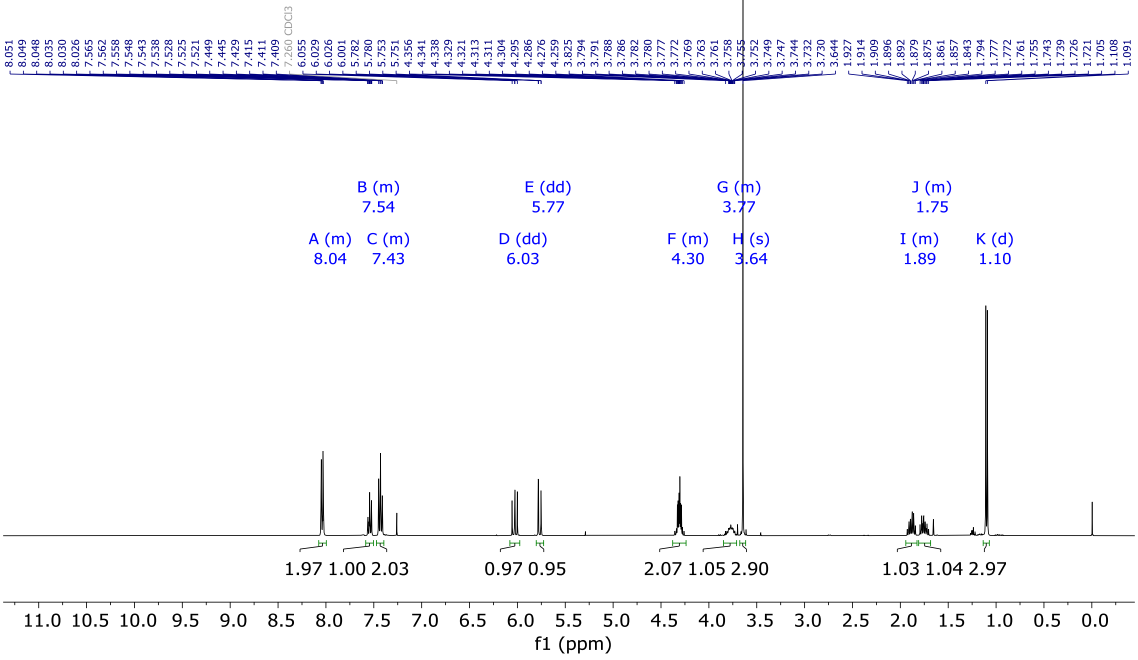

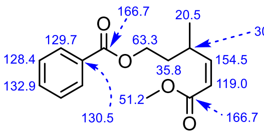

3g

23030566_B400FA_17032023_LRG353CH.2.fid 13C{1H} 101 MHz
Equip: B400F / N.Inv: 1037597
N.Reg: 23030566
Usuari: san / Mostra: LRG353CH
Nom: LAURA RODRIGUEZ GONZALEZ
Data: 17/03/2023 04:15:06 h./ Ope.: AUTOSERVEI
Experiment: A-C13-zgpg30 Solvent: CDCl3

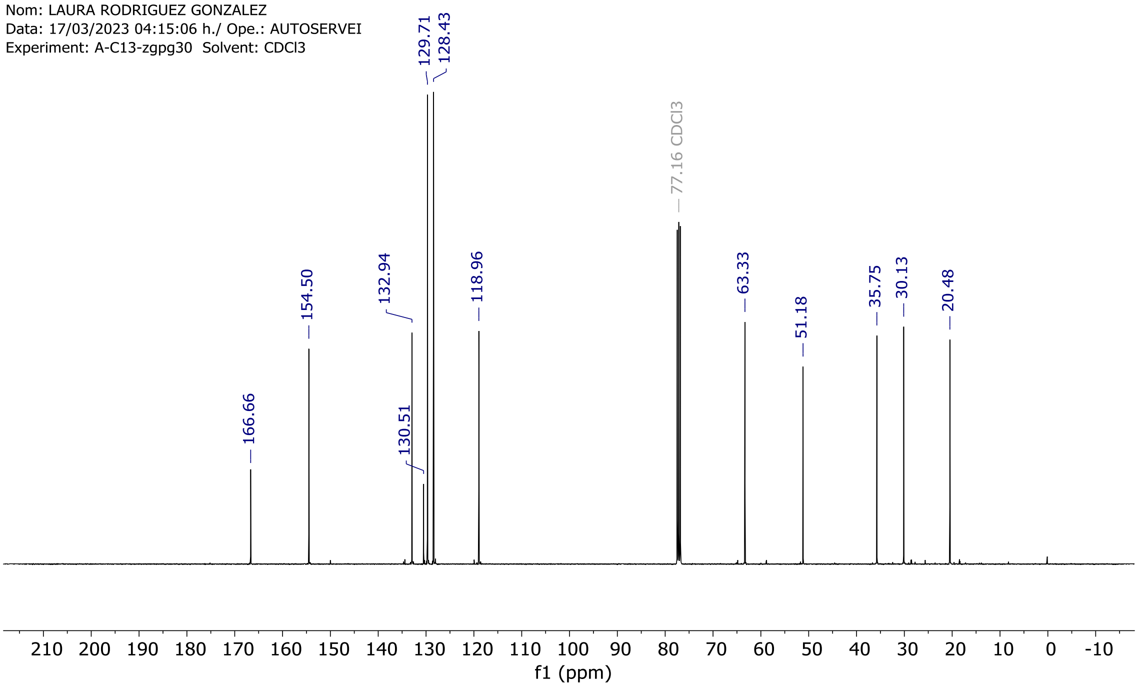

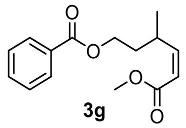

3g

2D-COSY

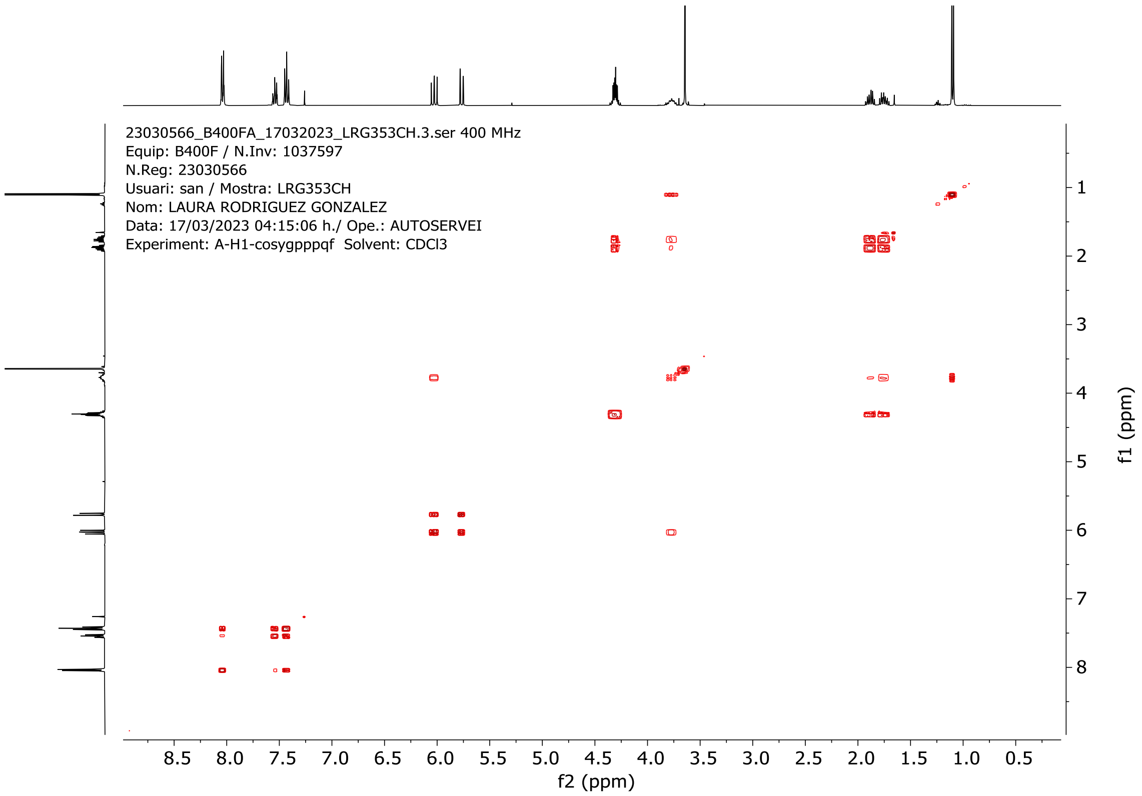

2D-HSQC

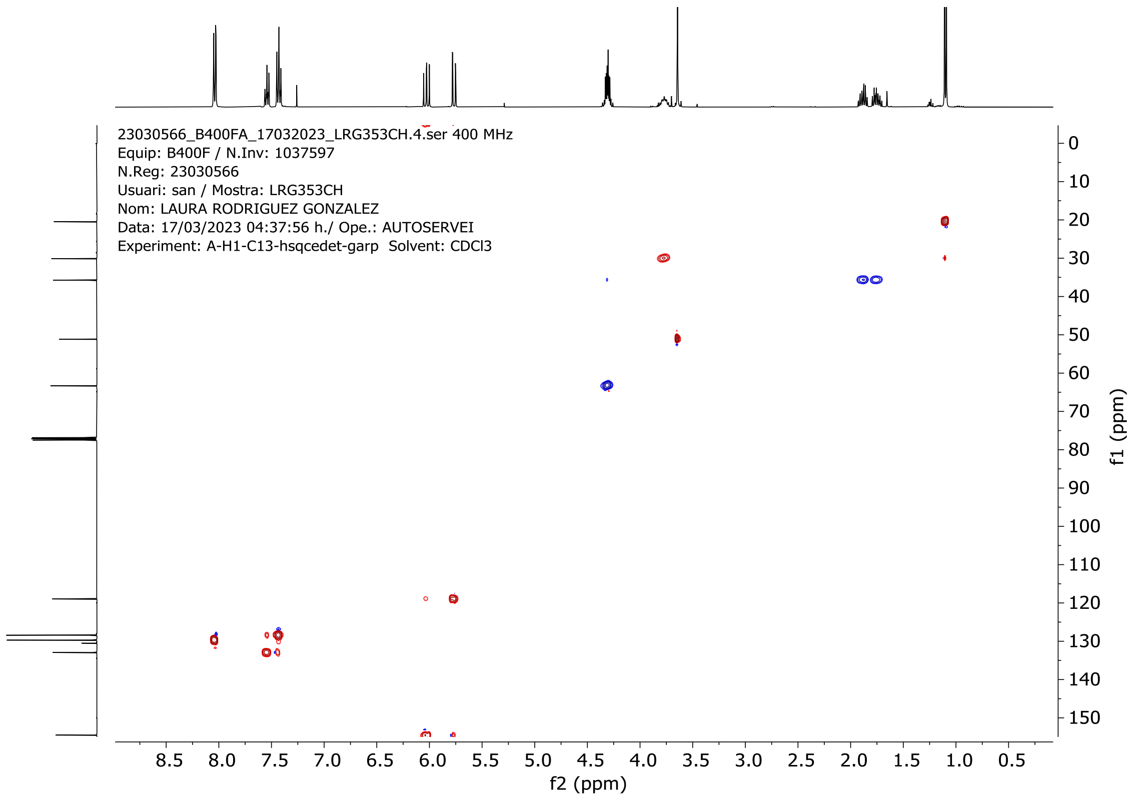

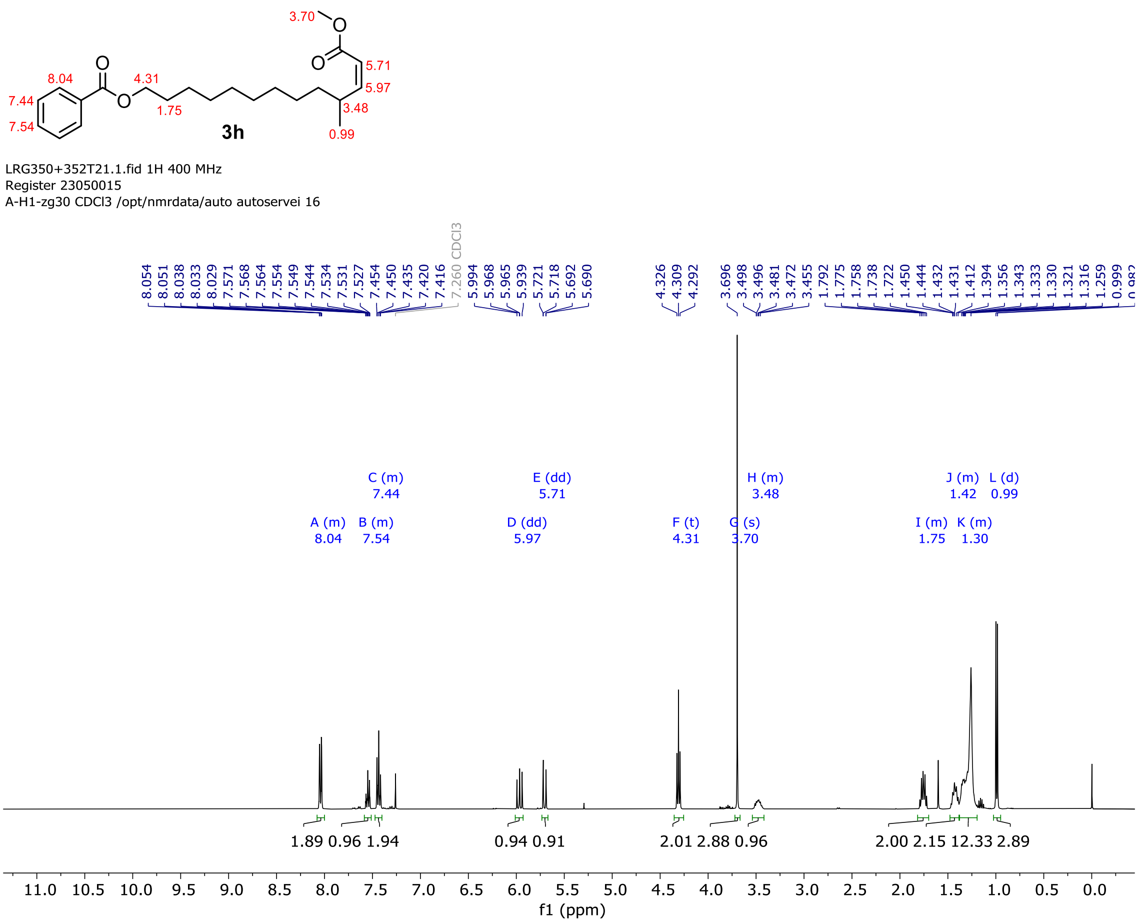
3.70
5.71
5.97
3.48
0.99
8.04
4.31
7.44
7.54
1.75
3h
LRG350+352T21.1.fid 1H 400 MHz
Register 23050015
A-H1-zg30 CDCl3 /opt/nmrdata/auto autoservei 16
A (m) 8.04
B (m) 7.54
C (m) 7.44
D (dd) 5.97
E (dd) 5.71
F (t) 4.31
G (s) 3.70
H (m) 3.48
I (m) 1.75
J (m) 1.42
K (m) 1.30
L (d) 0.99
1.89 0.96 1.94
0.94 0.91
2.01 2.88 0.96
2.00 2.15 12.33 2.89
f1 (ppm)

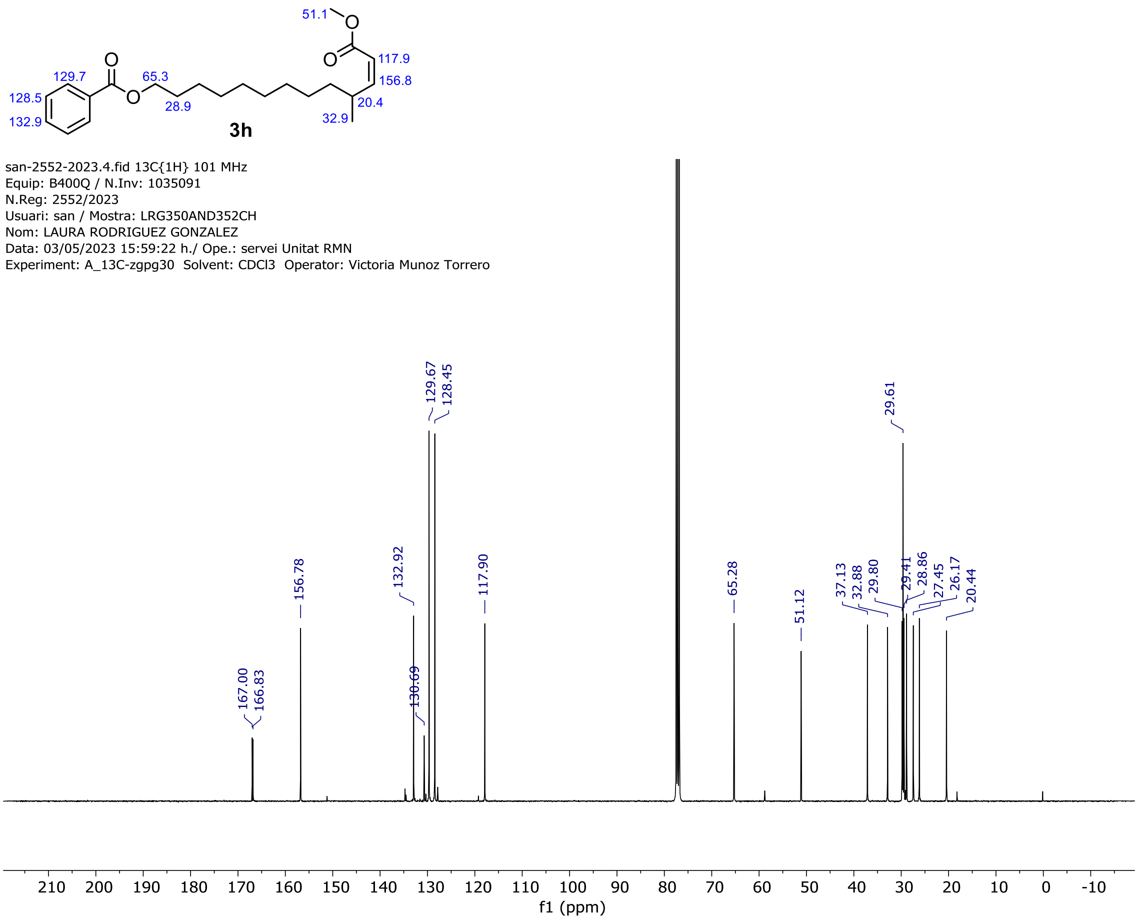
51.1
117.9
156.8
129.7
65.3
128.5
28.9
20.4
132.9
32.9
3h
san-2552-2023.4.fid 13C{1H} 101 MHz
Equip: B400Q / N.Inv: 1035091
N.Reg: 2552/2023
Usuari: san / Mostra: LRG350AND352CH
Nom: LAURA RODRIGUEZ GONZALEZ
Data: 03/05/2023 15:59:22 h./ Ope.: servei Unitat RMN
Experiment: A_13C-zgpg30 Solvent: CDCl3 Operator: Victoria Munoz Torrero
167.00
166.83
156.78
132.92
130.69
129.67
128.45
117.90
65.28
51.12
37.13
32.88
29.80
29.61
29.41
28.86
27.45
26.17
20.44
f1 (ppm)

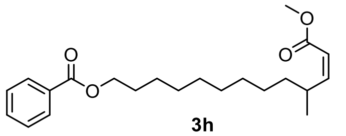

3h

2D-COSY

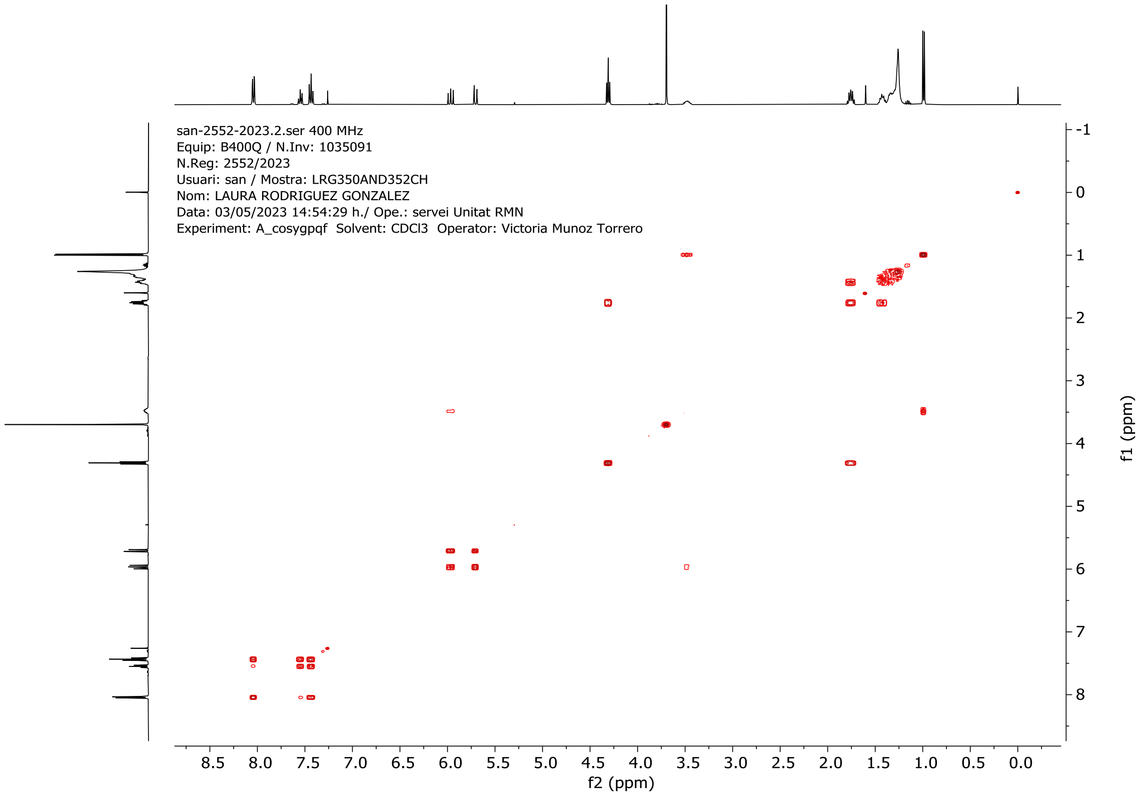

2D-HSQC

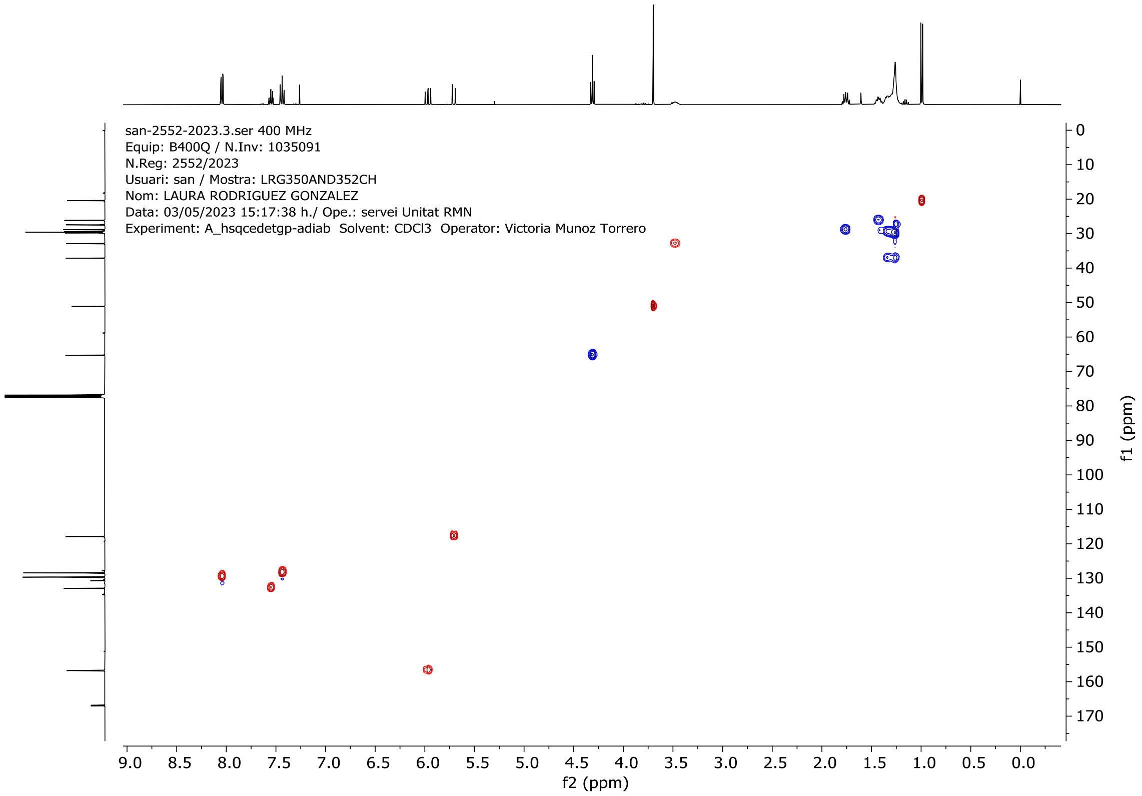

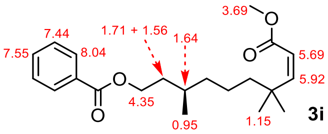

san-1891-2023.1.fid 1H 400 MHz
Equip: B400Q / N.Inv: 1035091
N.Reg: 1891/2023
Usuari: san / Mostra: LRG364CH
Nom: LAURA RODRIGUEZ GONZALEZ
Data: 29/03/2023 17:19:06 h./ Ope.: servei Unitat RMN
Experiment: A_1H-zg30 Solvent: CDCl3 Operator: FRANCISCO CARDENAS LOPEZ

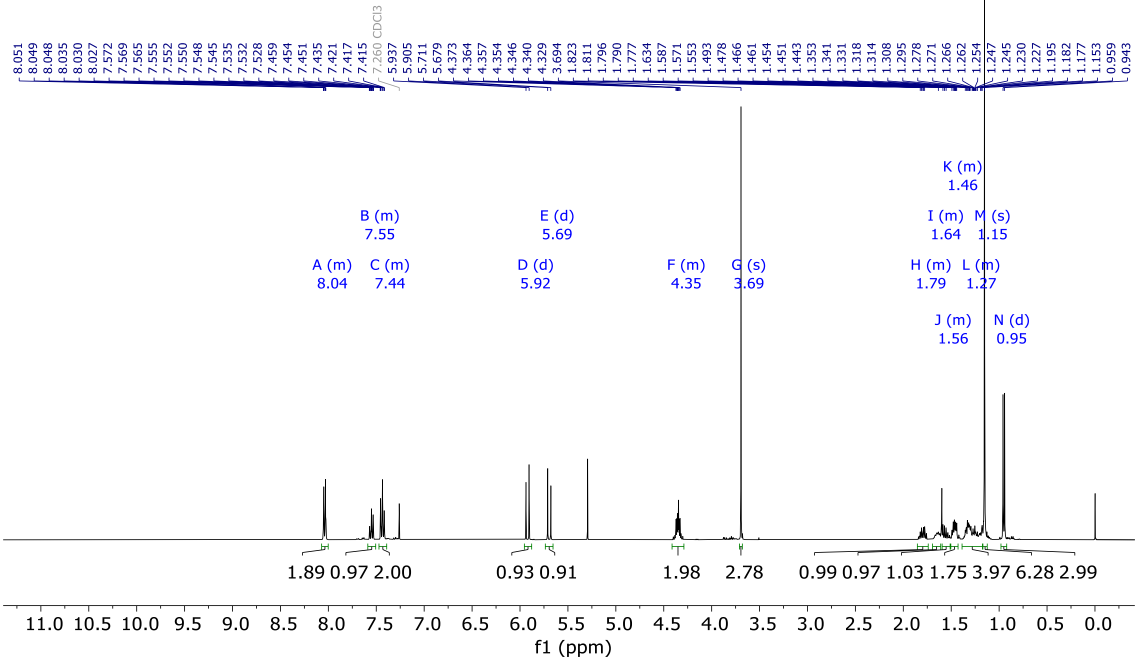

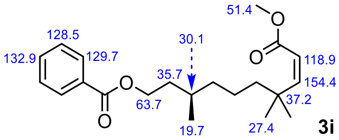

san-1891-2023.2.fid 13C{1H} 101 MHz
Equip: B400Q / N.Inv: 1035091
N.Reg: 1891/2023
Usuari: san / Mostra: LRG364CH
Nom: LAURA RODRIGUEZ GONZALEZ
Data: 29/03/2023 17:19:06 h./ Ope.: servei Unitat RMN
Experiment: A_13C-zgpg30 Solvent: CDCl3 Operator: FRANCISCO CARDENAS LOPEZ

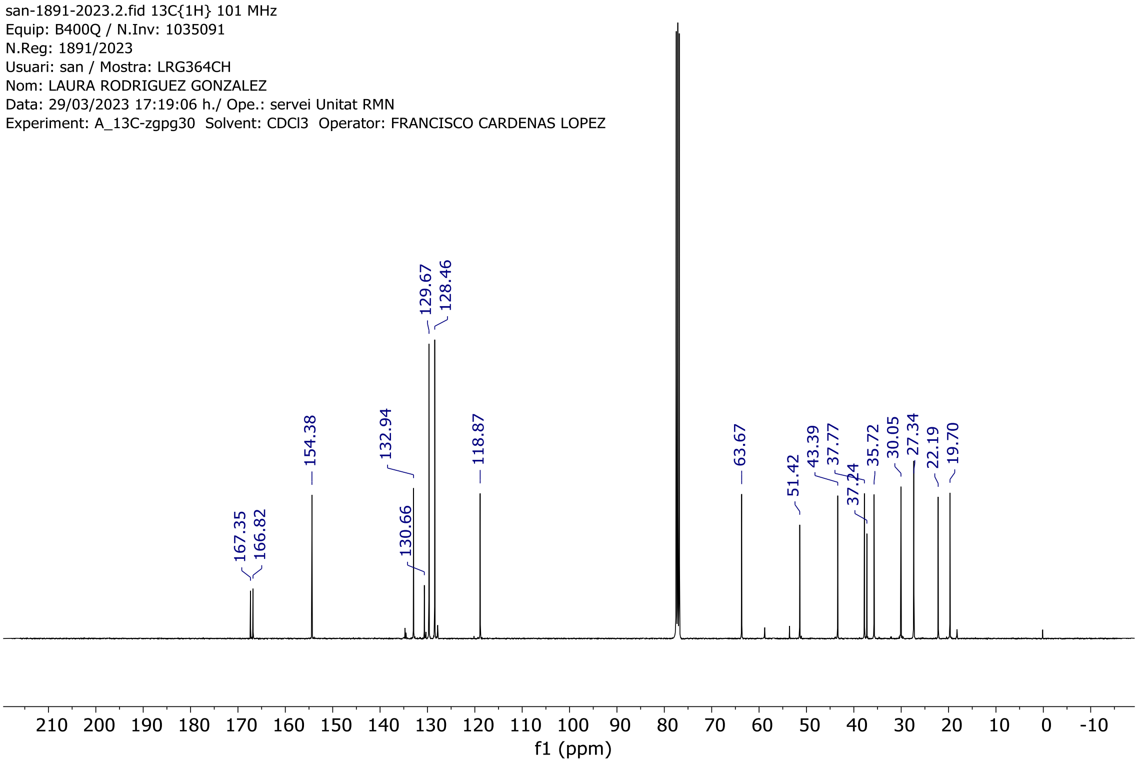

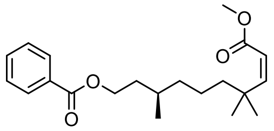

3i

2D-COSY

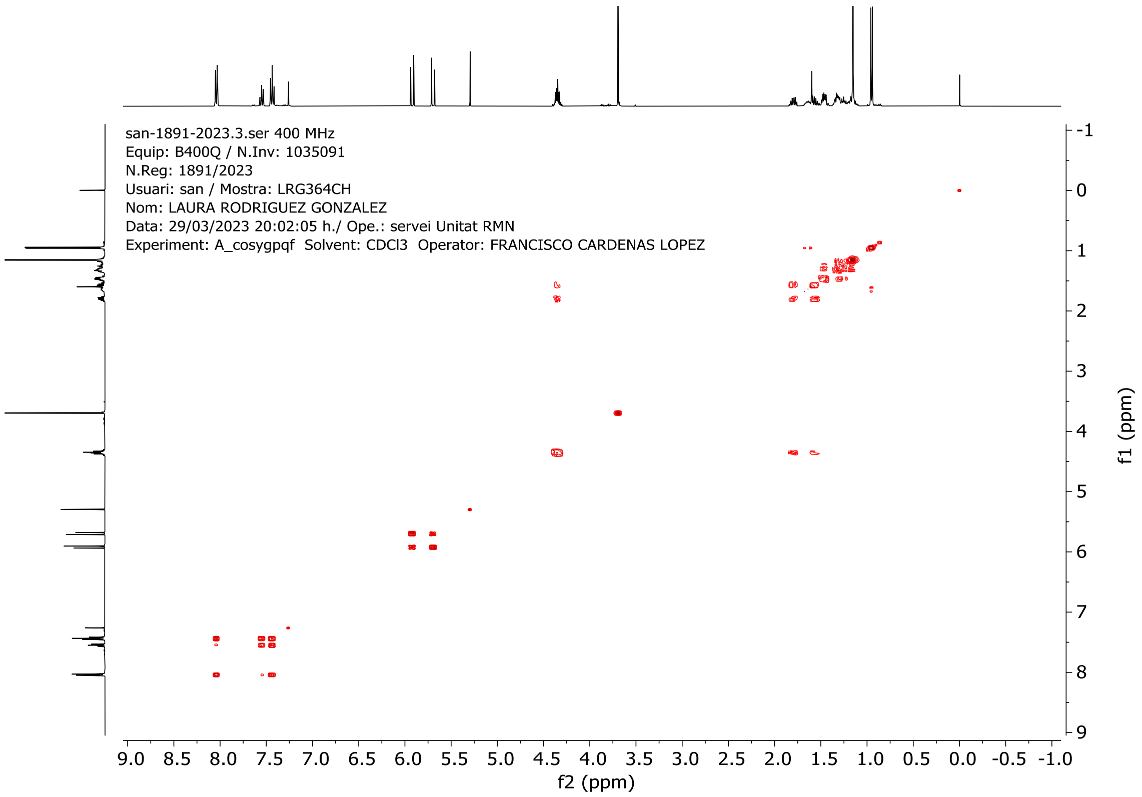

2D-HSQC

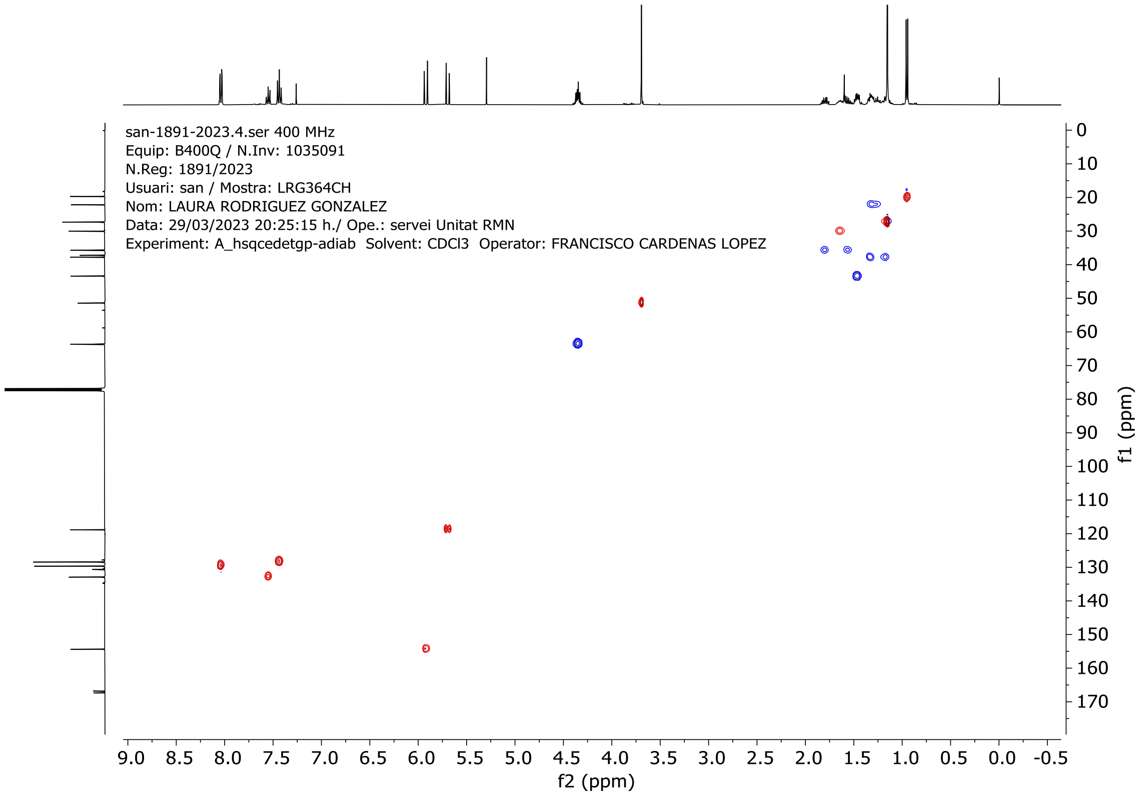

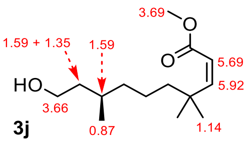

3j

23070080_B400FA_05072023_LRG426COLT34.1.fid 1H 400 MHz
Equip: B400F / N.Inv: 1037597
N.Reg: 23070080
Usuari: san / Mostra: LRG426COLT34
Nom: LAURA RODRIGUEZ GONZALEZ
Data: 05/07/2023 11:44:19 h./ Ope.: AUTOSERVEI
Experiment: A-H1-zg30 Solvent: CDCl3

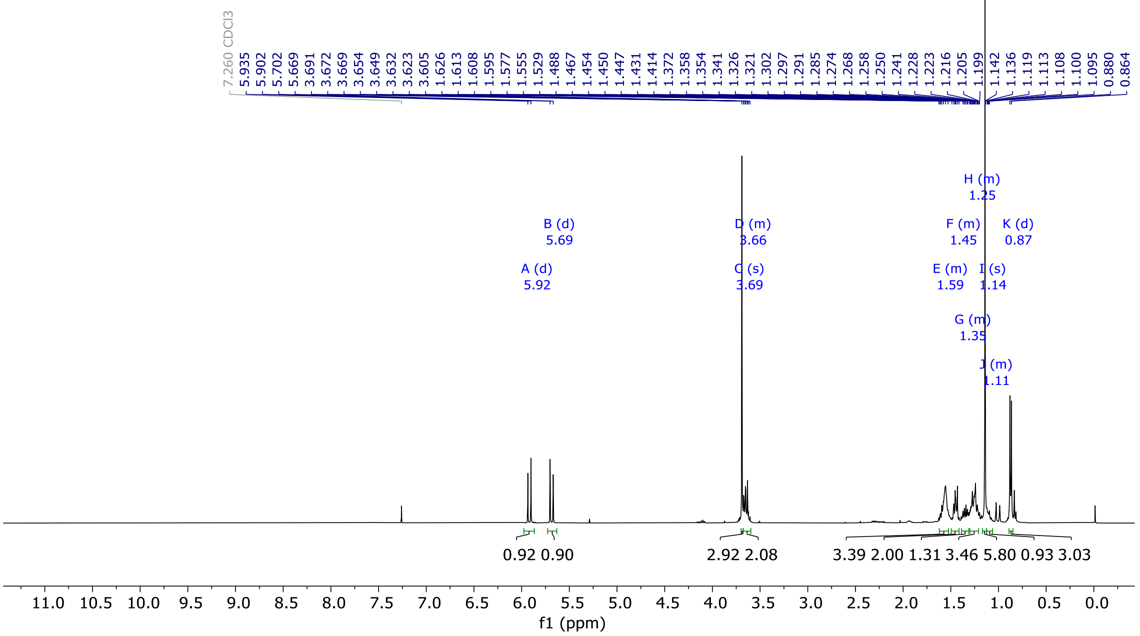

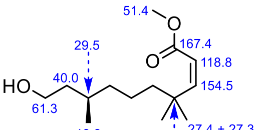

3j

23070073_B400FA_05072023_LRG426CH.2.fid 13C{1H} 101 MHz
Equip: B400F / N.Inv: 1037597
N.Reg: 23070073
Usuari: san / Mostra: LRG426CH
Nom: LAURA RODRIGUEZ GONZALEZ
Data: 05/07/2023 21:06:50 h./ Ope.: AUTOSERVEI
Experiment: A-C13-zgpg30 Solvent: CDCl3

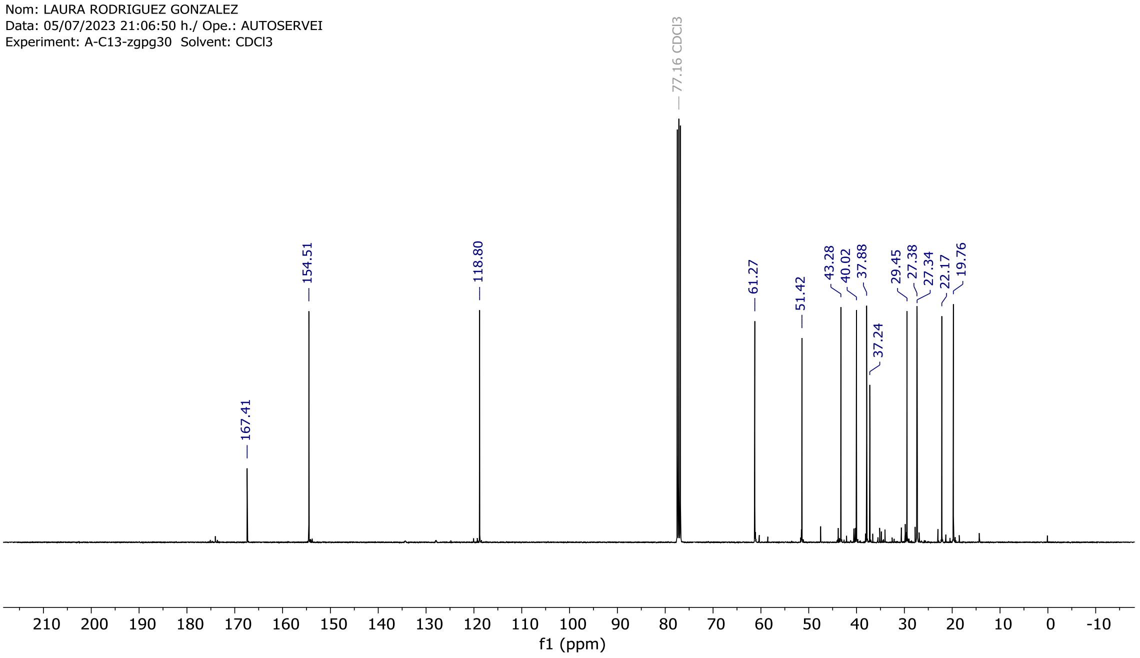

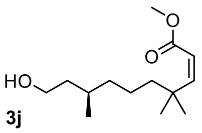

3j

2D-COSY

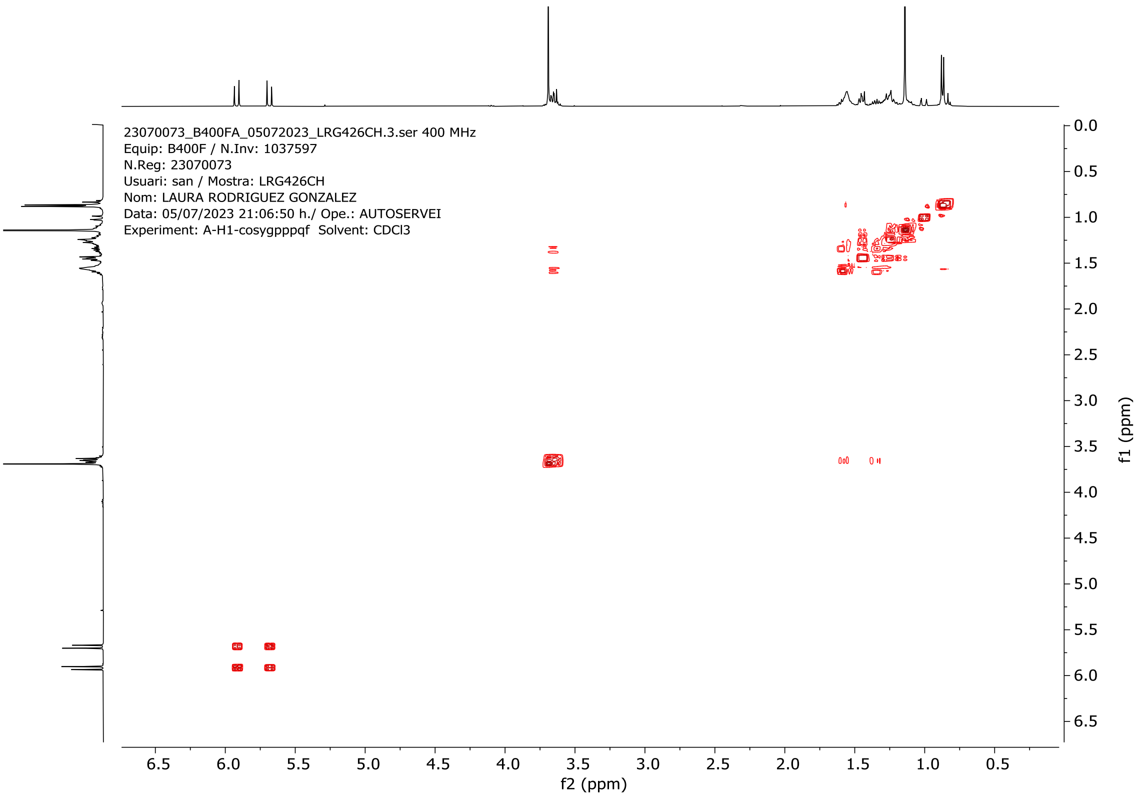

2D-HSQC

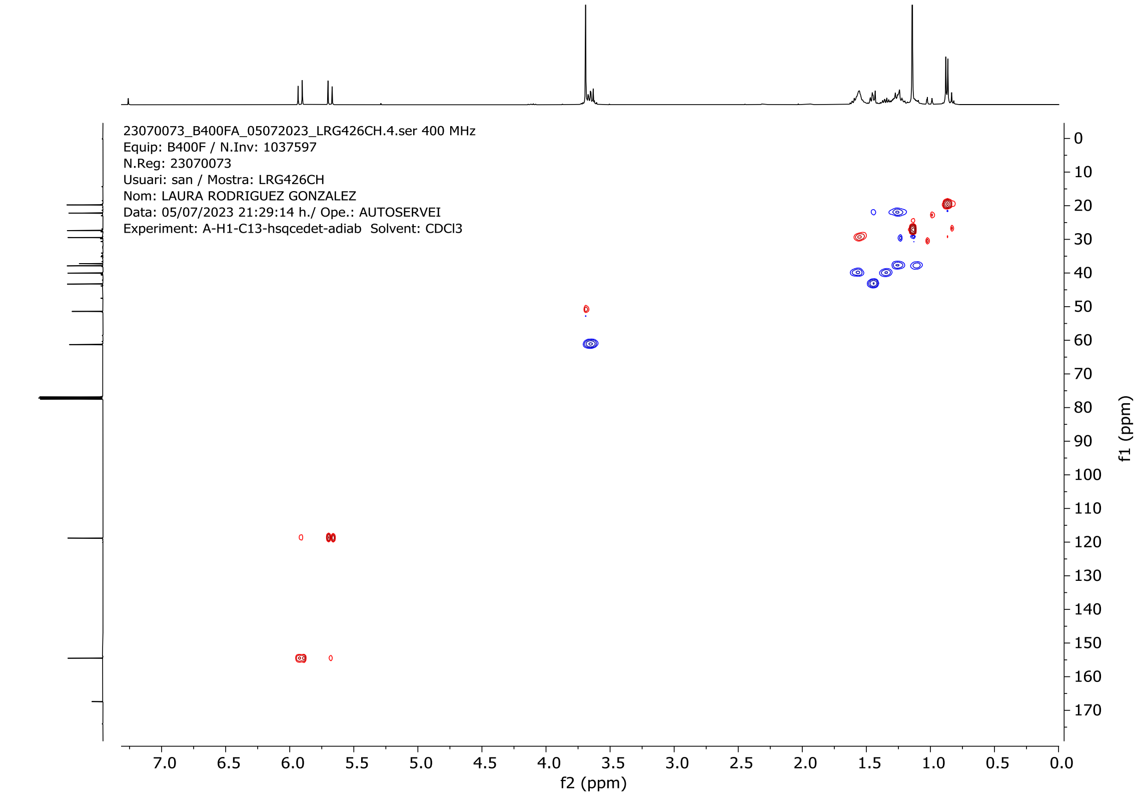

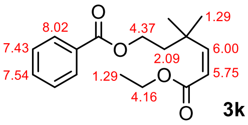

3k

LRG323CHcomp.1.fid 1H 400 MHz
Equip: B400F / N.Inv: 1037597
N.Reg: 23020706
Usuari: san / Mostra: LRG323CH
Nom: LAURA RODRIGUEZ GONZALEZ
Data: 17/02/2023 13:49:57 h./ Ope.: AUTOSERVEI
Experiment: A-H1-zg30 Solvent: CDCl3

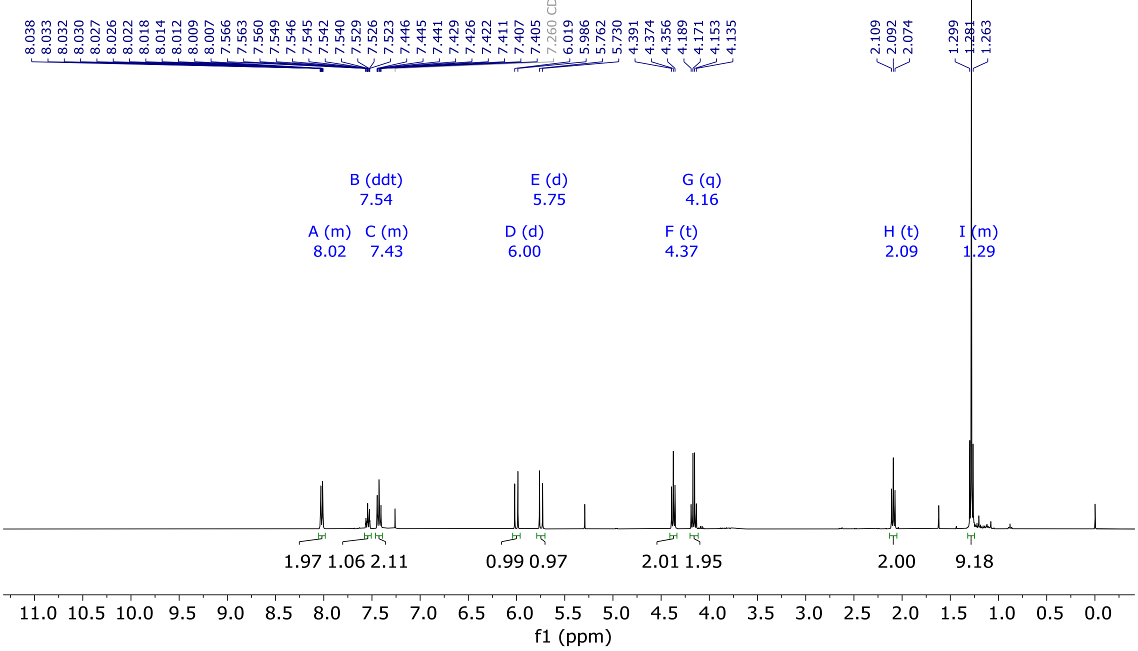

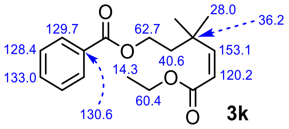

3k

LRG323CHcomp.2.fid 13C{1H} 101 MHz
Equip: B400F / N.Inv: 1037597
N.Reg: 23020706
Usuari: san / Mostra: LRG323CH
Nom: LAURA RODRIGUEZ GONZALEZ
Data: 18/02/2023 01:21:14 h./ Ope.: AUTOSERVEI
Experiment: A-C13-zgpg30 Solvent: CDCl3

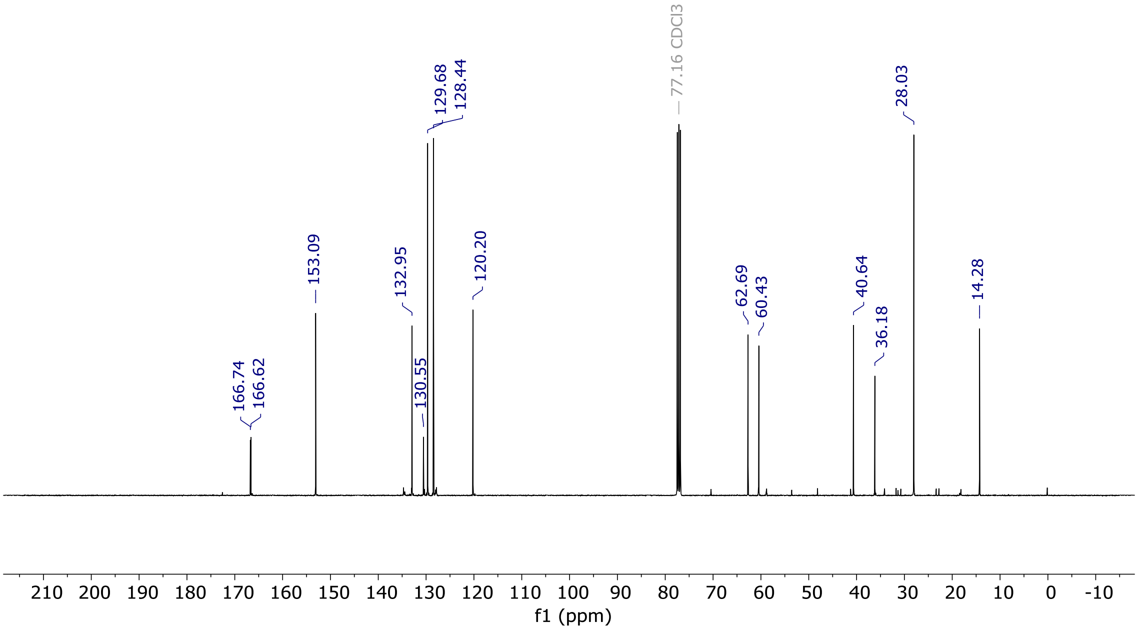

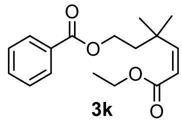

3k

2D-COSY

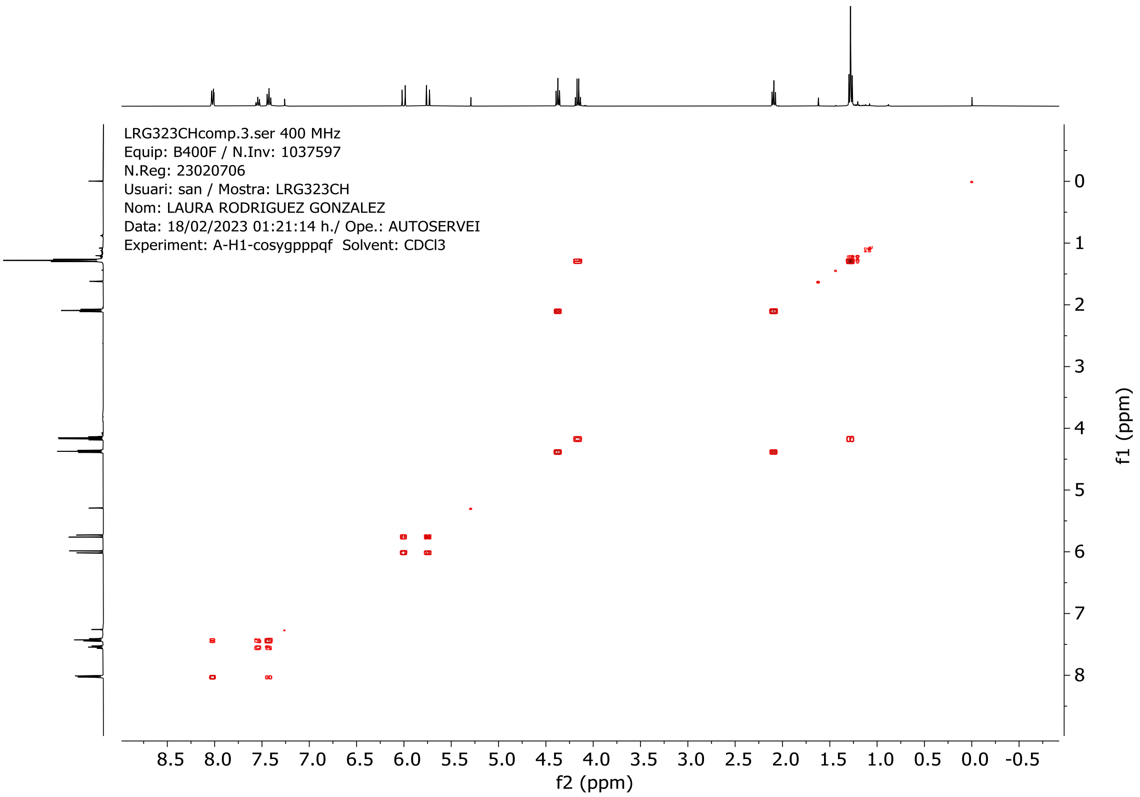

2D-HSQC

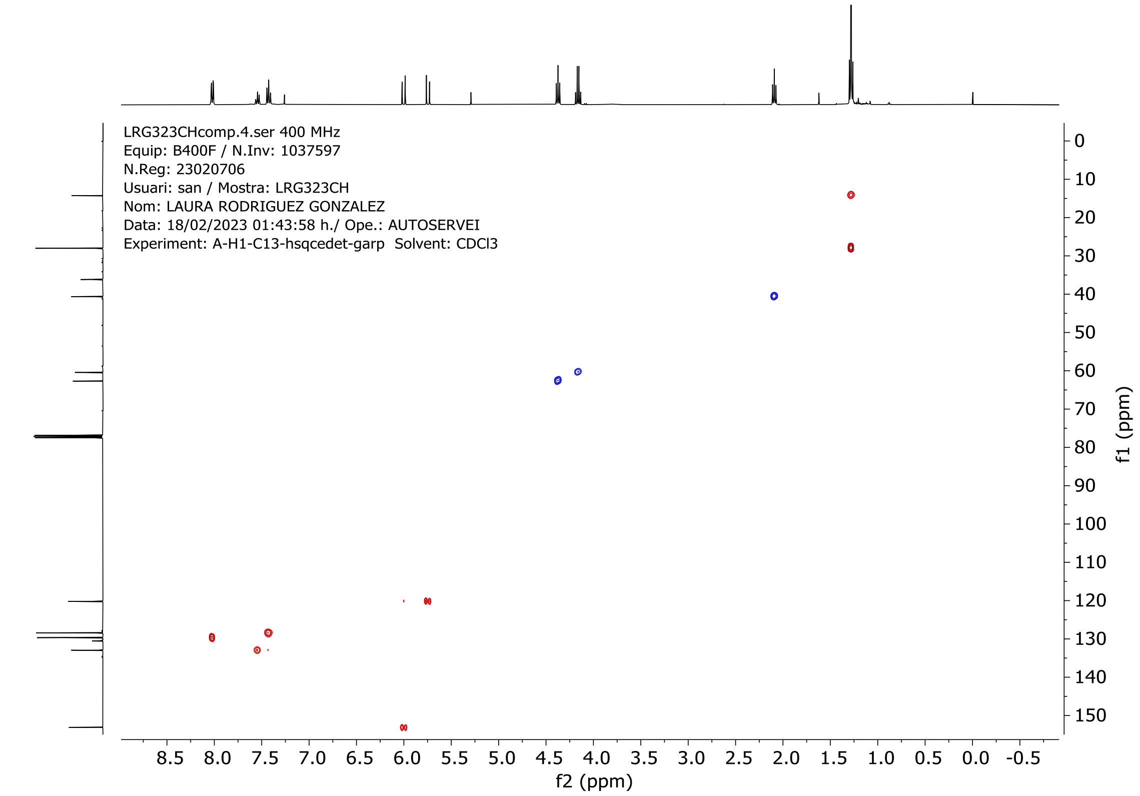

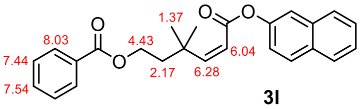

3l

23050548_B400FA_18052023_LRG389COL1T25T26CH.1.fid 1H 400 MHz
Equip: B400F / N.Inv: 1037597
N.Reg: 23050548
Usuari: san / Mostra: LRG389COL1T25T26CH
Nom: LAURA RODRIGUEZ GONZALEZ
Data: 17/05/2023 15:34:14 h./ Ope.: AUTOSERVEI
Experiment: A-H1-zg30 Solvent: CDCl3

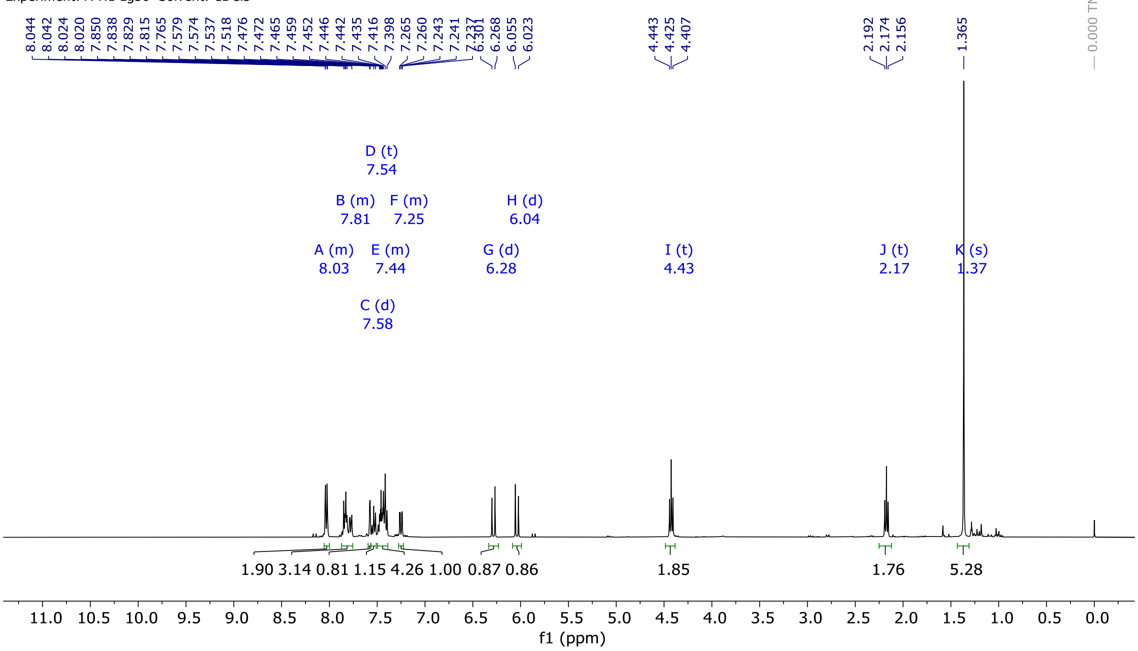

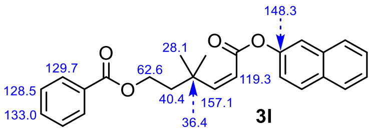

3l

23050548_B400FA_18052023_LRG389COL1T25T26CH.2.fid 13C{1H} 101 MHz
Equip: B400F / N.Inv: 1037597
N.Reg: 23050548
Usuari: san / Mostra: LRG389COL1T25T26CH
Nom: LAURA RODRIGUEZ GONZALEZ
Data: 18/05/2023 02:14:54 h./ Ope.: AUTOSERVEI
Experiment: A-C13-zgpg30 Solvent: CDCl3

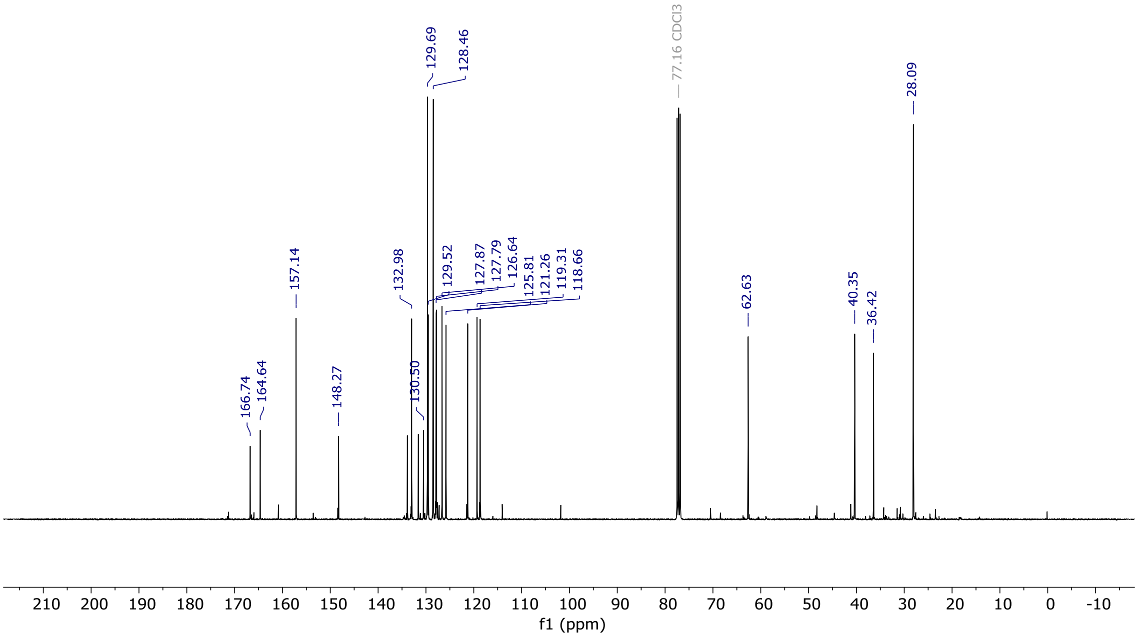

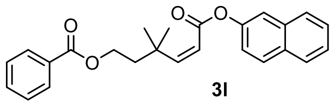

3l

2D-COSY

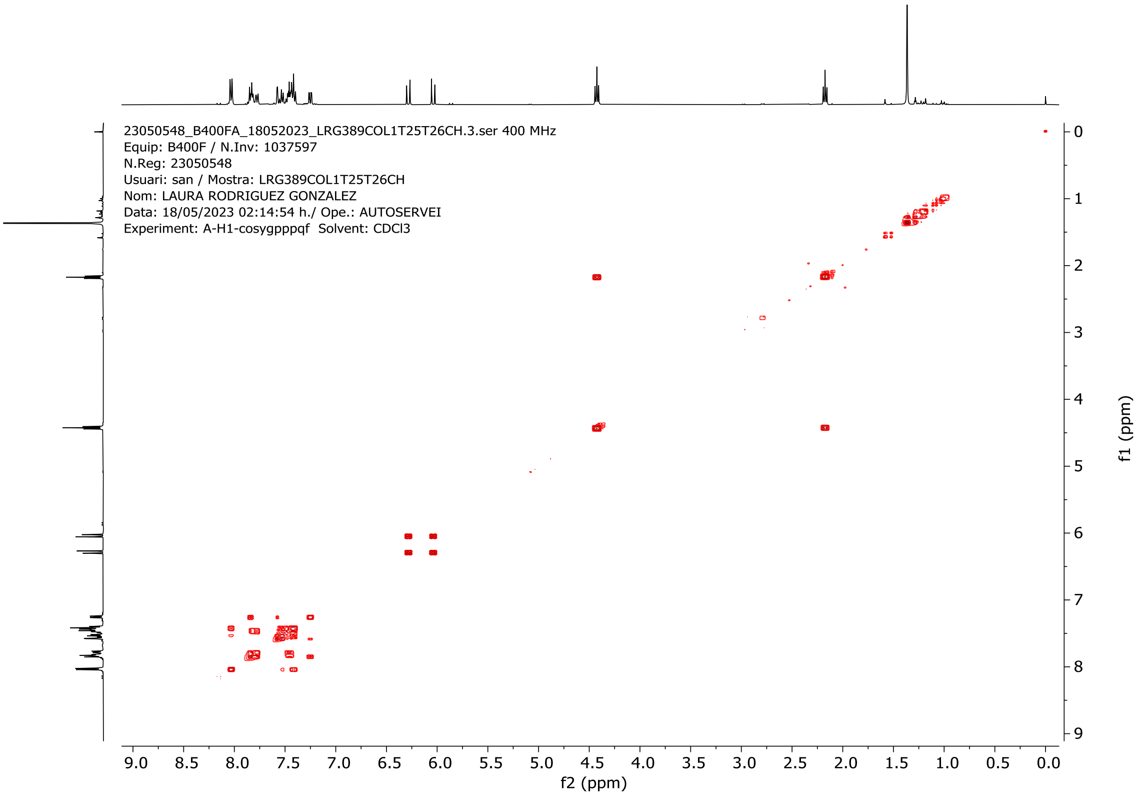

2D-HSQC

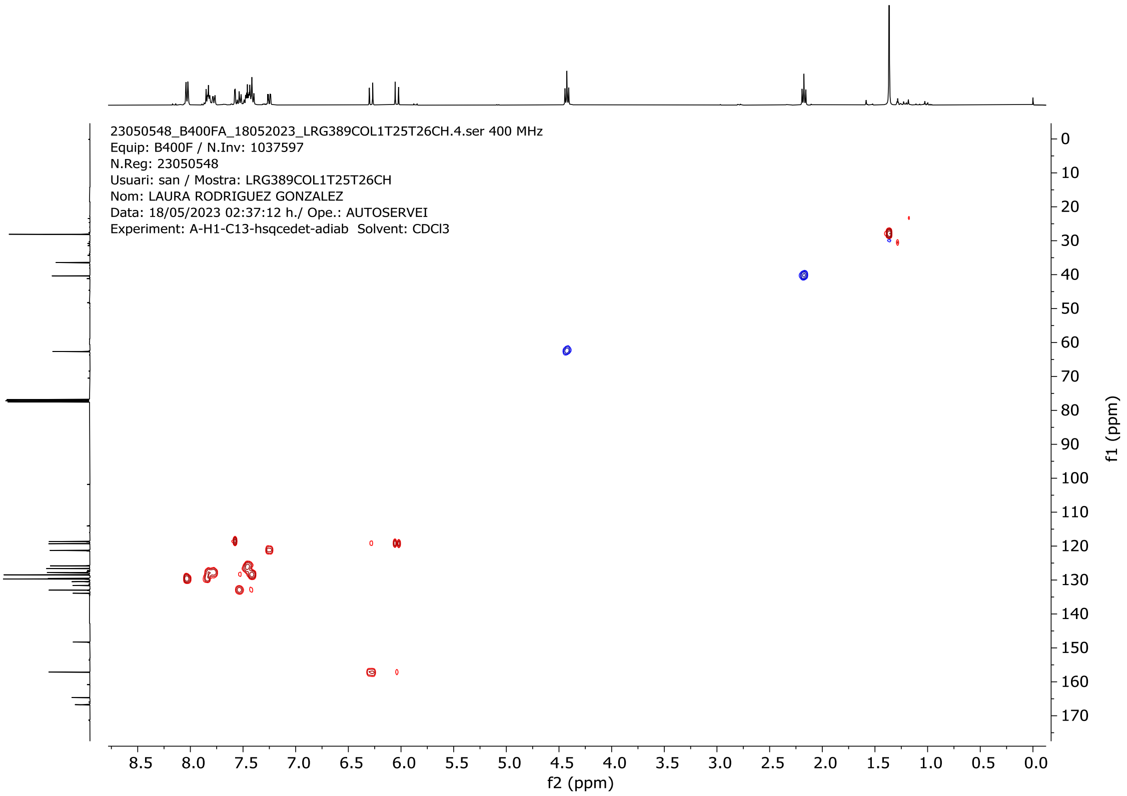

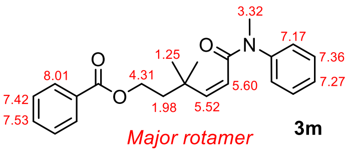

3m

23060362_B400FA_15062023_LRG401RECT37T38.1.fid 1H 400 MHz
Equip: B400F / N.Inv: 1037597
N.Reg: 23060362
Usuari: san / Mostra: LRG401RECT37T38
Nom: LAURA RODRIGUEZ GONZALEZ
Data: 15/06/2023 16:51:30 h./ Ope.: AUTOSERVEI
Experiment: A-H1-zg30 Solvent: CDCl3

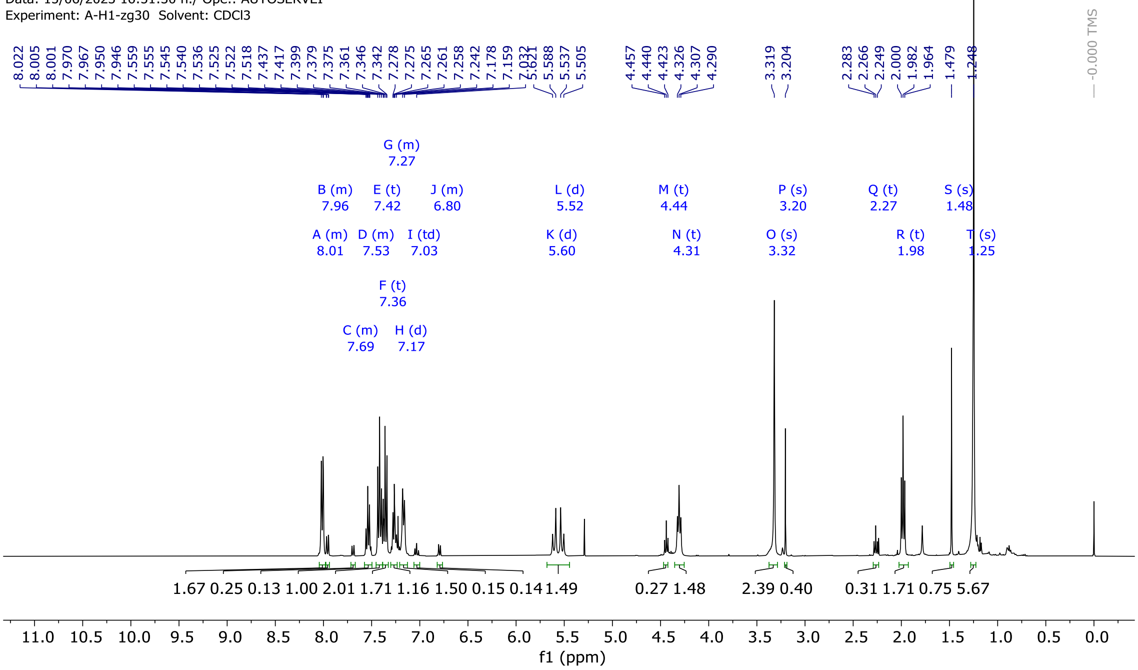

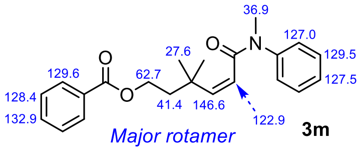

3m

23060362_B400FA_16062023_LRG401RECT37T38.2.fid 13C{1H} 101 MHz
Equip: B400F / N.Inv: 1037597
N.Reg: 23060362
Usuari: san / Mostra: LRG401RECT37T38
Nom: LAURA RODRIGUEZ GONZALEZ
Data: 16/06/2023 04:16:09 h./ Ope.: AUTOSERVEI
Experiment: A-C13-zgpg30 Solvent: CDCl3

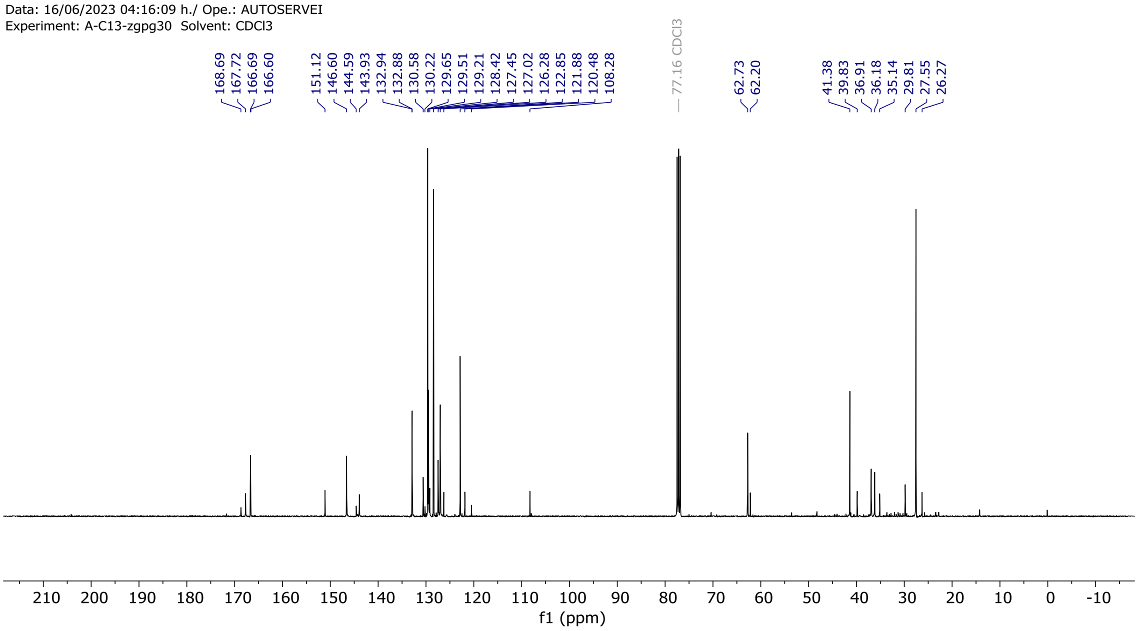

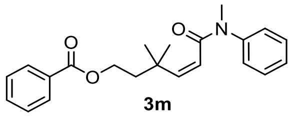

3m

2D-COSY

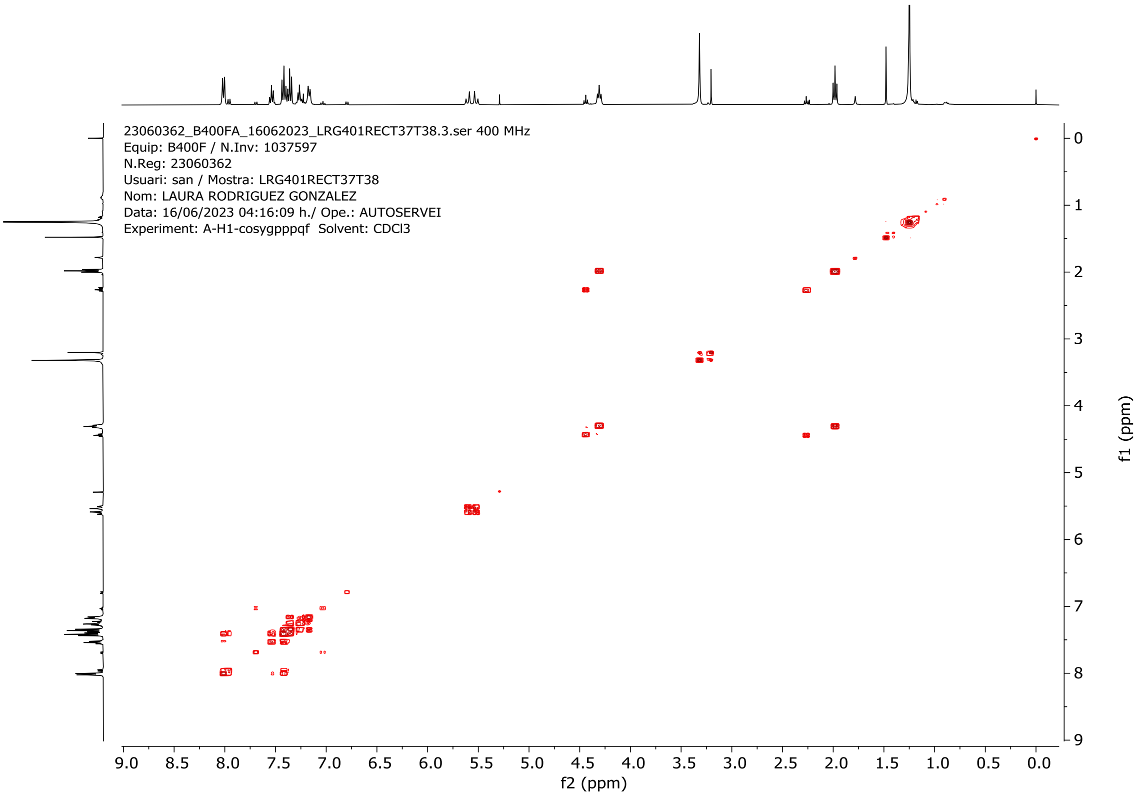

2D-HSQC

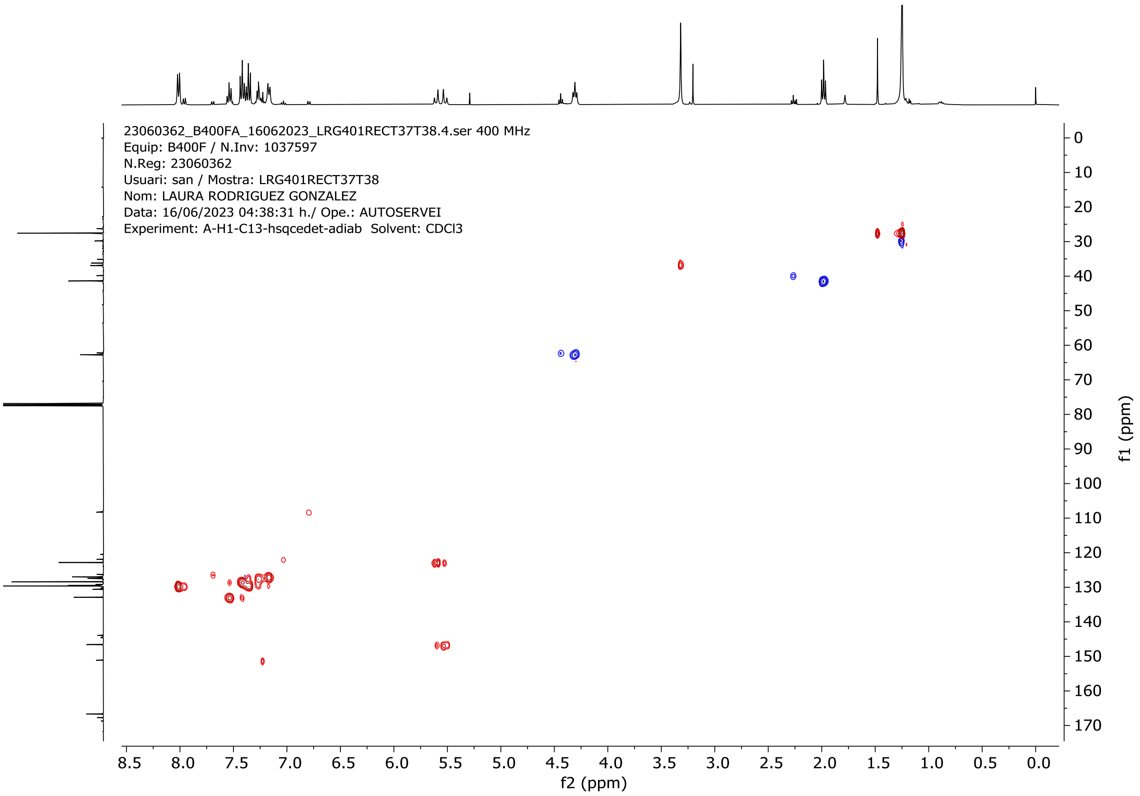

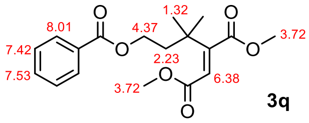

3q

san-2381-2023.10.fid 1H 500 MHz
Equip: B500Q / N.Inv: 1028917
N.Reg: 2381/2023
Usuari: san / Mostra: LRG377CH
Nom: LAURA RODRIGUEZ GONZALEZ
Data: 26/04/2023 00:33:49 h./ Ope.: servei Unitat RMN
Experiment: A_1H-zg30 Solvent: CDCl3 Operator: M. ANTONIA MOLINS

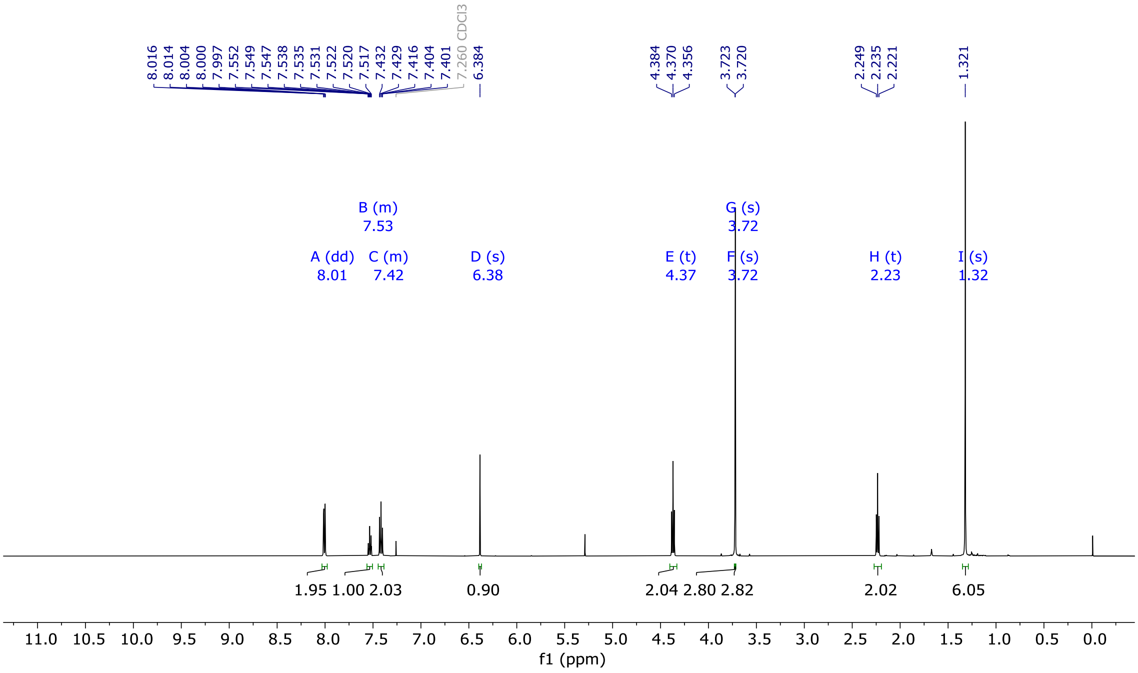

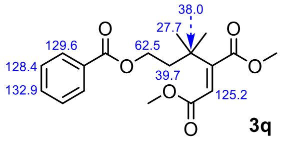

3q

2604230430-2381-2023_B500QA_26042023_LRG377CH.13.fid 13C{1H} 126 MHz
Equip: B500Q / N.Inv: 1028917
N.Reg: 2381/2023
Usuari: san / Mostra: LRG377CH
Nom: LAURA RODRIGUEZ GONZALEZ
Data: 26/04/2023 01:55:38 h./ Ope.: servei Unitat RMN
Experiment: A_13C-zgpg30 Solvent: CDCl3 Operator: M. ANTONIA MOLINS

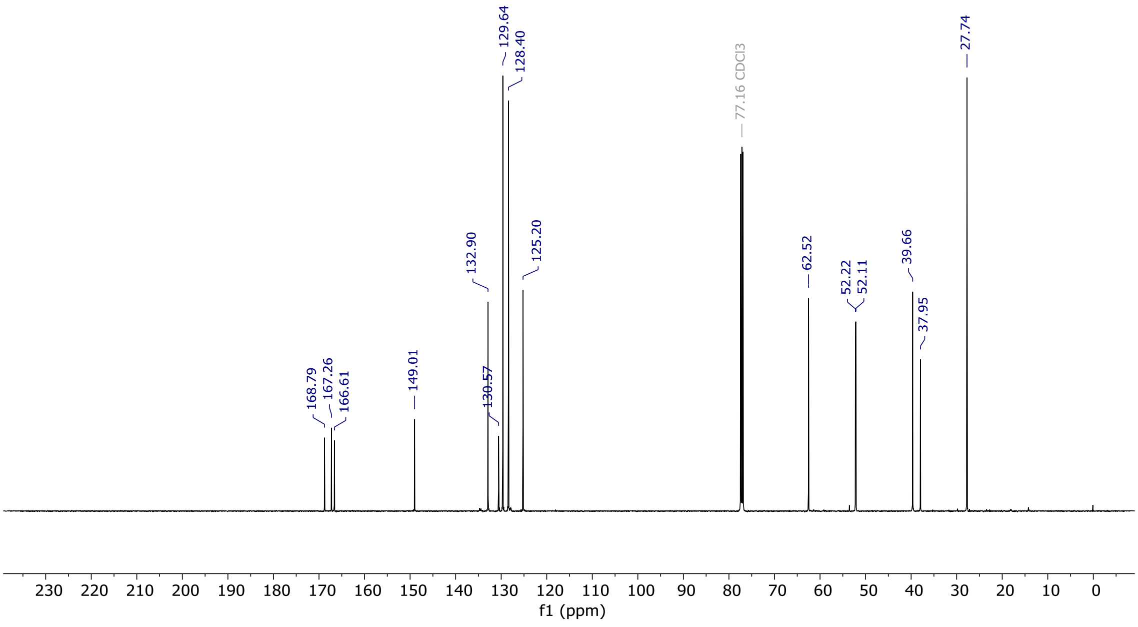

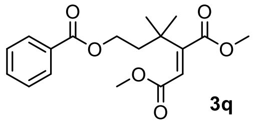

3q

2D-COSY

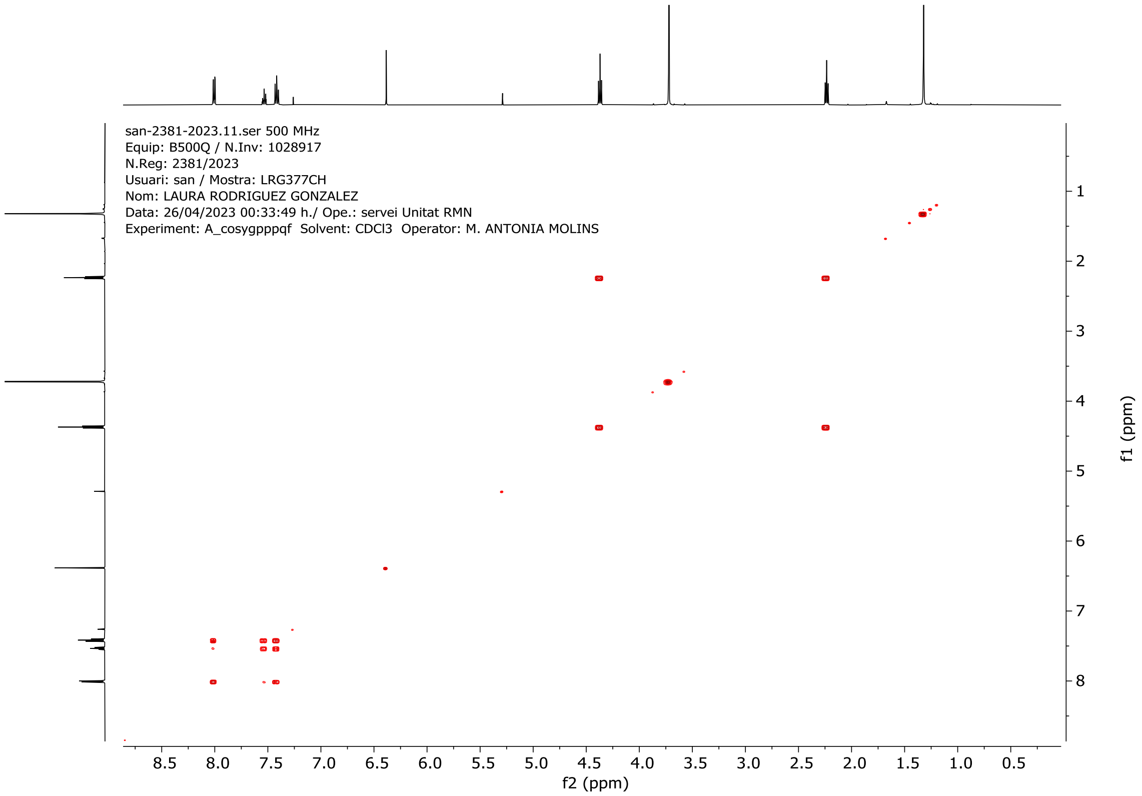

2D-HSQC

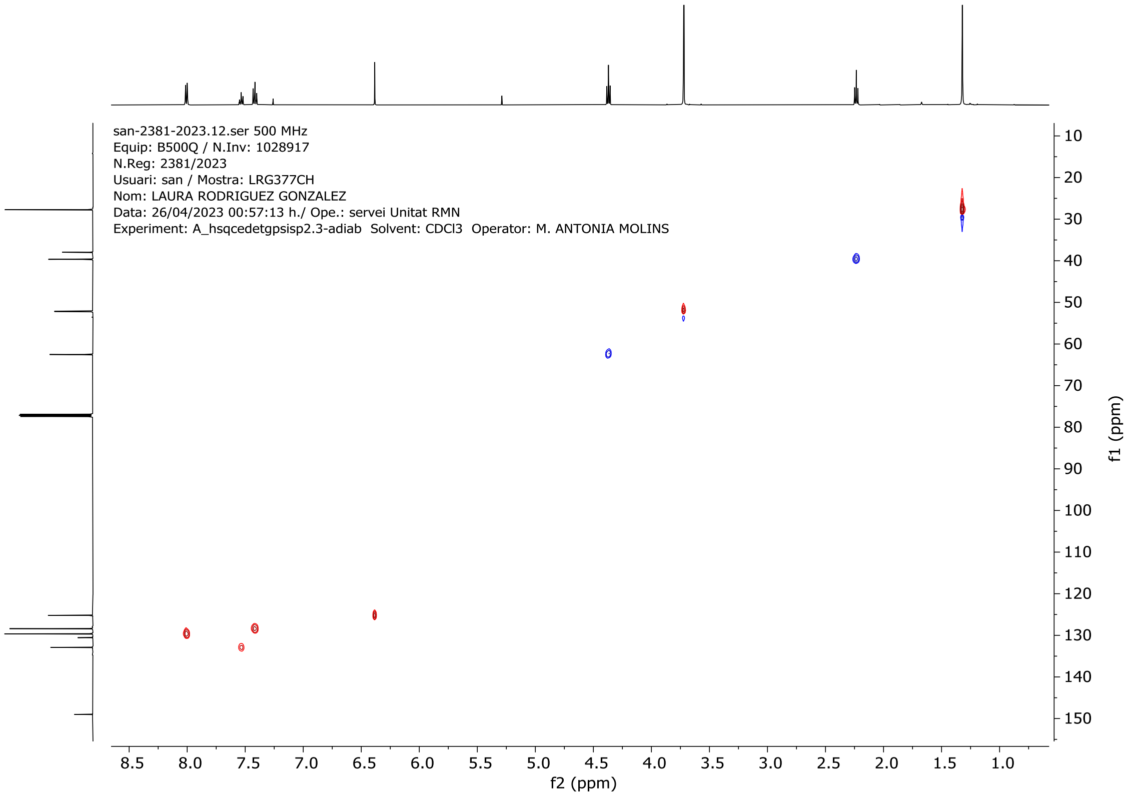

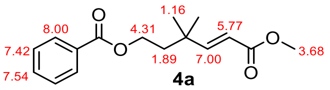

23060391_B400FA_16062023_LRG406RECT30T31.1.fid 1H 400 MHz
Equip: B400F / N.Inv: 1037597
N.Reg: 23060391
Usuari: san / Mostra: LRG406RECT30T31
Nom: LAURA RODRIGUEZ GONZALEZ
Data: 16/06/2023 11:41:09 h./ Ope.: AUTOSERVEI
Experiment: A-H1-zg30 Solvent: CDCl3

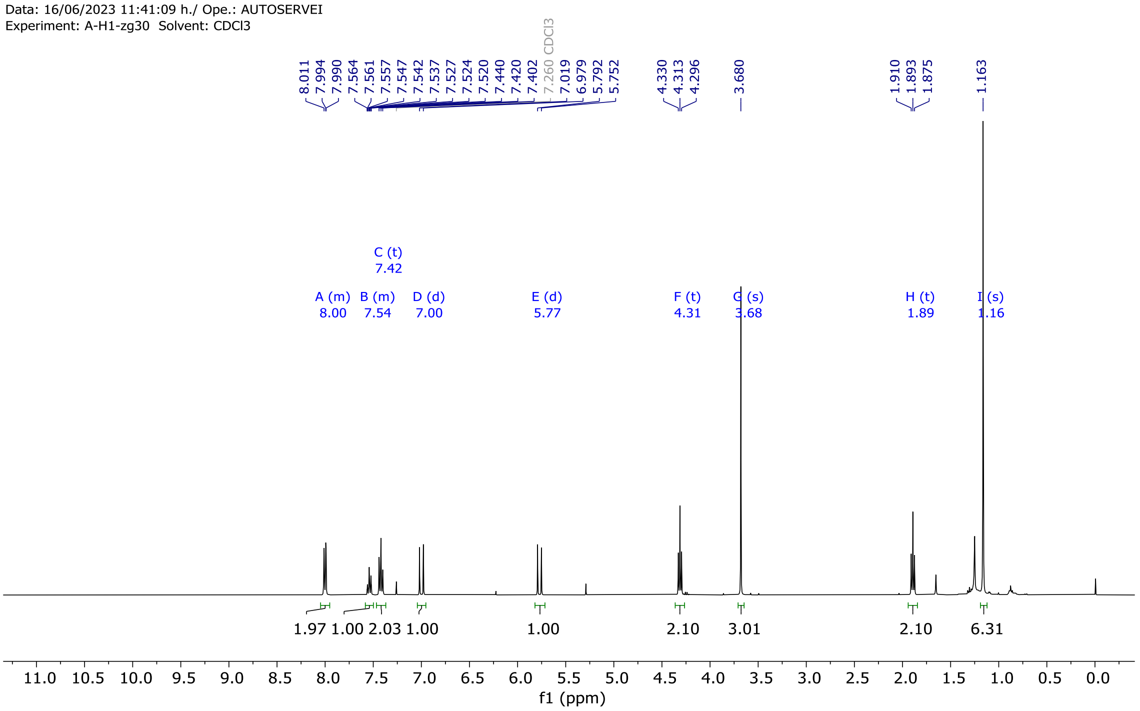

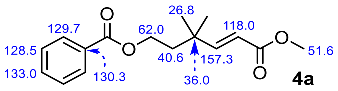

23060392_B400FA_17062023_LRG406RECT30T31CH.2.fid 13C{1H} 101 MHz
Equip: B400F / N.Inv: 1037597
N.Reg: 23060392
Usuari: san / Mostra: LRG406RECT30T31CH
Nom: LAURA RODRIGUEZ GONZALEZ
Data: 16/06/2023 22:46:14 h./ Ope.: AUTOSERVEI
Experiment: A-C13-zgpg30 Solvent: CDCl3

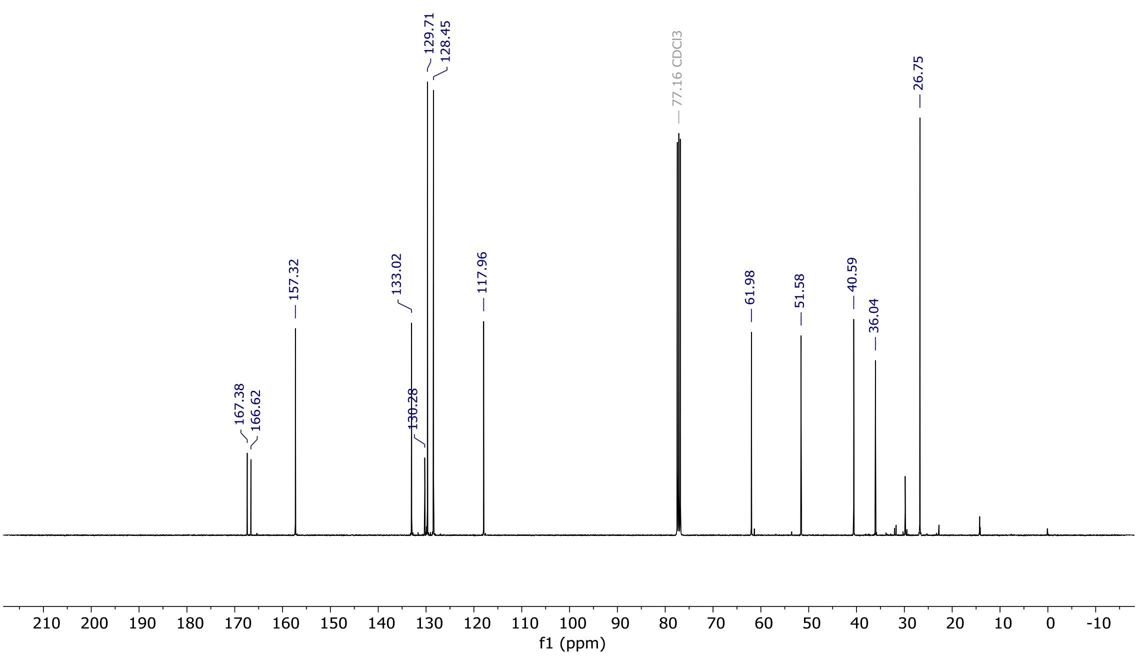

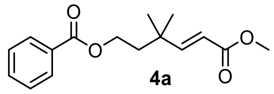

4a

2D-COSY

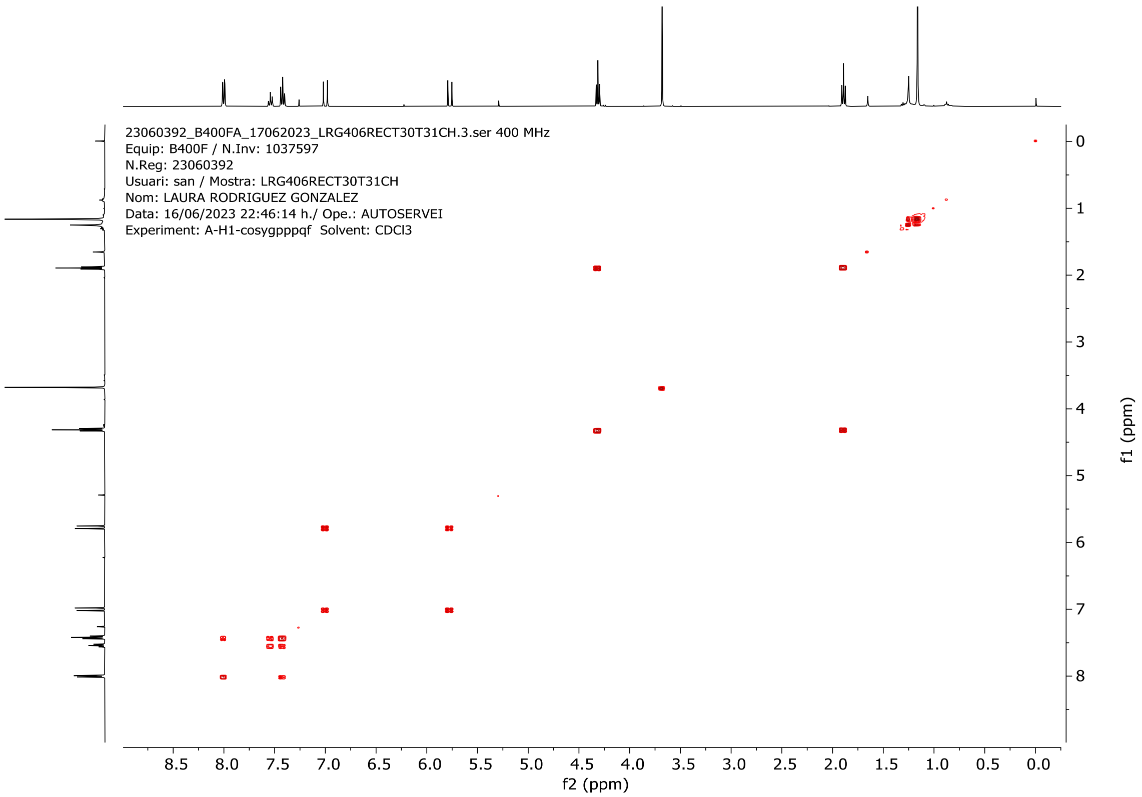

2D-HSQC

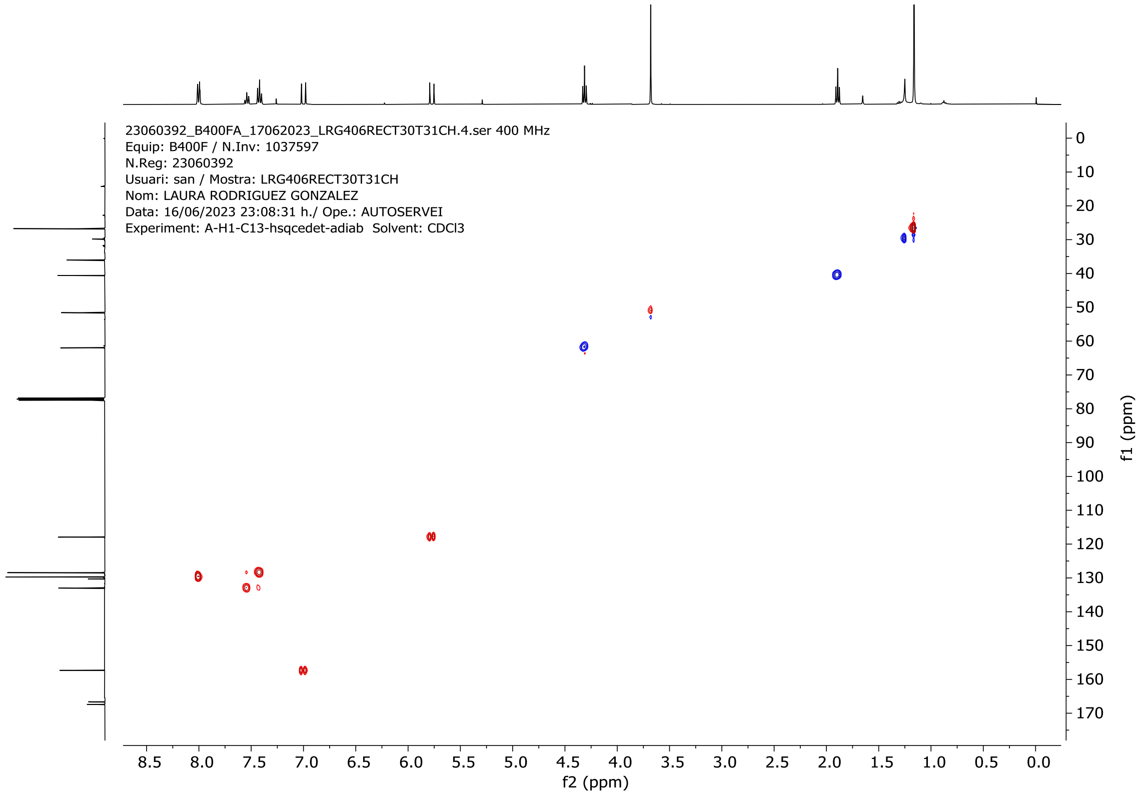

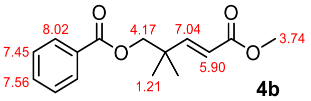

san-5107-2023.1.fid 1H 400 MHz
Equip: B400Q / N.Inv: 1035091
N.Reg: 5107/2023
Usuari: san / Mostra: LRG472TOP
Nom: LAURA RODRIGUEZ GONZALEZ
Data: 03/10/2023 16:01:24 h./ Ope.: servei Unitat RMN
Experiment: A_1H-zg30 Solvent: CDCl3 Operator: DANIEL PUIG

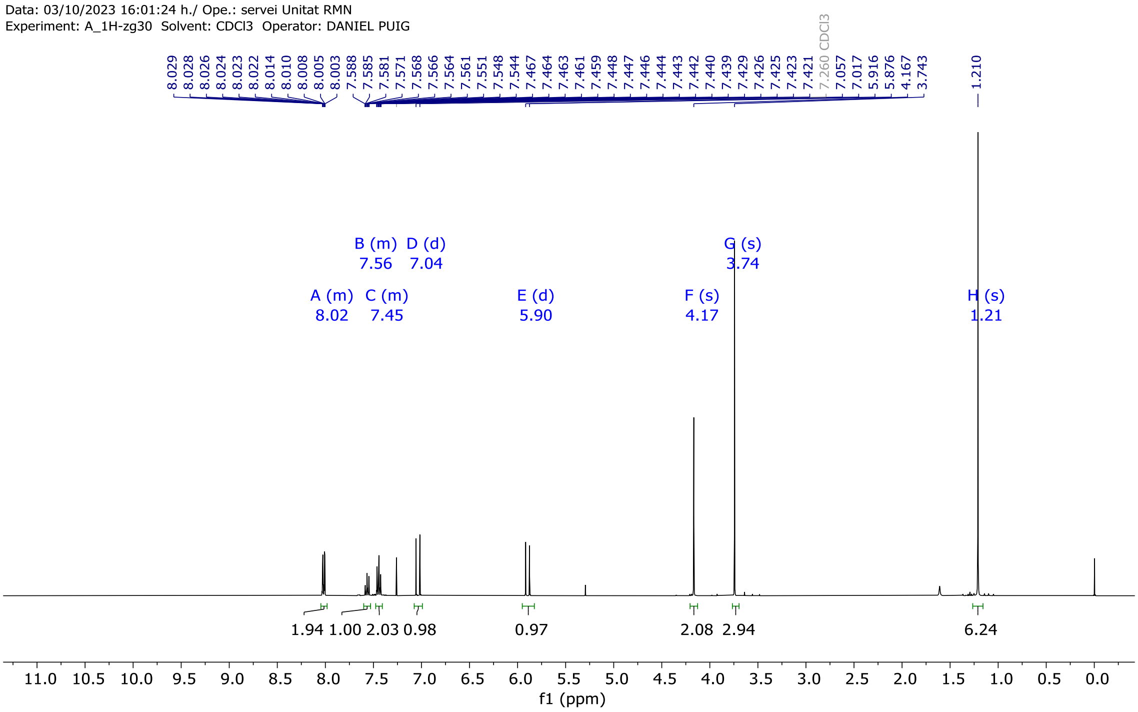

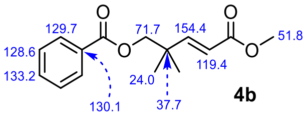

san-5107-2023.3.fid 13C{1H} 101 MHz
Equip: B400Q / N.Inv: 1035091
N.Reg: 5107/2023
Usuari: san / Mostra: LRG472TOP
Nom: LAURA RODRIGUEZ GONZALEZ
Data: 04/10/2023 03:39:14 h./ Ope.: servei Unitat RMN
Experiment: A_13C-zgpg30 Solvent: CDCl3 Operator: FRANCISCO CARDENAS LOPEZ

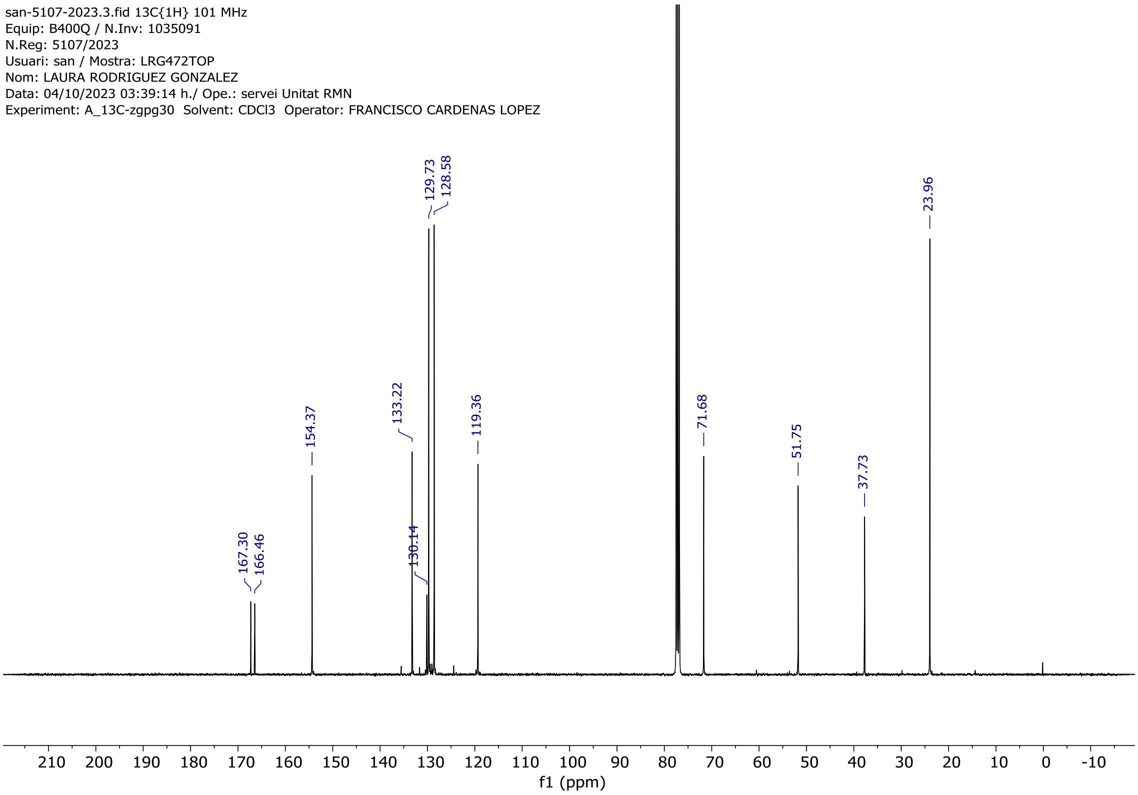

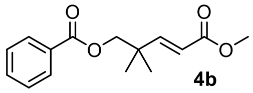

4b

2D-COSY

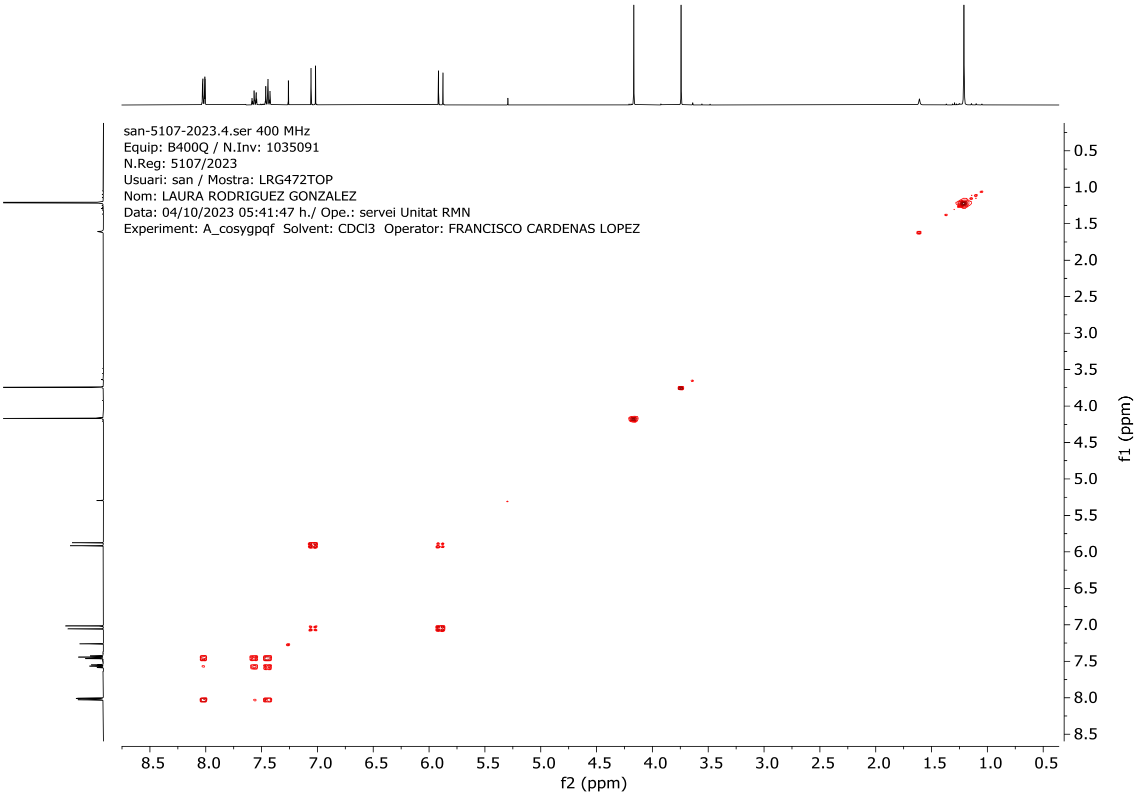

2D-HSQC

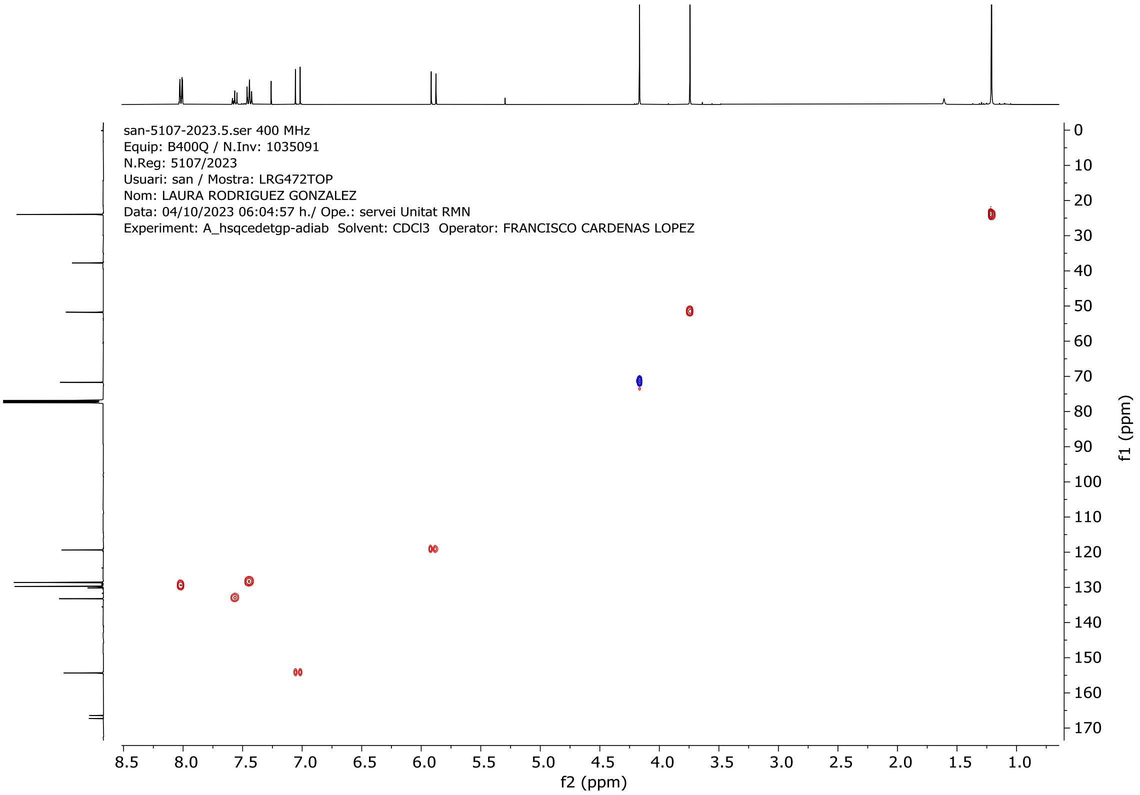

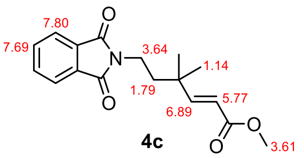

23090335_B400FA_19092023_LRG456COLT34.1.fid 1H 400 MHz
Equip: B400F / N.Inv: 1037597
N.Reg: 23090335
Usuari: san / Mostra: LRG456COLT34
Nom: LAURA RODRIGUEZ GONZALEZ
Data: 19/09/2023 13:30:23 h./ Ope.: AUTOSERVEI
Experiment: A-H1-zg30 Solvent: CDCl3

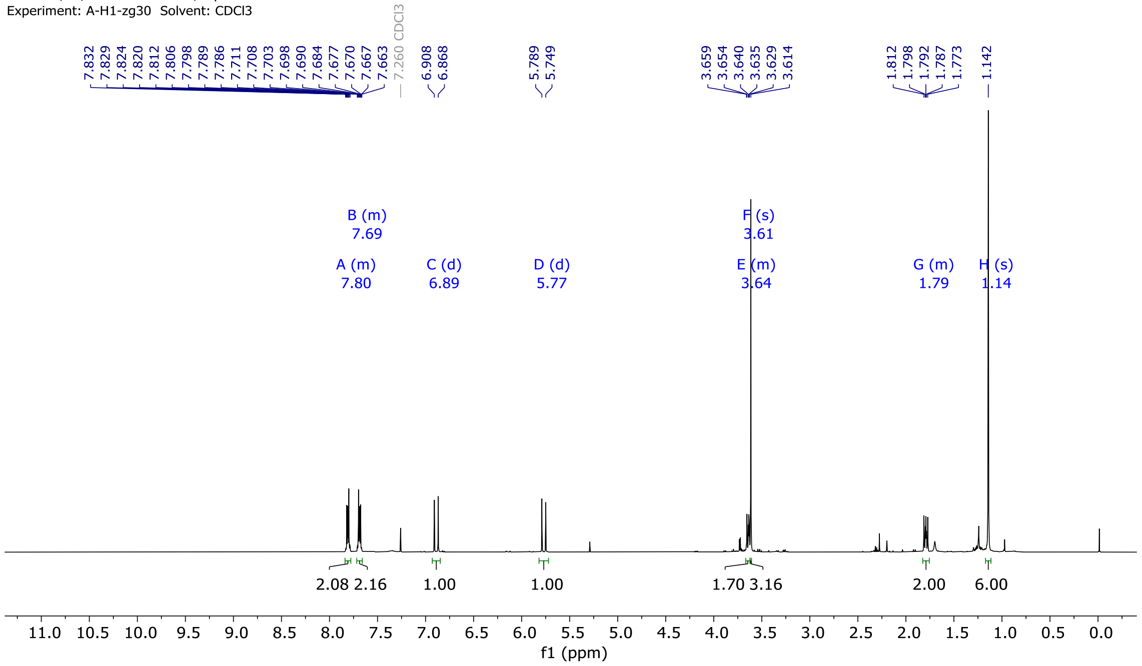

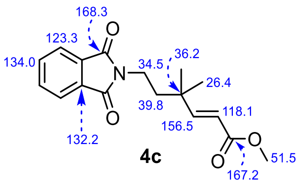

23090332_B400FA_19092023_LRG456CH.2.fid 13C{1H} 101 MHz
Equip: B400F / N.Inv: 1037597
N.Reg: 23090332
Usuari: san / Mostra: LRG456CH
Nom: LAURA RODRIGUEZ GONZALEZ
Data: 19/09/2023 21:08:15 h./ Ope.: AUTOSERVEI
Experiment: A-C13-zgpg30 Solvent: CDCl3

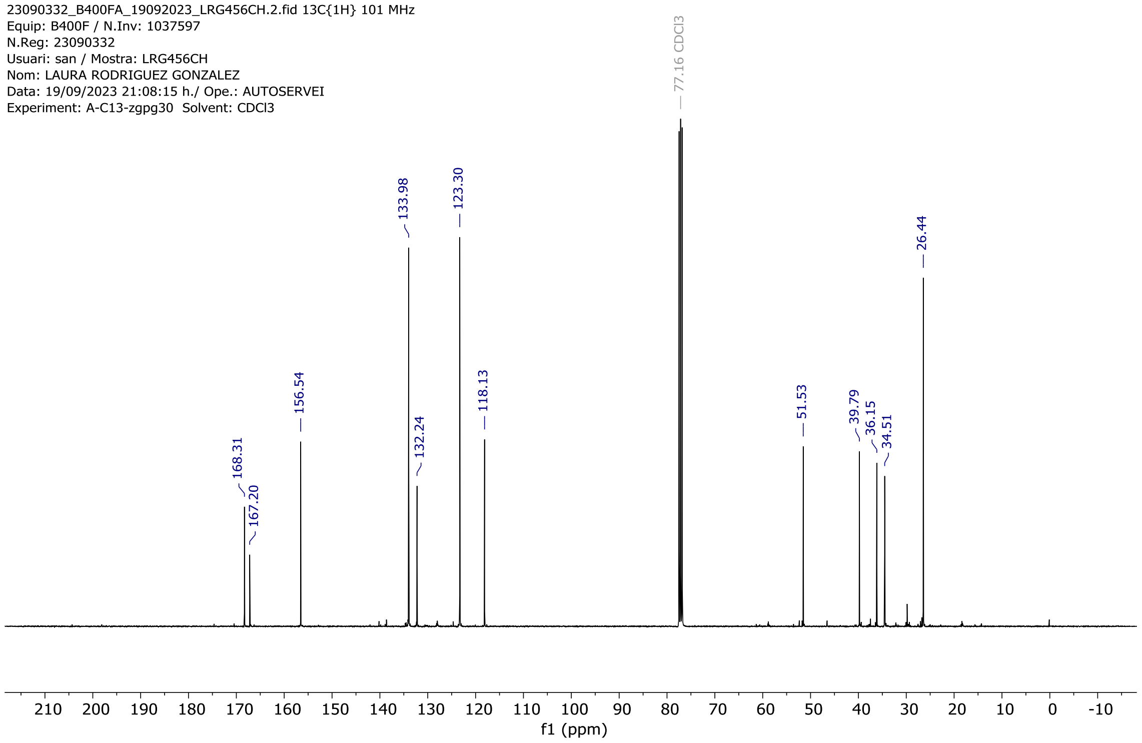

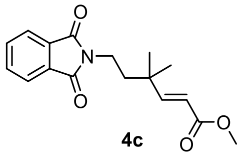

4c

2D-COSY

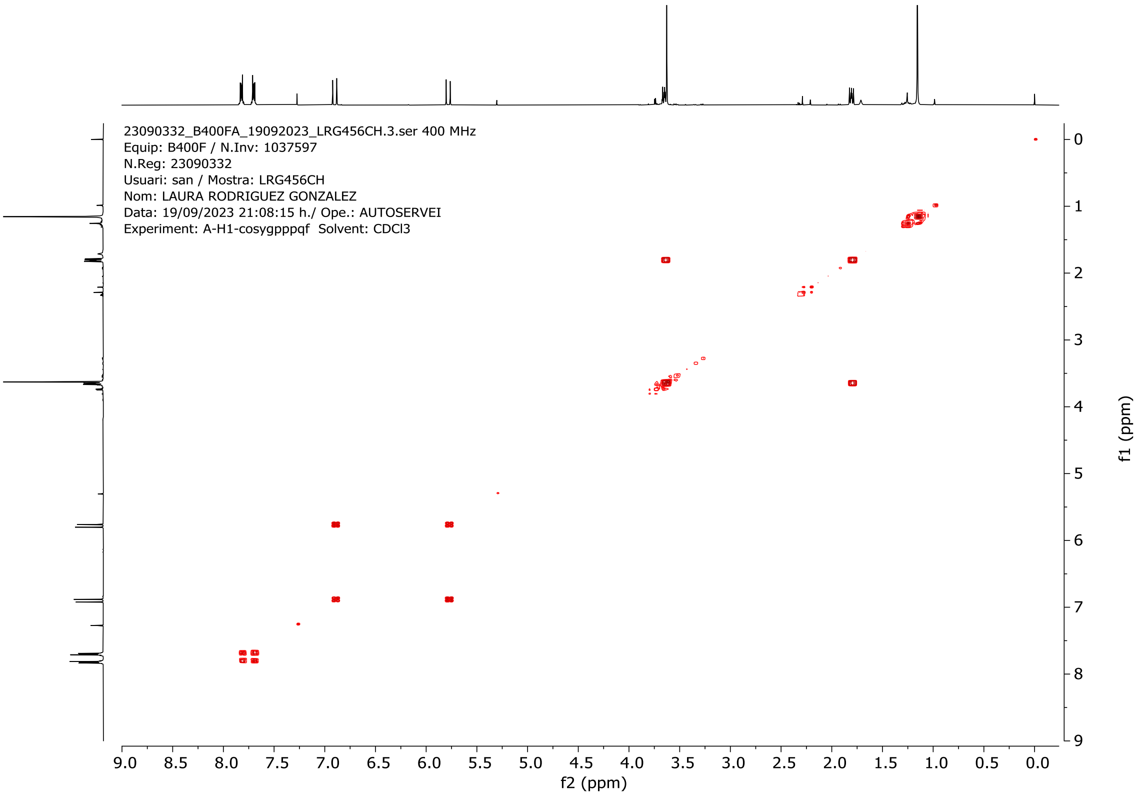

2D-HSQC

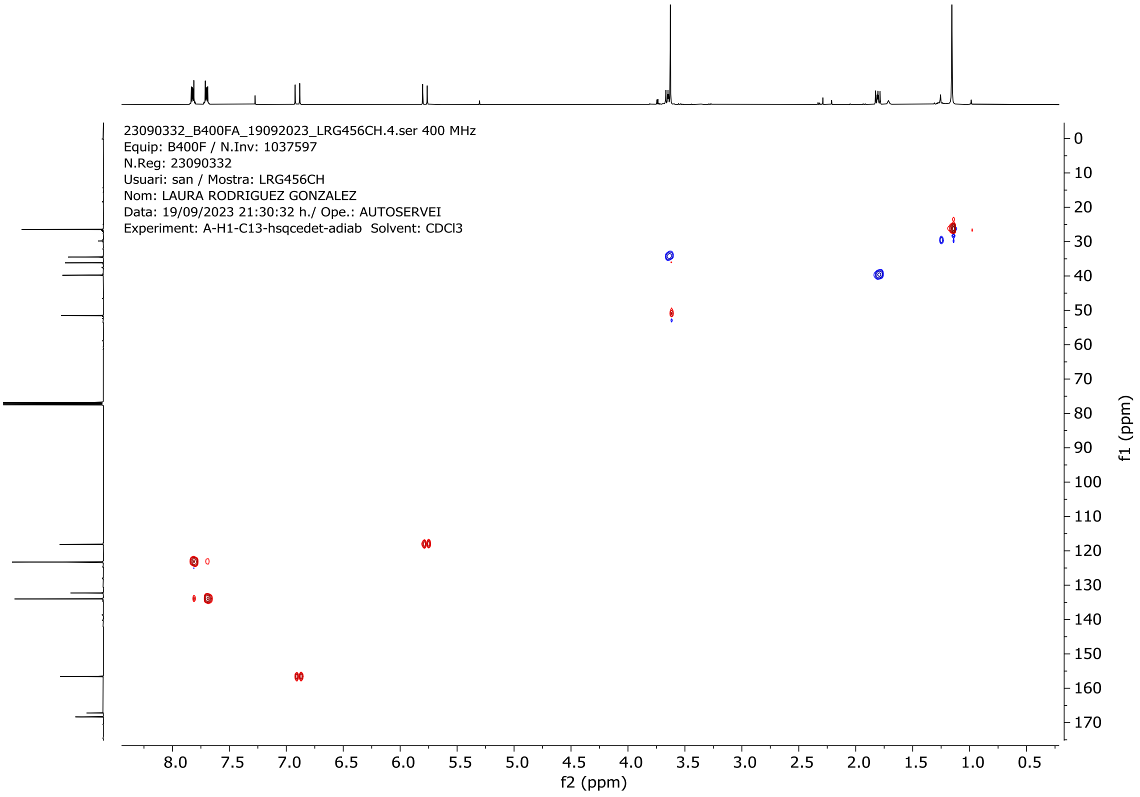

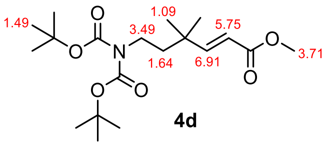

4d

23100264_B400FA_25102023_LRG488CH.1.fid 1H 400 MHz
Equip: B400F / N.Inv: 1037597
N.Reg: 23100264
Usuari: san / Mostra: LRG488CH
Nom: LAURA RODRIGUEZ GONZALEZ
Data: 25/10/2023 11:39:26 h./ Ope.: AUTOSERVEI
Experiment: A-H1-zg30 Solvent: CDCl3

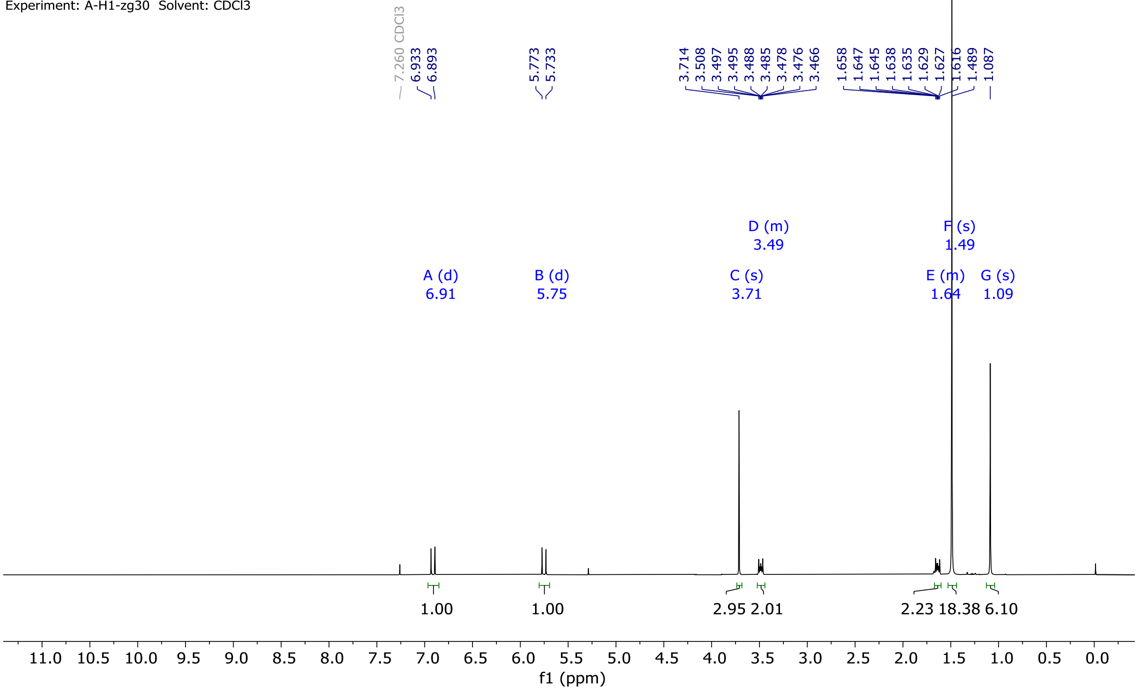

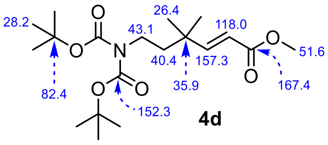

4d

23100264_B400FA_25102023_LRG488CH.2.fid 13C{1H} 101 MHz
Equip: B400F / N.Inv: 1037597
N.Reg: 23100264
Usuari: san / Mostra: LRG488CH
Nom: LAURA RODRIGUEZ GONZALEZ
Data: 25/10/2023 22:04:40 h./ Ope.: AUTOSERVEI
Experiment: A-C13-zgpg30 Solvent: CDCl3

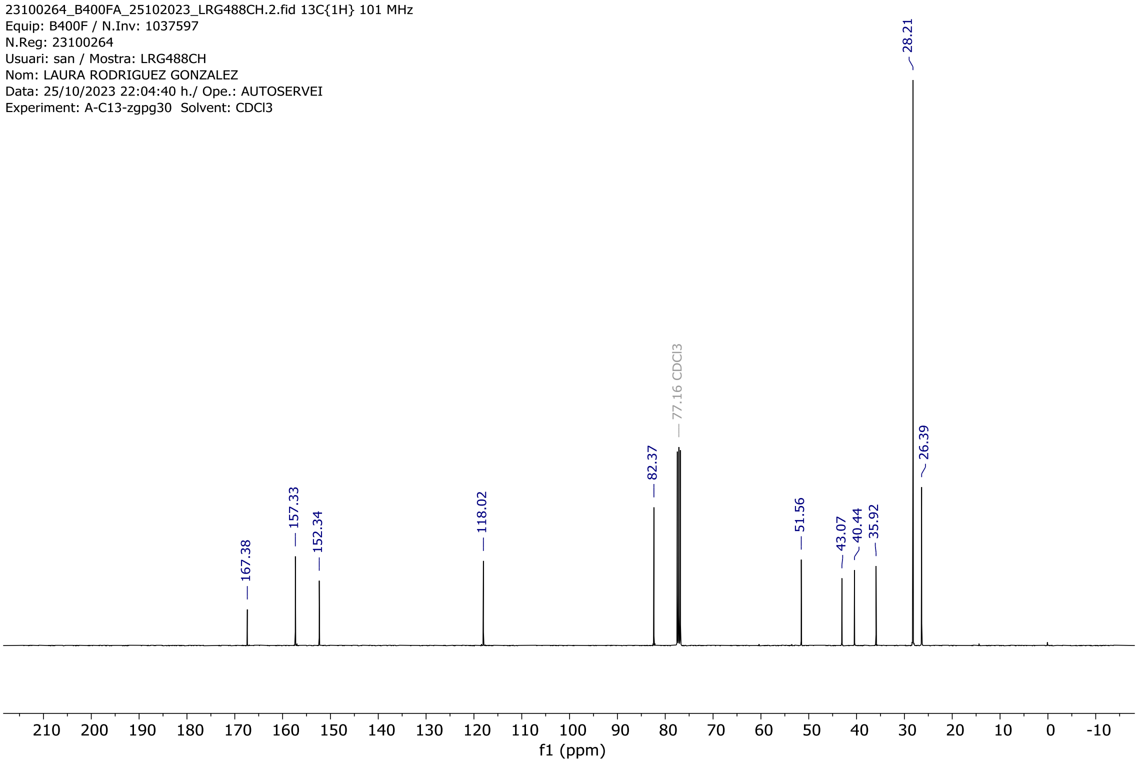

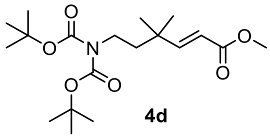

4d

2D-COSY

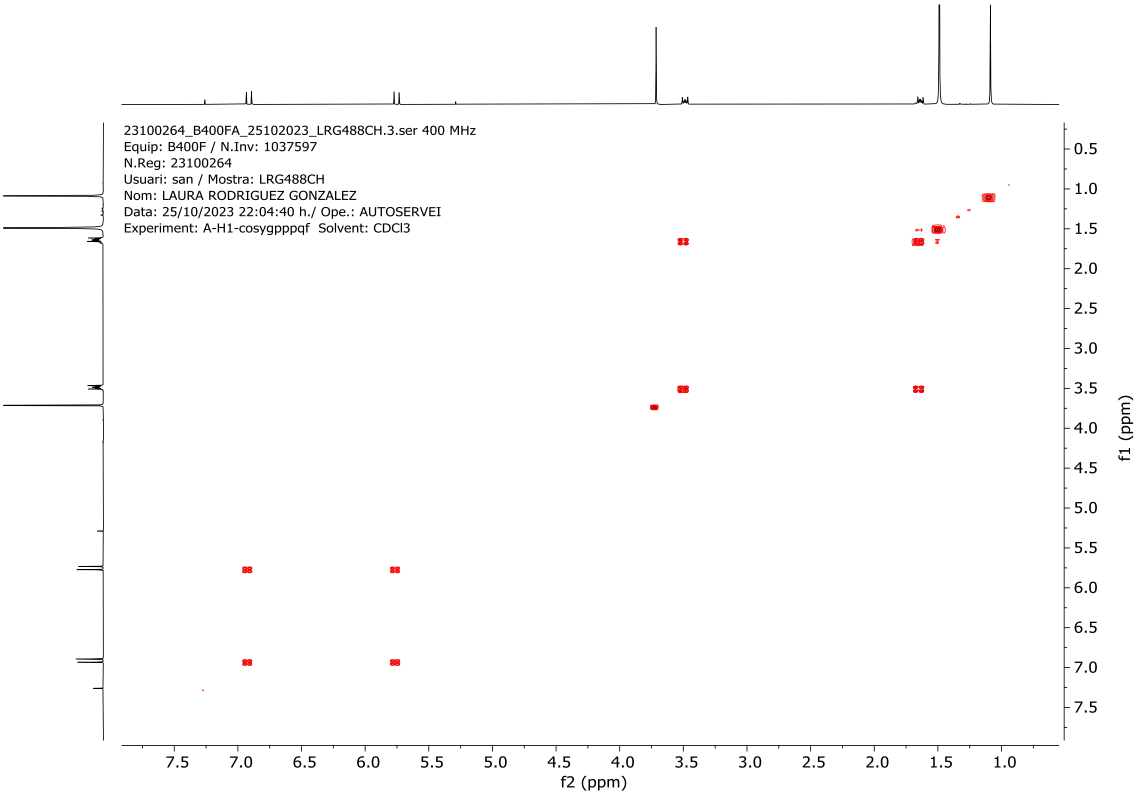

2D-HSQC

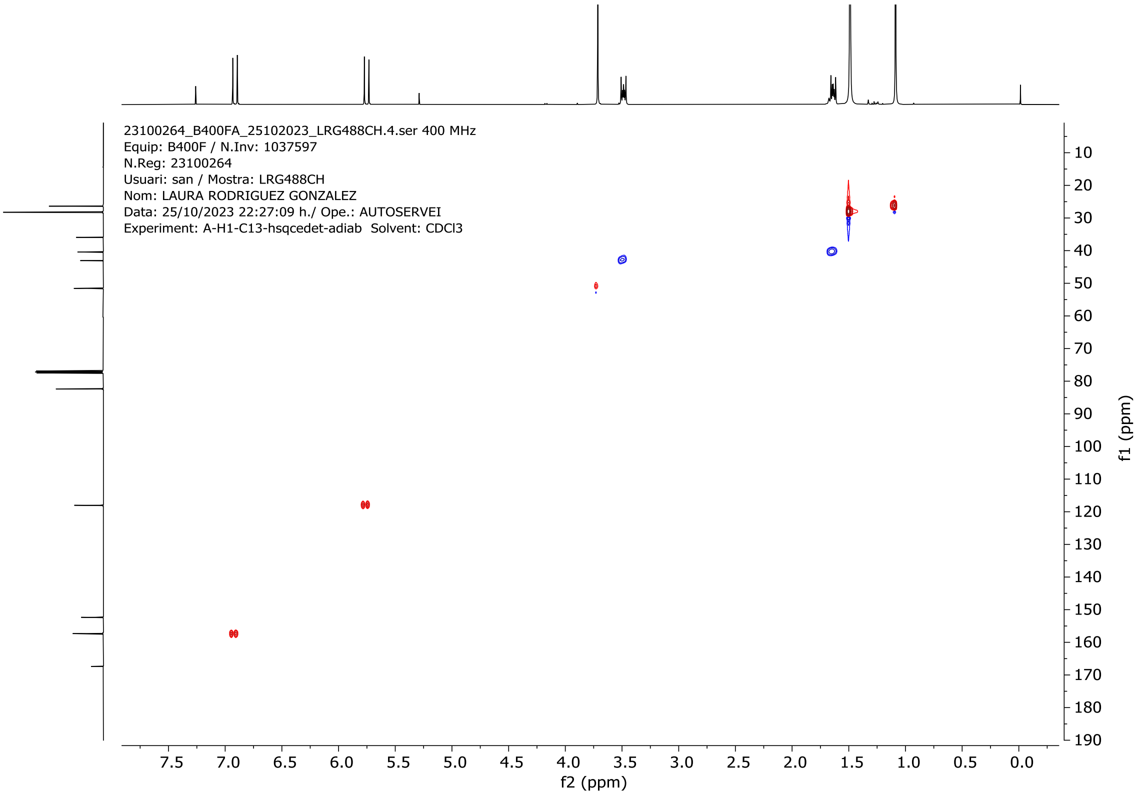

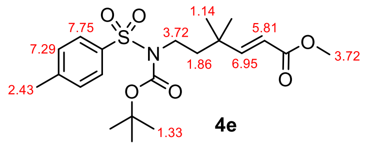

4e

23100314_B400FA_26102023_LRG492CH.1.fid 1H 400 MHz
Equip: B400F / N.Inv: 1037597
N.Reg: 23100314
Usuari: san / Mostra: LRG492CH
Nom: LAURA RODRIGUEZ GONZALEZ
Data: 26/10/2023 13:26:45 h./ Ope.: AUTOSERVEI
Experiment: A-H1-zg30 Solvent: CDCl3

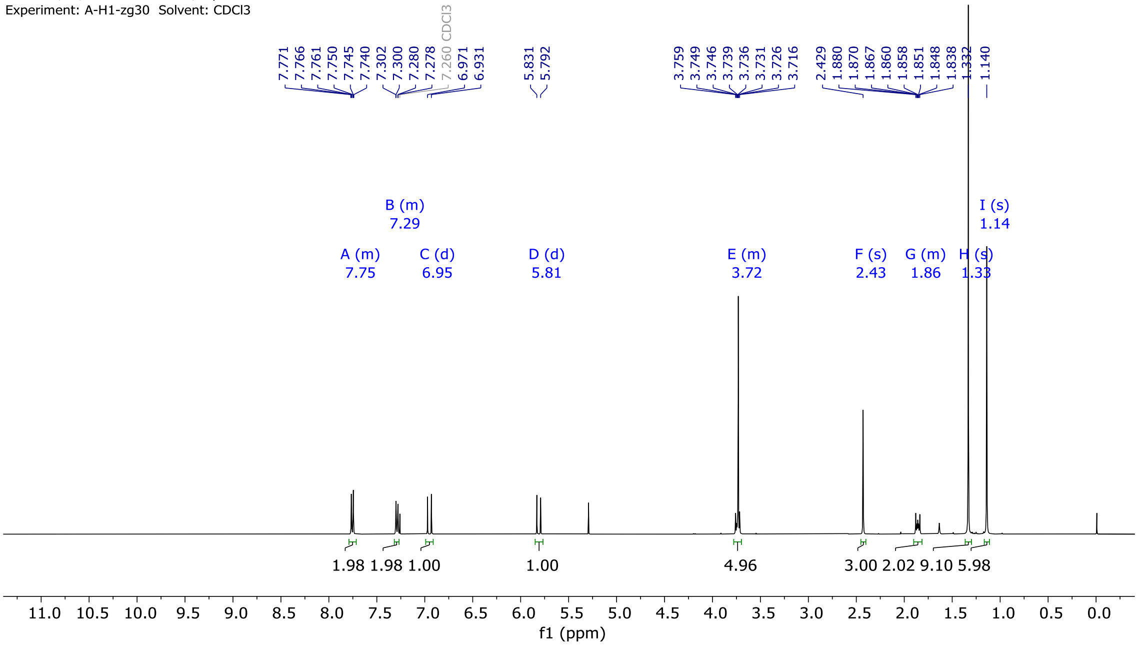

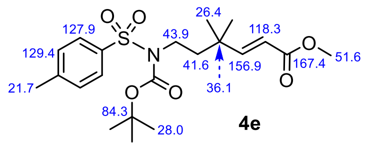

4e

23100314_B400FA_27102023_LRG492CH.2.fid 13C{1H} 101 MHz
Equip: B400F / N.Inv: 1037597
N.Reg: 23100314
Usuari: san / Mostra: LRG492CH
Nom: LAURA RODRIGUEZ GONZALEZ
Data: 27/10/2023 07:59:59 h./ Ope.: AUTOSERVEI
Experiment: A-C13-zgpg30 Solvent: CDCl3

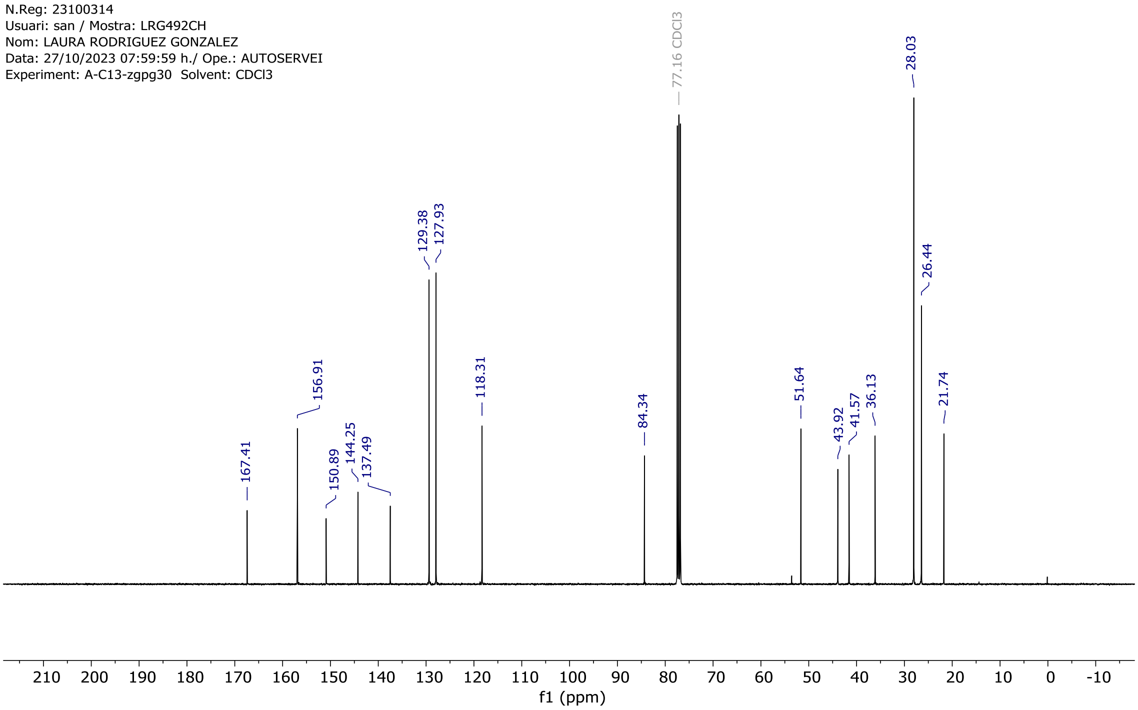

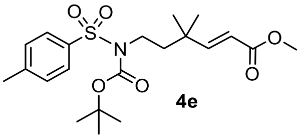

4e

2D-COSY

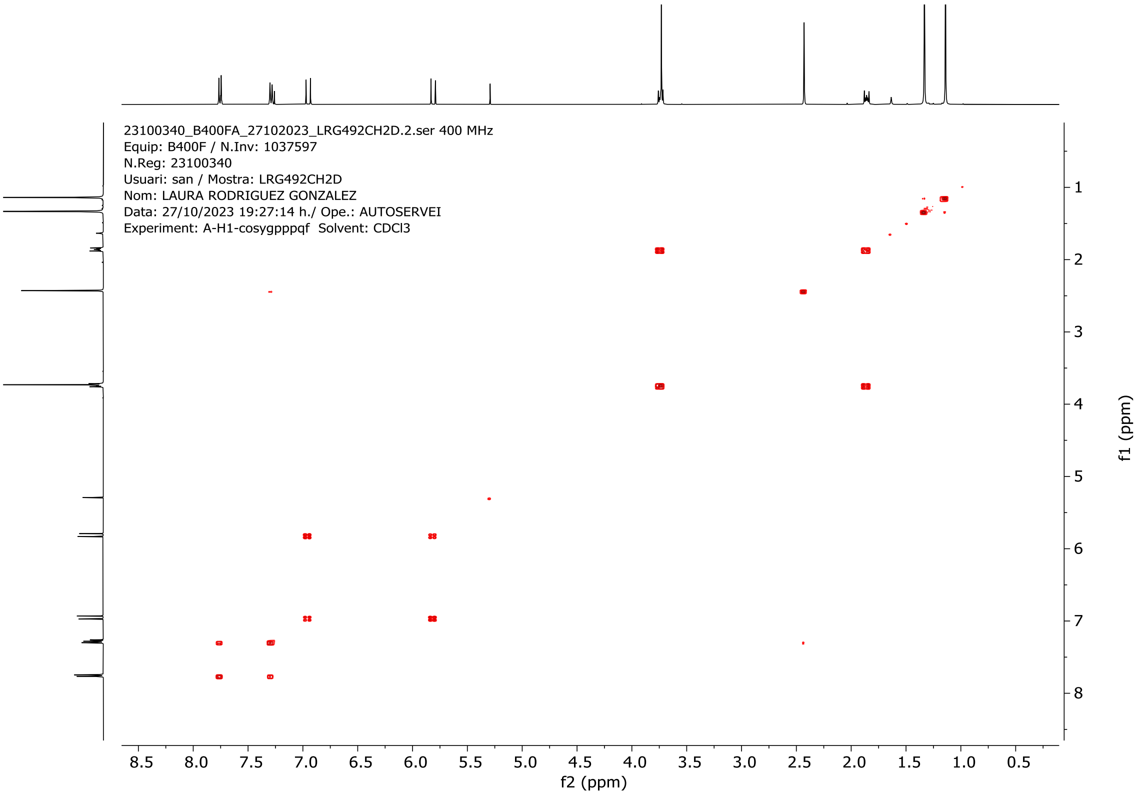

2D-HSQC

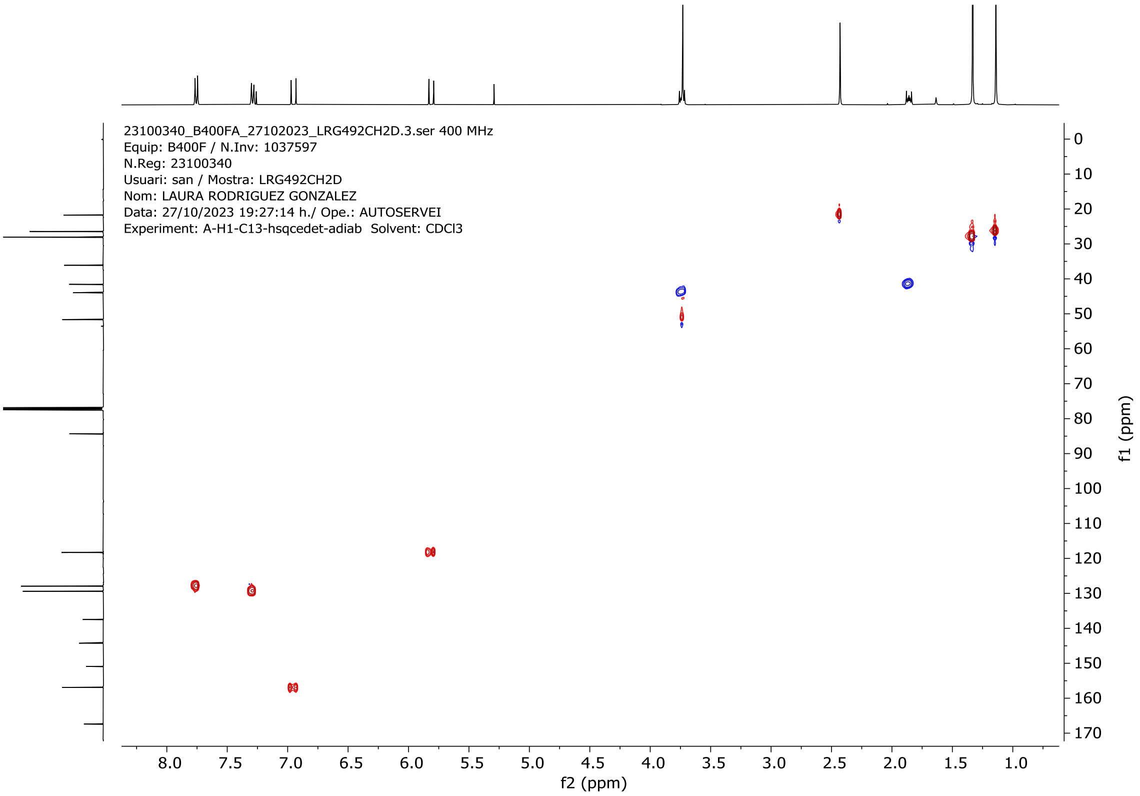

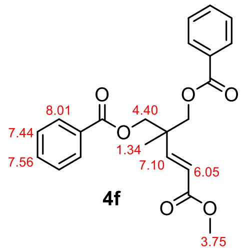

23100103_B400FA_18102023_LRG482LOW.1.fid 1H 400 MHz
Equip: B400F / N.Inv: 1037597
N.Reg: 23100103
Usuari: san / Mostra: LRG482LOW
Nom: LAURA RODRIGUEZ GONZALEZ
Data: 18/10/2023 18:37:07 h./ Ope.: AUTOSERVEI
Experiment: A-H1-zg30 Solvent: CDCl3

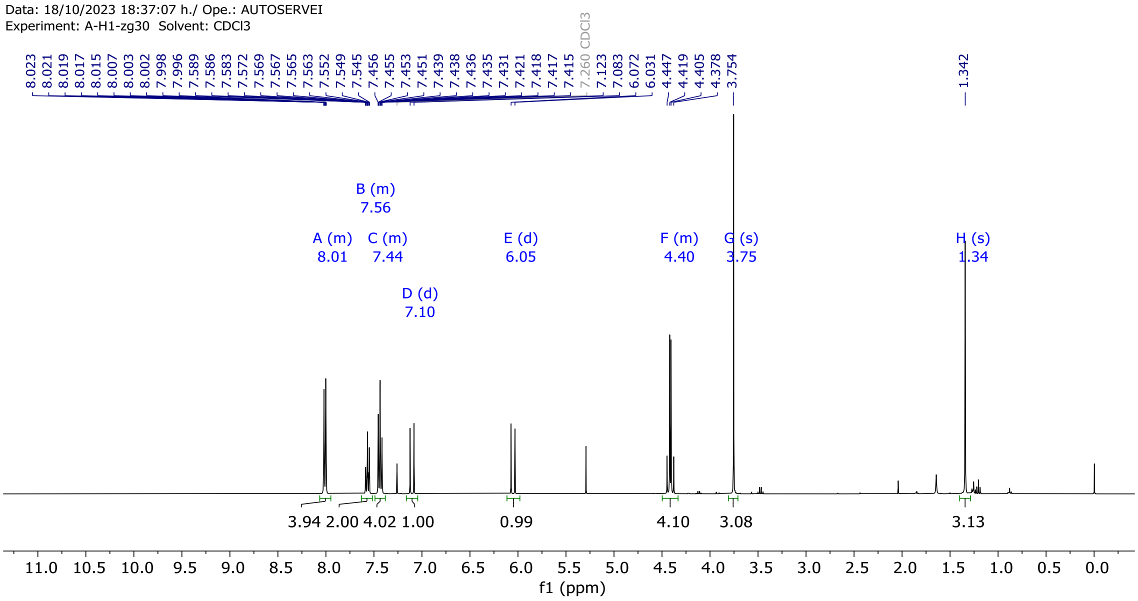

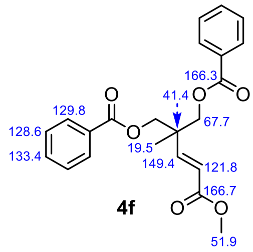

23100056_B400FA_18102023_LRG482CH.2.fid 13C{1H} 101 MHz
Equip: B400F / N.Inv: 1037597
N.Reg: 23100056
Usuari: san / Mostra: LRG482CH
Nom: LAURA RODRIGUEZ GONZALEZ
Data: 18/10/2023 21:16:46 h./ Ope.: AUTOSERVEI
Experiment: A-C13-zgpg30 Solvent: CDCl3

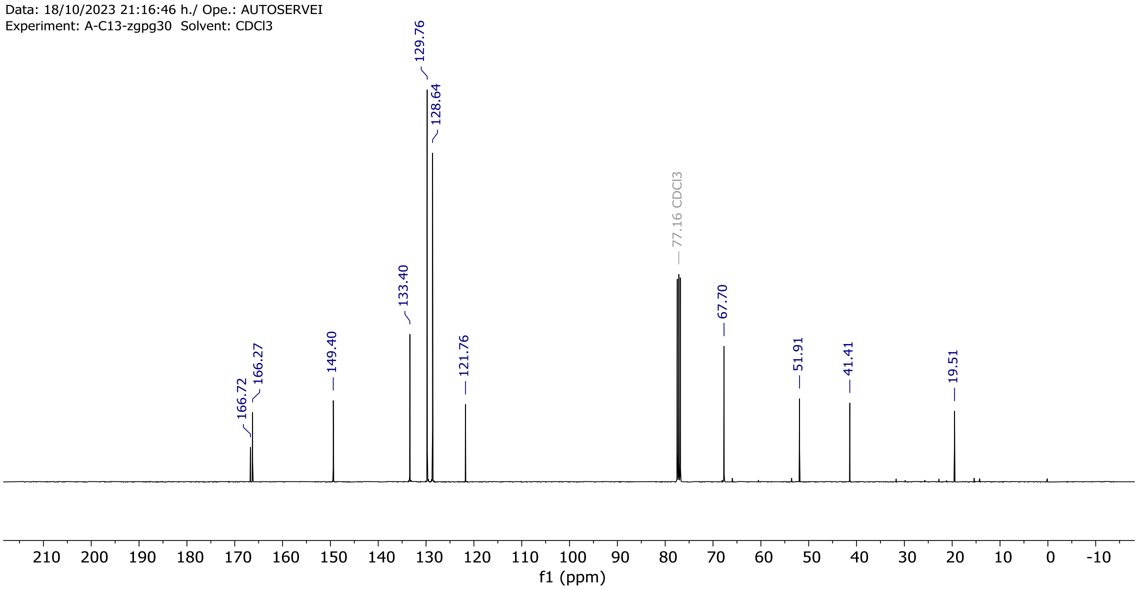

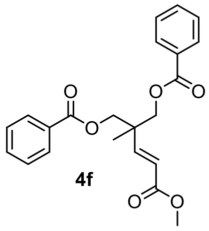

4f

2D-COSY

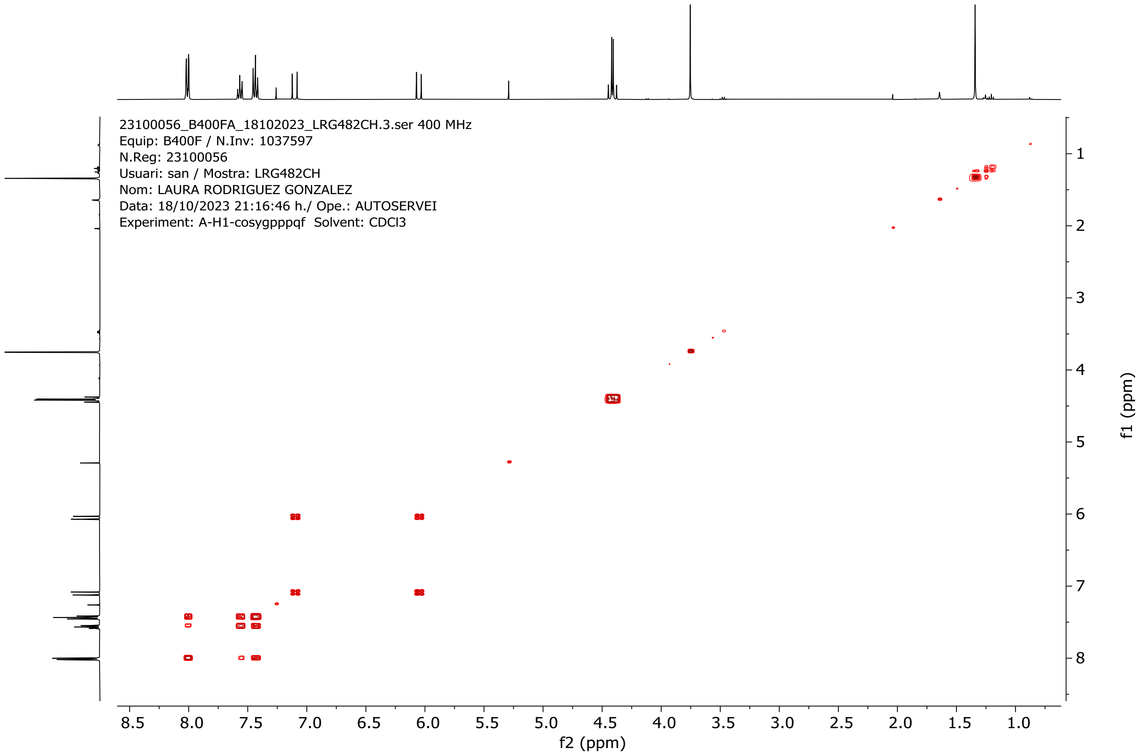

2D-HSQC

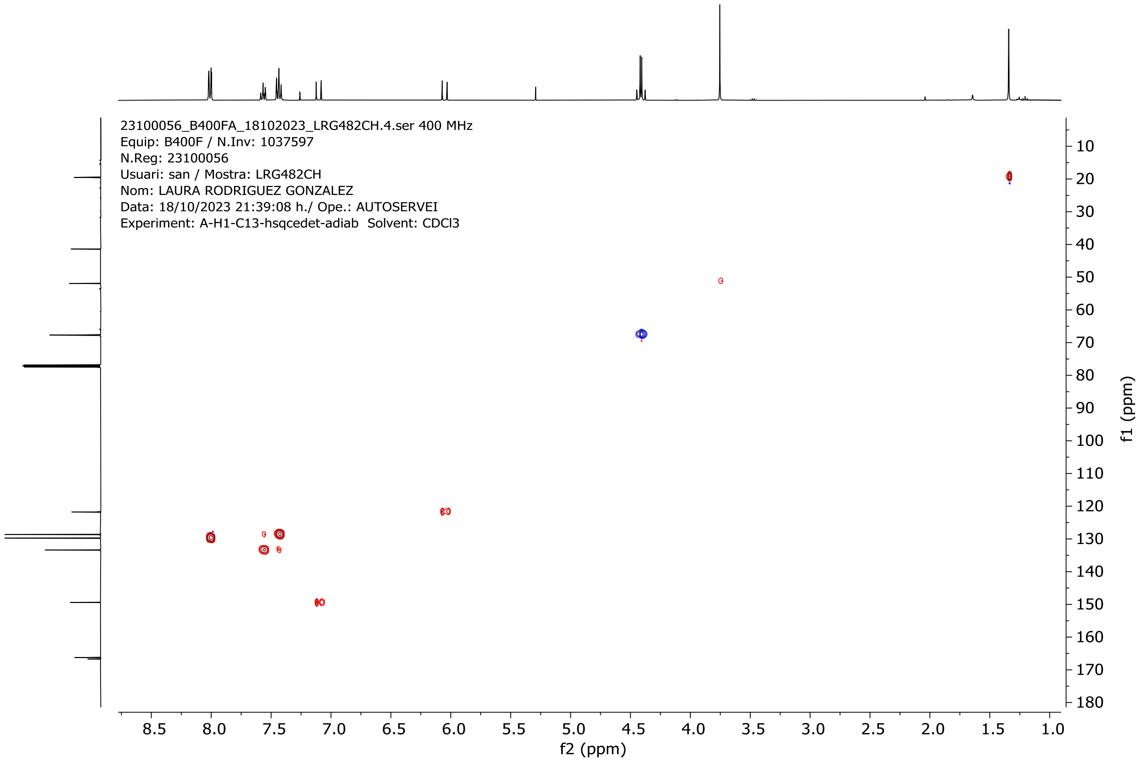

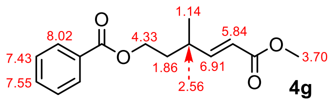

4g

23100133_B400FA_19102023_LRG485COLT25T27.1.fid 1H 400 MHz
Equip: B400F / N.Inv: 1037597
N.Reg: 23100133
Usuari: san / Mostra: LRG485COLT25T27
Nom: LAURA RODRIGUEZ GONZALEZ
Data: 19/10/2023 16:14:53 h./ Ope.: AUTOSERVEI
Experiment: A-H1-zg30 Solvent: CDCl3

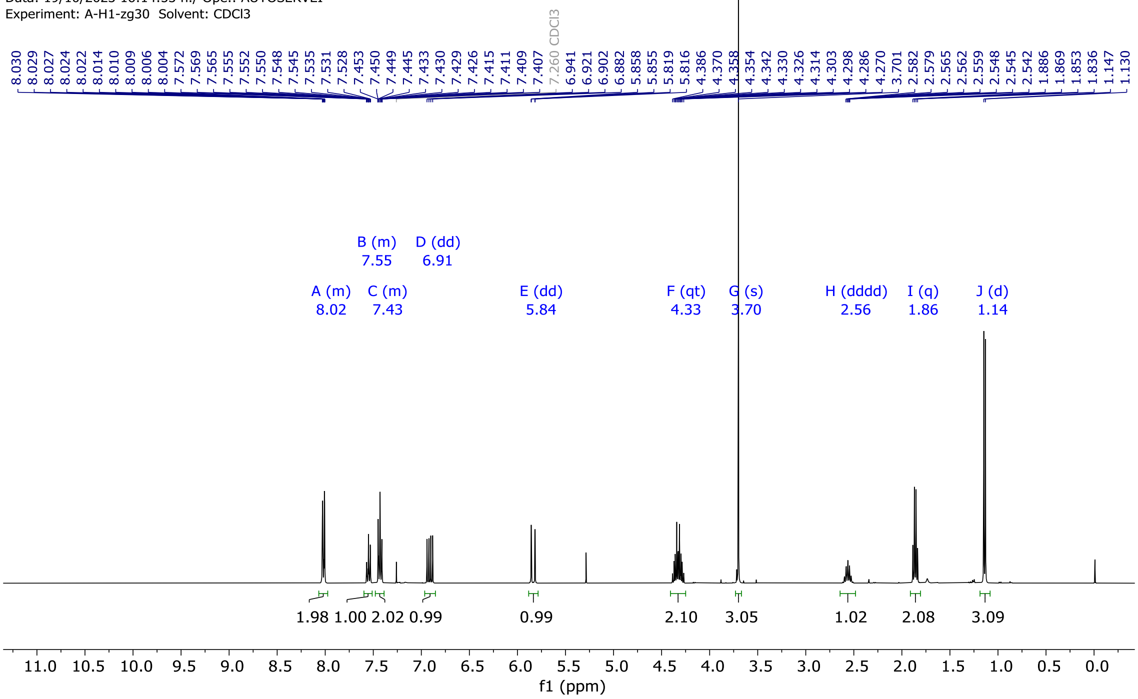

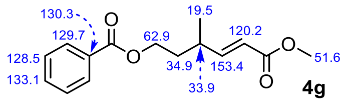

4g

23100121_B400FA_19102023_LRG484CH.2.fid 13C{1H} 101 MHz
Equip: B400F / N.Inv: 1037597
N.Reg: 23100121
Usuari: san / Mostra: LRG484CH
Nom: LAURA RODRIGUEZ GONZALEZ
Data: 19/10/2023 21:08:47 h./ Ope.: AUTOSERVEI
Experiment: A-C13-zgpg30 Solvent: CDCl3

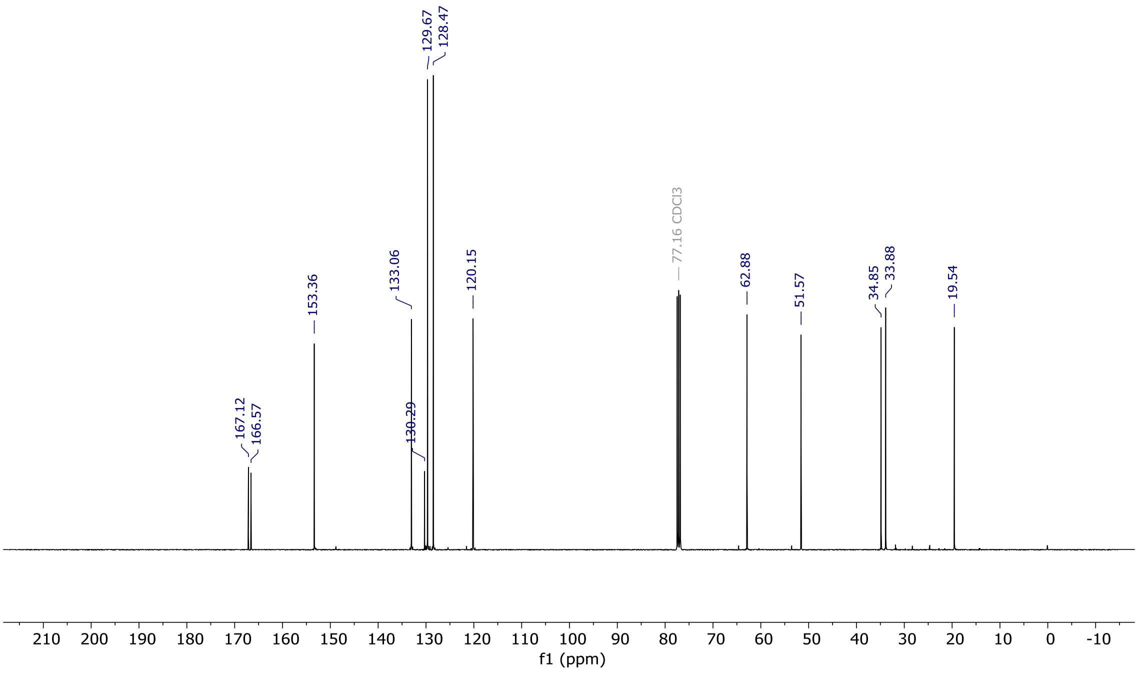

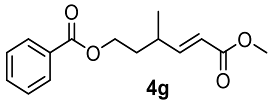

4g

2D-COSY

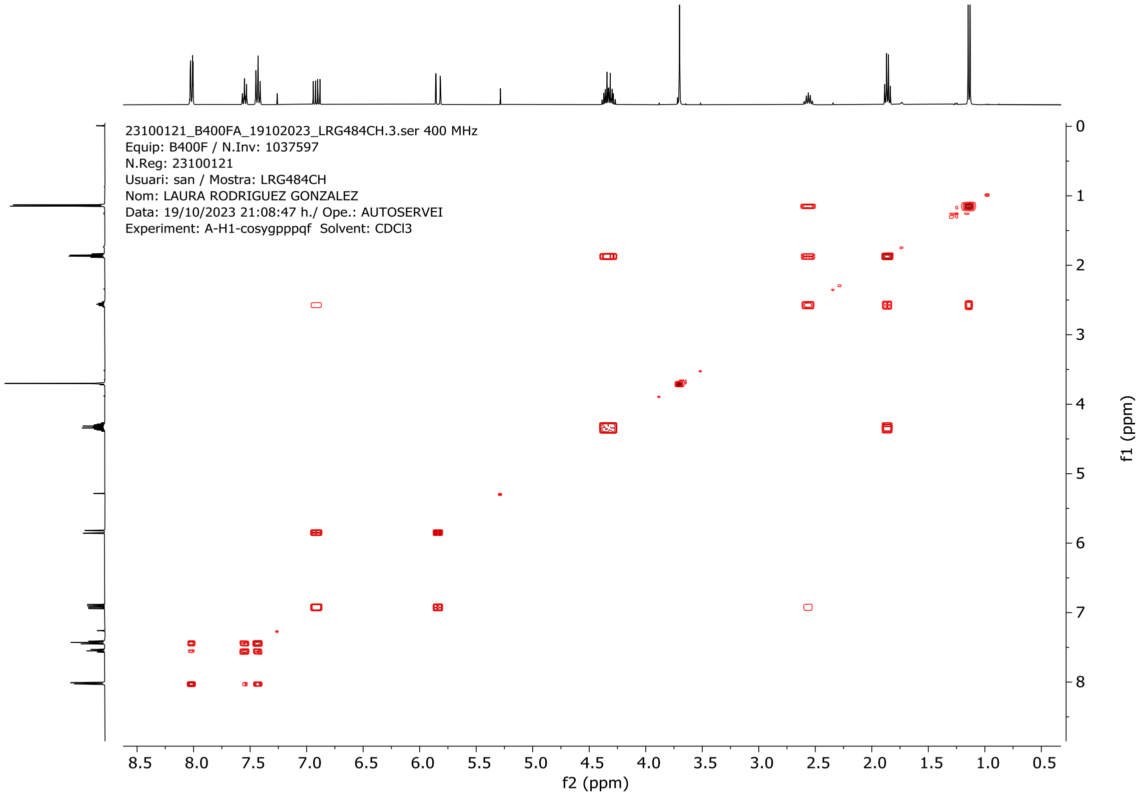

2D-HSQC

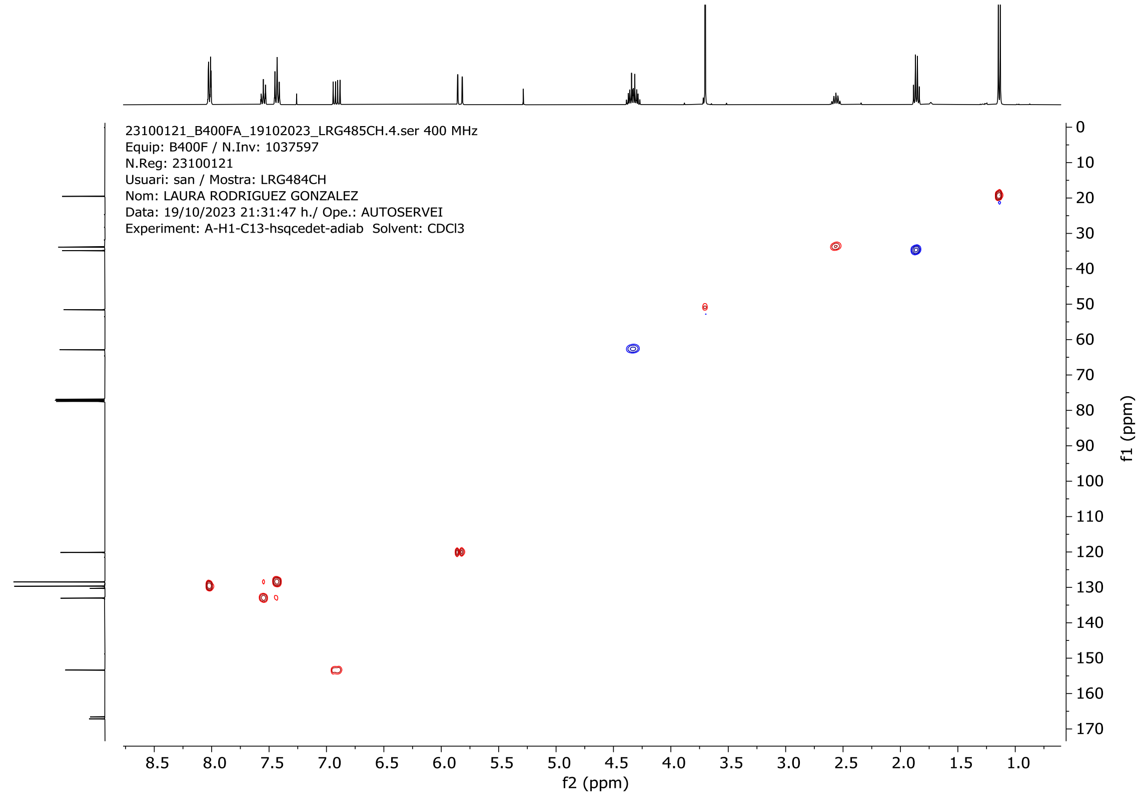

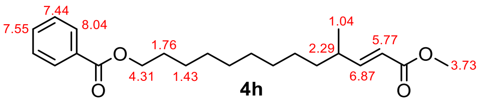

5488-2023_B500QA_10102023_LRG476COLT24T25.10.fid 1H 500 MHz
Equip: B500Q / N.Inv: 1028917
N.Reg: 5488/2023
Usuari: san / Mostra: LRG476COLT24T25
Nom: LAURA RODRIGUEZ GONZALEZ
Data: 10/10/2023 11:07:42 h./ Ope.: servei Unitat RMN
Experiment: A_1H-zg30 Solvent: CDCl3 Operator: DANIEL PUIG

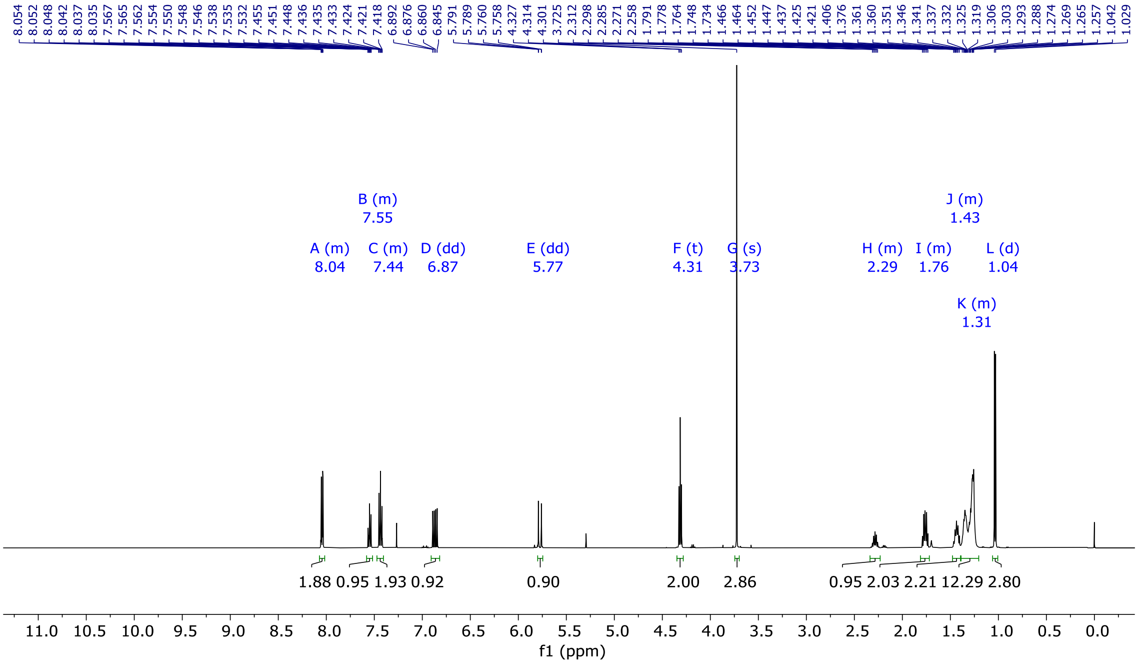

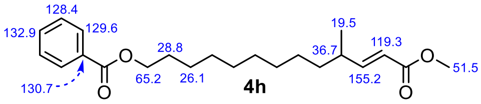

san-5488-2023.2.fid 13C{1H} 101 MHz
Equip: B400Q / N.Inv: 1035091
N.Reg: 5488/2023
Usuari: san / Mostra: LRG476COLT24T25
Nom: LAURA RODRIGUEZ GONZALEZ
Data: 10/10/2023 17:03:23 h./ Ope.: servei Unitat RMN
Experiment: A_13C-zgpg30 Solvent: CDCl3 Operator: VICTOR MERIEL

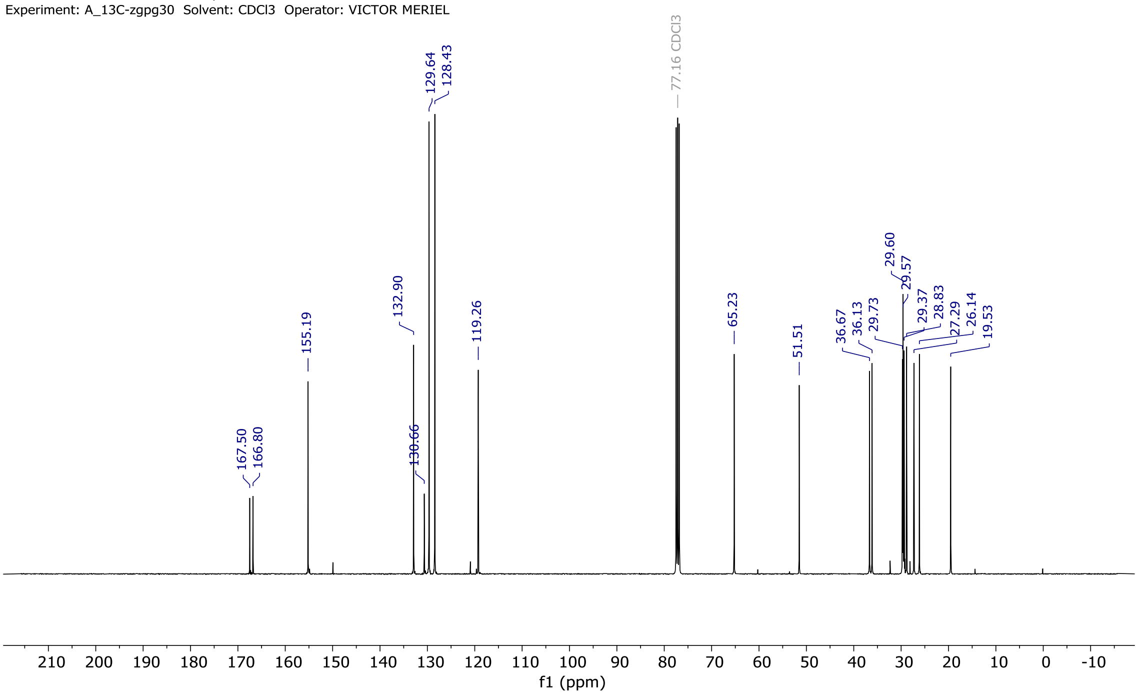

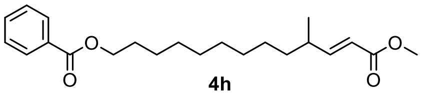

4h

2D-COSY

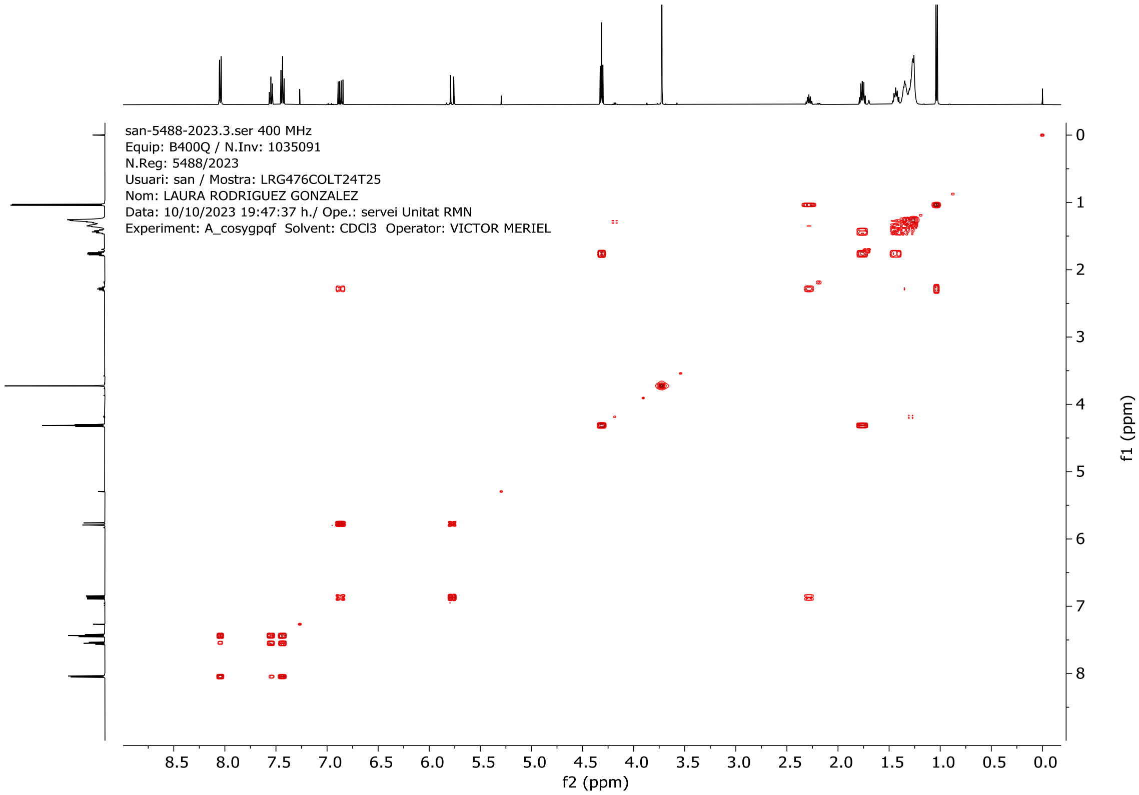

2D-HSQC

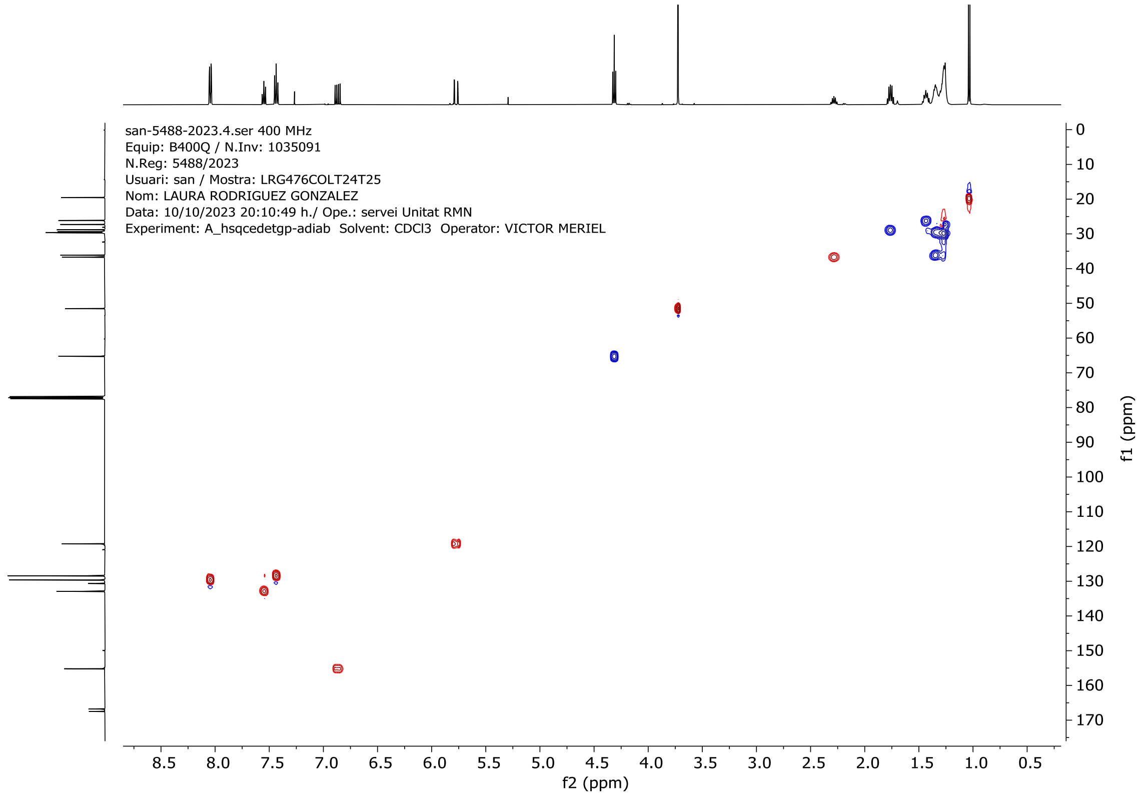

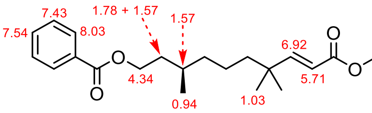

4i

23100356_B400FA_27102023_LRG495COLT16T18.1.fid 1H 400 MHz
Equip: B400F / N.Inv: 1037597
N.Reg: 23100356
Usuari: san / Mostra: LRG495COLT16T18
Nom: LAURA RODRIGUEZ GONZALEZ
Data: 27/10/2023 12:07:07 h./ Ope.: AUTOSERVEI
Experiment: A-H1-zg30 Solvent: CDCl3

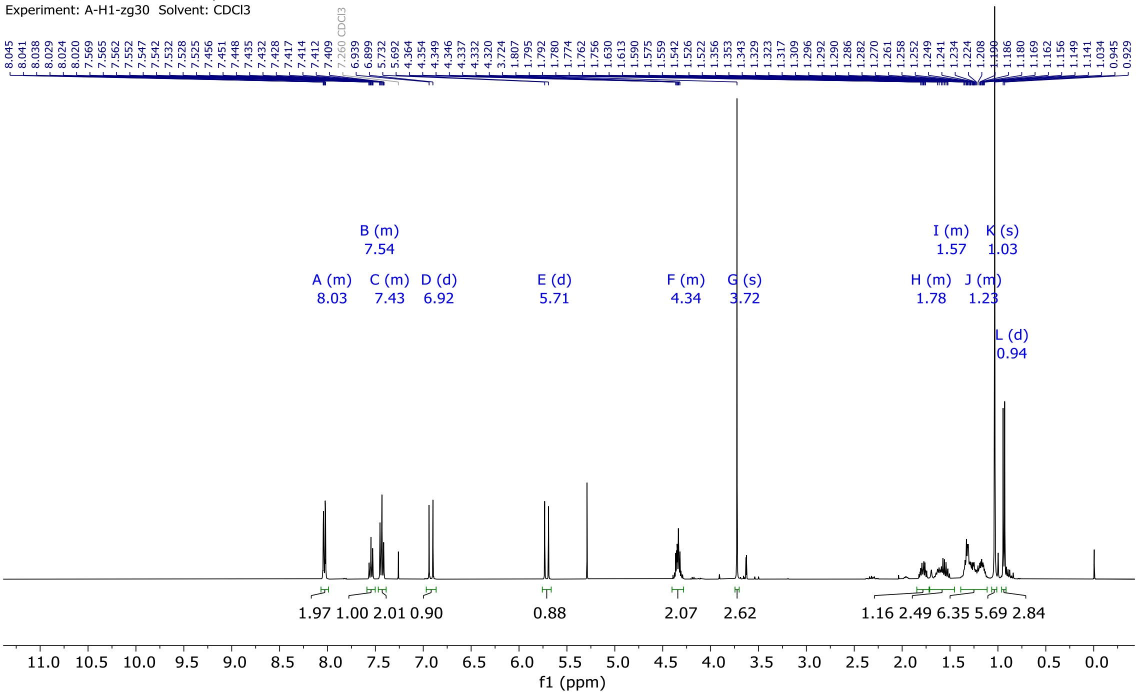

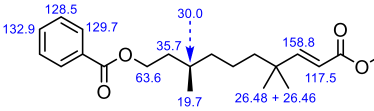

4i

23100341_B400FA_27102023_LRG495CH.2.fid 13C{1H} 101 MHz
Equip: B400F / N.Inv: 1037597
N.Reg: 23100341
Usuari: san / Mostra: LRG495CH
Nom: LAURA RODRIGUEZ GONZALEZ
Data: 27/10/2023 22:32:02 h./ Ope.: AUTOSERVEI
Experiment: A-C13-zgpg30 Solvent: CDCl3

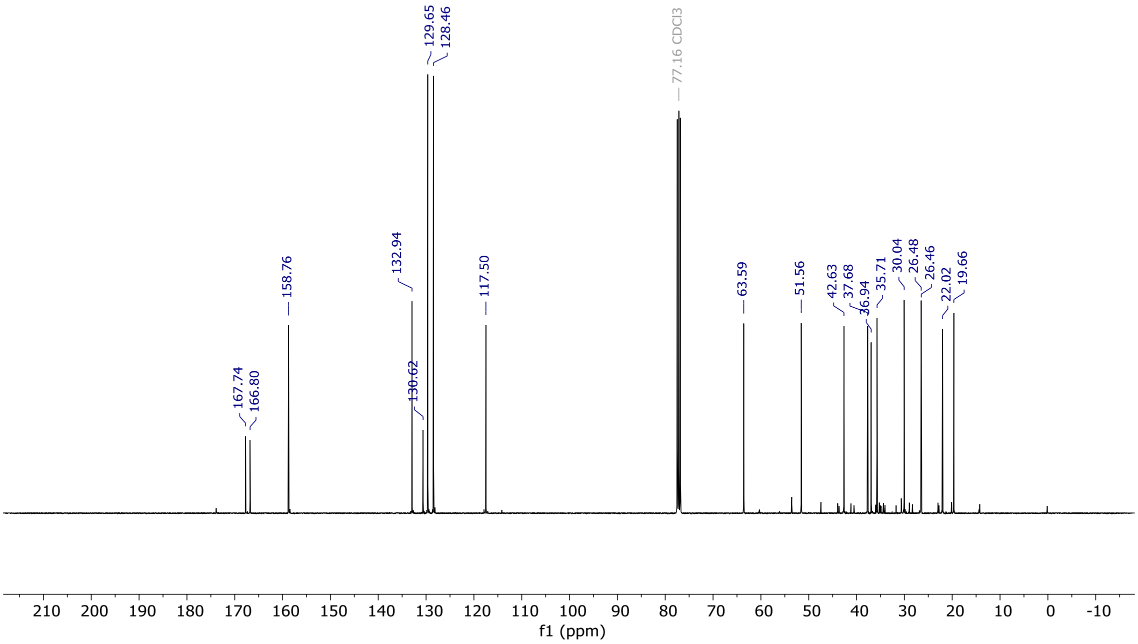

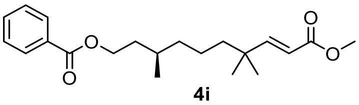

4i

2D-COSY

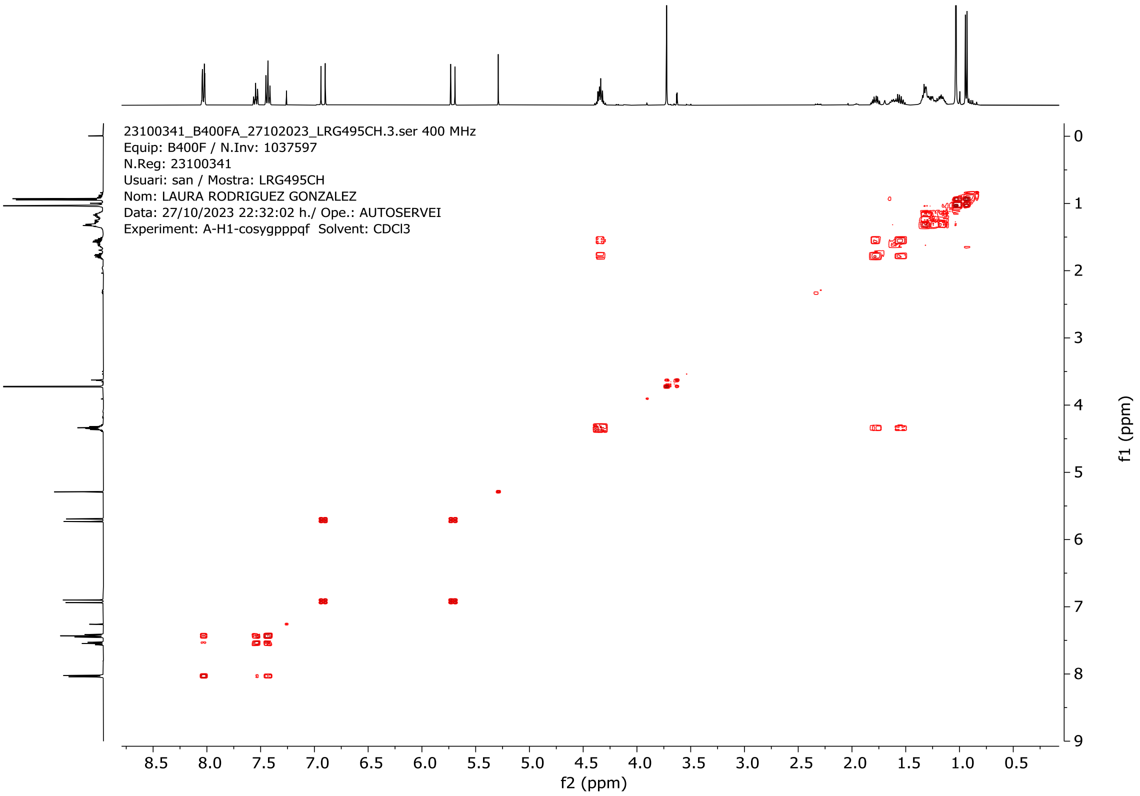

2D-HSQC

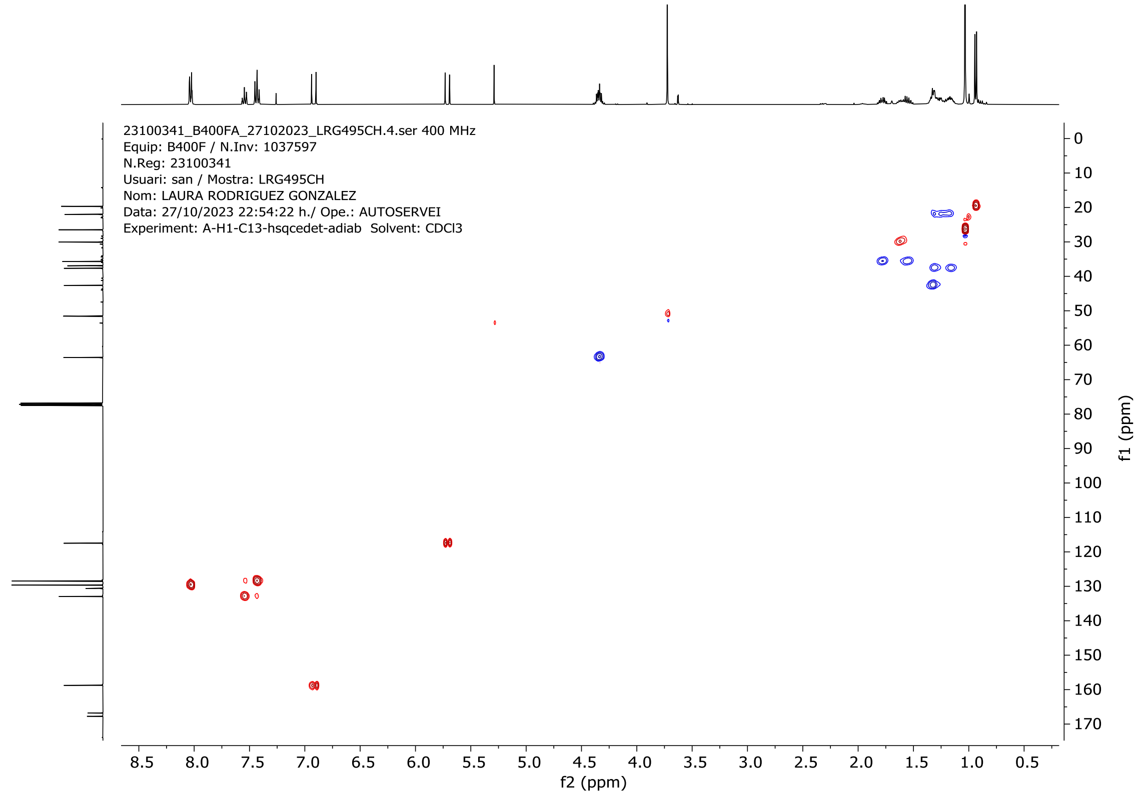

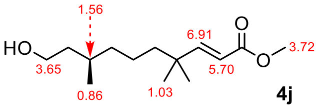

23110776_B400FA_28112023_LRG509COLT37T39.1.fid 1H 400 MHz
Equip: B400F / N.Inv: 1037597
N.Reg: 23110776
Usuari: san / Mostra: LRG509COLT37T39
Nom: LAURA RODRIGUEZ GONZALEZ
Data: 28/11/2023 12:06:30 h./ Ope.: AUTOSERVEI
Experiment: A-H1-zg30 Solvent: CDCl3

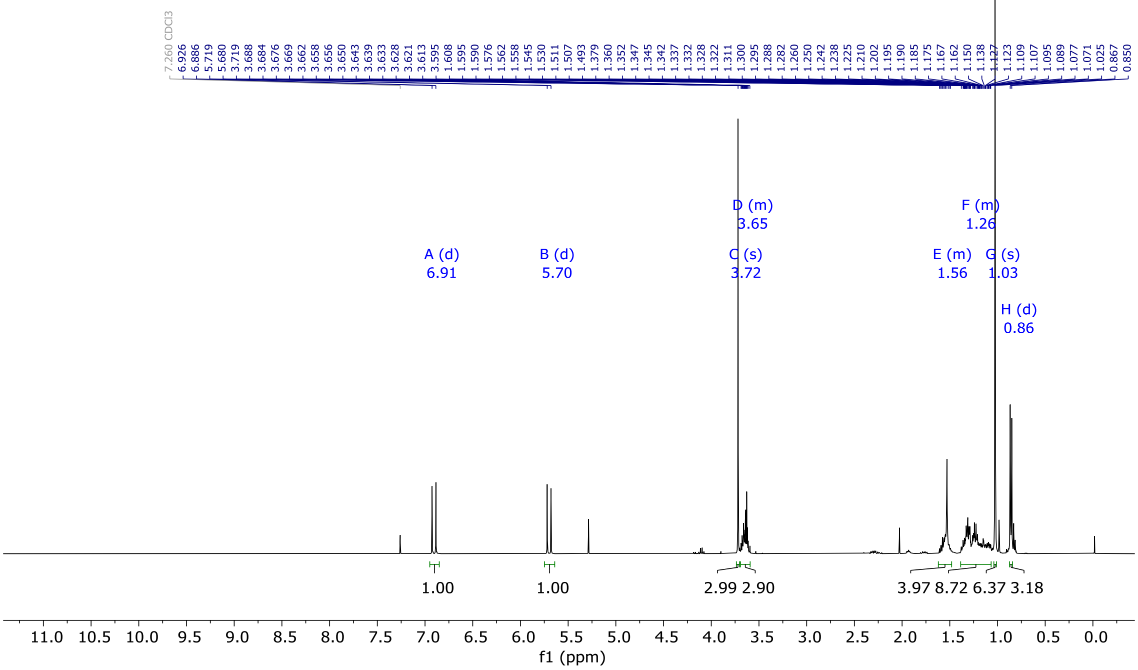

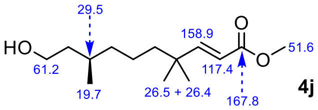

23110760_B400FA_28112023_LRG509CH.2.fid 13C{1H} 101 MHz
Equip: B400F / N.Inv: 1037597
N.Reg: 23110760
Usuari: san / Mostra: LRG509CH
Nom: LAURA RODRIGUEZ GONZALEZ
Data: 28/11/2023 21:07:53 h./ Ope.: AUTOSERVEI
Experiment: A-C13-zgpg30 Solvent: CDCl3

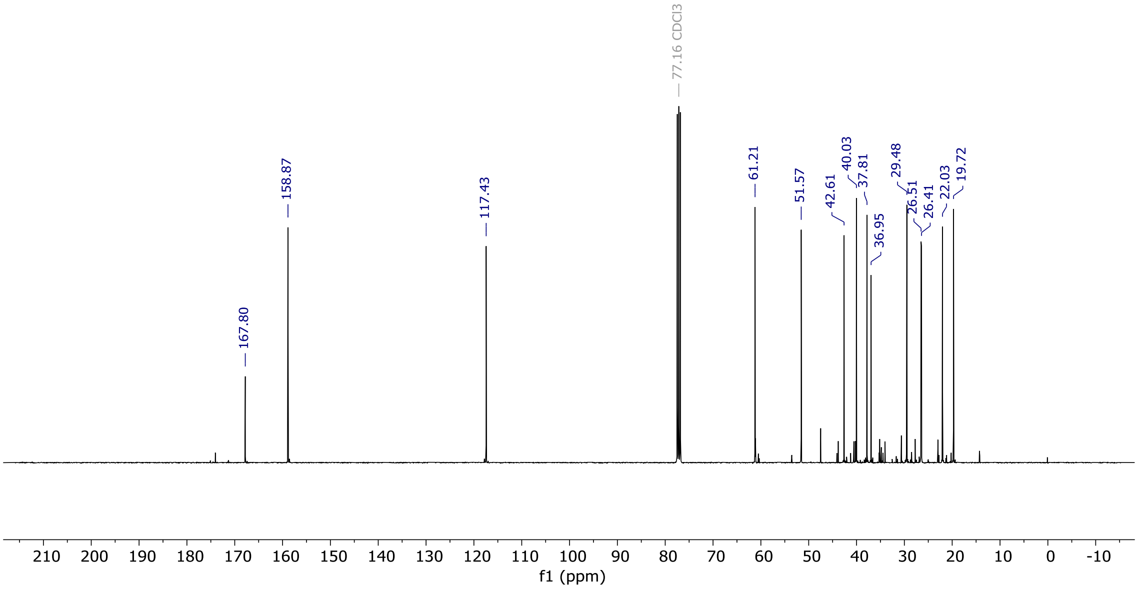

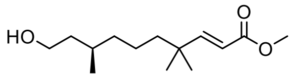

4j

2D-COSY

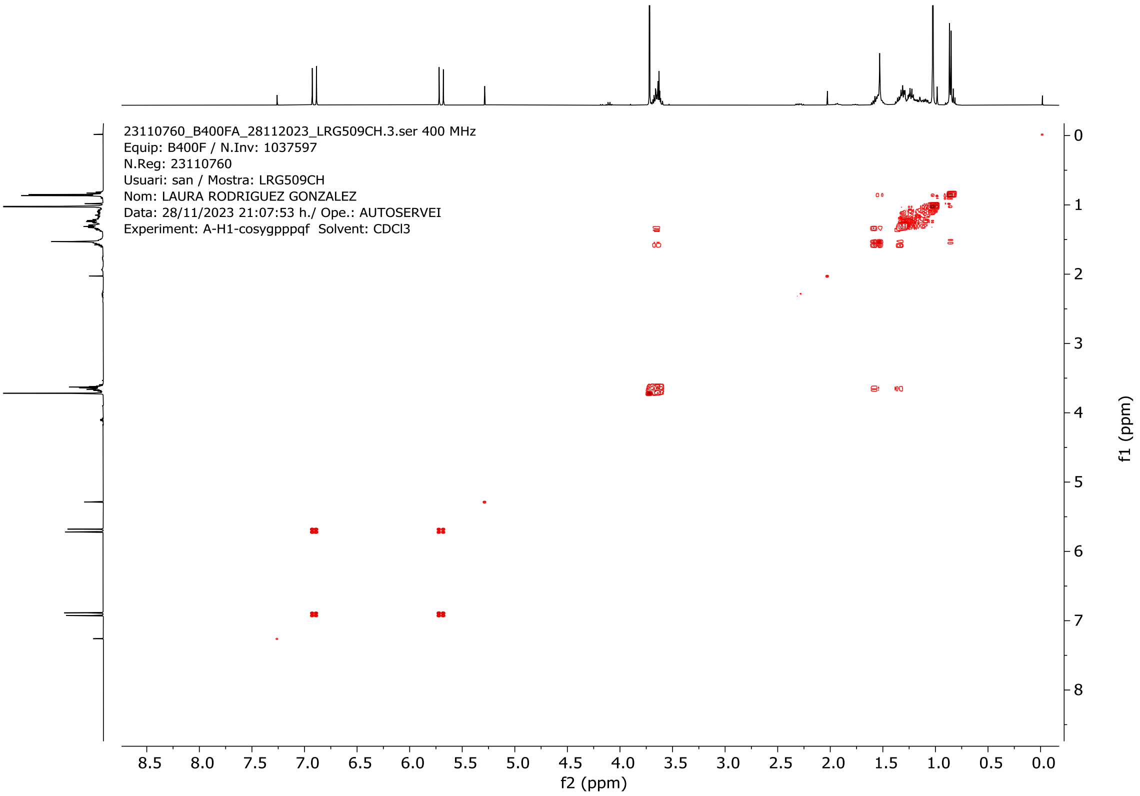

2D-HSQC

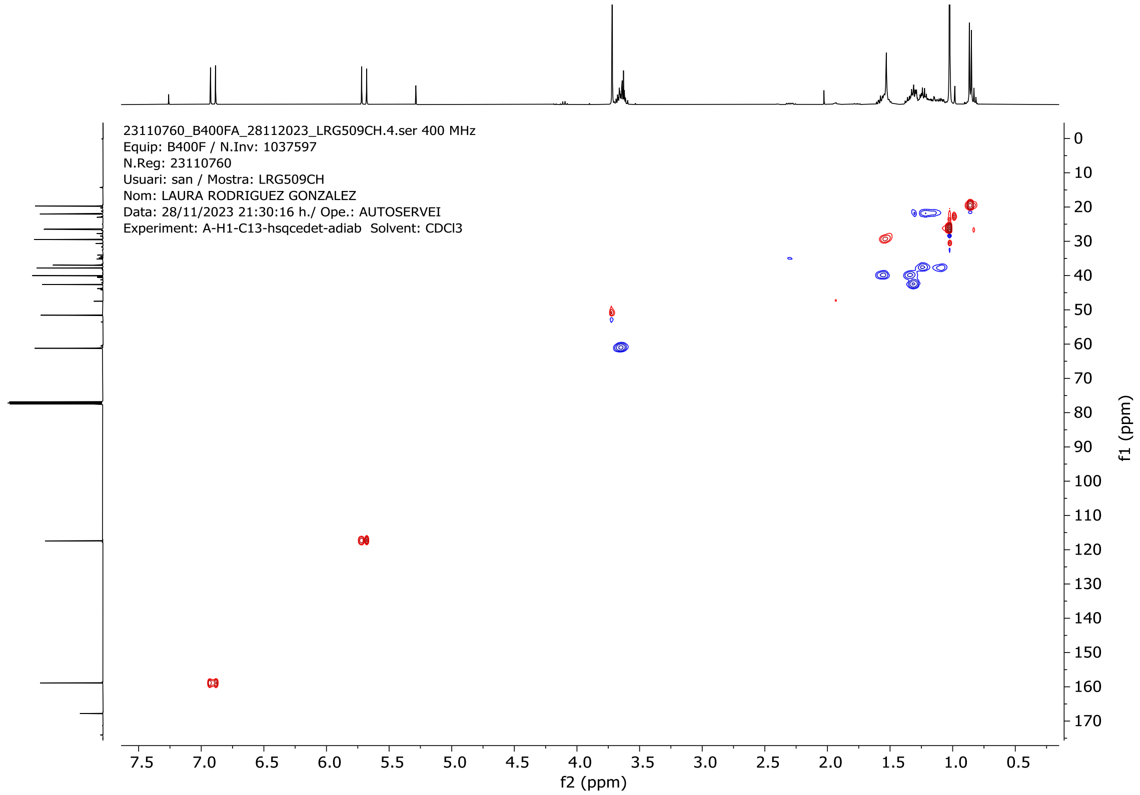

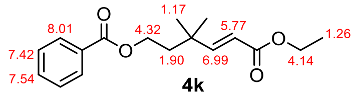

san-5030-2023.1.fid 1H 400 MHz
Equip: B400Q / N.Inv: 1035091
N.Reg: 5030/2023
Usuari: san / Mostra: LRG470PROD
Nom: LAURA RODRIGUEZ GONZALEZ
Data: 02/10/2023 15:33:59 h./ Ope.: servei Unitat RMN
Experiment: A_1H-zg30 Solvent: CDCl3 Operator: VICTOR MERIEL

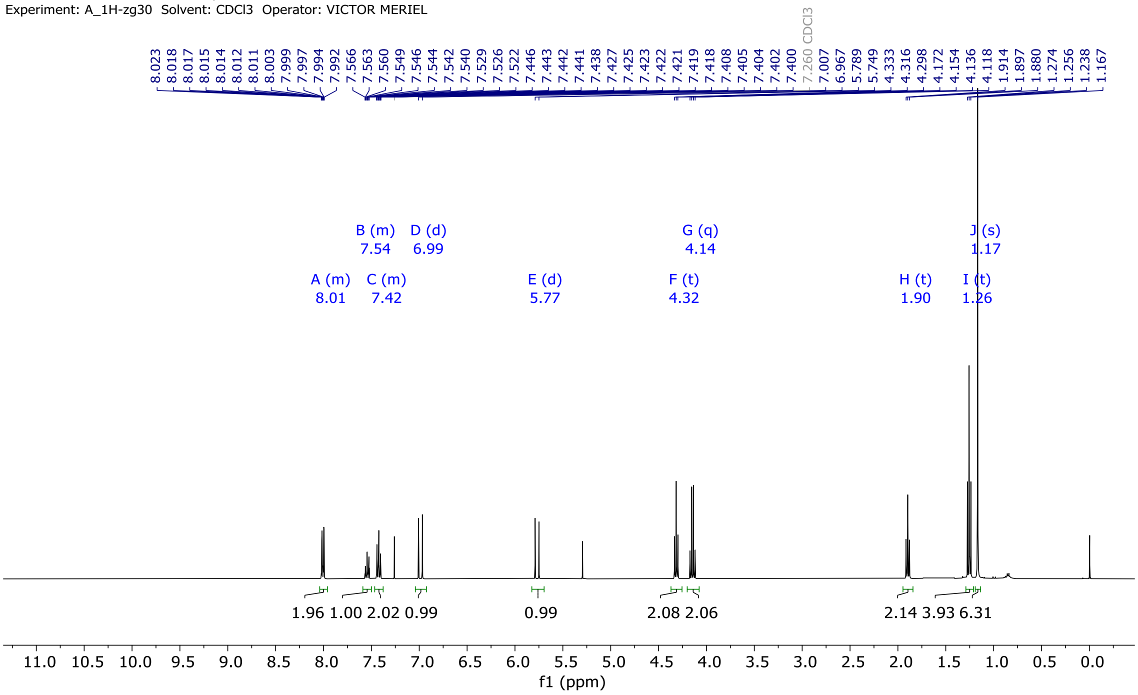

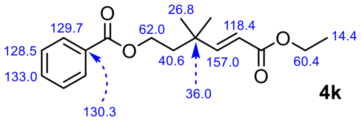

san-5030-2023.2.fid 13C{1H} 101 MHz
Equip: B400Q / N.Inv: 1035091
N.Reg: 5030/2023
Usuari: san / Mostra: LRG470PROD
Nom: LAURA RODRIGUEZ GONZALEZ
Data: 03/10/2023 03:35:44 h./ Ope.: servei Unitat RMN
Experiment: A_13C-zgpg30 Solvent: CDCl3 Operator: VICTOR MERIEL

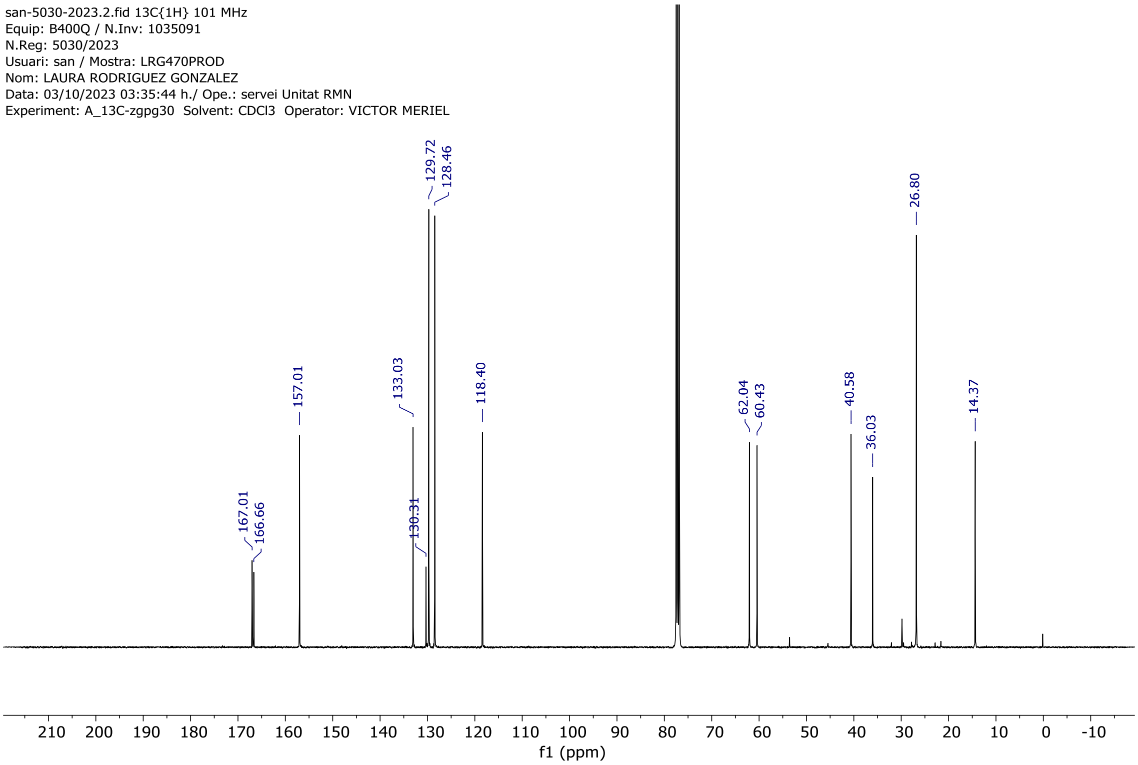

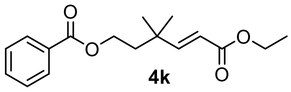

4k

2D-COSY

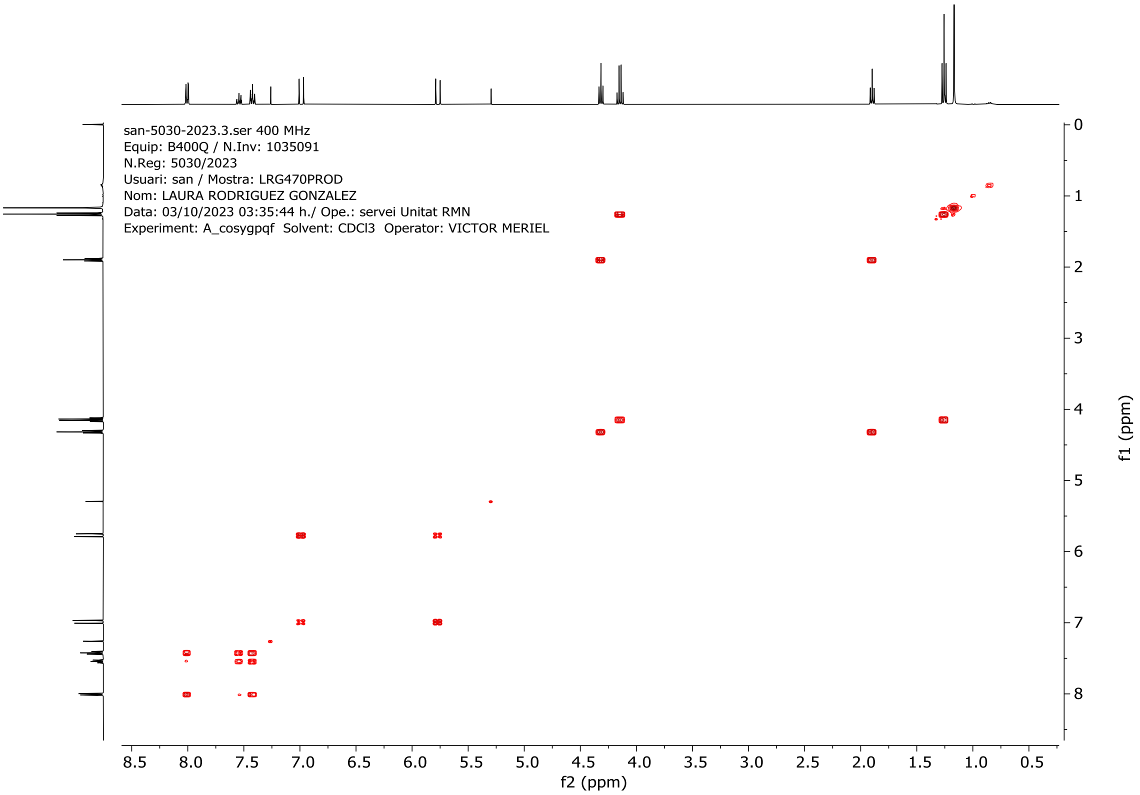

2D-HSQC

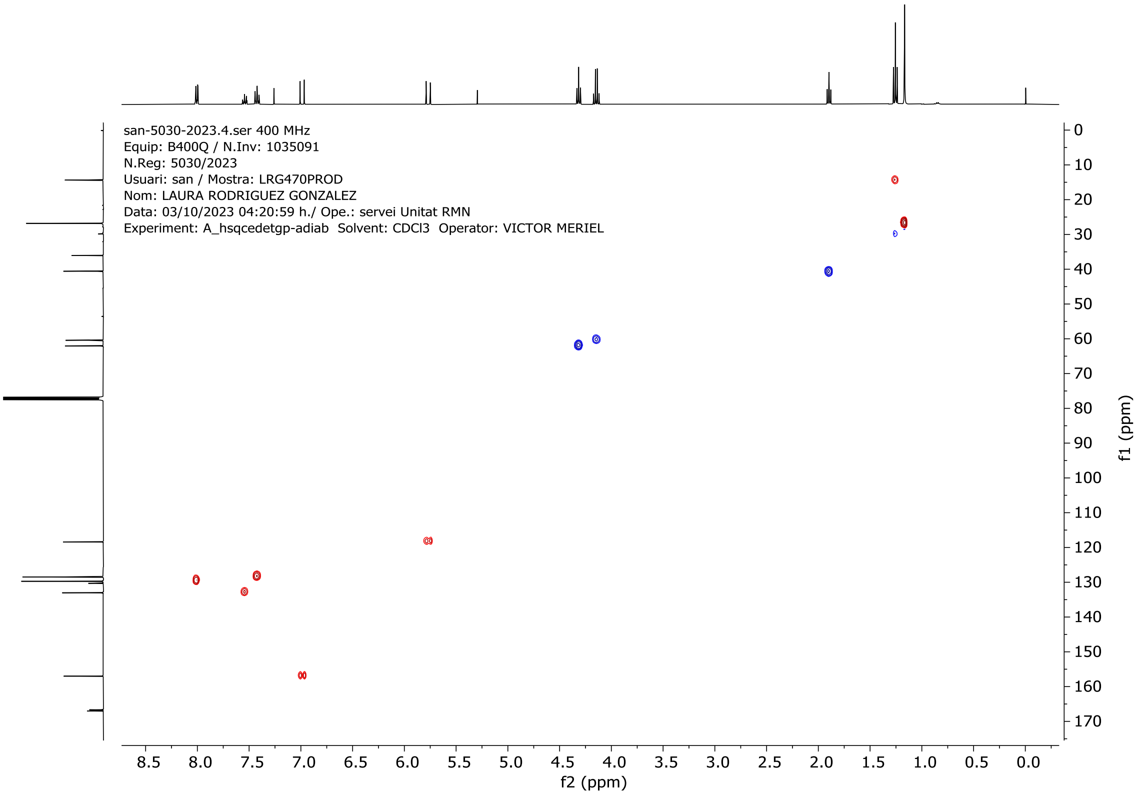

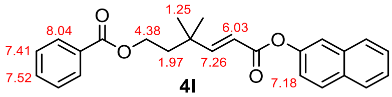

23050540_B400FA_17052023_LRG389T30T31CH.1.fid 1H 400 MHz
Equip: B400F / N.Inv: 1037597
N.Reg: 23050540
Usuari: san / Mostra: LRG389T30T31CH
Nom: LAURA RODRIGUEZ GONZALEZ
Data: 17/05/2023 14:13:42 h./ Ope.: AUTOSERVEI
Experiment: A-H1-zg30 Solvent: CDCl3

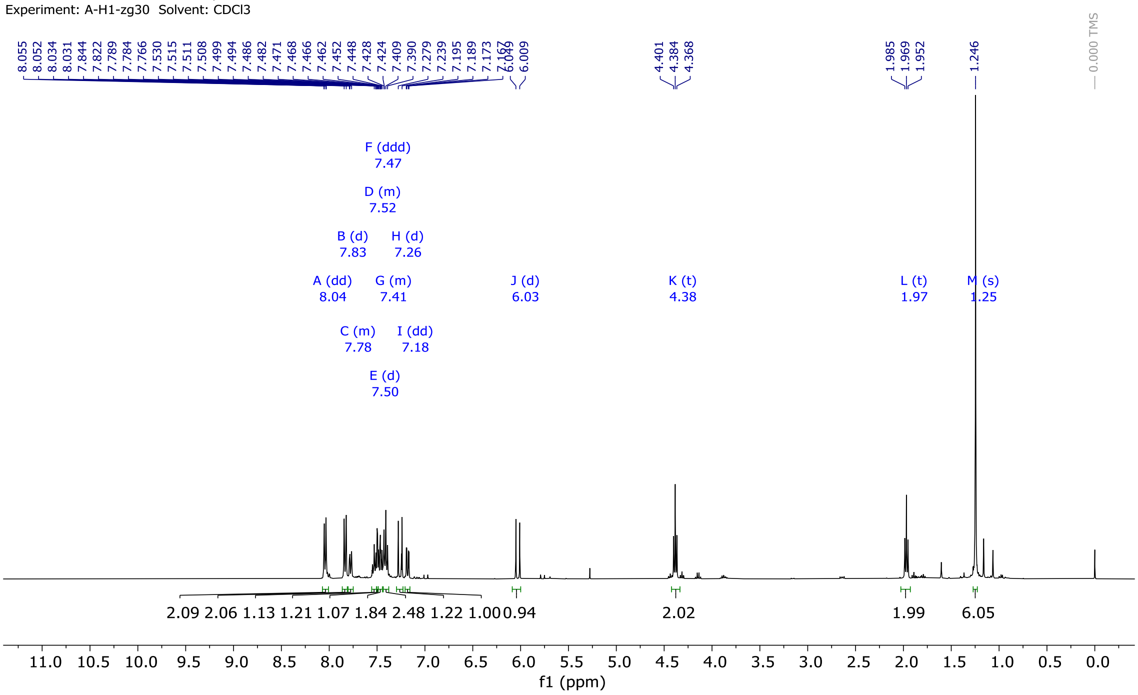

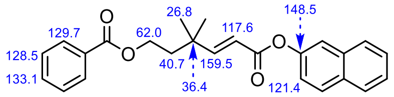

4l

23050540_B400FA_17052023_LRG389T30T31CH.2.fid 13C{1H} 101 MHz
Equip: B400F / N.Inv: 1037597
N.Reg: 23050540
Usuari: san / Mostra: LRG389T30T31CH
Nom: LAURA RODRIGUEZ GONZALEZ
Data: 17/05/2023 21:08:39 h./ Ope.: AUTOSERVEI
Experiment: A-C13-zgpg30 Solvent: CDCl3

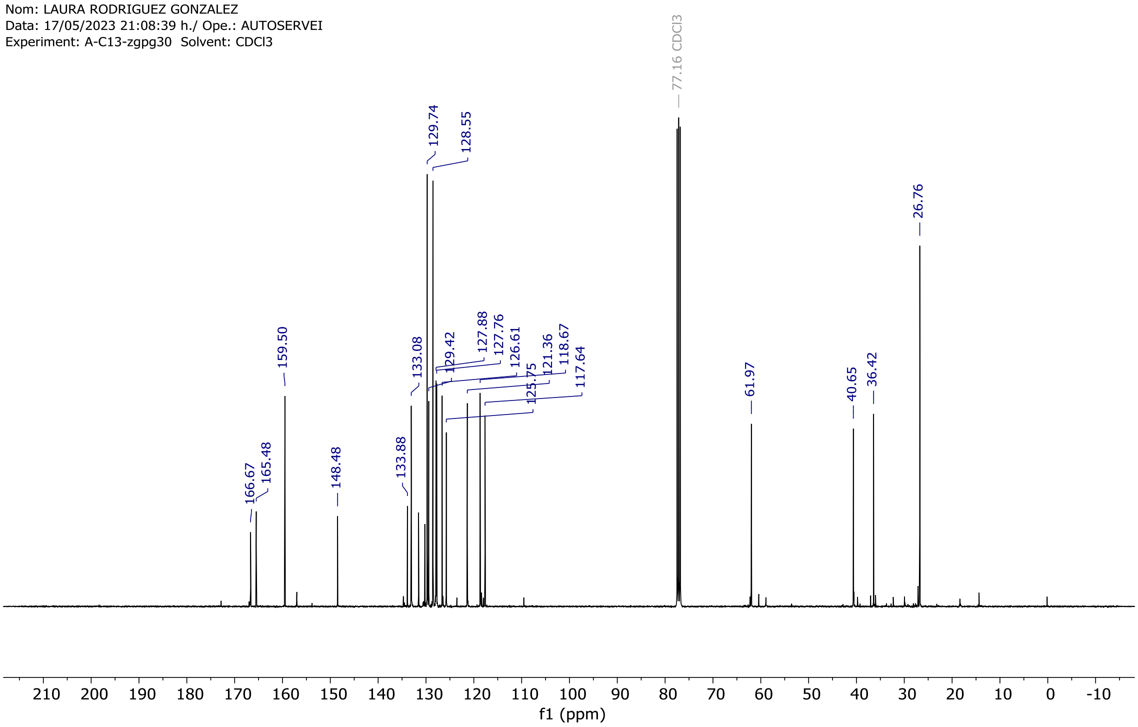

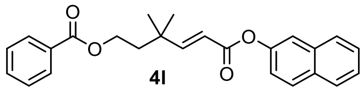

4l

2D-COSY

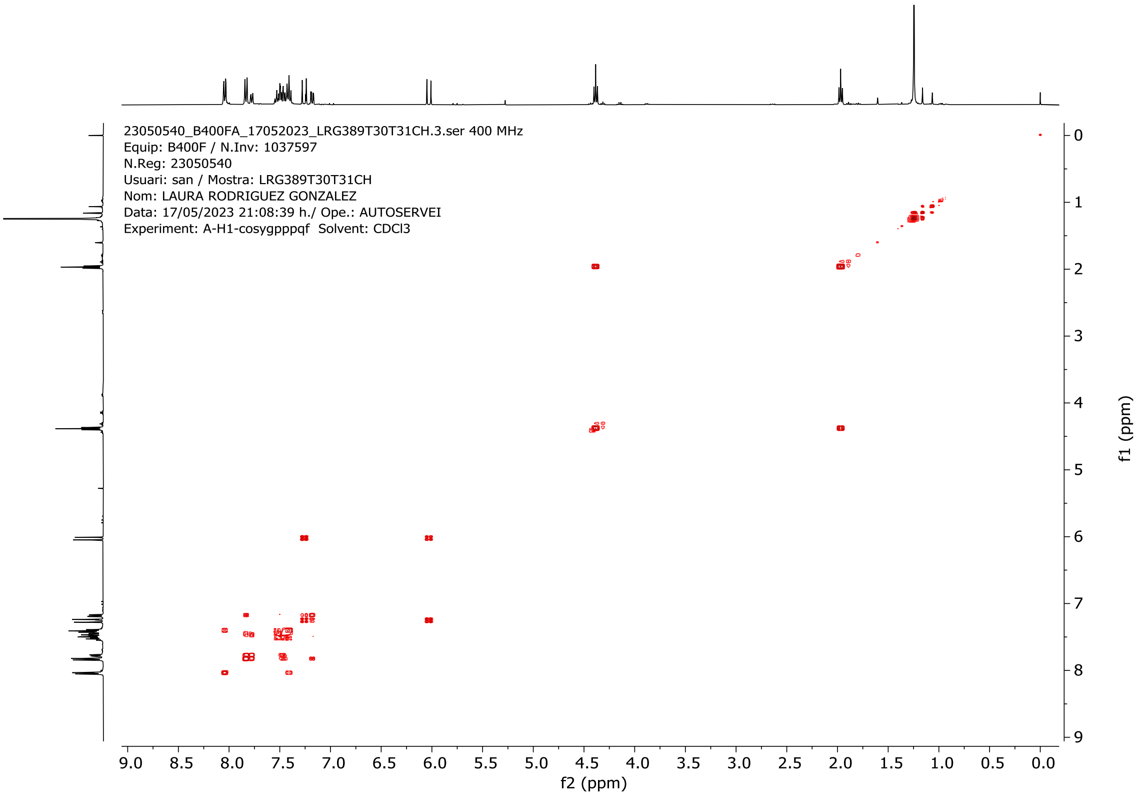

2D-HSQC

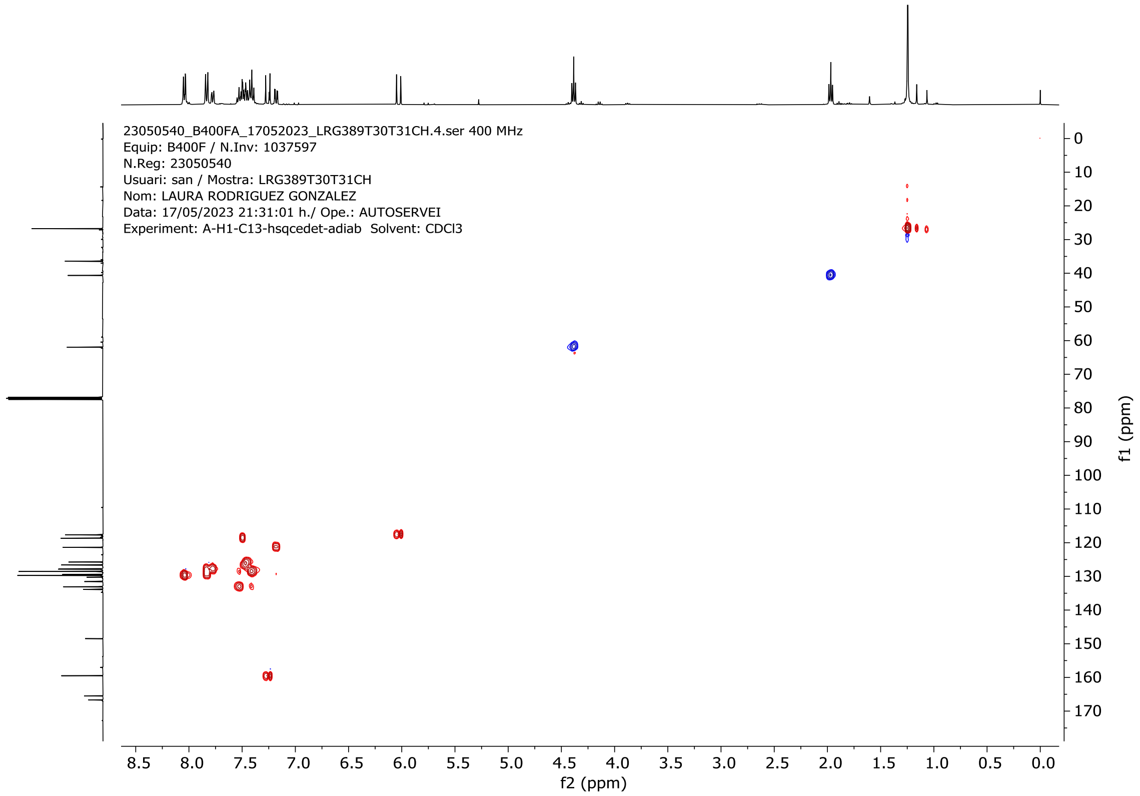

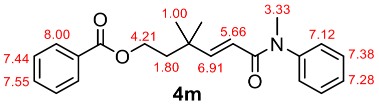

4m

23060349_B400FA_15062023_LRG401COLT41T42.1.fid 1H 400 MHz
Equip: B400F / N.Inv: 1037597
N.Reg: 23060349
Usuari: san / Mostra: LRG401COLT41T42
Nom: LAURA RODRIGUEZ GONZALEZ
Data: 15/06/2023 14:17:06 h./ Ope.: AUTOSERVEI
Experiment: A-H1-zg30 Solvent: CDCl3

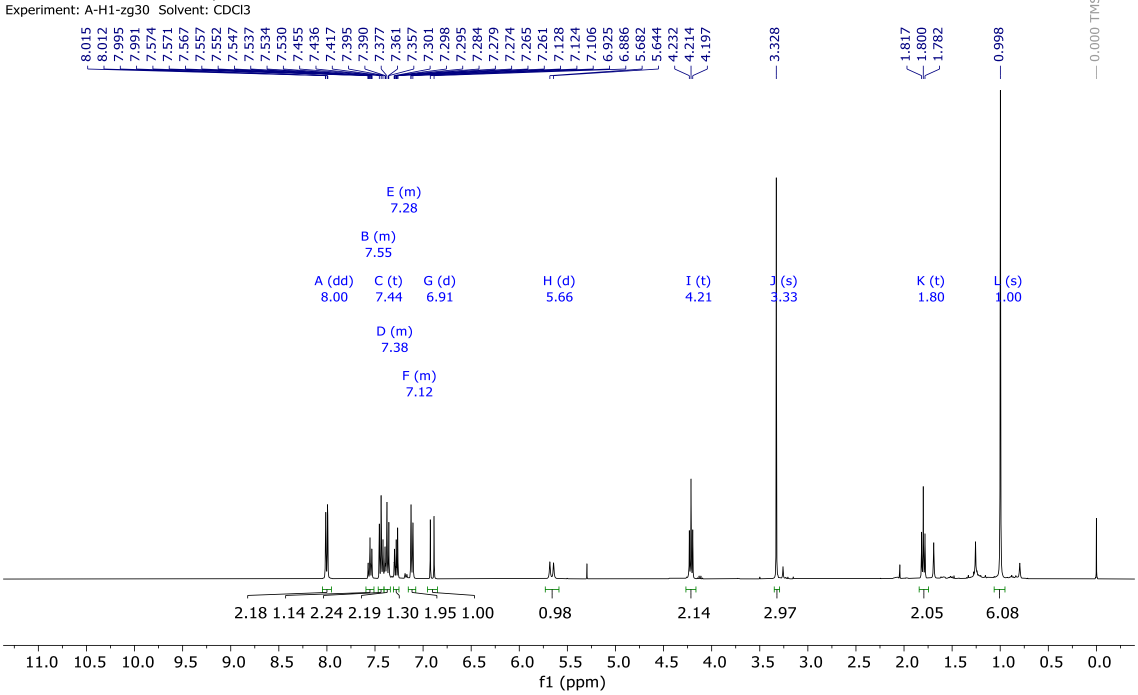

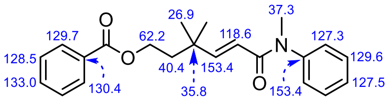

4m

23060352_B400FA_16062023_LRG401T41T42CH.2.fid 13C{1H} 101 MHz
Equip: B400F / N.Inv: 1037597
N.Reg: 23060352
Usuari: san / Mostra: LRG401T41T42CH
Nom: LAURA RODRIGUEZ GONZALEZ
Data: 16/06/2023 00:02:37 h./ Ope.: AUTOSERVEI
Experiment: A-C13-zgpg30 Solvent: CDCl3

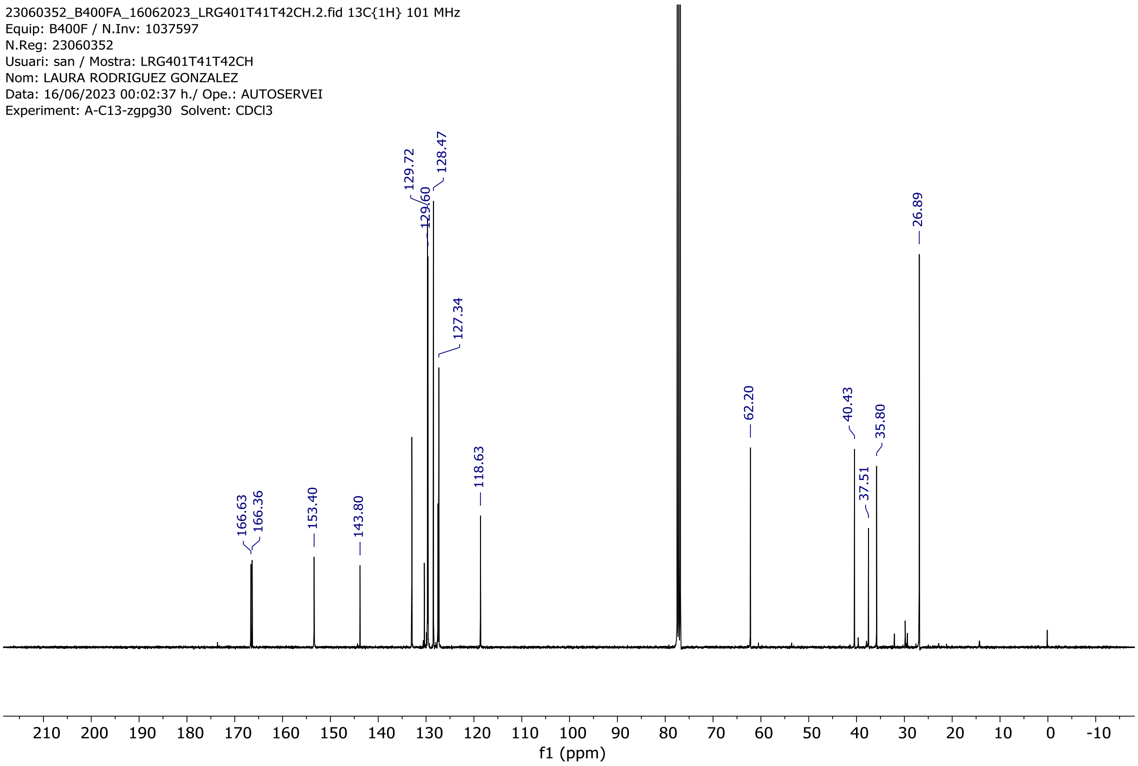

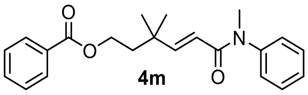

4m

2D-COSY

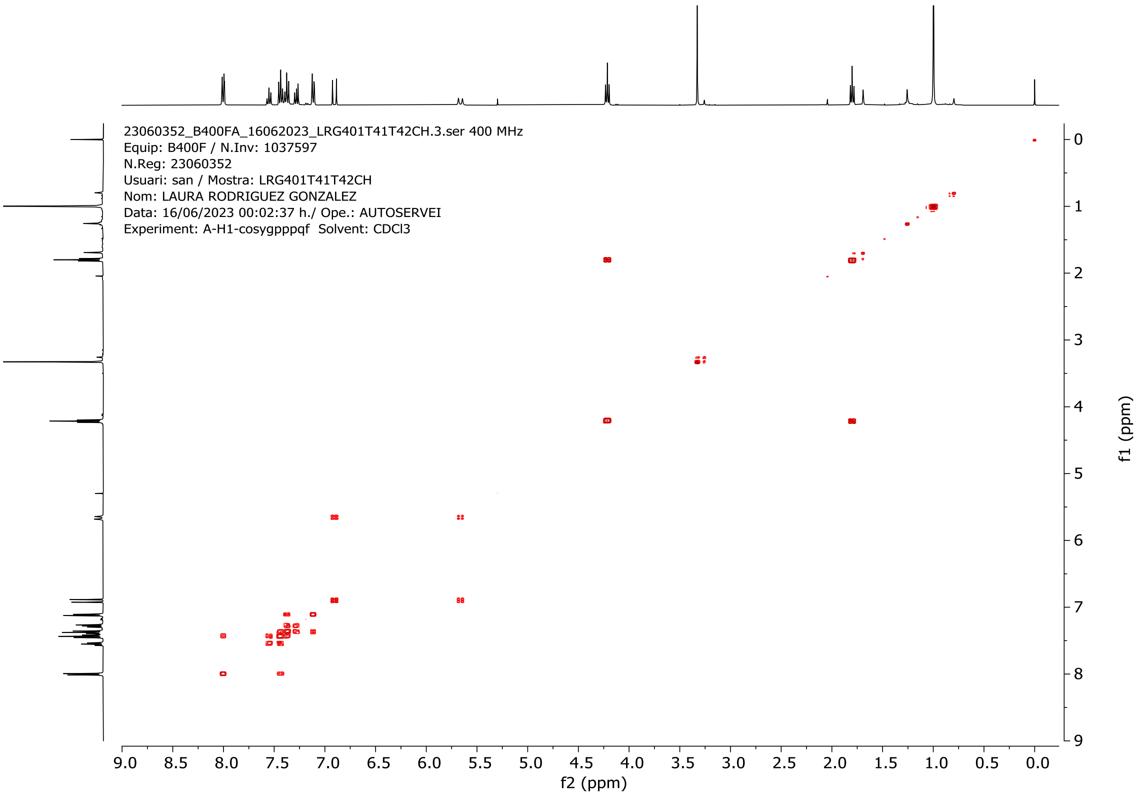

2D-HSQC

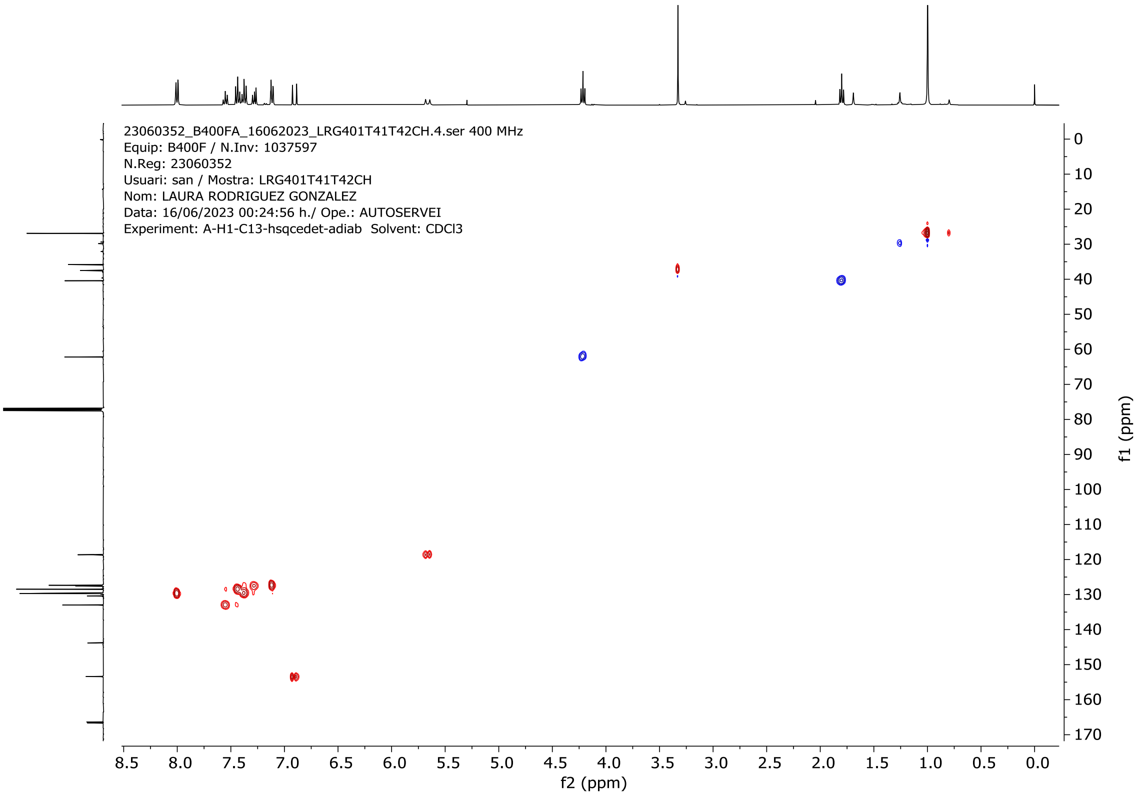

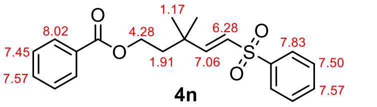

23070039_B400FA_04072023_LRG424COLT45T46.1.fid 1H 400 MHz
Equip: B400F / N.Inv: 1037597
N.Reg: 23070039
Usuari: san / Mostra: LRG424COLT45T46
Nom: LAURA RODRIGUEZ GONZALEZ
Data: 04/07/2023 10:51:45 h./ Ope.: AUTOSERVEI
Experiment: A-H1-zg30 Solvent: CDCl3

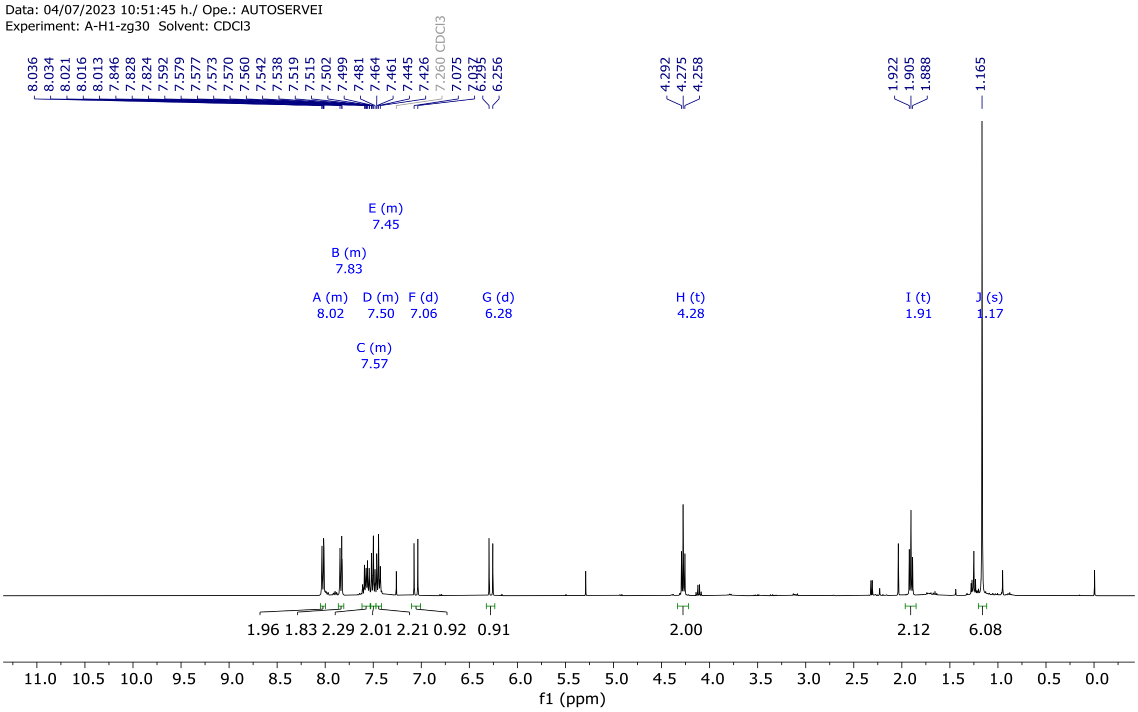

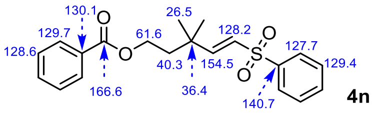

23070043_B400FA_04072023_LRG424T45T46CH.2.fid 13C{1H} 101 MHz
Equip: B400F / N.Inv: 1037597
N.Reg: 23070043
Usuari: san / Mostra: LRG424T45T46CH
Nom: LAURA RODRIGUEZ GONZALEZ
Data: 04/07/2023 21:05:45 h./ Ope.: AUTOSERVEI
Experiment: A-C13-zgpg30 Solvent: CDCl3

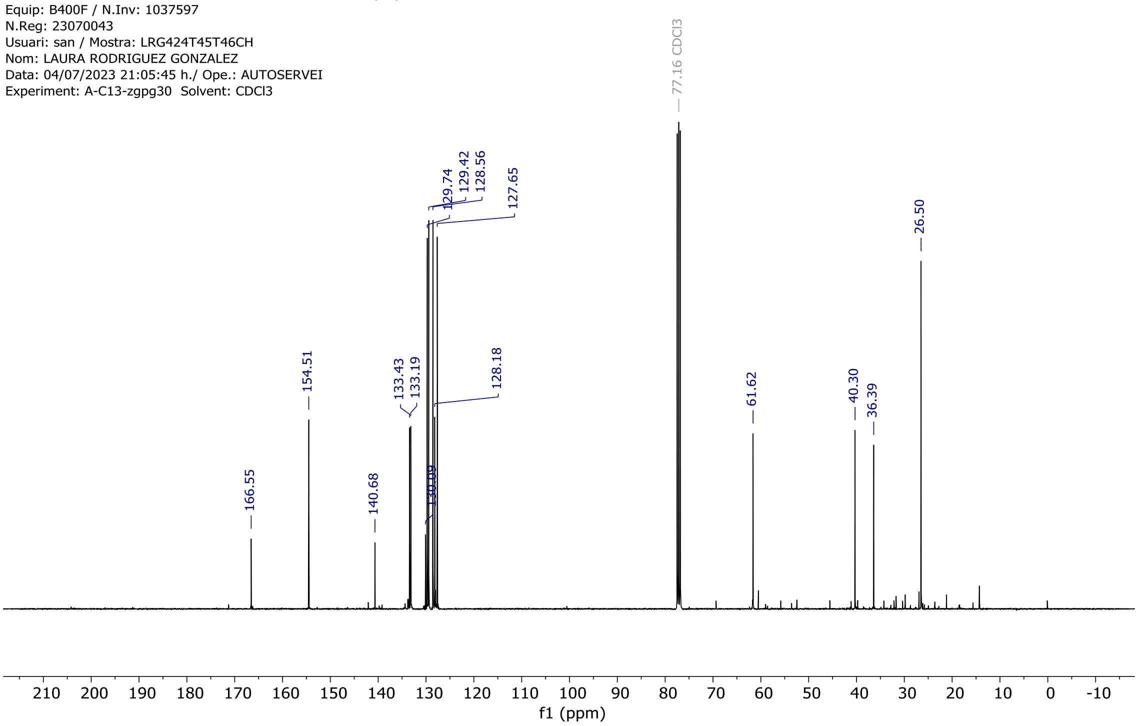

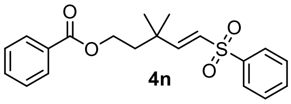

4n

2D-COSY

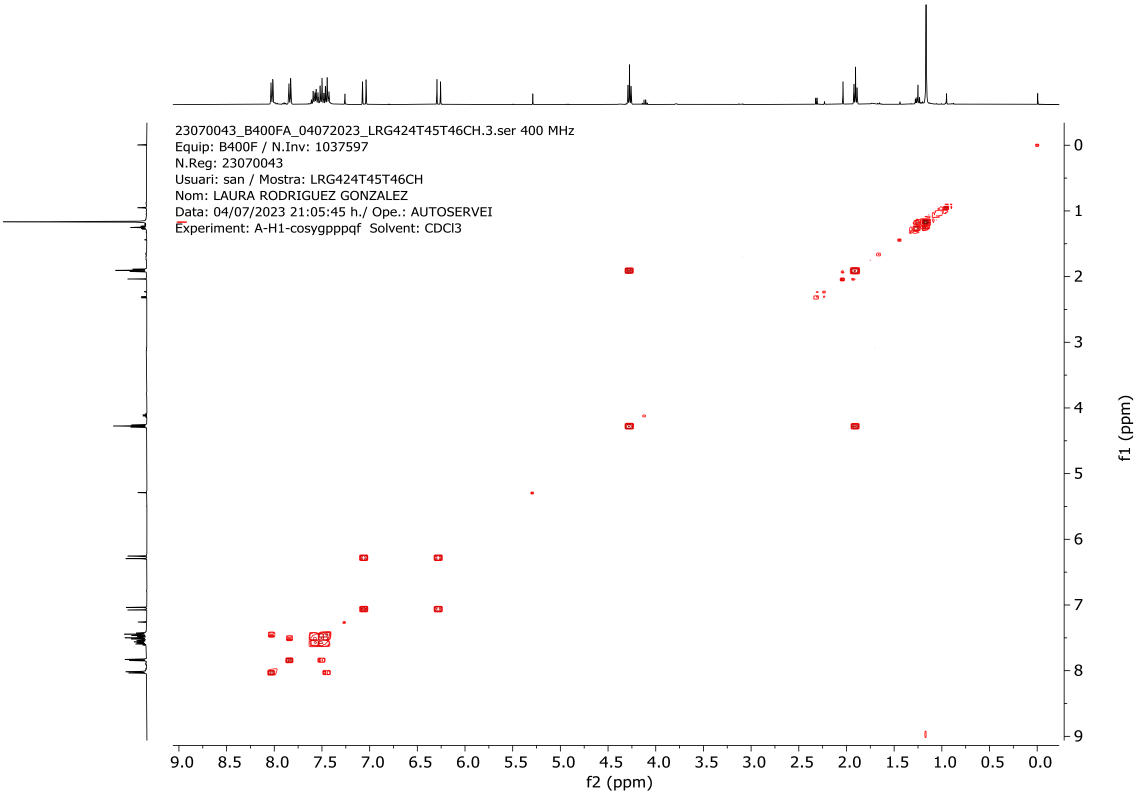

2D-HSQC

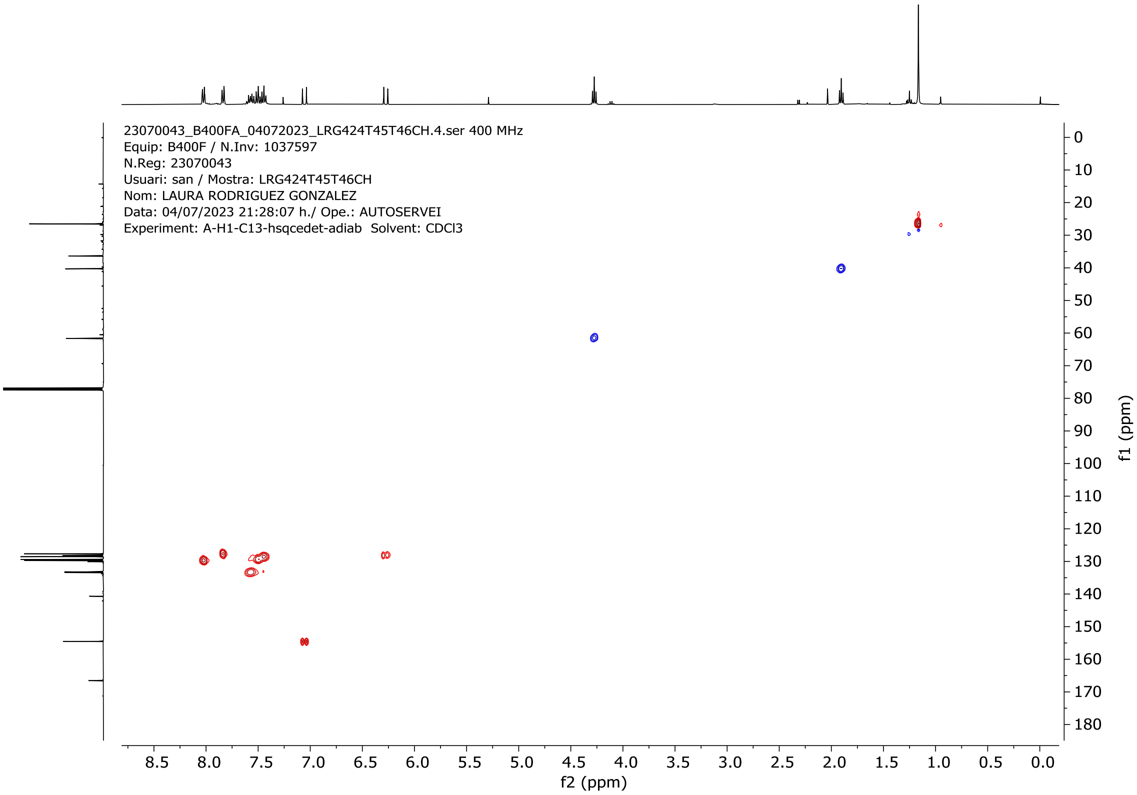

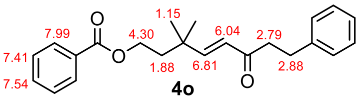

san-3874-2023.1.fid 1H 400 MHz
Equip: B400Q / N.Inv: 1035091
N.Reg: 3874/2023
Usuari: san / Mostra: LRG422T37T38CH
Nom: LAURA RODRIGUEZ GONZALEZ
Data: 28/06/2023 19:16:02 h./ Ope.: servei Unitat RMN
Experiment: A_1H-zg30 Solvent: CDCl3 Operator: VICTORIA MUÑOZ-TORRERO

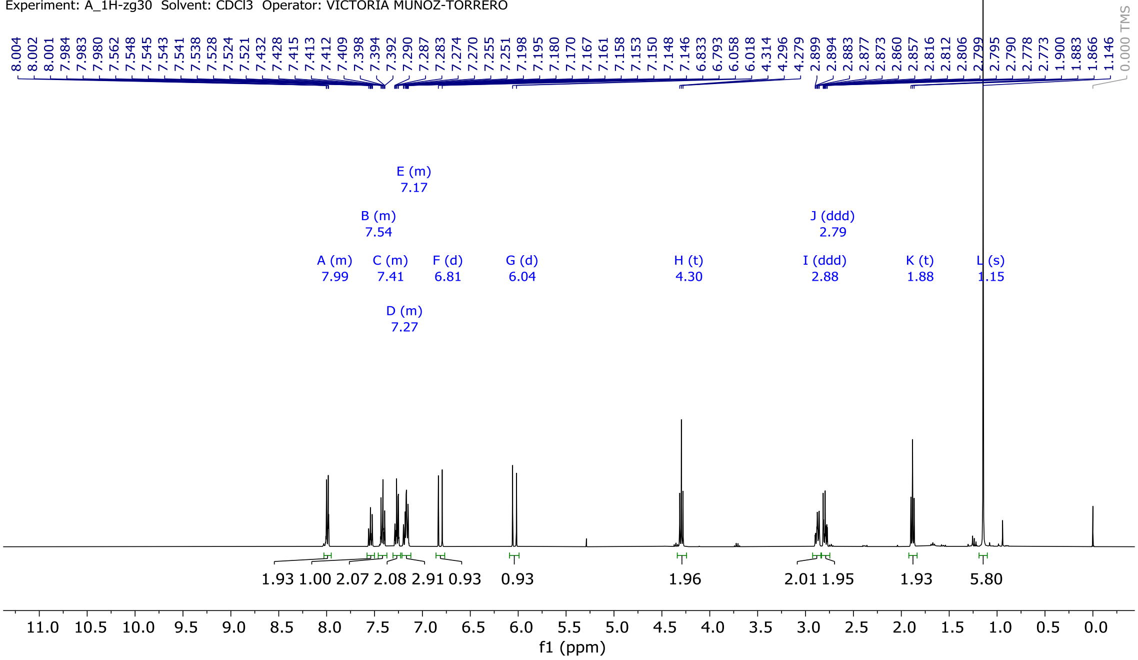

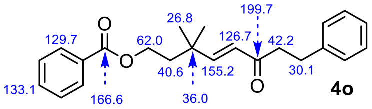

san-3874-2023.4.fid 13C{1H} 101 MHz
Equip: B400Q / N.Inv: 1035091
N.Reg: 3874/2023
Usuari: san / Mostra: LRG422T37T38CH
Nom: LAURA RODRIGUEZ GONZALEZ
Data: 28/06/2023 20:21:18 h./ Ope.: servei Unitat RMN
Experiment: A_13C-zgpg30 Solvent: CDCl3 Operator: VICTORIA MUÑOZ-TORRERO

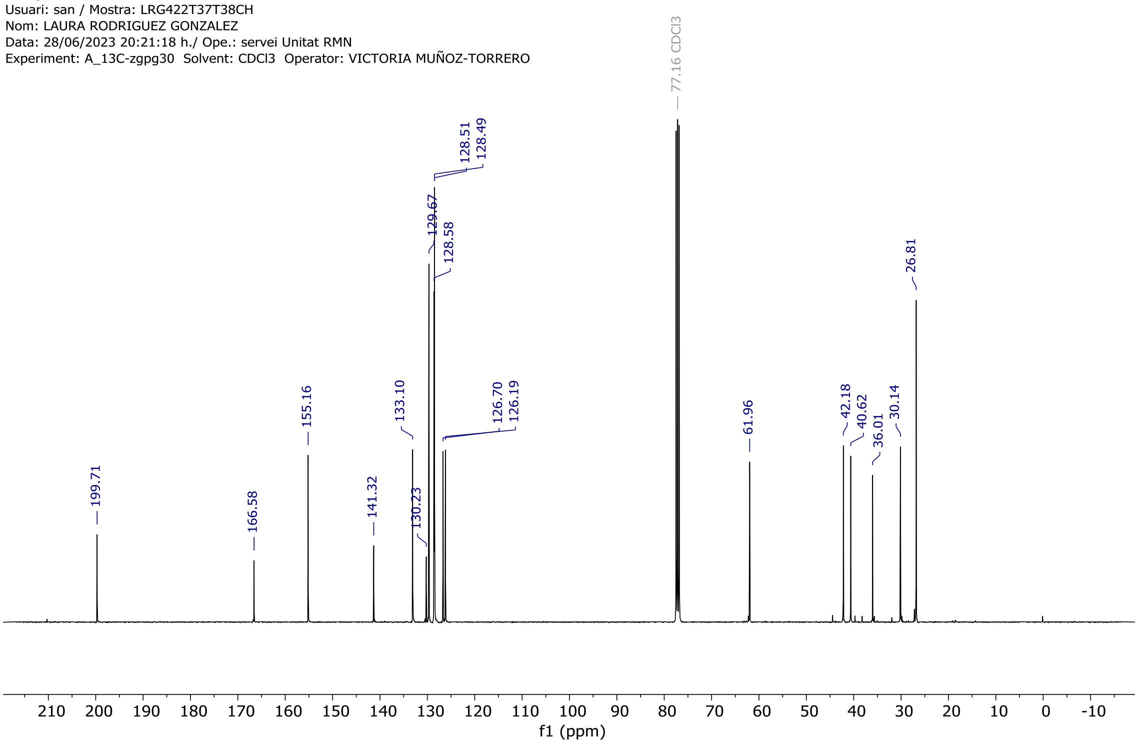

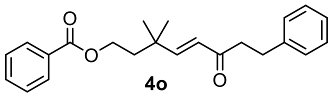

4o

2D-COSY

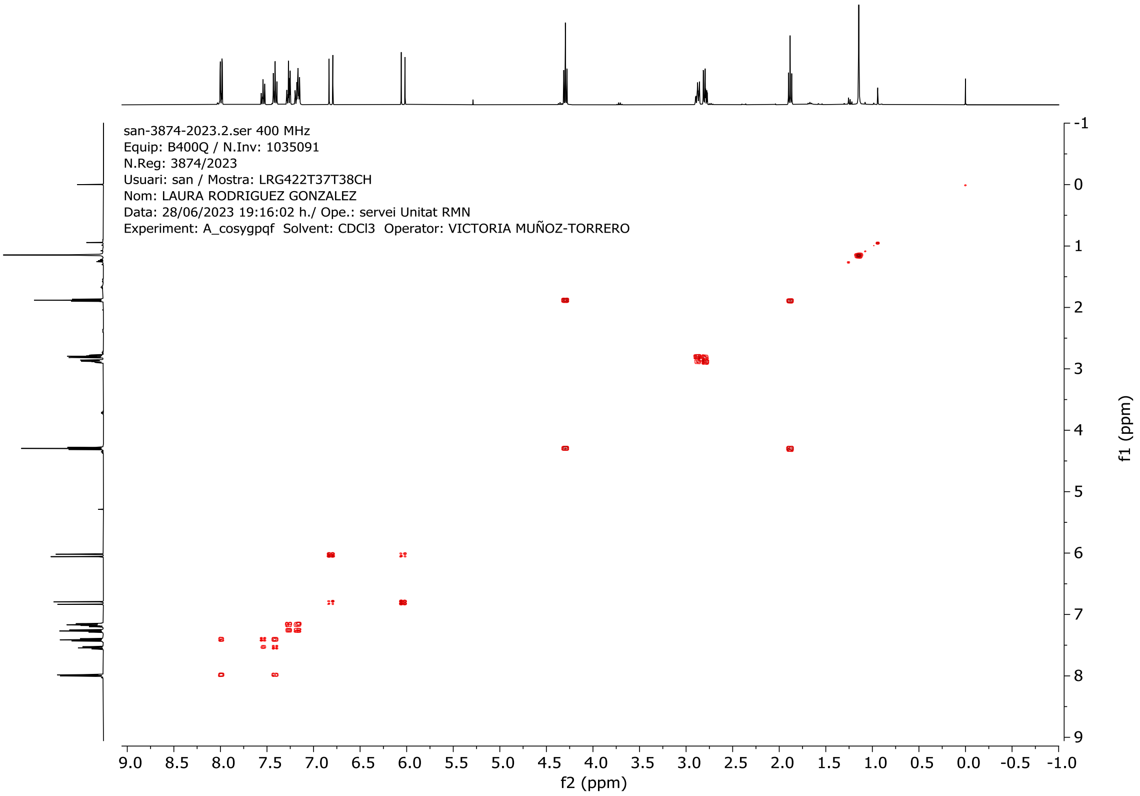

2D-HSQC

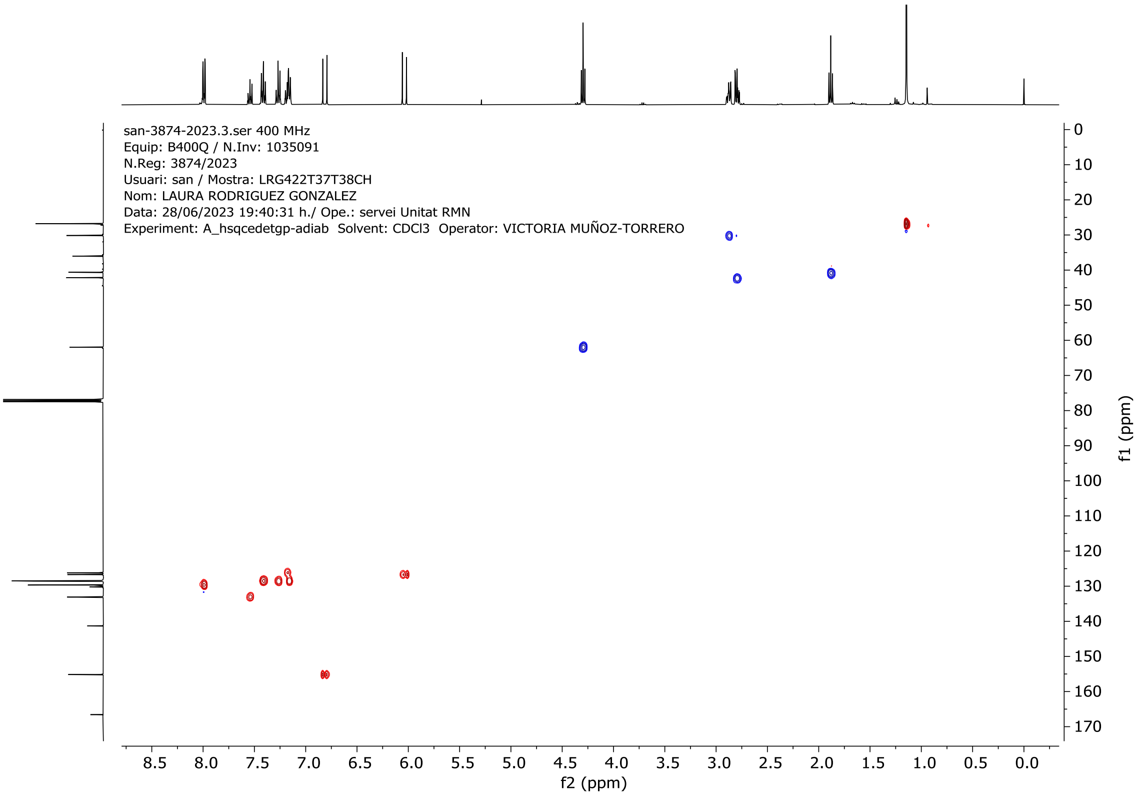

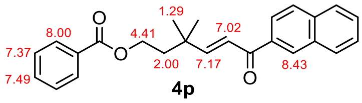

23060599_B400FA_23062023_LRG417COLT40.1.fid 1H 400 MHz
Equip: B400F / N.Inv: 1037597
N.Reg: 23060599
Usuari: san / Mostra: LRG417COLT40
Nom: LAURA RODRIGUEZ GONZALEZ
Data: 23/06/2023 11:00:16 h./ Ope.: AUTOSERVEI
Experiment: A-H1-zg30 Solvent: CDCl3

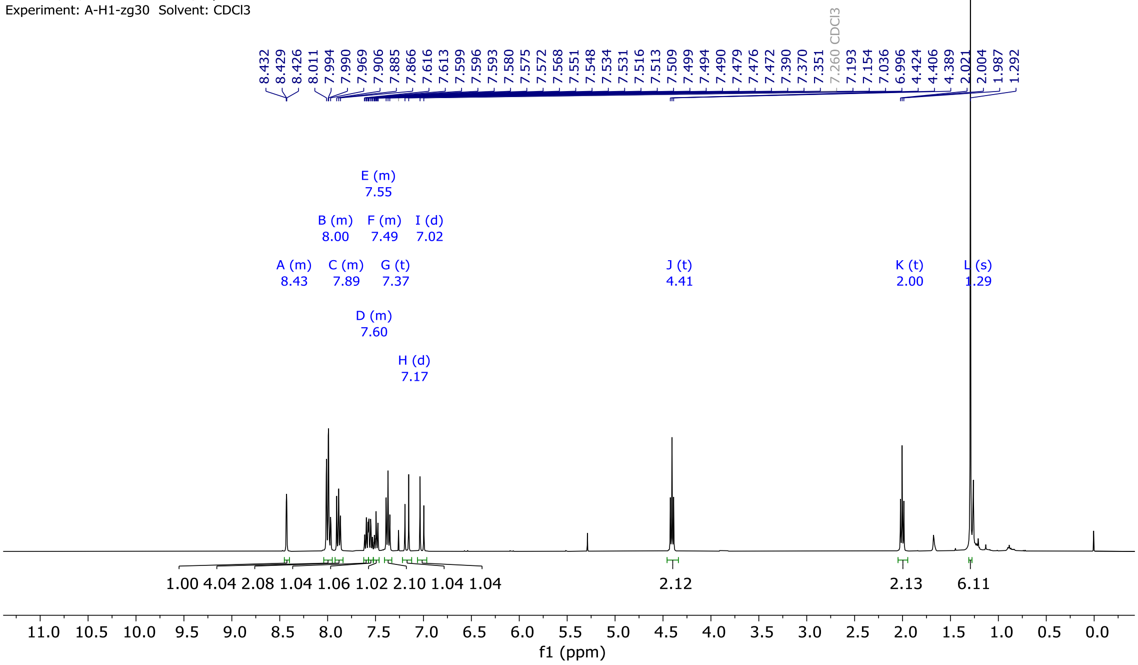

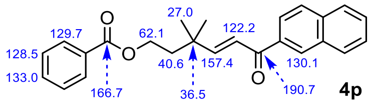

23060601_B400FA_25062023_LRG417T40CH.2.fid 13C{1H} 101 MHz
Equip: B400F / N.Inv: 1037597
N.Reg: 23060601
Usuari: san / Mostra: LRG417T40CH
Nom: LAURA RODRIGUEZ GONZALEZ
Data: 25/06/2023 15:33:26 h./ Ope.: AUTOSERVEI
Experiment: A-C13-zgpg30 Solvent: CDCl3

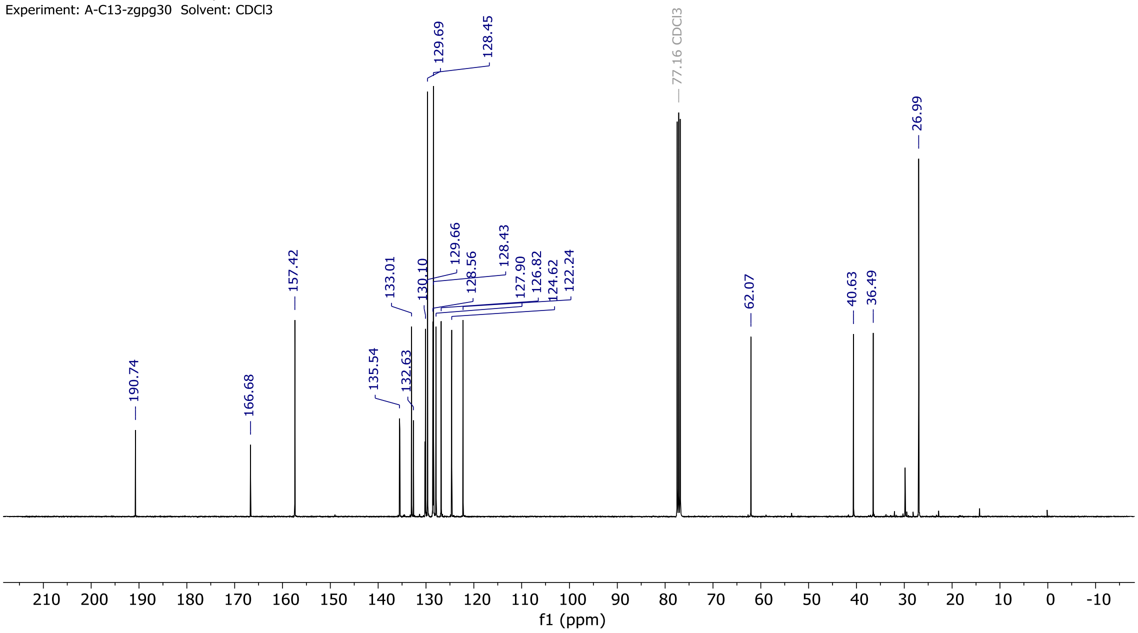

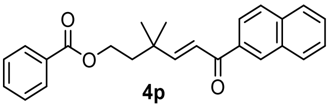

4p

2D-COSY

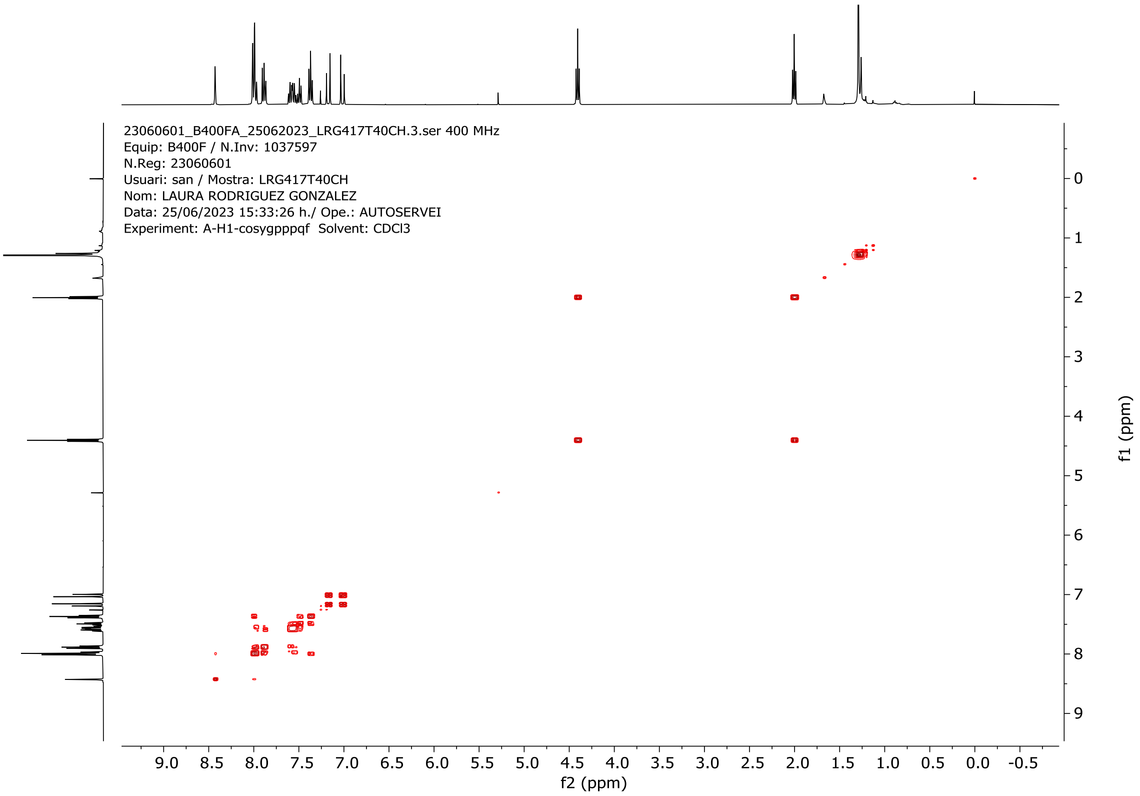

2D-HSQC

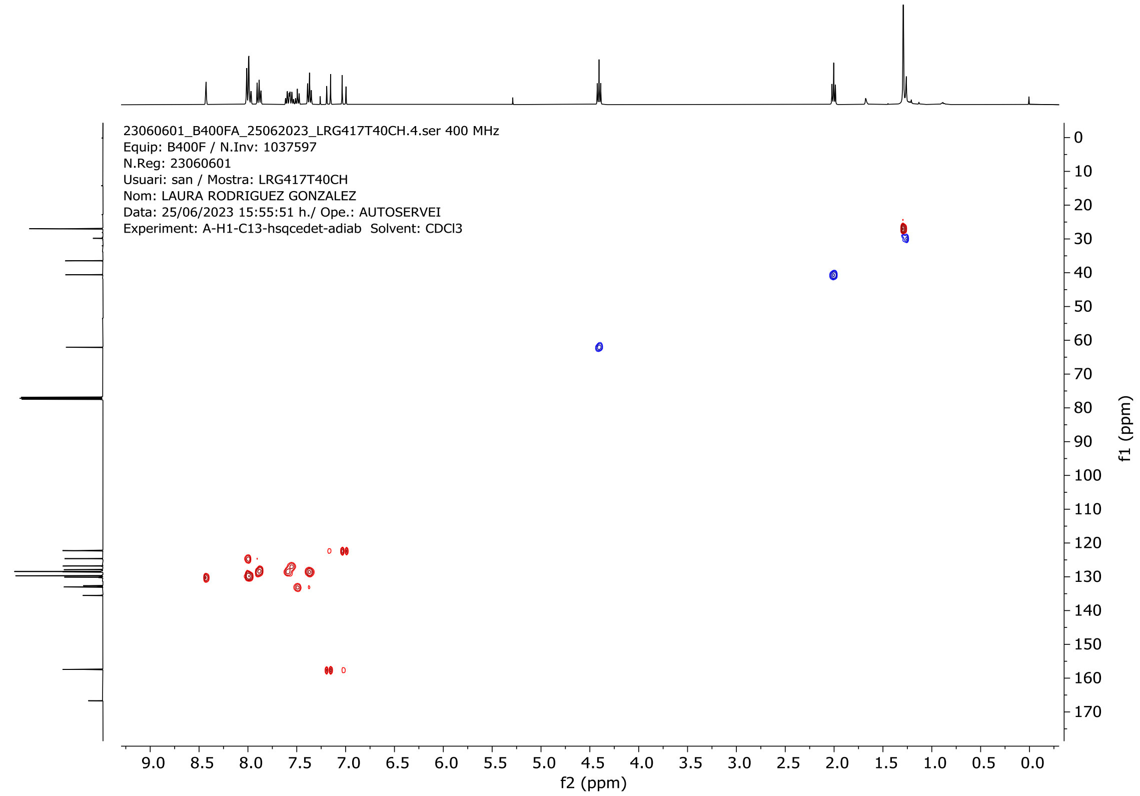

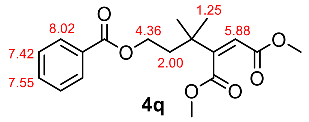

23090277_B400FA_15092023_LRG455COLT30.1.fid 1H 400 MHz
Equip: B400F / N.Inv: 1037597
N.Reg: 23090277
Usuari: san / Mostra: LRG455COLT30
Nom: LAURA RODRIGUEZ GONZALEZ
Data: 15/09/2023 15:14:11 h./ Ope.: AUTOSERVEI
Experiment: A-H1-zg30 Solvent: CDCl3

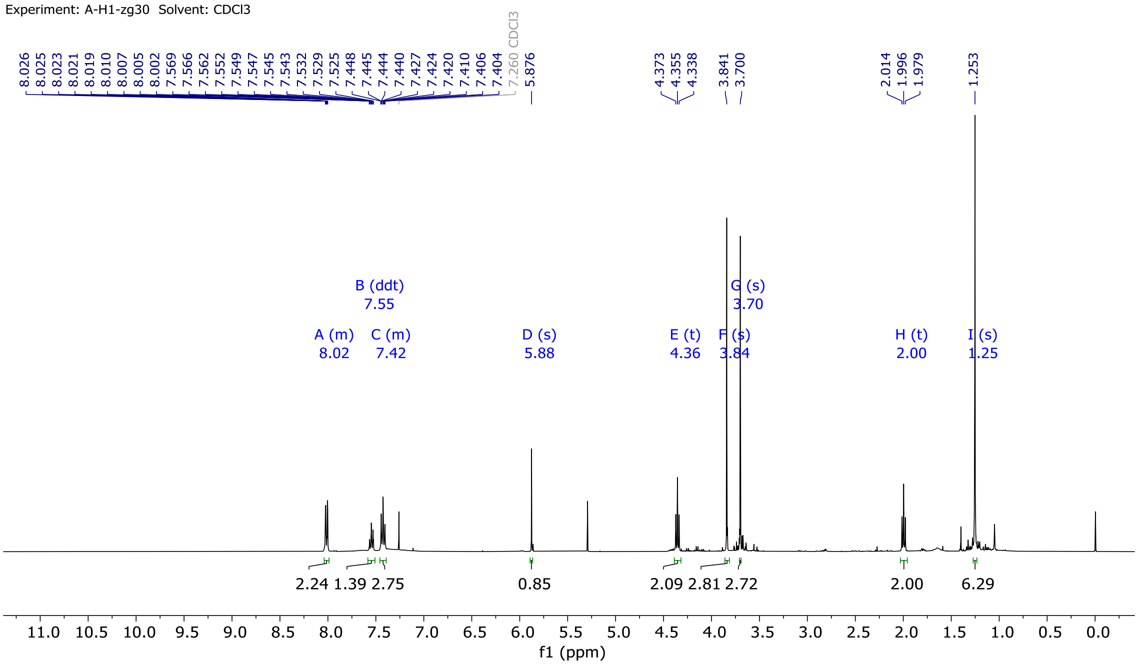

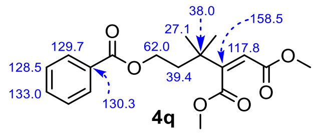

23090280_B400FA_17092023_LRG455COLT30CH.2.fid 13C{1H} 101 MHz
Equip: B400F / N.Inv: 1037597
N.Reg: 23090280
Usuari: san / Mostra: LRG455COLT30CH
Nom: LAURA RODRIGUEZ GONZALEZ
Data: 17/09/2023 13:11:46 h./ Ope.: AUTOSERVEI
Experiment: A-C13-zgpg30 Solvent: CDCl3

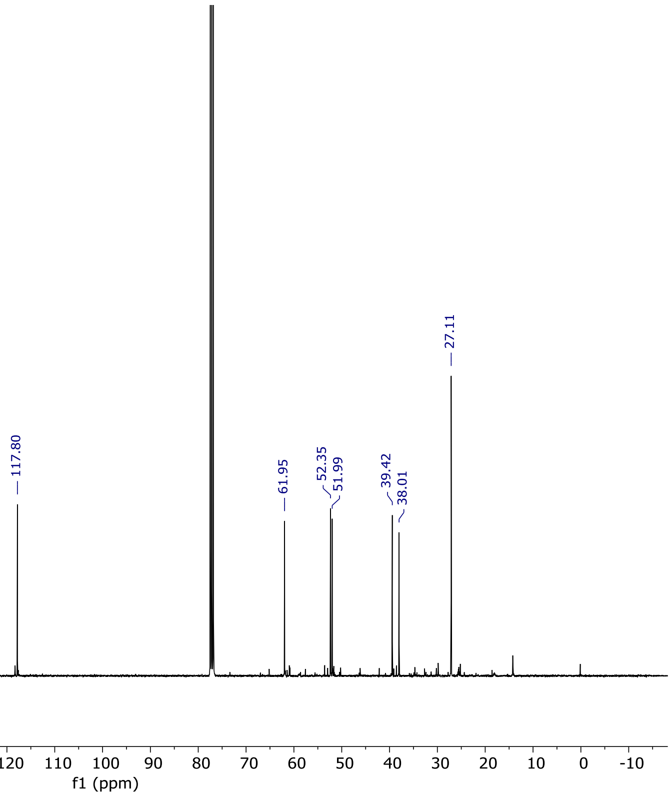

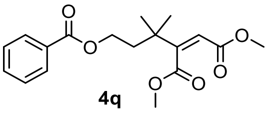

4q

2D-COSY

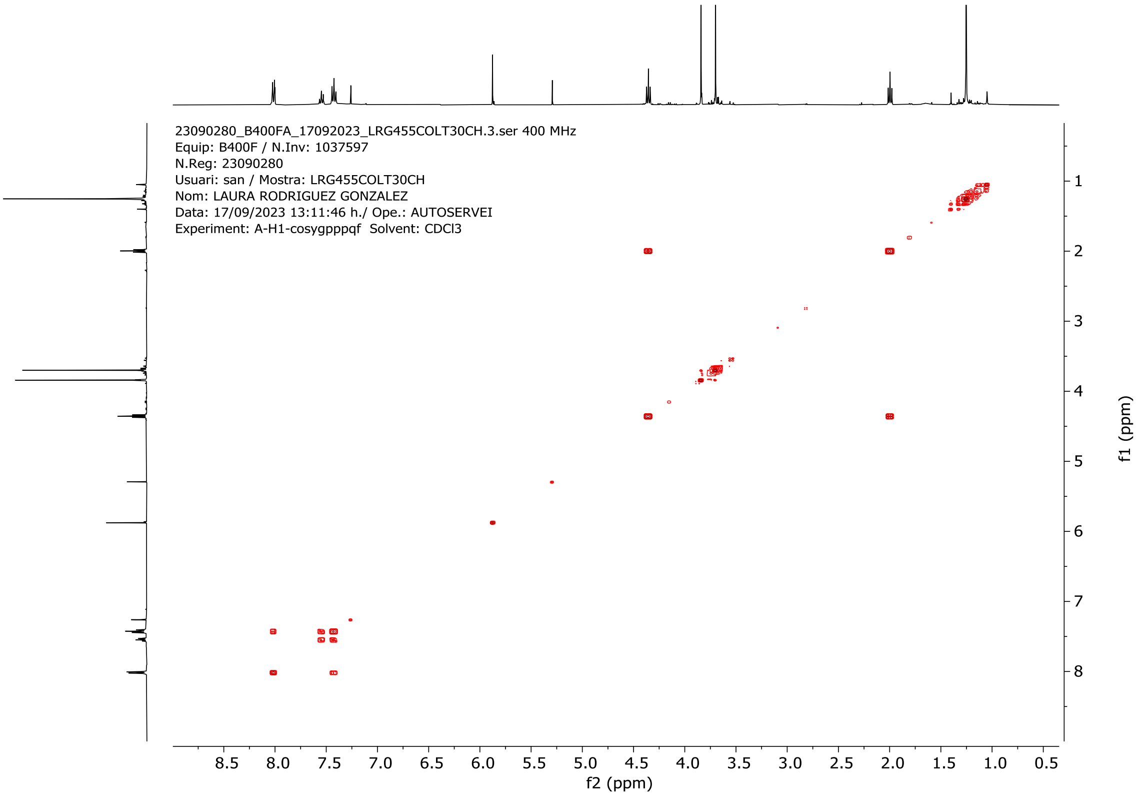

2D-HSQC

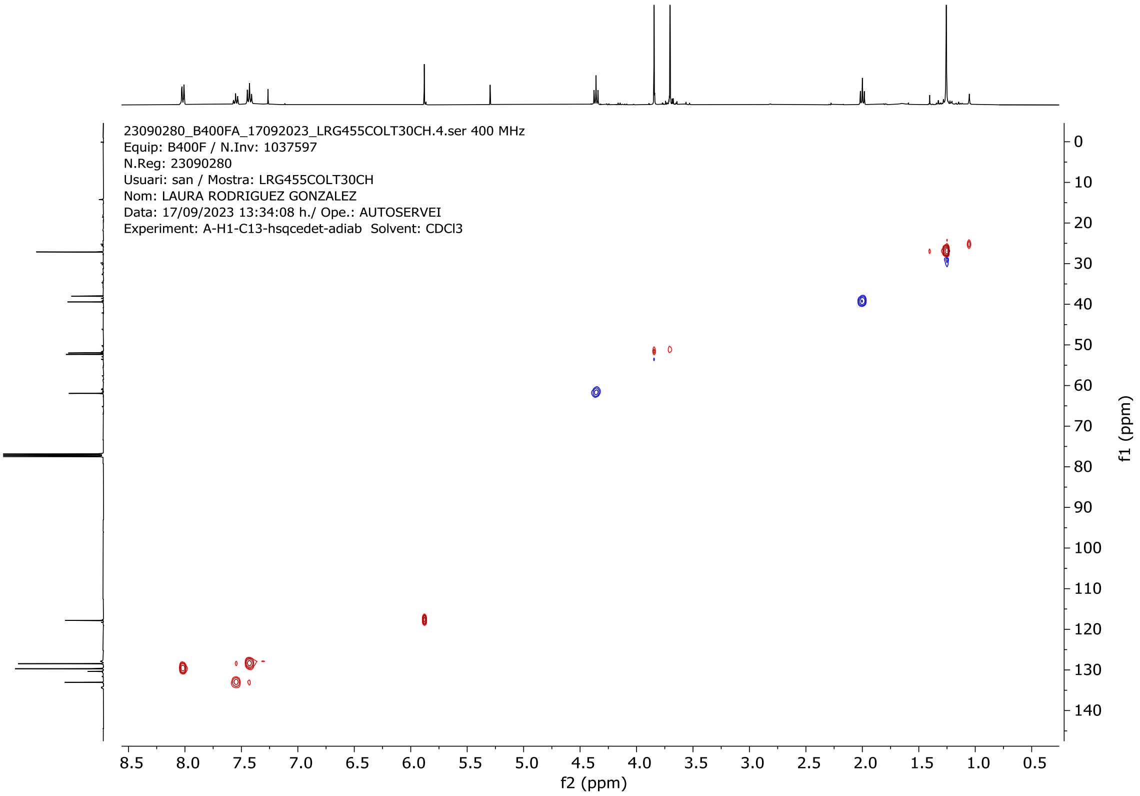

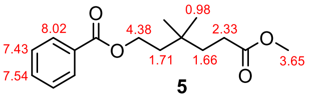

5

23030101_B400FA_02032023_LRG338COL.1.fid 1H 400 MHz
Equip: B400F / N.Inv: 1037597
N.Reg: 23030101
Usuari: san / Mostra: LRG338COL
Nom: LAURA RODRIGUEZ GONZALEZ
Data: 02/03/2023 12:15:12 h./ Ope.: AUTOSERVEI
Experiment: A-H1-zg30 Solvent: CDCl3

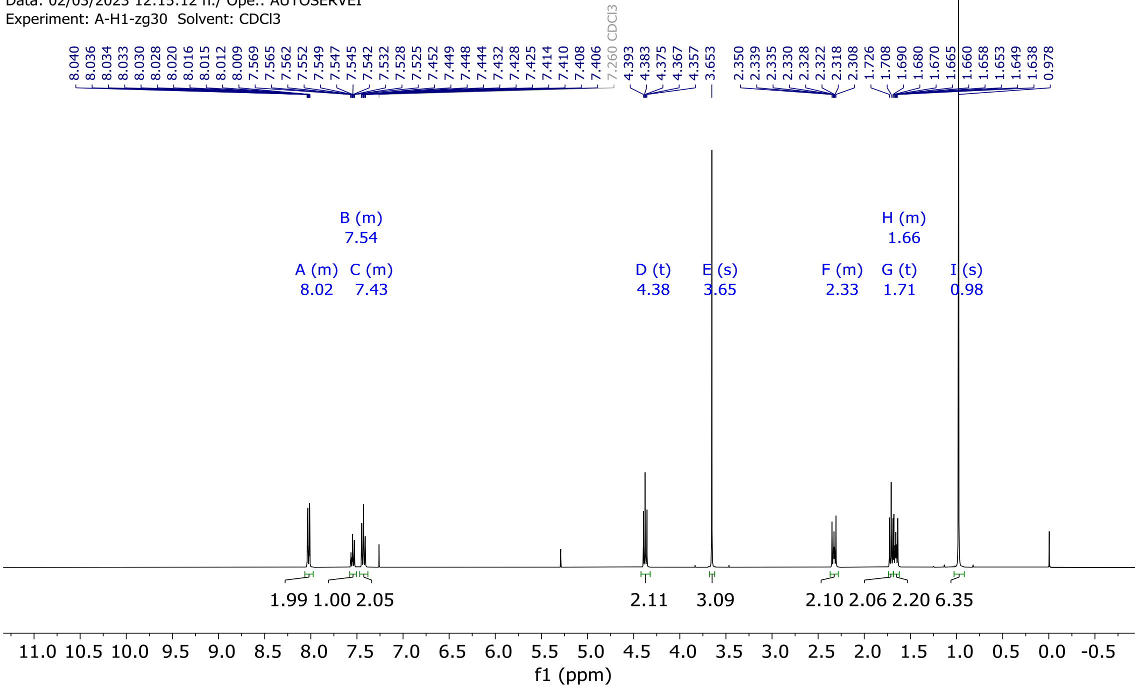

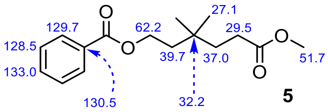

5

LRG338COL13C.2.fid 13C{1H} 101 MHz
Equip: B400F / N.Inv: 1037597
N.Reg: 23030101
Usuari: san / Mostra: LRG338COL
Nom: LAURA RODRIGUEZ GONZALEZ
Data: 02/03/2023 22:34:07 h./ Ope.: AUTOSERVEI
Experiment: A-C13-zgpg30 Solvent: CDCl3

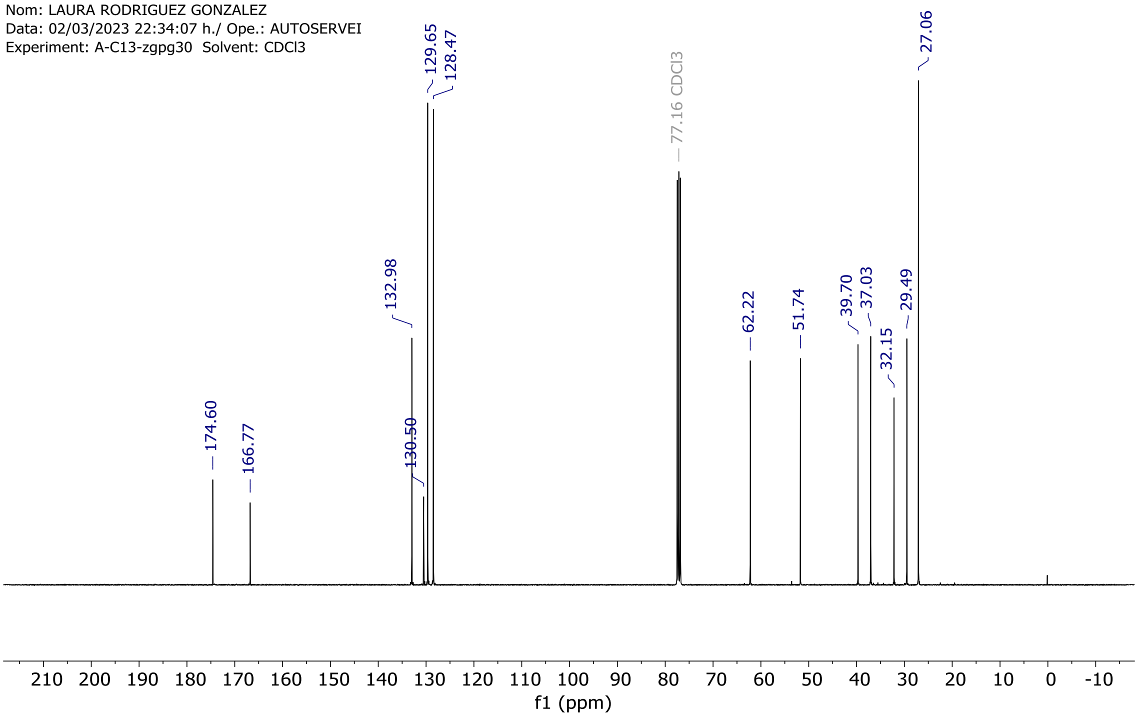

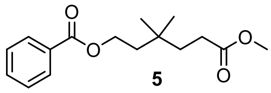

5

2D-COSY

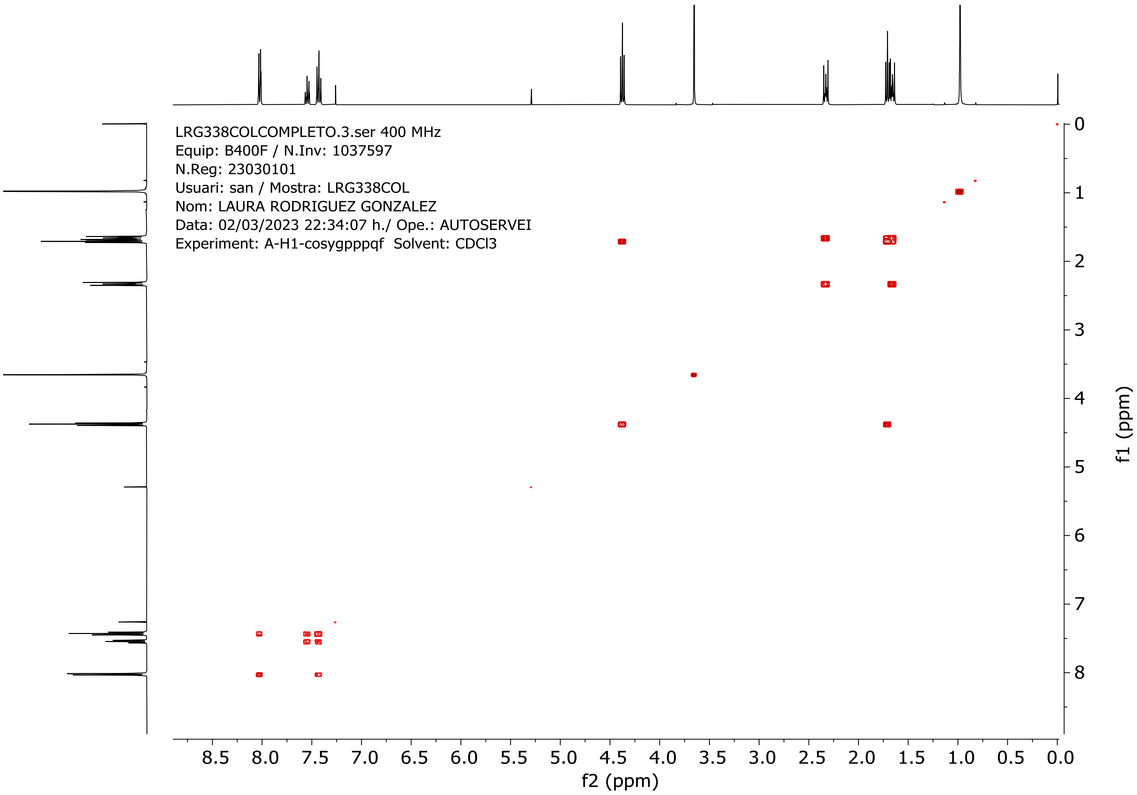

2D-HSQC

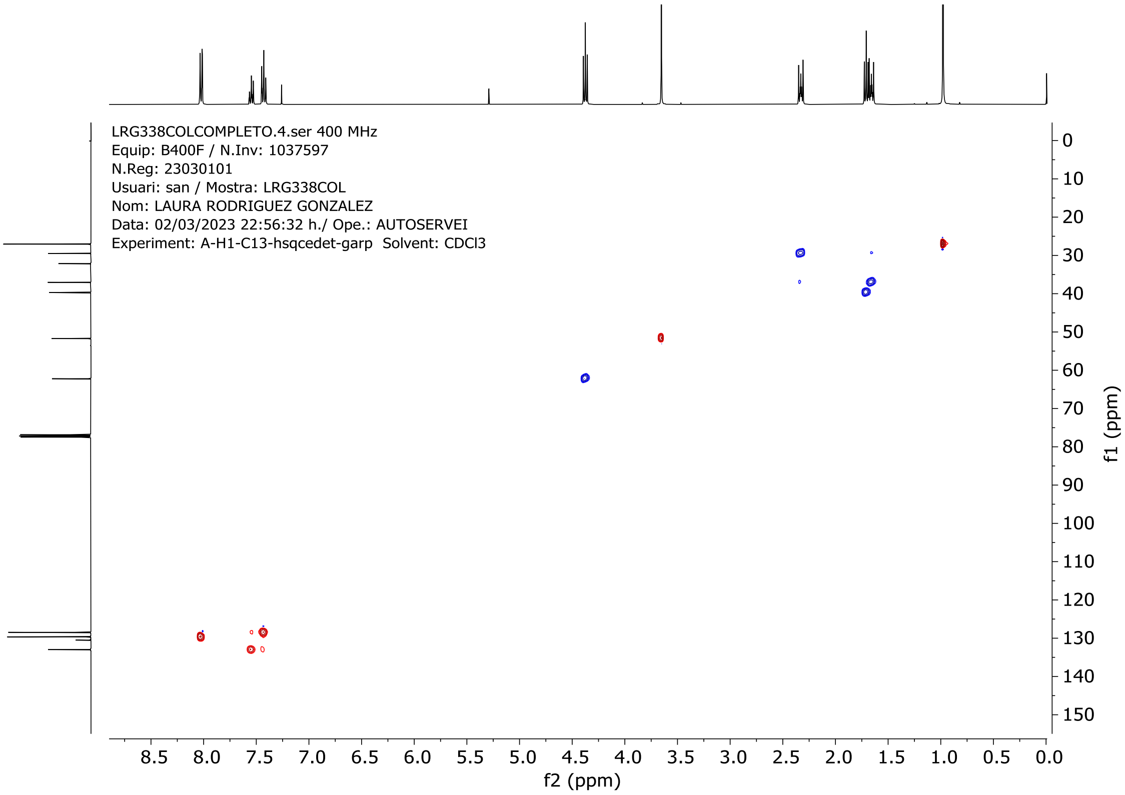

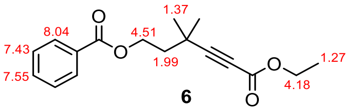

6

san-2803-2023.1.fid 1H 400 MHz
Equip: B400Q / N.Inv: 1035091
N.Reg: 2803/2023
Usuari: san / Mostra: LRG385COLT25T26
Nom: LAURA RODRIGUEZ GONZALEZ
Data: 10/05/2023 15:07:07 h./ Ope.: servei Unitat RMN
Experiment: A_1H-zg30 Solvent: CDCl3 Operator: Victoria Munoz Torrero

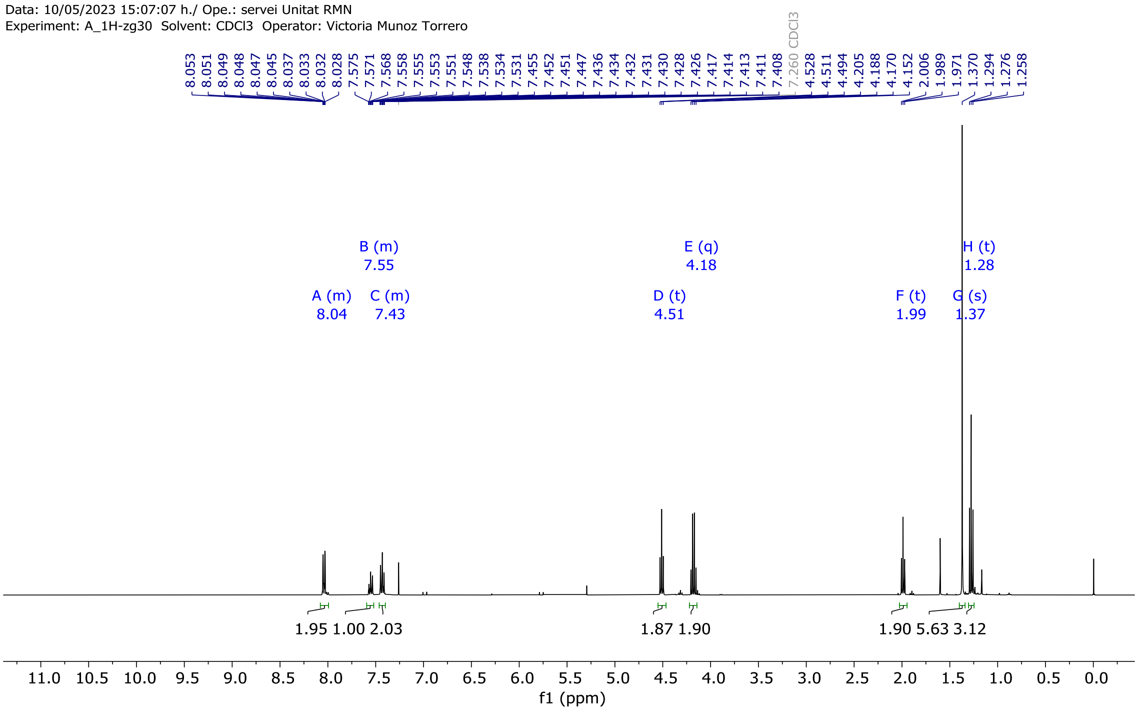

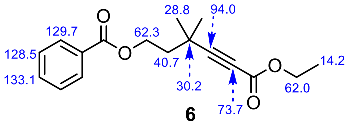

6

san-2848-2023-2.2.fid 13C{1H} 101 MHz
Equip: B400Q / N.Inv: 1035091
N.Reg: 2848/2023
Usuari: san / Mostra: LRG385CH
Nom: LAURA RODRIGUEZ GONZALEZ
Data: 11/05/2023 21:19:32 h./ Ope.: servei Unitat RMN
Experiment: A_13C-zgpg30 Solvent: CDCl3 Operator: Victor Meriel

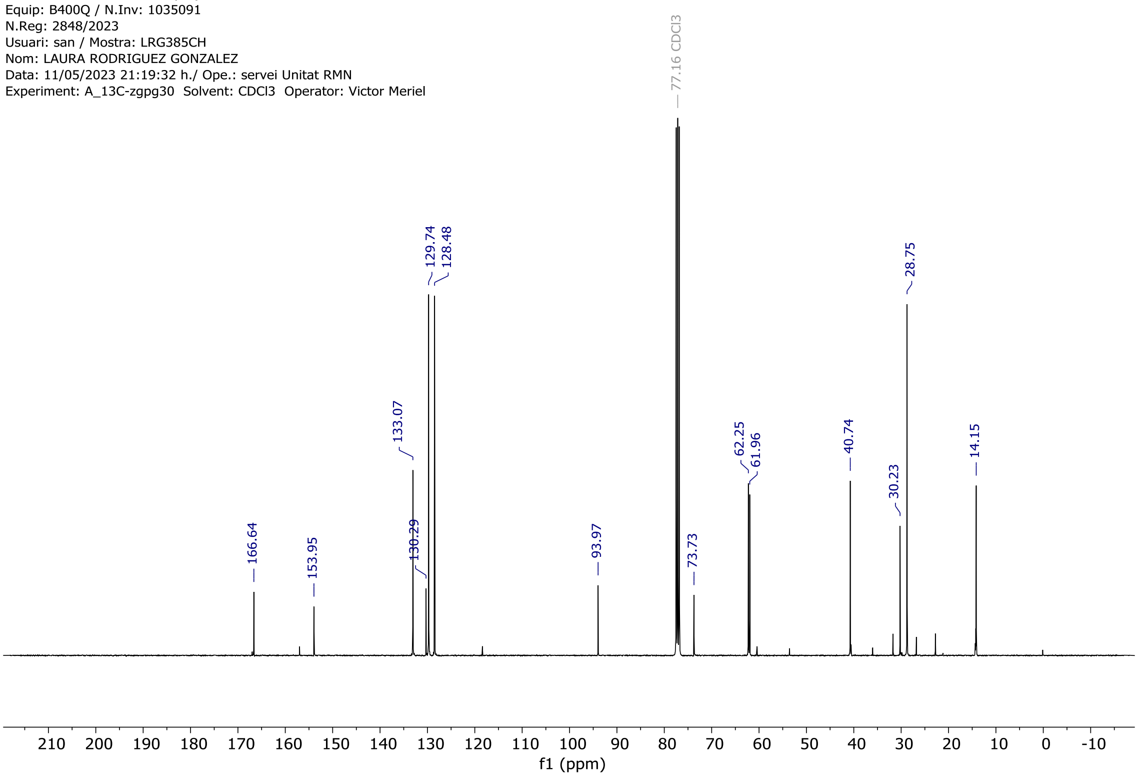

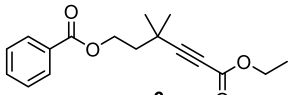

6

2D-COSY

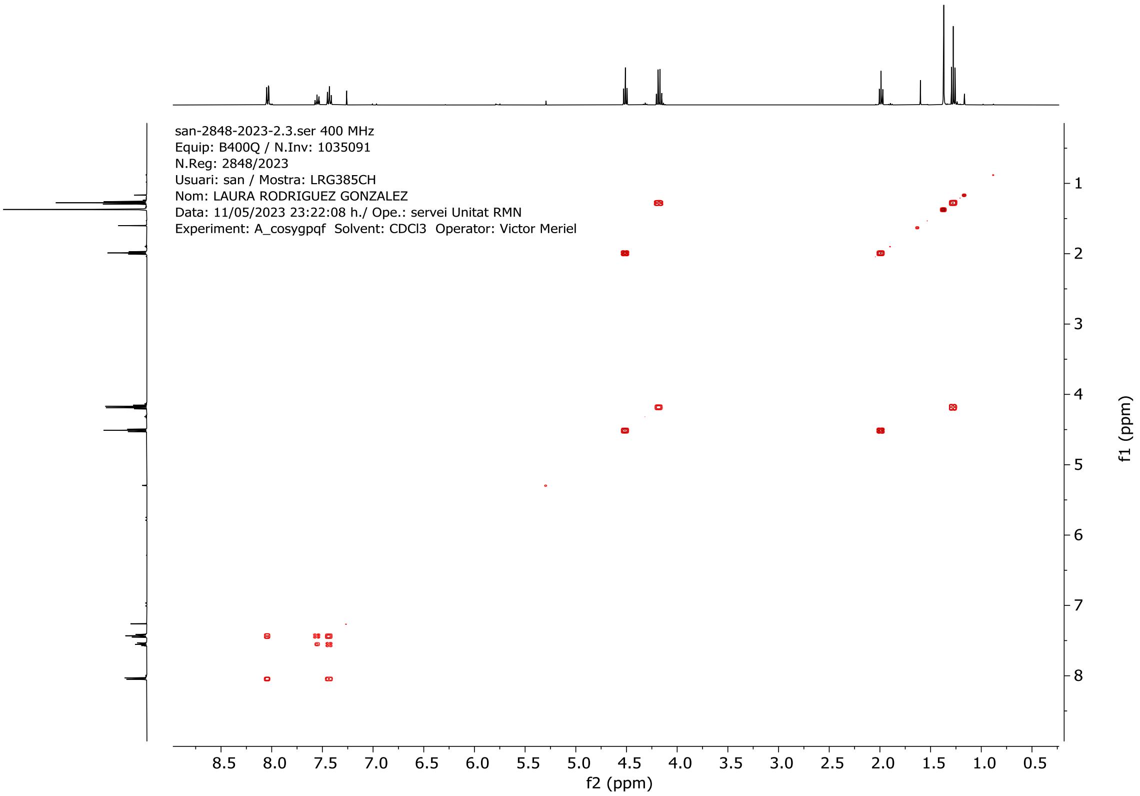

2D-HSQC

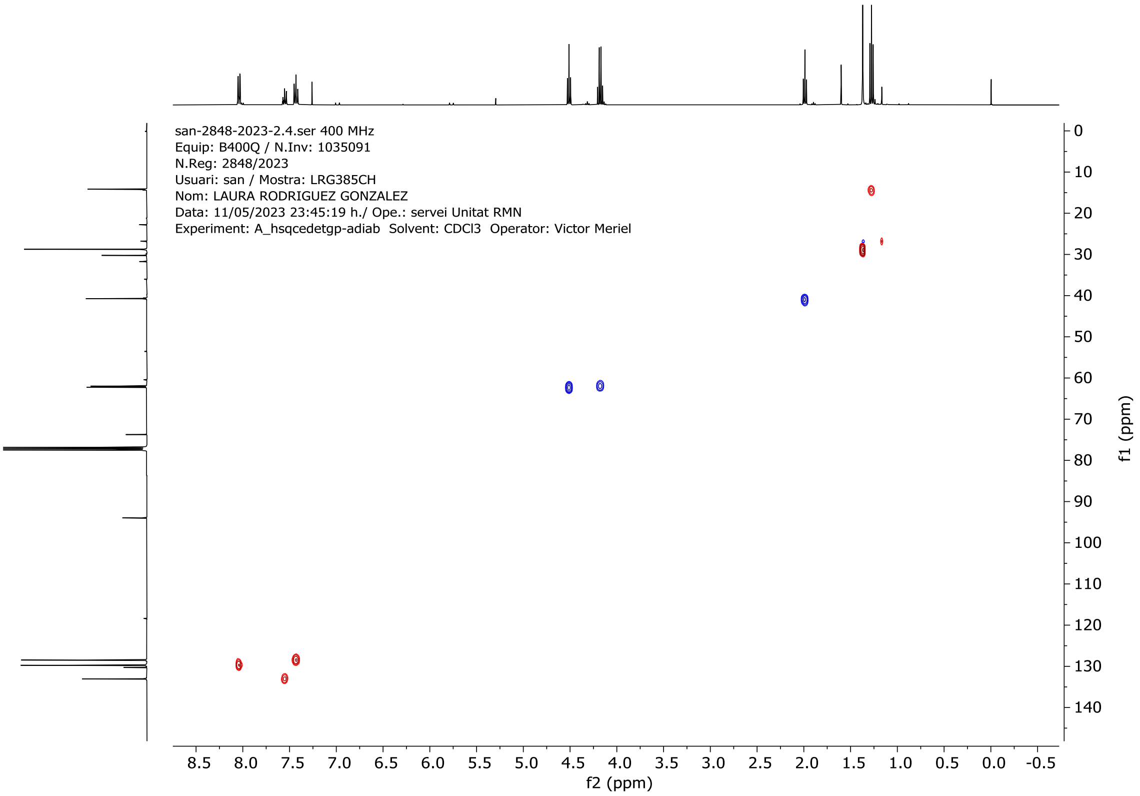

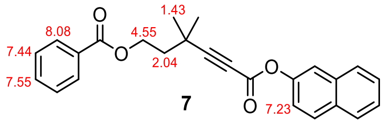

23050702_B400FA_23052023_LRG392COL1T32T33CH.1.fid 1H 400 MHz
Equip: B400F / N.Inv: 1037597
N.Reg: 23050702
Usuari: san / Mostra: LRG392COL1T32T33CH
Nom: LAURA RODRIGUEZ GONZALEZ
Data: 23/05/2023 12:50:20 h./ Ope.: AUTOSERVEI
Experiment: A-H1-zg30 Solvent: CDCl3

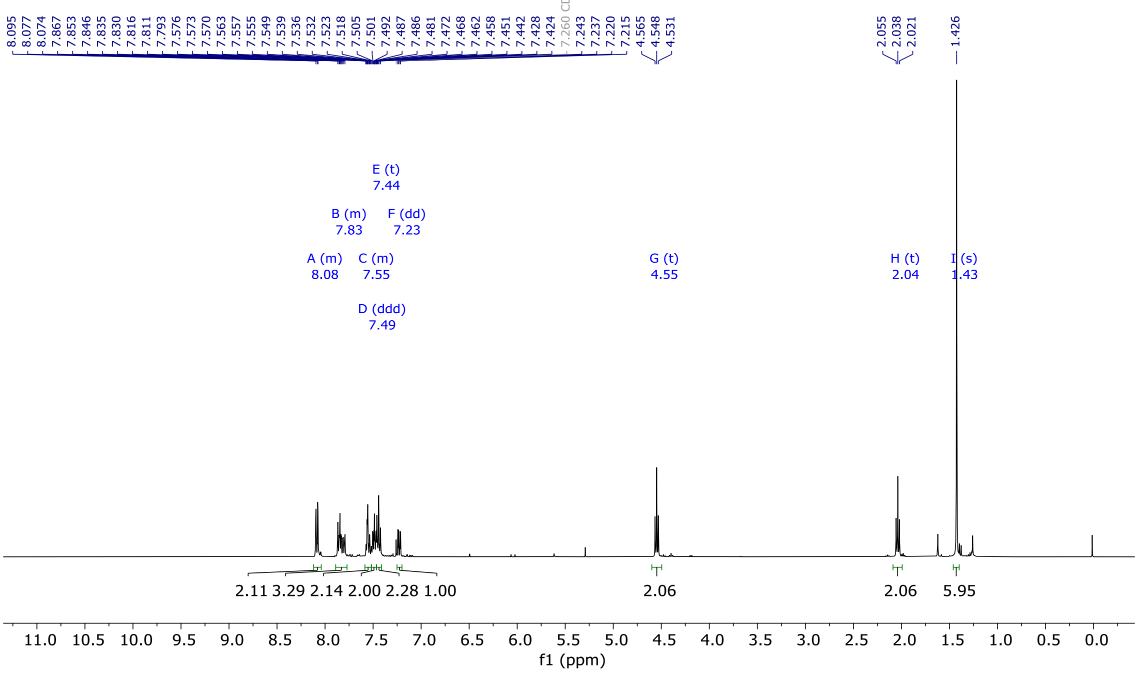

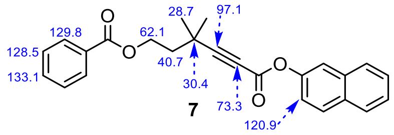

23050702_B400FA_23052023_LRG392COL1T32T33CH.2.fid 13C{1H} 101 MHz
Equip: B400F / N.Inv: 1037597
N.Reg: 23050702
Usuari: san / Mostra: LRG392COL1T32T33CH
Nom: LAURA RODRIGUEZ GONZALEZ
Data: 23/05/2023 21:06:32 h./ Ope.: AUTOSERVEI
Experiment: A-C13-zgpg30 Solvent: CDCl3

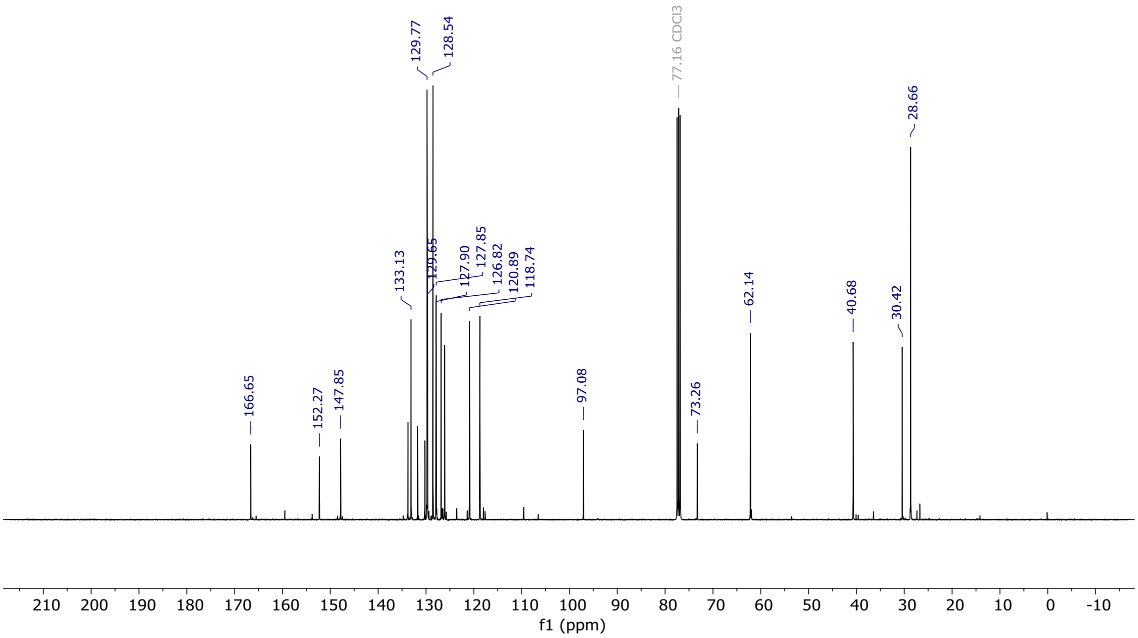

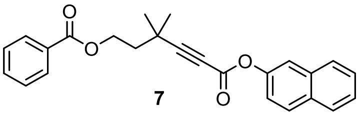

7

2D-COSY

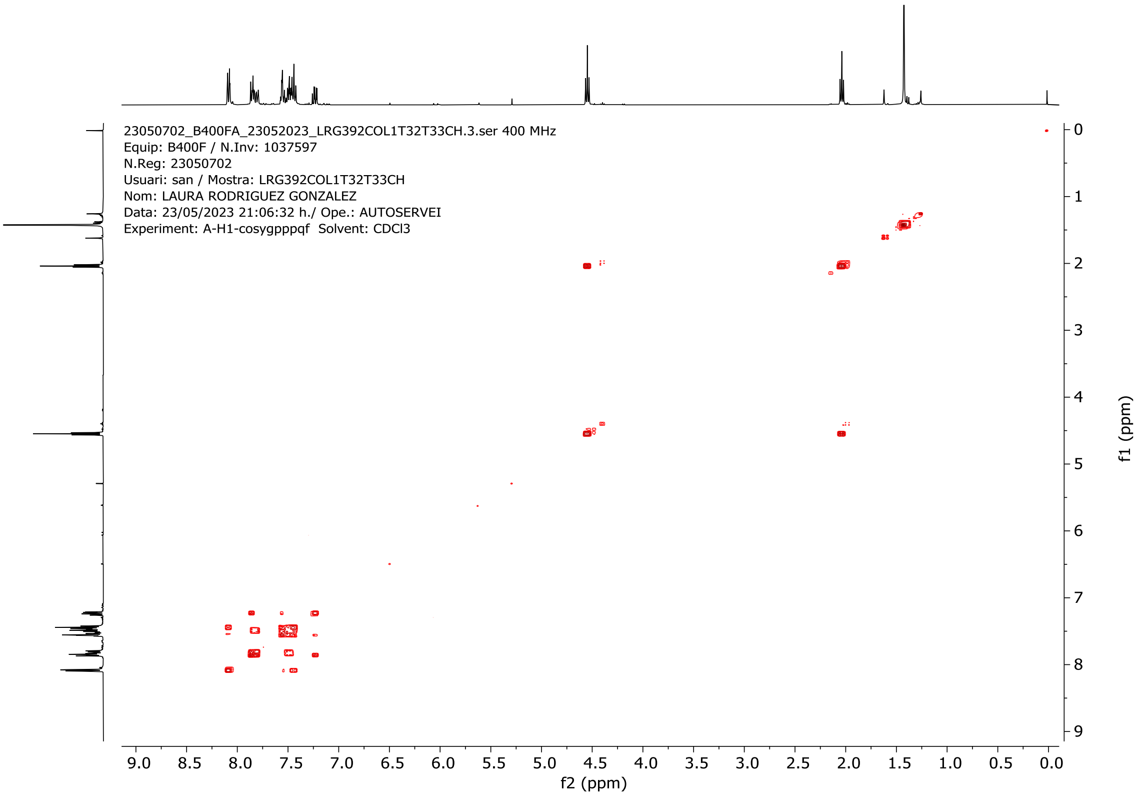

2D-HSQC

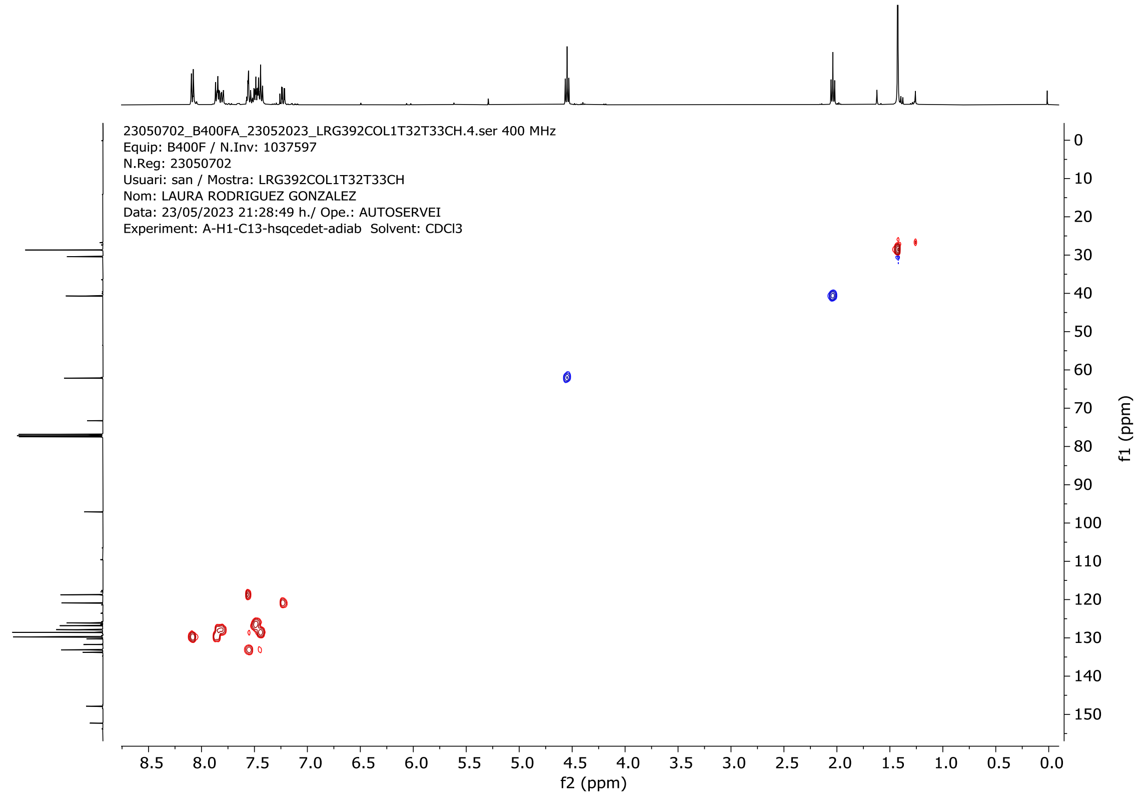

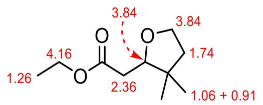

SI-A

23060491_B400FA_20062023_LRG411CH.1.fid 1H 400 MHz
Equip: B400F / N.Inv: 1037597
N.Reg: 23060491
Usuari: san / Mostra: LRG411CH
Nom: LAURA RODRIGUEZ GONZALEZ
Data: 20/06/2023 16:21:33 h./ Ope.: AUTOSERVEI
Experiment: A-H1-zg30 Solvent: CDCl3

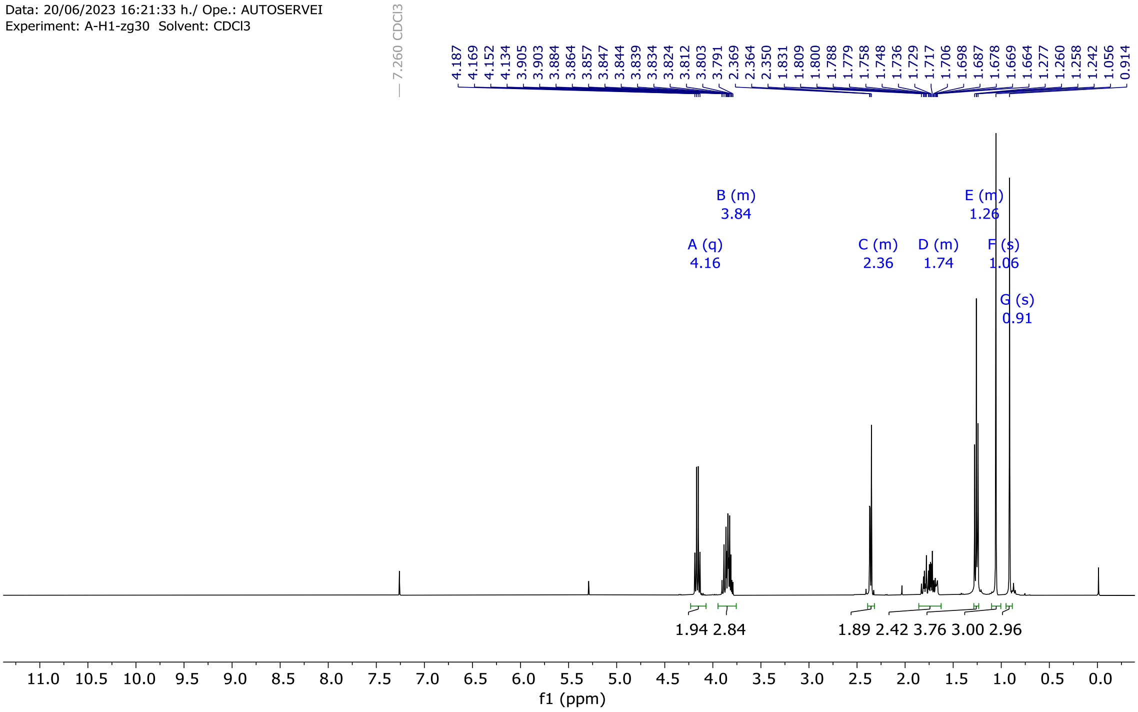

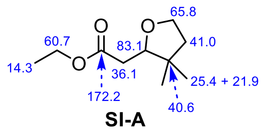

SI-A

23060491_B400FA_20062023_LRG411CH.2.fid 13C{1H} 101 MHz
Equip: B400F / N.Inv: 1037597
N.Reg: 23060491
Usuari: san / Mostra: LRG411CH
Nom: LAURA RODRIGUEZ GONZALEZ
Data: 20/06/2023 21:54:42 h./ Ope.: AUTOSERVEI
Experiment: A-C13-zgpg30 Solvent: CDCl3

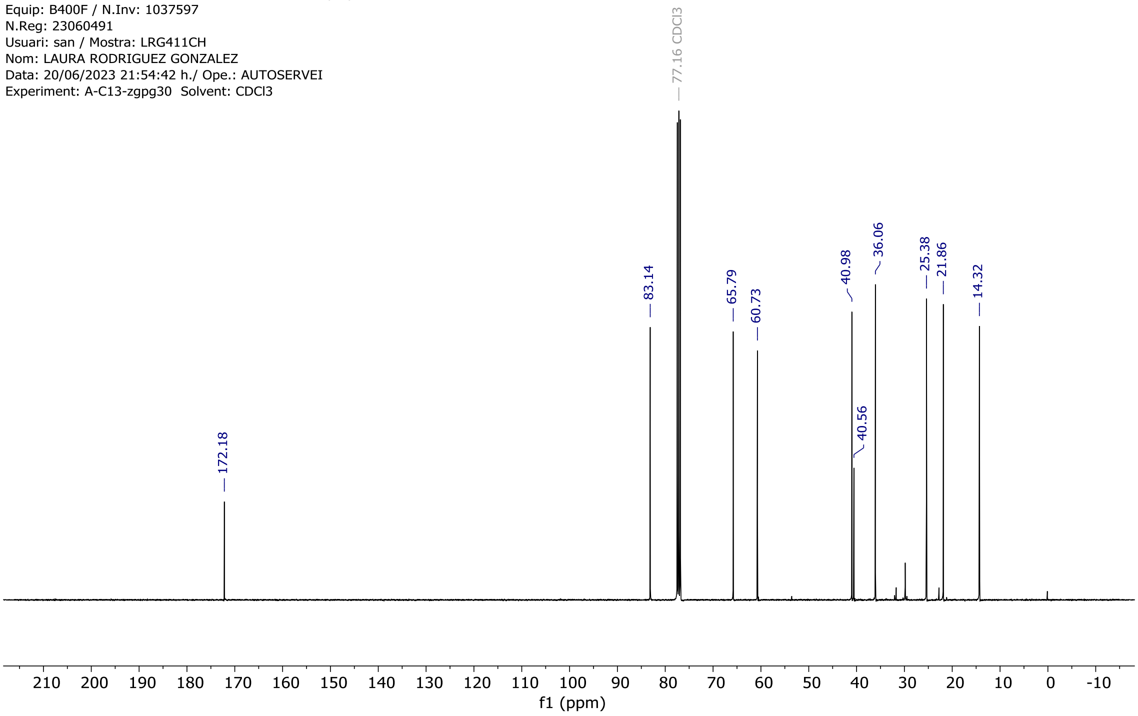

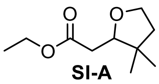

SI-A

2D-COSY

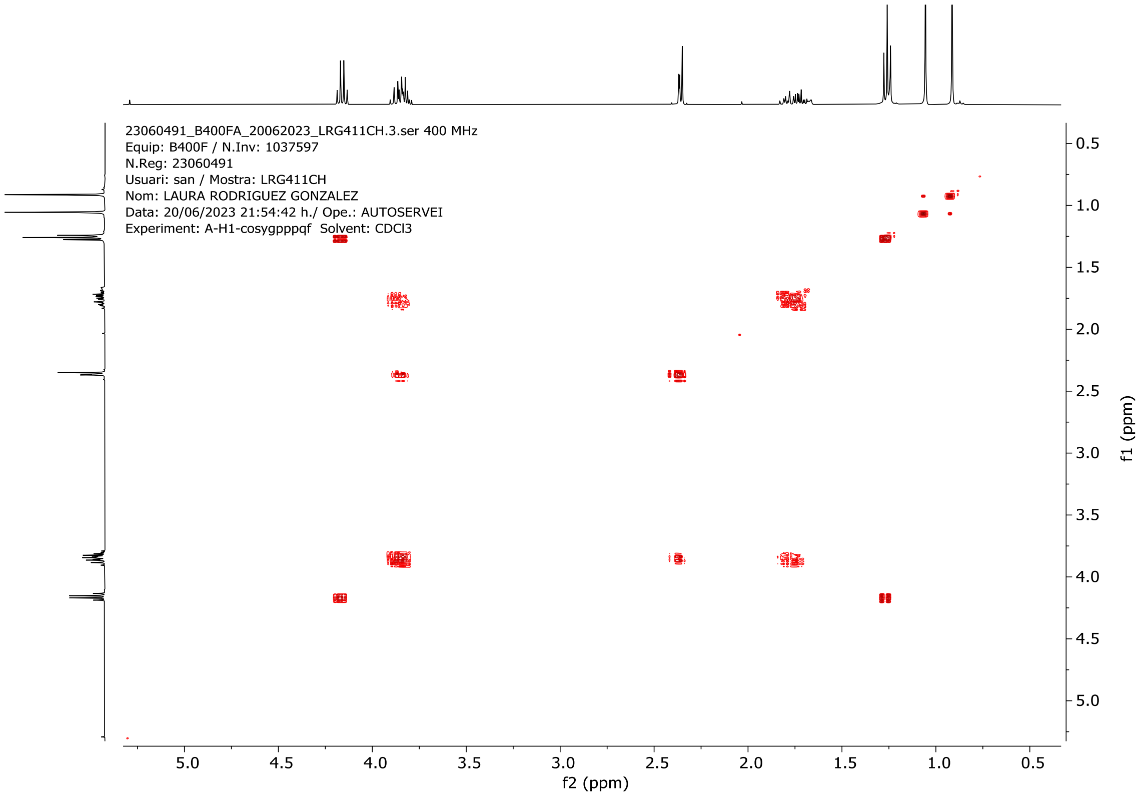

2D-HSQC

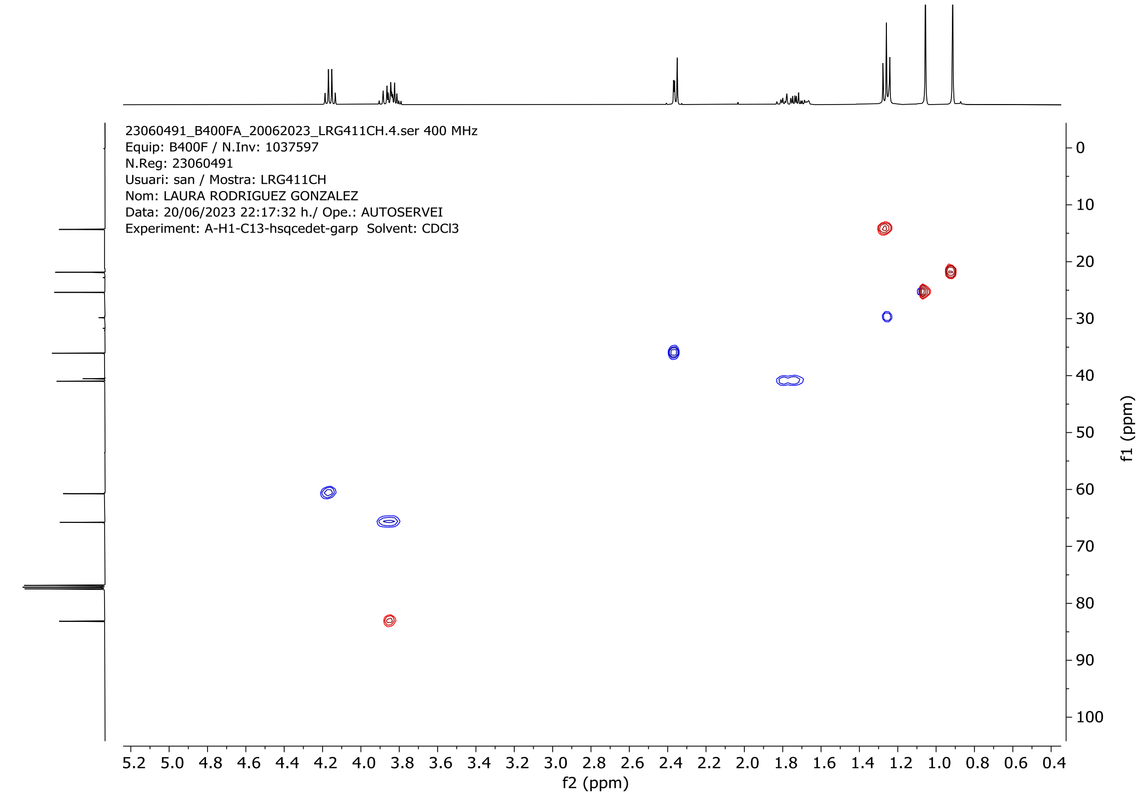

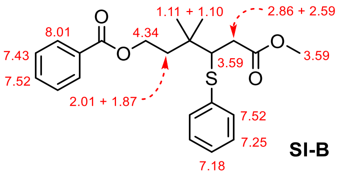

SI-B

23110265_B400FA_10112023_LRG498CH.1.fid 1H 400 MHz
Equip: B400F / N.Inv: 1037597
N.Reg: 23110265
Usuari: san / Mostra: LRG498CH
Nom: LAURA RODRIGUEZ GONZALEZ
Data: 10/11/2023 10:44:23 h./ Ope.: AUTOSERVEI
Experiment: A-H1-zg30 Solvent: CDCl3

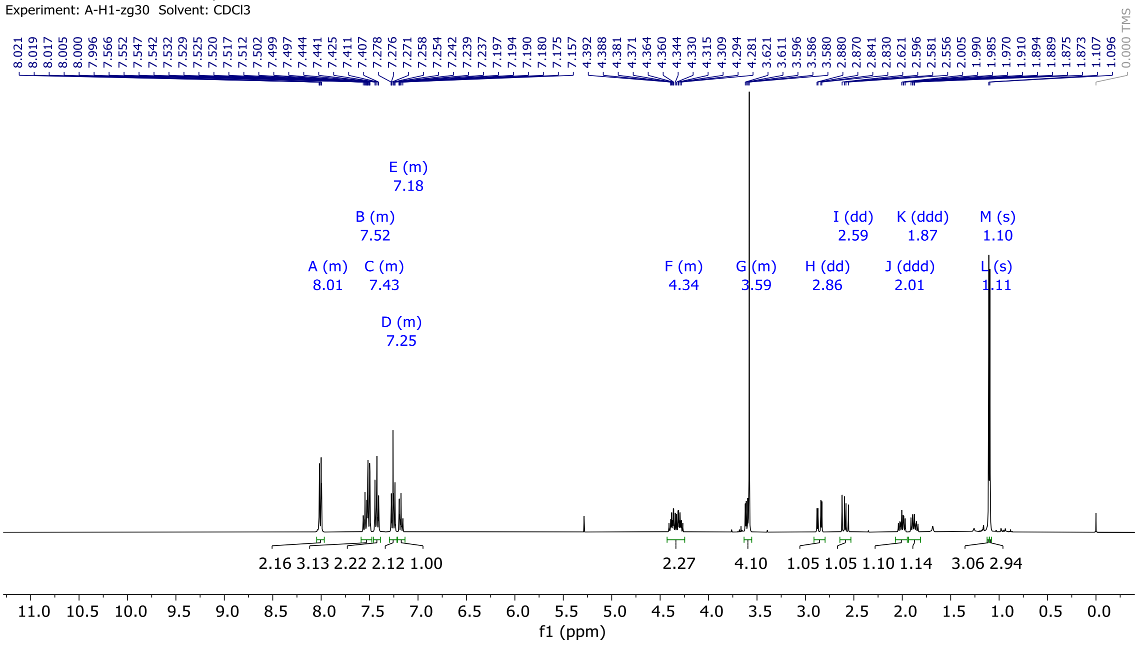

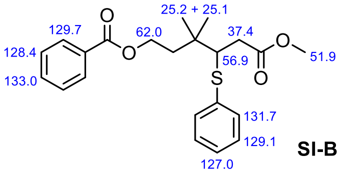

SI-B

23110265_B400FA_10112023_LRG498CH.2.fid 13C{1H} 101 MHz
Equip: B400F / N.Inv: 1037597
N.Reg: 23110265
Usuari: san / Mostra: LRG498CH
Nom: LAURA RODRIGUEZ GONZALEZ
Data: 10/11/2023 21:54:29 h./ Ope.: AUTOSERVEI
Experiment: A-C13-zgpg30 Solvent: CDCl3

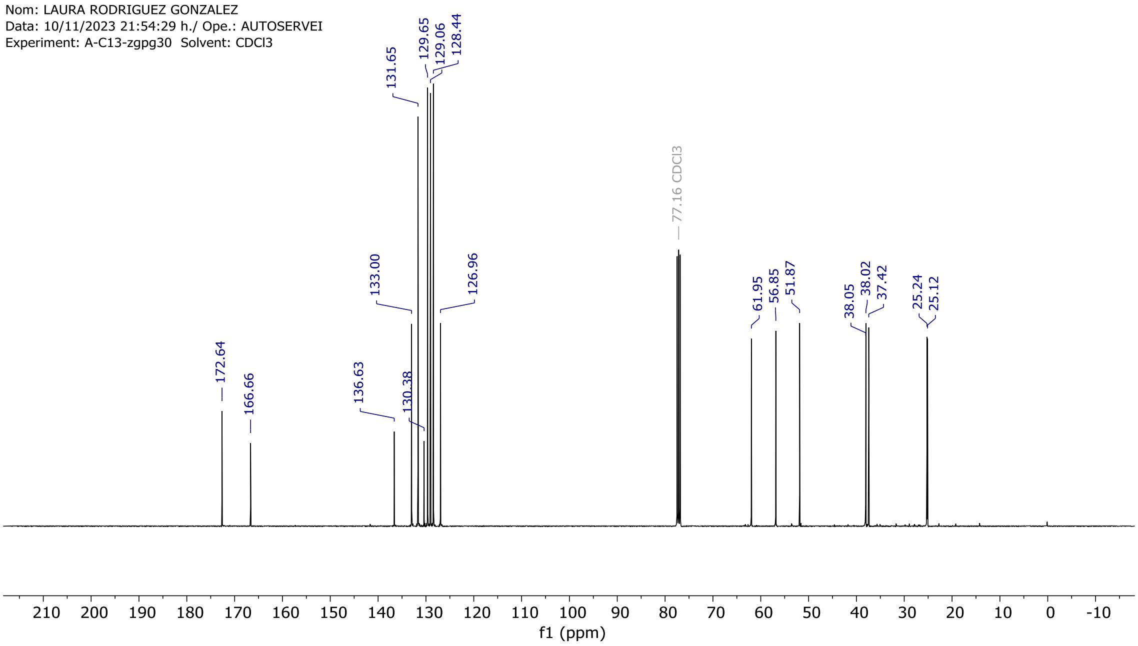

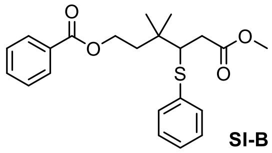

SI-B

2D-COSY

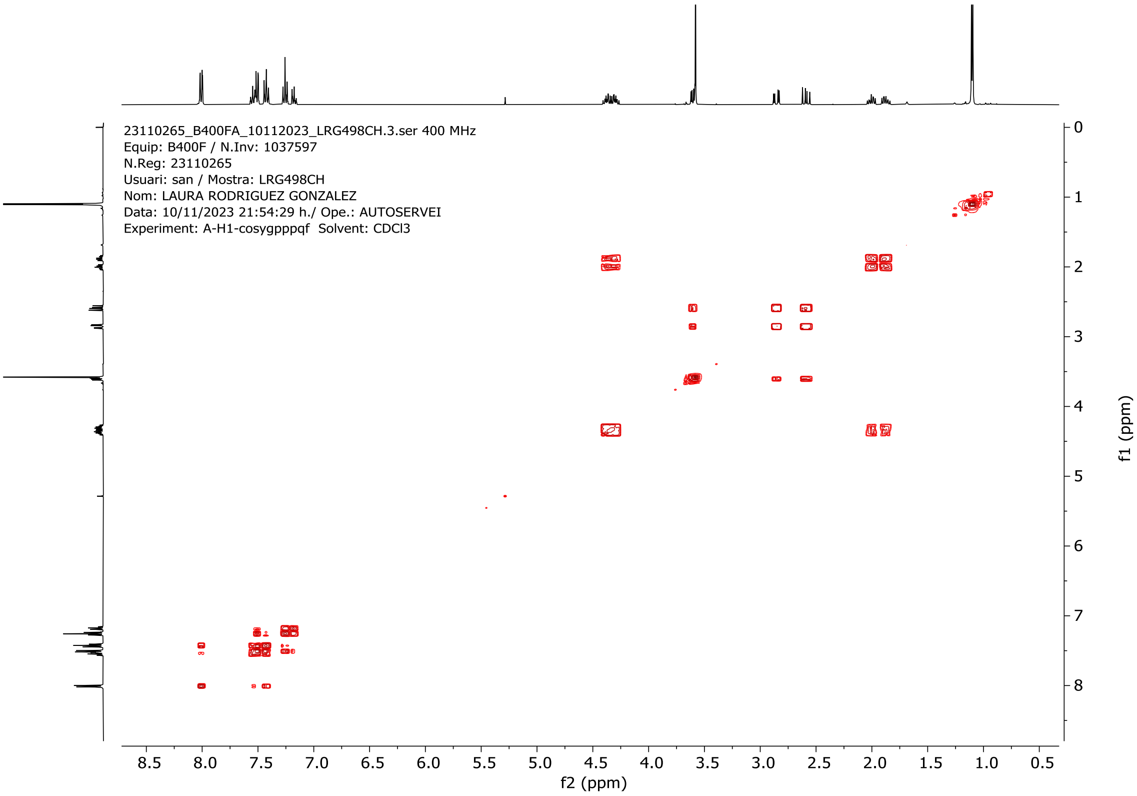

2D-HSQC

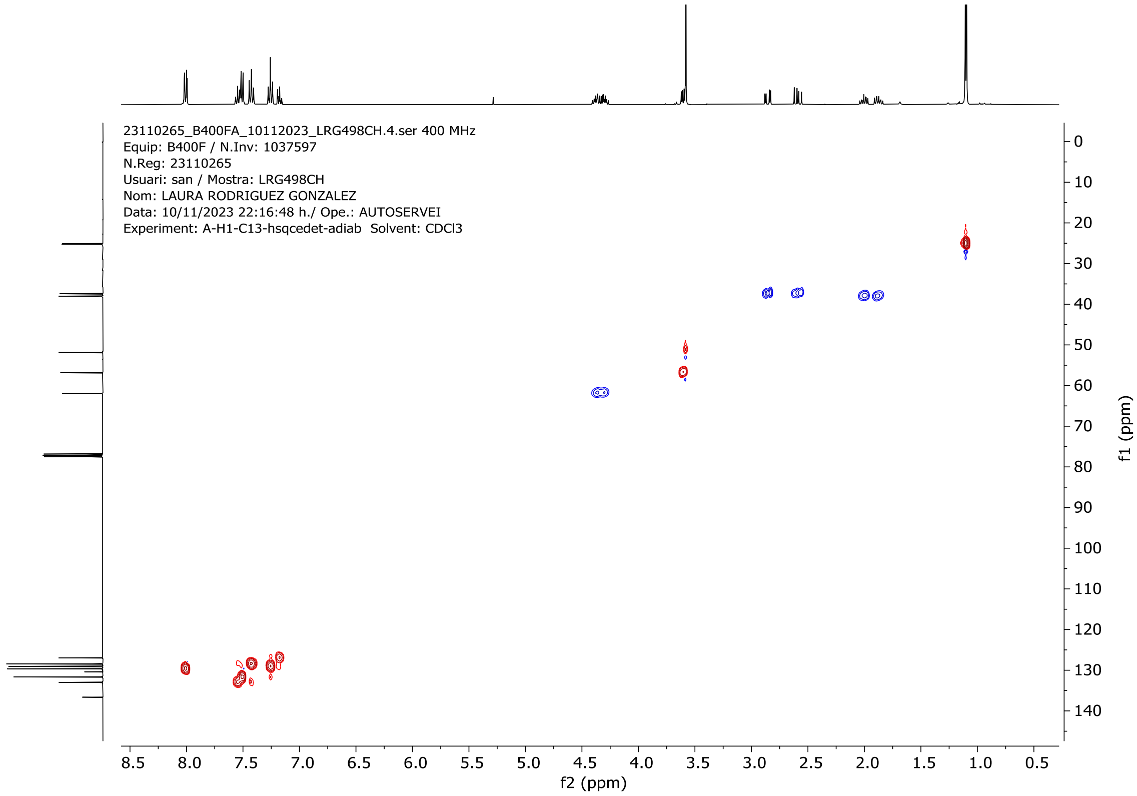

Supplement: Supplementary file 1 — ol4c03943_si_001.pdf [file ol4c03943_si_001.pdf]
